# Adverse events associated with immune checkpoint inhibitors in non-small cell lung cancer: a safety analysis of clinical trials and FDA pharmacovigilance system

**DOI:** 10.3389/fimmu.2024.1396752

**Published:** 2024-04-30

**Authors:** Xueyan Liang, Hewei Xiao, Huijuan Li, Xiaoyu Chen, Yan Li

**Affiliations:** ^1^Phase 1 Clinical Trial Laboratory, Guangxi Academy of Medical Sciences and the People’s Hospital of Guangxi Zhuang Autonomous Region, Nanning, China; ^2^Department of Scientific Research, Guangxi Academy of Medical Sciences and the People’s Hospital of Guangxi Zhuang Autonomous Region, Nanning, China; ^3^Department of Clinical Pharmacy, Guangxi Academy of Medical Sciences and the People’s Hospital of Guangxi Zhuang Autonomous Region, Nanning, China

**Keywords:** non-small cell lung cancer, pharmacovigilance, immune checkpoint inhibitors, real-world study, FAERS

## Abstract

**Objectives:**

Immune checkpoint inhibitors (ICIs) have revolutionized the treatment of non-small cell lung cancer (NSCLC). However, the application of ICIs can also cause treatment-related adverse events (trAEs) and immune-related adverse events (irAEs). This study was to evaluate both the irAEs and trAEs of different ICI strategies for NSCLC based on randomized clinical trials (RCTs). The study also examined real-world pharmacovigilance data from the Food and Drug Administration Adverse Event Reporting System (FAERS) regarding claimed ICI-associated AEs in clinical practice.

**Methods:**

Based on Pubmed, Embase, Medline, and the Cochrane CENTRAL, we retrieved RCTs comparing ICIs with chemotherapy drugs or with different ICI regimens for the treatment of NSCLC up to October 20, 2023. Bayesian network meta-analysis (NMA) was performed using odds ratios (ORs) with 95% credible intervals (95%CrI). Separately, a retrospective pharmacovigilance study was performed based on FAERS database, extracting ICI-associated AEs in NSCLC patients between the first quarter (Q1) of 2004 and Q4 of 2023. The proportional reports reporting odds ratio was calculated to analyze the disproportionality.

**Results:**

The NMA included 51 RCTs that involved a total of 26,958 patients with NSCLC. Based on the lowest risk of any trAEs, cemiplimab, tislelizumab, and durvalumab were ranked as the best. Among the agents associated with the lowest risk of grades 3-5 trAEs, tislelizumab, avelumab, and nivolumab were most likely to rank highest. As far as any or grades 3-5 irAEs are concerned, atezolizumab plus bevacizumab plus chemotherapy is considered the most safety option. However, it is associated with a high risk of grades 3-5 trAEs. As a result of FAERS pharmacovigilance data analysis, 9,420 AEs cases have been identified in 7,339 NSCLC patients treated with ICIs, and ICIs were related to statistically significant positive signal with 311 preferred terms (PTs), and comprehensively investigated and identified those AEs highly associated with ICIs. In total, 152 significant signals were associated with Nivolumab, with malignant neoplasm progression, death, and hypothyroidism being the most frequent PTs.

**Conclusion:**

These findings revealed that ICIs differed in their safety profile. ICI treatment strategies can be improved and preventive methods can be developed for NSCLC patients based on our results.

## 1 Introduction

The prevalence and mortality of lung cancer are among the highest in the world (1). Nearly 85% of lung cancers are non-small cell lung cancers (NSCLC), and about 30% of patients diagnosed with NSCLC have locally advanced disease (stage III) (2). Over the past decade, immunotherapy has demonstrated promise when it comes to treat NSCLC. There has been a significant transformation in the environment of therapeutic for variety of cancer types due to immune checkpoint inhibitors (ICIs), including agents that target the programmed death-ligand 1 (PD-L1), programmed death-1 receptor (PD-1), and cytotoxic T-lymphocyte-associated protein 4 (CTLA-4) (3).

It has been demonstrated that ICIs are related to superior survival outcomes among patients with NSCLC in randomized clinical trials (RCTs) as compared to chemotherapy drugs that have been traditionally used. ICIs have proven to provide survival benefits (2), but they can also cause adverse events (AEs), including cutaneous reactions, including morbilliform, psoriasiform, lichenoid, eczematous, and rash (4, 5). Due to their ability to block the pathways that are involved in regulating the immune system, ICIs may associate with high risk of immune-related adverse events (irAEs) by inducing inflammation in the organs (6). ICI-associated irAEs can potentially involve multiple system organ classes (SOC), including the skin (eg, rash and pruritus) (7), gastrointestinal tract (eg, diarrhea and colitis) (8), endocrine (eg, hypothyroidism and hypophysitis) (9), lung (eg, pneumonitis) (7), and psychiatric disorders (eg, delirium) (10). Without proper management, irAEs can be severe and life-threatening and may result in treatment discontinuation or failure.

In previously published network meta-analysis (NMA), irAEs related to ICI therapy were primarily examined, but no studies have examined irAEs related to System Organ Classes (SOC) and treatment-related adverse events (trAEs) (11–14). Also, most of these published studies did not specifically evaluate the risk of trAEs associated with different ICI regimens, which may vary depending on the regimen. Furthermore, pharmacovigilance data from real-world clinical practice were not evaluated simultaneously. The use of ICIs has revolutionized cancer treatment standards and significantly enhanced patient prognoses. However, the utilization of these groundbreaking therapies has led to the observation and reporting of various types of adverse events, commonly known as irAEs (15). We conducted a NMA evaluating both the irAEs and trAEs due to no head-to-head RCTs comparing different ICI strategies. The study also examined real-world pharmacovigilance data from the Food and Drug Administration (FDA) Adverse Event Reporting System (FAERS) regarding claimed ICI-associated AEs in clinical practice (16).

## 2 Methods

### 2.1 Study design

A NMA of RCTs was conducted to determine the risks associated with trAEs and irAEs in NSCLC patients receiving ICI therapy. The FAERS database was further analyzed retrospectively to determine the risk of AEs among NSCLC patients.

### 2.2 Systematic review procedures

#### 2.2.1 Data sources and searches

This study was registered in PROSPERO (CRD42022324171). The NMA was performed according to the PRISMA checklist (17). Pubmed, Embase, and Cochrane CENTRAL were systematic searched, with no language restrictions, up to October 20, 2023.

#### 2.2.2 Study selection and data extraction

We included RCTs that compared an ICI (eg, nivolumab, pembrolizumab) or a combination therapy of ICIs with (1) placebo, or (2) a chemotherapy drug (eg, carboplatin, cisplatin, pemetrexed, paclitaxel), or (3) a different ICI for patients with NSCLC. Data extraction was performed by two authors (XY L and HW X) independently. The third reviewer was responsible for adjudicating any disagreement between the two investigators (Y L). We obtained the following outcomes for each included study: (1) study information (ie, phase, design, source, and registered ID), (2) baseline characteristics (eg, sex, age, histology), and (3) interventions and outcomes (ie, population size, different treatment regimens, and different trAEs or irAEs number).

Two authors (HJ L and XY C) independently evaluated the risk-of-bias of included studies using the risk-of-bias tool 2.0 (18).

#### 2.2.3 Outcome measures

In this study, the primary outcome was any trAEs or irAEs (severity grade 1-5), as well as severe trAEs or irAEs (severity grade 3-5) (19). There were five grades: mild-to-moderate AEs (grades 1 and 2), severe or medically significant but not immediately AEs (grade 3), life-threatening AEs (grade 4), and death-related AEs (grade 5) (20). In NMA, trAEs and irAEs were defined according to how each RCT reported its trAEs and irAEs. There was a strong association between trAEs and irAEs and the intervention treatment in the trial. Additionally, secondary outcomes included trAEs and irAEs specific to each SOC. AEs are standardized using the SOC of the Medical Dictionary for Regulatory Activities (MedDRA) terminology (21), which contains 27 SOCs. Consequently, MedDRA (version 25.0) was used to categorize AEs to their related SOC levels for each report.

#### 2.2.4 Data Synthesis and *statistical analysis*

Data synthesis was performed using the “gemtc” package and a Bayesian NMA using R software (R Foundation for Statistical Computing, Vienna, Austria). Network plots of different treatment regimens were carried out using the “netmeta” package. We used a Markov chain Monte Carlo simulation using vague priors with four chains, with a burn-in period of 50,000 iterations, followed by 500,000 iterations. An odds ratio (OR) with a 95% credible interval (CrI) was reported as the effect estimate of the NMA. Matrix plots were used to illustrate the NMA estimates. To depict the ranking of the interventions, the surface under the cumulative ranking curve (SUCRA) was calculated. A narrative review was used when it was not possible to synthesize the data from the trials in an NMA. Among the treatments, the one associated with the lowest AE risk was ranked as the superior treatment regimen.

### 2.3 Pharmacovigilance study procedures

#### 2.3.1 Data source

As part of our pharmacovigilance study, we examined AEs related to ICIs in patients with NSCLC using the FAERS database, which is a publicly accessible database of safety reports (22). ICIs and NSCLC were obtained as search items to search report data of ICIs and NSCLC from the FAERS between the decade quarter (Q1) of 2004 and Q4 of 2023. We included only cases in which ICIs for the therapy of NSCLC patients were used by the primary suspect (PS). The FAERS database also codes reported AEs according to preferred term (PT) codes from the MedDRA, which are logically categorized into five levels. PTs represent distinct descriptions of a single medical concept. The hierarchy also includes “high-level terms” (HLTs) and “high-level group terms” (HLGTs). SOCs are further divided into aetiologies, sites of presentation, and purposes of HLGTs. It is important to note that different PTs are classified into different SOCs, but unique primary SOC can be connected, a characteristic known as multiaxiality. MedDRA (version 25.0) was used and PTs with primary SOC = “Yes” were obtained and analyzed, thereby ensuring that the PTs analyzed were clinically relevant AEs.

#### 2.3.2 Signal mining

It is commonly used in pharmacovigilance studies as a method for evaluating potential associations between different types of AE and a particular drug, which can then be evaluated clinically through the assessment of individual case reports (23). Reporting odds ratio (ROR) reflects the likelihood of a particular drug being reported for a specific event of interest in comparison to the likelihood of reporting events for other drugs in the FAERS database (24), and ROR was calculated in this study to evaluate AE signals in ICI reports among NSCLC patients (10). Using full AE reports of NSCLC patients from the FAERS database as comparators, we conducted disproportionality analysis to evaluated possible relationship between AEs and ICIs in NSCLC patients by the ROR (25). Initially, we created a drug AE contingency table and then calculated ROR based on that table before performing the disproportionality analysis of ICI-related AE (26). It was determined that an AE signal was related to different ICI regimens if there were at least three reports of PT levels AEs, and the lower limit of the 95% CI of the ROR did not fall below one.

#### 2.3.3 Descriptive analysis

In our study, we examined the clinical characteristics of NSCLC patients resulting from ICI-related AEs, including sex, age, age group, country, outcome, the most recent FDA acceptance year, the ICI regimens, the priority of the case, and the type of report. Death, disability, and life-threatening outcomes were reported as serious outcomes. Data importing and analysis were conducted using PostgreSQL (version 14) and R, based on previous published literature (26).

## 3 Results

### 3.1 Characteristics and quality of included studies

After searching the databases for 7,068 articles, 51 RCTs were included in the NMA, which included 26,958 patients (**Supplementary Figure 1**) (27–77). We summarize the detailed baseline characteristics of the included studies in **Supplementary Table 1**. A total of 34 studies showed a low risk of bias, while other studies had a ‘some concerns’ risk of bias in randomization and deviations from the intended intervention, as depicted in **Supplementary Table 2**.

### 3.2 Network meta-analysis outcomes

#### 3.2.1 Primary outcomes: ranking the probability of treatment regimen

**Figure 1:**
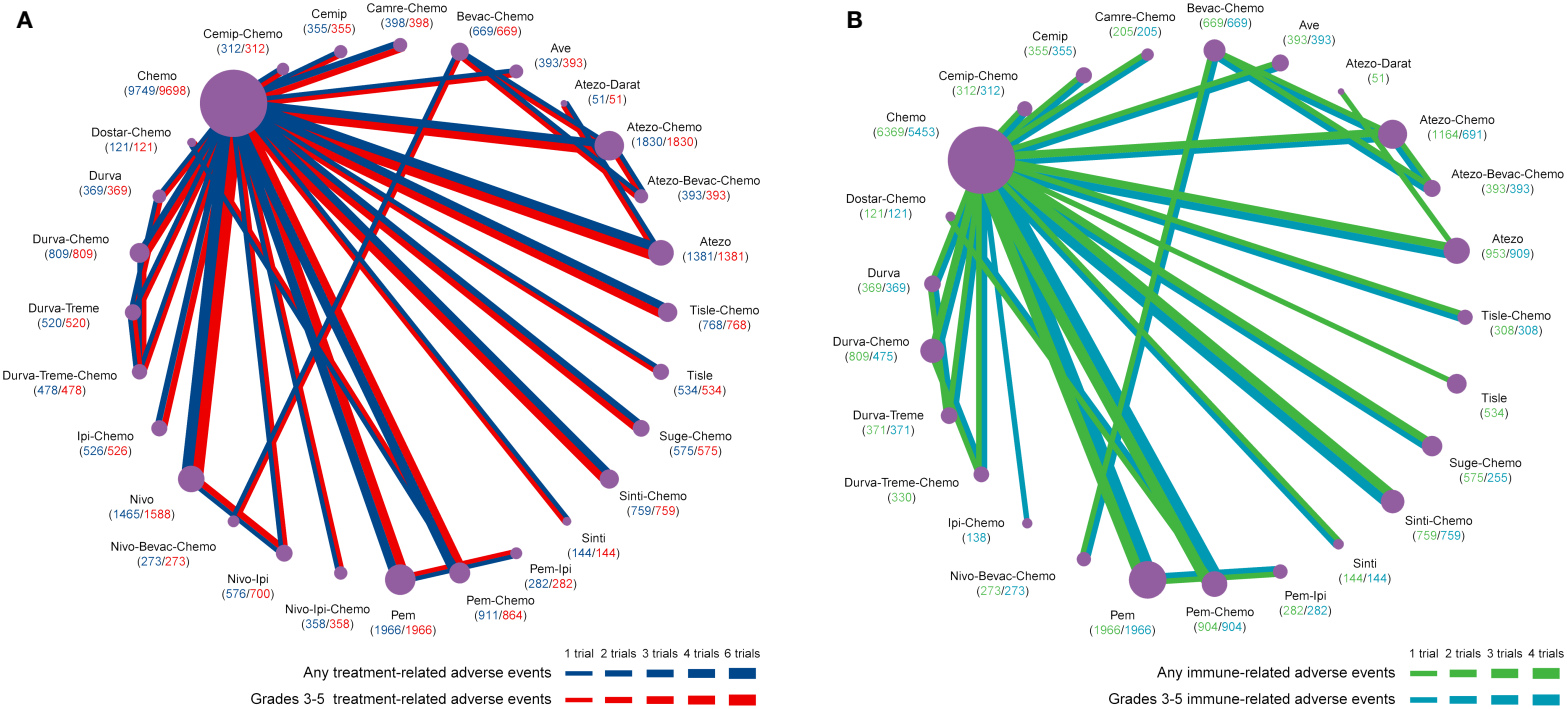
Network plots of adverse events of ICIs for NSCLC. **(A)** Comparisons were performed on any and grades 3-5 treatment-related adverse events. **(B)** Comparisons were performed on any and grade 3-5 immune-related adverse events. Atezo, atezolizumab; Ave, avelumab; Beva, bevacizumab; Camre, camrelizumab; Cemip, cemiplimab; Chemo, chemotherapy; Darat, daratumumab; Dostra, dostarlimab; Durva, durvalumab; ICI, immune checkpoint inhibitor; Ipi, ipilimumab; Nivo, nivolumab; NSCLC, non-small cell lung cancer; Pem, pembrolizumab; Sint, sintilimab; Sugema, sugemalimab; Tisle, tislelizumab; Treme, tremelimumab.

**Figure 2:**
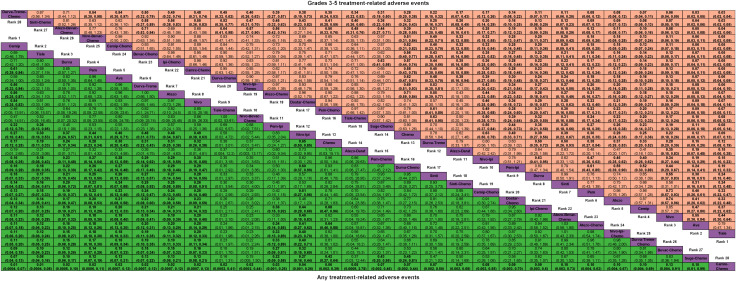
Odds ratio (95% CrI) of any and grades 3-5 treatment-related adverse events associated with each treatment regimen. Atezo, atezolizumab; Ave, avelumab; Beva, bevacizumab; Camre, camrelizumab; Cemip, cemiplimab; Chemo, chemotherapy; CrI, credible interval; Darat, daratumumab; Dostra, dostarlimab; Durva, durvalumab; Ipi, ipilimumab; Nivo, nivolumab; Pem, pembrolizumab; Sint, sintilimab; Sugema, sugemalimab; Tisle, tislelizumab; Treme, tremelimumab.

**Figure 3:**
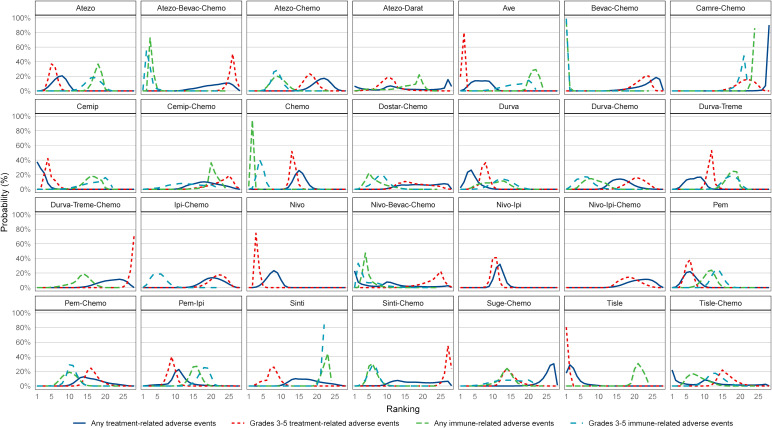
Ranking of the probability of being the best treatment regimen. Atezo, atezolizumab; Ave, avelumab; Beva, bevacizumab; Camre, camrelizumab; Cemip, cemiplimab; Chemo, chemotherapy; CrI, credible interval; Darat, daratumumab; Dostra, dostarlimab; Durva, durvalumab; Ipi, ipilimumab; Nivo, nivolumab; Pem, pembrolizumab; Sint, sintilimab; Sugema, sugemalimab; Tisle, tislelizumab; Treme, tremelimumab.

For any or grade 3-5 trAEs, network plots are displayed in **Figure 1A** and each comparison results of ORs and 95% Crls are revealed in **Figure 2**, and each rank probability of each ICI are shown in **Figure 3**. Regarding any trAEs, cemiplimab (Cemip), tislelizumab (Tisle), and durvalumab (Durva) were the lowest risk of any trAEs are likely to rank as the best following by pembrolizumab (Pem), avelumab (Ave), Durva plus tremelimumab (Durva-Treme), atezolizumab (Atezo) and nivolumab (Nivo), and there was a greater degree of safety than most other ICIs (ORs ranging between 0.01 and 0.61), whereas camrelizumab plus chemotherapy (Camre-Chemo) and sugemalimab plus chemotherapy (Suge-Chemo) were among the least safety drugs. In terms of grades 3-5 trAEs, Tisle, Ave, and Nivo were most likely to rank highest due to their association with the lowest risk of grades 3-5 trAEs following by Cemip, Atezo, Pem, sintilimab (Sinti), and Durva and they were more safety than most of other ICIs (ORs ranging between 0.04 and 0.74), whereas Durva-Treme-Chemo, Sinti-Chemo, and atezolizumab plus bevacizumab plus chemotherapy (Atezo-Bevac-Chemo) were among the least safety ICIs.

**Figure 4:**
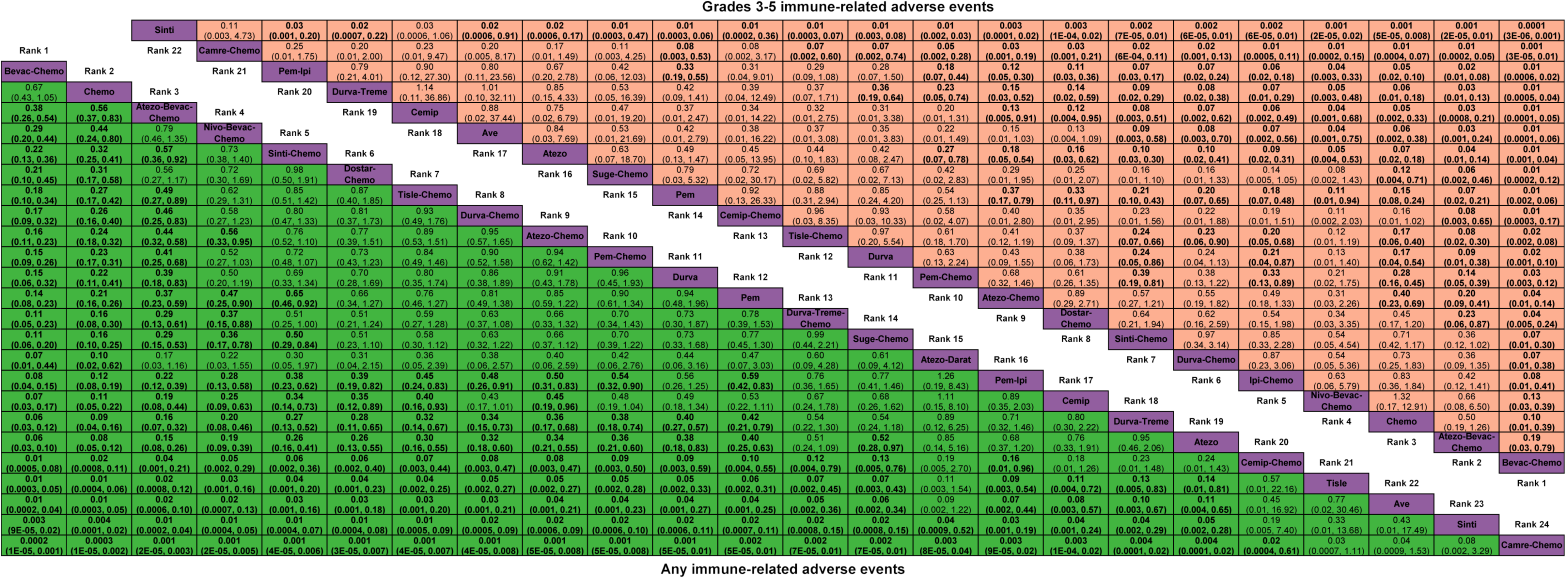
Odds ratio (95% CrI) of any and grades 3-5 immune-related adverse events associated with each treatment regimen. Atezo, atezolizumab; Ave, avelumab; Beva, bevacizumab; Camre, camrelizumab; Cemip, cemiplimab; Chemo, chemotherapy; CrI, credible interval; Darat, daratumumab; Dostra, dostarlimab; Durva, durvalumab; Ipi, ipilimumab; Nivo, nivolumab; Pem, pembrolizumab; Sint, sintilimab; Sugema, sugemalimab; Tisle, tislelizumab; Treme, tremelimumab.

Network plots of any or grade 3-5 irAEs are displayed in **Figure 1B** and each comparison results of ORs and 95% Crls are displayed in **Figure 4**, and each rank probability of each ICI are displayed in **Figure 3**. Regarding any irAEs, Bevac-Chemo, Chemo, and Atezo-Bevac-Chemo associated with the lowest risk and provided greater safety than most of the other ICIs (ORs ranging between 0.0003 and 0.57), they were most likely to rank as the best, whereas Camre-Chemo, Sinti, and Ave were among the least safety ICIs. In terms of grades 3-5 irAEs, Bevac-Chemo, Atezo-Bevac-Chemo, and Chemo associated with the lowest risk and were safer than most other ICIs (ORs ranging between 0.0001 and 0.40), they were most likely to rank as the best, whereas Sinti, Camre-Chemo, and Pem plus ipilimumab (Pem-Ipi) were among the least safety ICIs.

#### 3.2.2 Secondary outcomes: ranking the probability of treatment regimen

The ranking probability of an ICI treatment regimen having the lowest risk is dependent on the SOC, as well as on the irAE and trAE. We describe herein the secondary outcomes that are statistically significant. We evaluated trAEs using 13 SOCs and irAEs using seven SOCs in this study. Network plots of secondary outcomes are shown in **Supplementary Figures 2**, **3** and each comparison results of ORs and 95% Crls are shown in **Supplementary Figures 4**, **5**, and each rank probability of each ICI regimen are shown in **Supplementary Figures 6**, **7**.

First, we evaluated the trAEs of different SOCs. Sinti, Cemip, and Atezo-Chemo were ranked as having the lowest risk of blood and lymphatic system disorders (ORs ranging between 0.0002 and 0.64), whereas Durva-Treme-Chemo was the least safe. Chemo had the lowest risk of endocrine disorders, followed by Suge-Chemo and Atezo-Chemo (ORs ranging between 0.008 and 0.26), whereas Nivo-Ipi-Chemo and Nivo-Ipi was the lowest safety regimen. There was the lowest risk of gastrointestinal disorders related to Durva, Nivo, Durva-Treme, and Pem (ORs ranging from 0.02 to 0.63), whereas Pem-Chemo appeared to be among the most unsafe ICI regimens. The lowest-ranking ICIs in terms of general disorders and administration site conditions were Cemip, Nivo, Ave, and Durva (ORs ranging from 0.18 to 0.69), while Durva-Treme-Chemo was considered one of the least safe ICIs. It was noted that Atezo, Nivo-Ipi, and Tisle were ranked as the lowest risk ICIs in terms of investigations (ORs ranging from 0.002 to 0.42) while Cemip-Chemo was ranked as the least safe ICI. Among the ICIs tested for metabolism and nutrition disorders, Tisle, Durva, and Cemip displayed the lowest risks (ORs ranging from 0.15 to 0.59), whereas Tisle-Chemo presented the lowest level of safety. It was found that Ave had the lowest risk of musculoskeletal and connective tissue disorders (ORs between 0.01 and 0.30), whereas Atezo-Chemo had the least safety. In terms of nervous system disorders, Pem, Cemip and Nivo were most likely to rank as the lowest risk (ORs ranging between 0.02 and 0.49), whereas Atezo-Chemo was among the least safety ICIs. Pem-Chemo has the lowest risk of respiratory, thoracic, and mediastinal disorders (ORs ranging from 0.01 to 0.78), whereas Pem-Ipi has the greatest risk. Ave, Tisle and Atezo were most likely to rank as the lowest risk ICIs in terms of skin and subcutaneous tissue disorders (ORs ranging from 0.0004 to 0.05), whereas Nivo-Ipi-Chemo ranked among the lowest in safety terms. Ave (ORs ranging between 0.01 and 0.15), was the ICI regimen associated with the lowest risks of infections and infestations, whereas Atezo-Darat was among the least safe.

Second, we evaluated the irAEs of different SOC. Among the ICIs, Durva was associated with a low risk of endocrine disorders, however, most of the comparisons did not reveal any significant difference, whereas Nivo-Ipi-Chemo was associated with a low safety profile. There was a low rate of gastrointestinal disorders associated with Nivo (ORs ranging between 0.01 and 0.64) while Nivo-Ipi-Chemo was one of the least safely associated ICIs. Chemo and Nivo were associated with the least risk of hepatobiliary disorders, however, most comparisons did not result in a significant difference, whereas Camre-Chemo was associated with the least safety. Pem was related to the lowest risk of injury, poisoning, and procedural complications (ORs ranging between 0.04, 0.37), whereas Atezo-Chemo was related to the lowest risk of safety. The lowest risk of investigations was reported for Chemo, followed by Atezo-Chemo and Sinti-Chemo (ORs ranging from 0.01 to 0.55), whereas Camre-Chemo had the lowest safety rating. As far as respiratory, thoracic, and mediastinal disorders are concerned, Chemo was the best performing ICI, followed by Durva (ORs varying between 0.04 and 0.48), while Camre-Chemo was the least safe. Chemo had the lowest incidence of skin and subcutaneous tissue disorders, followed by Tisle-Chemo and Pem-Chemo (ORs ranging from 0.01 to 0.48), whereas Nivo-Ipi-Chemo had the lowest incidence.

#### 3.2.3 Heterogeneity and inconsistency

The results of this study indicate that there is favorable transitivity and consistency across the included trials, which allows for direct and indirect comparisons to be made. Based on the Q test and the *I^2^* statistic, it was found that most of the heterogeneity and inconsistency were minimal (*I^2 ^* 0%) or low (*I^2^* < 25%) across the included studies (**Supplementary Figure 8**).

### 3.3 Pharmacovigilance analysis

#### 3.3.1 Adverse events among ICI NSCLC patients in the FDA adverse events reporting system

FAERS contained 23,941 reports related to ICI immunotherapy for NSCLC patients (**Supplementary Table 3**). Statistics on AEs in patients treated with ICIs in cases with NSCLC were obtained after excluding cases that occurred as a result of concomitant medications, adverse reactions and related treatment indications. A relatively constant number of cases were reported each year. Patients with NSCLC experienced AEs differently according to their ICI treatment strategy. Considering the proportion of AEs following various ICI treatment strategies, it appears that ICIs may contribute to a significant portion of AEs.

#### 3.3.2 Scanning for ICI-related adverse events

**Figure 5:**
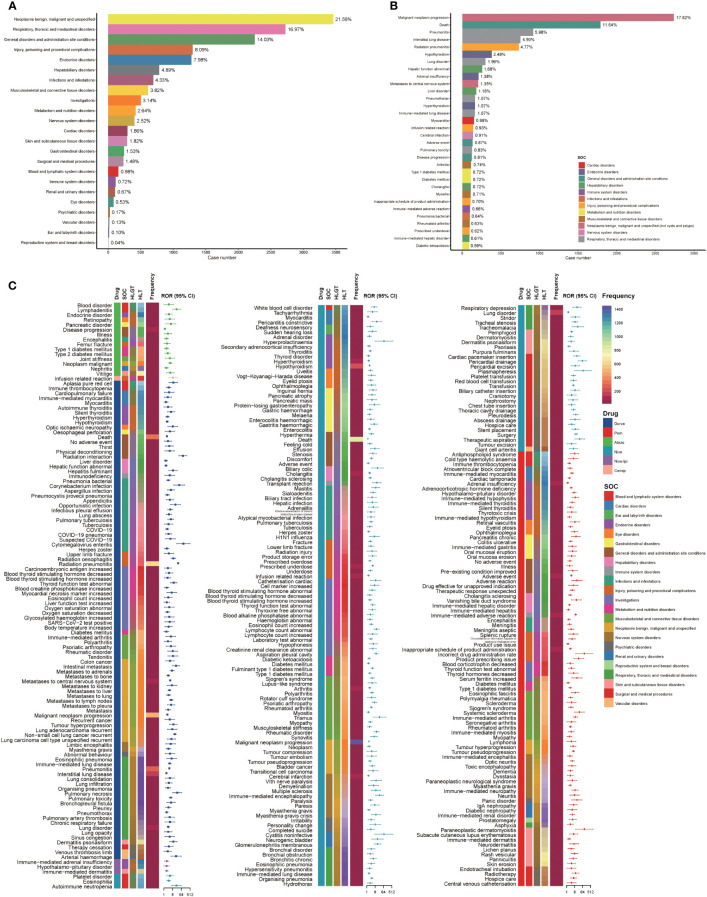
Scanning for ICI-related adverse events based on the FAERS database. **(A)** Bar plot shows the statistics of SOC regarding PTs of adverse events. The percentage values labeled in the figure represent the proportion of cases with such adverse events out of the total ICI-related AE cases. **(B)** Bar plot shows the statistics of the top 10% PTs of adverse events. The color indicates the SOC of the corresponding PT. The percentage values labeled in the figure represent the proportion of cases with such adverse events out of the total ICI-related adverse event cases. **(C)** The heatmap and forest plot shows the ROR for 311 adverse events (with cases no less than 3 and the lower limit of the 95% CI of the ROR exceeds one) in the FAERS database under different ICI treatment strategies (including Atezo, Ave, Durva, Nivo, Nivo-Ipi). The color indicates the ICI drugs, SOC, HLGT, HLT of the corresponding PT, and the frequency of PTs. This figure also depicted the hierarchical relationship of PTs for categories of ICI-related AEs in MedDRA. Due to the limitation of the figure, the legends of HLT and HLGT were provided in **Supplementary Figure 9**. Atezo, atezolizumab; Ave, avelumab; CI, confidence interval; Durva, durvalumab; FAERS, FDA Adverse Event Reporting System; HLGT, high level group term; HLT, high level term; ICI, immune checkpoint inhibitor; Ipi, ipilimumab; Nivo, nivolumab; PT, preferred term; ROR, reporting odds ratio; SOC, systemic organ class.

Based on the reports of ICIs in NSCLC patients, we counted the types and the number of AEs (**Supplementary Table 4**). Malignant neoplasm progression (N = 3,337, 5.27%), death (N = 1,978, 3.12%), pneumonitis (N = 1,093, 1.73%), pneumonia (N = 1,087, 1.72%), pyrexia (N = 1,042, 1.65%) were the five most categories of AEs. Using the full AEs of NSCLC patients in the FAERS database as a comparator, we calculated the ROR of PTs with no less than three cases (N = 3,532) in the AEs. NSCLC patients with PTs that met the aforementioned conditions were considered to have ICI-related AEs. ICI reports with 9,420 AEs related to ICI were reviewed for further analysis in NSCLC patients (N = 7,339). We found that 6 types of ICIs either monotherapy or in combination related to a statistically significant positive signal in 311 PTs and 23 different SOCs, after filtering by our criteria for a valid signal (**Figure 5**). In cases involving ICI-related AEs, the most common concomitant AEs were neoplasms benign, malignant and unspecified, respiratory, thoracic and mediastinal disorders, and general disorders and administration site conditions. Only 0.72% of ICI-related AEs occurred in conjunction with immune system disorders (**Figure 5A**). Furthermore, malignant neoplasm progression (17.82%), death (11.64%), and pneumonitis (5.98%) were among the top 10% of ICI-related AEs (**Figure 5B**). In addition, a comparison of the ROR of different ICI monotherapy or combination therapy for different ICI-associated AEs is shown in **Figure 5C**. Based on the disproportionality analysis, there were 152 significant safety signals for Nivo, 98 for Durva, 89 for Pem, 15 for Atezo, 3 for Nivo-Ipi, and 1 for Cemip within the PTs, and those 311 PTs linked to 23 SOCs. As a result of disproportionality analysis of PTs linked to SOCs, there were 22 significant safety signals for Nivo and 20 for Durva, 21 for Pem, 12 for Atezo, 2 for Nivo-Ipi and 1 for Cemip. Upon further analysis of the detected safety PTs signals, it was determined that the following AEs were most frequently reported: for Nivo, malignant neoplasm progression (N = 1,439), death (N = 858) and hypothyroidism (N = 196); for Durva, radiation pneumonitis (N = 601) and malignant neoplasm progression (N = 469); for Pem, immune-mediated adverse reaction (N = 86); for Atezo, disease progression (N = 104). SOC signals were most frequently associated with the following: for Nivo, neoplasms benign, malignant and unspecified (N = 1,509) and general disorders and administration site conditions (N = 970); for Durva, neoplasms benign, malignant and unspecified (N = 782) and respiratory, thoracic and mediastinal disorders (N = 644); for Pem, endocrine disorders (N = 167); for Atezo, general disorders and administration site conditions (N = 109).

#### 3.3.3 Descriptive analysis of cases with ICI-related adverse events and comparison between the fatal and non-fatal groups

The clinical characteristics of the ICI-related AEs reported in the FAERS database were analyzed statistically following the screening of the reports of ICIs in the FAERS database (N = 7,339) (**Table 1**). Data from 5,391 cases reports showed that the median age of the patients was 69 years old (interquartile range [IQR] 61-75). There are a large number of reported male cases (N =4,517, 69%) and most of cases are reported from Japan (N = 2,814, 38%). 37.63% (N =2,762) Cases occurred fatal related outcomes.

**Table 1:** Characteristics of reports with ICI-related adverse events of NSCLC patients sourced from the FAERS database.

Clinical characteristics	Overall (N = 7,339)	Fatal (N = 2,762)	Non-fatal (N = 4,577)	P-value
**Gender**				`<0.001
Male	4,517 (69%)	1,797 (72%)	2,720 (68%)	
Female	2,007 (31%)	709 (28%)	1,298 (32%)	
Missing	815	256	559	
**Age**				0.016
Median (IQR)	69 (61, 75)	68 (60, 75)	69 (61, 76)	
Missing	1,948	739	1,209	
**Age group**				0.062
18–64	1,914 (36%)	752 (37%)	1,162 (35%)	
65–75	1,965 (36%)	738 (36%)	1,227 (36%)	
≥75	1,512 (28%)	533 (26%)	979 (29%)	
Missing	1,948	739	1,209	
**Country**				<0.001
Japan	2,814 (38%)	824 (30%)	1,990 (43%)	
United States of America	1,199 (16%)	380 (14%)	819 (18%)	
Germany	312 (4.3%)	146 (5.3%)	166 (3.6%)	
France	302 (4.1%)	95 (3.4%)	207 (4.5%)	
Canada	257 (3.5%)	199 (7.2%)	58 (1.3%)	
Australia	217 (3.0%)	141 (5.1%)	76 (1.7%)	
Italy	208 (2.8%)	73 (2.6%)	135 (2.9%)	
United Kingdom of Great Britain and Northern Ireland	77 (1.0%)	32 (1.2%)	45 (1.0%)	
Other country	1,953 (27%)	872 (32%)	1,081 (24%)	
**Received year**				<0.001
2014	1 (<0.1%)	0 (0%)	1 (<0.1%)	
2015	171 (2.3%)	98 (3.5%)	73 (1.6%)	
2016	785 (11%)	456 (17%)	329 (7.2%)	
2017	1,009 (14%)	525 (19%)	484 (11%)	
2018	981 (13%)	372 (13%)	609 (13%)	
2019	1,332 (18%)	398 (14%)	934 (20%)	
2020	1,046 (14%)	350 (13%)	696 (15%)	
2021	875 (12%)	234 (8.5%)	641 (14%)	
2022	631 (8.6%)	191 (6.9%)	440 (9.6%)	
2023	508 (6.9%)	138 (5.0%)	370 (8.1%)	
**Case priority**				<0.001
Direct	32 (0.4%)	7 (0.3%)	25 (0.5%)	
Expedited	6,956 (95%)	2,747 (99%)	4,209 (92%)	
Non-expedited	351 (4.8%)	8 (0.3%)	343 (7.5%)	
**Reporter type**				<0.001
Consumer	1,790 (26%)	848 (33%)	942 (22%)	
Healthcare professional	5,125 (74%)	1,752 (67%)	3,373 (78%)	
Missing	424	162	262	
**Treatment strategy**				<0.001
Nivo	3,989 (54%)	1,956 (71%)	2,033 (44%)	
Durva	2,064 (28%)	561 (20%)	1,503 (33%)	
Pem	1,029 (14%)	154 (5.6%)	875 (19%)	
Atezo	205 (2.8%)	87 (3.1%)	118 (2.6%)	
Nivo-Ipi	49 (0.7%)	4 (0.1%)	45 (1.0%)	
Cemip	3 (<0.1%)	0 (0%)	3 (<0.1%)	

Atezo, atezolizumab; Ave, avelumab; Durva, durvalumab; FAERS, FDA Adverse Event Reporting System; ICI, immune checkpoint inhibitor; Ipi, ipilimumab; Nivo, nivolumab; NSCLC, non-small cell lung cancer; Pem, pembrolizumab.

Based on the proportion of ICI-related fatal related outcomes. A comparison of the outcomes of the fatal and non-fatal groups may provide further information regarding how to improve fatal related outcome in NSCLC patients. Compared with females, there is a greater proportion of males in the fatal group (P < 0.001). A significant difference exists between the fatal and non-fatal groups in the proportion of cases treated with different types of ICIs on a monotherapy or combination basis (P < 0.001), and Nivo and Atezo was related to increased risk of death. The age group of the fatal and non-fatal groups did not differ significantly (P = 0.062, respectively).

## 4 Discussion

AEs have been reported following increased ICI use, prompting additional investigations. This study is the first to analyze AEs associated with ICIs in NSCLC in a comprehensive manner, using data from both clinical trials and FAERS real-world data. A total of 26,958 patients from 51 RCTs for NSCLC were included in NMA. Through disproportionality analysis of the full list of AEs reported in NSCLC patients of FAERS database, we identified AEs that were highly related to the ICI regimens, and evaluated the characteristics of NSCLC patents of these events.

The NMA revealed several key findings related to ICI regimens and the risk of AEs among patients with NSCLC. First, Cemip and Tisle are related to the lowest risk of any or grades 3-5 trAEs. The Durva-Treme-Chemo regimen, on the other hand, was associated with higher risks than the other ICI regimens. Second, Atezo-Bevac-Chemo was related to a low risk of any or grades 3-5 irAE, but was related to grades 3-5 trAEs. There were higher risks associated with Sinti and Camre-Chemo than with the other ICI regimens. Third, as a result of the comparison of ICI regimens related AEs by different SOCs, different regimens were ranked based on the risk of trAEs or irAEs. For trAEs, Sinti was related to a lower risk of blood and lymphatic system disorders; Chemo was related to a reduced risk of endocrine disorders; A lower risk of gastrointestinal disorders was associated with Durva; As a result of Cemip, a lower risk of general disorders and administration site conditions was observed. There was a lower risk of investigations associated with Atezo; Tisle was related to a reduced risk of metabolism and nutrition disorders; It has been demonstrated that Ave reduces the risk of musculoskeletal and connective tissue disorders, skin and subcutaneous tissue disorders; It was found that Pem was related to a lower risk of nervous system disorders; Pem-Chemo is related to reduced risk of respiratory, thoracic, and mediastinal disorders. For irAEs, Durva was related to the lowest risk of endocrine disorders; Nivo was related to the lowest risk of gastrointestinal disorders and hepatobiliary disorders; Pem was related to the lowest risk of injury, poisoning and procedural complications; Chemo was related to the lowest risk of investigations, respiratory, thoracic and mediastinal disorders, and skin and subcutaneous tissue disorders.

Insights were gained from the disproportionality analysis. As a result of FAERS pharmacovigilance data analysis, 9,420 cases of AEs were associated with ICI treatment in 7,339 patients with NSCLC. There were 152 significant signals associated with Nivo, with malignant neoplasm progression, death and hypothyroidism being the most frequent PTs; For Durva, there were 98 significant signals, and radiation pneumonitis and malignant neoplasm progression were the most frequent PTs; A total of 89 significant signals were observed in the Pem, and immune-mediated adverse reactions are the most common PTs.

As for SOCs, Nivo was associated with 23 SOCs, which were associated with different PTs, with the most frequent SOCs being neoplasms benign, malignant and unspecified and general disorders and administration site conditions; Durva was associated with 20 SOCs, and the most common SOCs are neoplasms benign, malignant and unspecified and respiratory, thoracic and mediastinal disorders; Pem was linked to 21 SOCs, and the most frequency SOCs is endocrine disorders.

As previously reported, Cemip had the best safety profile among all ICIs for any-grade trAEs as indicated by previous studies (78), and ICIs and chemotherapy are known to increase the incidence of trAEs of grades 3-5, particularly when antiangiogenesis drugs are administered simultaneously (79), however, irAEs showed contrary results. In this study, ICI regimens were extensively compared and the risk of trAE and irAE was examined. However, this study has several different respects from previous published studies. For instance, previous studies provided limited evidence on the different type of ICIs, such as PD1 or PD-L1 (12, 13), limited evidence on only irAEs results and lack evidence of the novel ICIs, such as Cemip, or number of included RCTs (11, 14). In this study, we included recently published RCTs that evaluated novel ICIs, such as Cemip and Dostar, and the present study ranked the all evaluated treatment options in the RCTs. In previous studies, NSCLC treatment options were ranked differently from other cancers.

Oncologists, emergency department physicians, critical care providers, and other specialists need to be aware of the most serious toxic effects of ICIs used across cancer types. Immune-mediated damage to normal tissue is the most common presentation of AEs in various organ systems. There is a possibility that the differences in the risk of AEs may be related to the different mechanisms of action of each medication and to the combined use of ICIs (80). Further examination and verification of whether ICIs are associated with these AEs is necessary by conducting additional clinical studies. Furthermore, due to the high risk of irAEs, the clinical use of ICIs should be approached with particular care. In some published guidelines on managing irAEs, it is recommended that a rigorous clinical exam should be conducted prior to initiating ICI to assess the baseline (81, 82). Those with high incidences or poor prognoses should be closely monitored for ICI-associated AEs after immunotherapy has been decided upon. Clinical decisions regarding ICI treatment should be based on the findings presented in the present study. It is important that clinicians determine the duration and type of immunosuppressive treatment depending on the severity of the irAE, and consider whether to reintroduce ICIs after discontinuing them. This approach emphasizes the need to diagnose and treat each patient individually (19).

We believe that this is the first study of its kind to examine trAEs and irAEs associated with different ICI regimens for NSCLC, combining real-world data from the FAERS database with pharmacovigilance studies. The safety profile of ICIs in NSCLC has been analyzed in a full landscape of this study.

### 4.1 Limitations

There are some limitations of this study. First, there were a couple of differences in the terms used to describe AEs in the RCTs. Based on our review, we found that the grading system and terminology used to report AEs are consistent and compatible. Furthermore, with the increase in the number of NSCLC patients receiving ICI therapy, awareness of AEs may have increased. Moreover, those AEs have been matched to their corresponding MedDRA codes at the PT level. Second, there were a wide range of CrIs in this study. This is because there were only a few studies, only small sample sizes in some included trials, and different reporting standards. Third, based on the AEs reported in the RCTs, we were limited in our ability to rank a treatment regimen in terms of its probability of having the lowest risk of each AE. Several treatment options and SOCs could not be evaluated and ranked due to the limited number of available trials. Third, FAERS database is a system for reporting spontaneous incidents around the world. There are many factors that contribute to the selection bias of the data, including ethnicity and geographic location, the timing of drug approvals and market penetration, and understanding of specific AEs. Due to these limitations, we could neither calculate the incidence of ICI-related AEs nor obtain a causal association between ICIs and AEs. In addition, we were unable to obtain more detailed information and characteristic about the reports, and the specific time of death of NSCLC patients. We were unable to conduct further competitive risk analyses between ICIs and AEs. Fourth, we have difficulty extracting all the available anticancer regimens from the FAERS database individually and setting them as the comparator of the disproportionality analysis. It is the wide variety of anticancer regimens, including ICIs, targeted therapy regimens, chemotherapeutics, etc. There may be some indication bias as a result of this, which may lead to false positive associations being established. Last, AEs have only been reported for a few ICIs in the FAERS database, and no AEs have been reported for the novel ICIs. It is reasonable to assume that the NMA of this study can provide this evidence in light of these limitations.

## 5 Conclusions

In conclusion, comprehensive investigation and identification of AEs associated with ICIs was conducted using data from clinical trials and the FAERS database through NMA and disproportionality analyses. Given that cemip and tisle are associated with low trAE risks, they may be the preferred ICI regimens for NSCLC. There was a low risk of any or grades 3-5 irAEs with Atezo-Bevac-Chemo, whereas a high risk of grades 3-5 trAEs was associated with it. Those with autoimmune diseases or immunosuppressive medications may find this finding particularly important. There should be close monitoring and caution when using Durva-Treme-Chemo, Sinti, and Camre-Chemo. While the safety profiles of the ICIs differed, Nivo had the most significant signals, and we found several significant safety signals, including malignant neoplasm progression, death, and pneumonia. Based on the findings of this study, clinicians will be able to better understand potential AEs and be able to intervene early if they occur.

## Data availability statement

The original contributions presented in the study are included in the article/**Supplementary Material**. Further inquiries can be directed to the corresponding author.

## Author contributions

XL: Conceptualization, Data curation, Formal analysis, Investigation, Methodology, Project administration, Resources, Supervision, Validation, Visualization, Writing – original draft. HX: Conceptualization, Data curation, Formal analysis, Investigation, Methodology, Project administration, Resources, Supervision, Validation, Visualization, Writing – original draft. HL: Conceptualization, Formal analysis, Investigation, Project administration, Resources, Supervision, Validation, Visualization, Writing – review & editing. XC: Conceptualization, Investigation, Project administration, Resources, Supervision, Validation, Visualization, Writing – review & editing. YL: Conceptualization, Data curation, Formal analysis, Funding acquisition, Methodology, Project administration, Software, Validation, Visualization, Writing – original draft, Writing – review & editing.
