# Supplementary material for: Adverse events associated with immune checkpoint inhibitors in non-small cell lung cancer: a safety analysis of clinical trials and FDA pharmacovigilance system
Source: Front Immunol. 2024 Apr 30;15:1396752. doi: 10.3389/fimmu.2024.1396752 (PMC11091284; doi:10.3389/fimmu.2024.1396752)
Supplement: Supplementary file 1 [file DataSheet_1.pdf]

Supplementary Content

Supplementary Figure S1. Preferred reporting items for systematic reviews and meta-analyses (PRISMA) flow diagram.

Supplementary Figure S2. Network plots of system organ classes specific treatment-related adverse events of ICIs for NSCLC.

Supplementary Figure S3. Network plots of system organ classes specific immune-related adverse events of ICIs for NSCLC.

Supplementary Figure S4. Odds ratio (95% CrI) of system organ classes specific treatment-related adverse events associated with each treatment regimen.

Supplementary Figure S5. Odds ratio (95% CrI) of system organ classes specific immune-related adverse events associated with each treatment regimen.

Supplementary Figure S6. Ranking of the probability of being the best treatment regimen in system organ classes specific treatment-related adverse events.

Supplementary Figure S7. Ranking of the probability of being the best treatment regimen in system organ classes specific immune-related adverse events.

Supplementary Figure S8. Heterogeneity and inconsistency analysis of network meta-analysis results.

Supplementary Figure S9. HLGT and HLT legend of **Fig. 5**.

Supplementary Table S1. Characteristics of included randomized controlled trials for NSCLC.

Supplementary Table S2. Risk of bias assessment of the included studies.

Supplementary Table S3. Characteristics of reports with ICI-related adverse events in NSCLC patients.

Supplementary Table S4. The case number of different adverse events in NSCLC cases receiving ICI treatment in FAERS database.

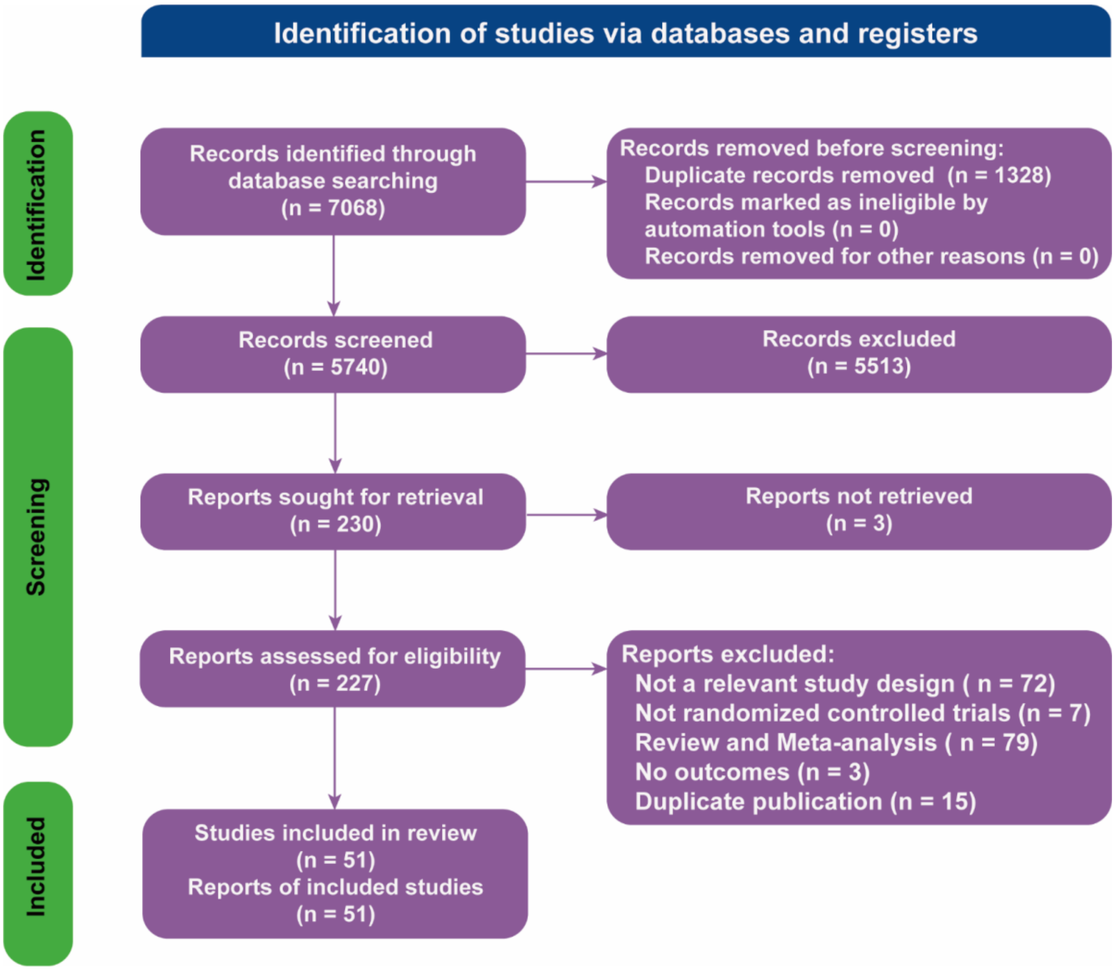

Supplementary Figure S1. Preferred reporting items for systematic reviews and meta-analyses (PRISMA) flow diagram.

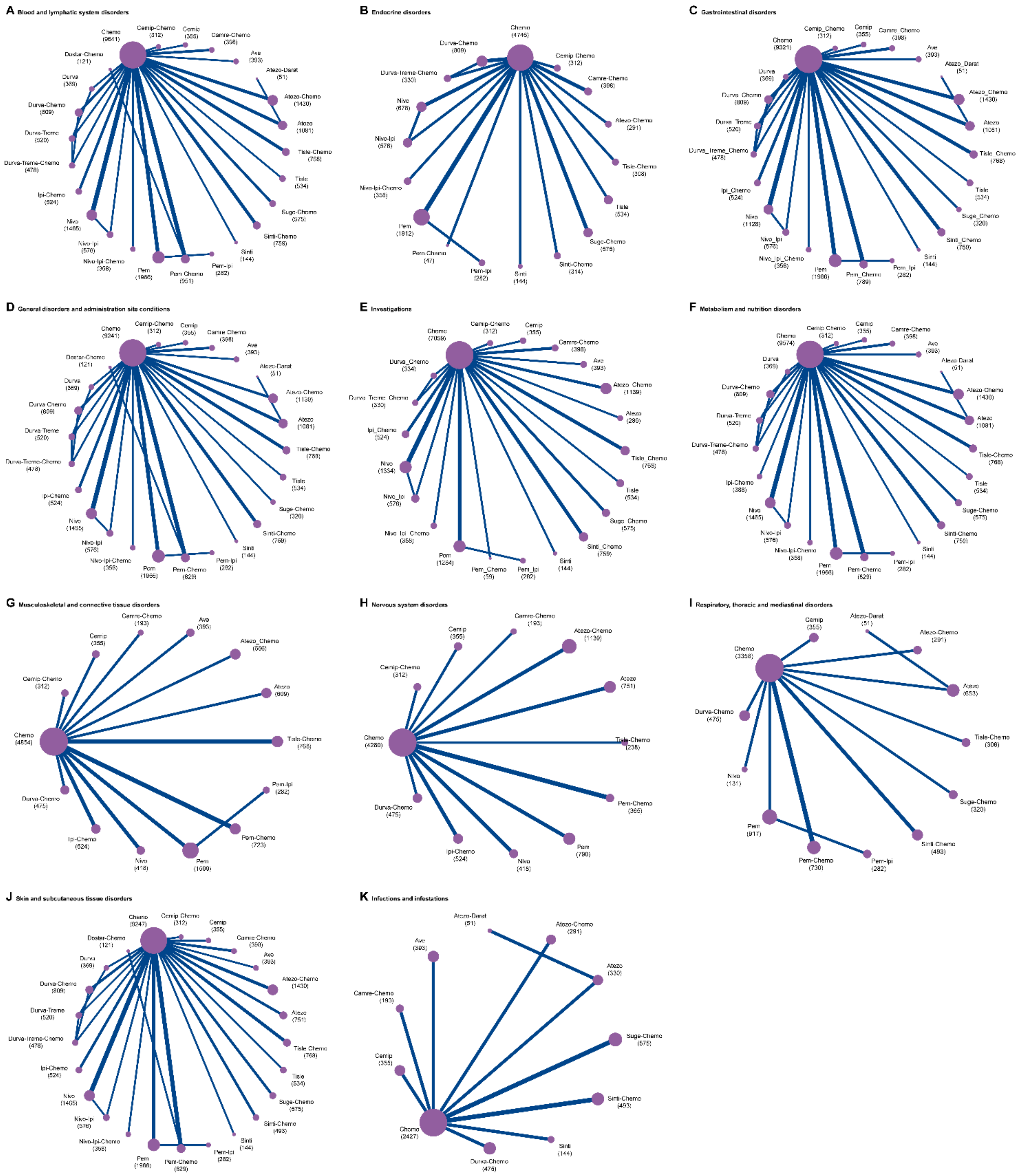

Supplementary Figure S2. Network plots of system organ classes specific treatment-related adverse events of ICIs for NSCLC. (A) Blood and lymphatic system disorders; (B) Endocrine disorders; (C) Gastrointestinal disorders; (D) General disorders and administration site conditions; (E) Investigations; (F) Metabolism and nutrition disorders; (G) Musculoskeletal and connective tissue disorders; (H) Nervous system disorders; (I) Respiratory, thoracic and mediastinal disorders; (J) Skin and subcutaneous tissue disorders; (K) Infections and infestations. Atezo: atezolizumab; Ave: avelumab; Beva: bevacizumab; Camre: camrelizumab; Cemip: cemiplimab; Chemo, chemotherapy; Darat: daratumumab; Dostra, dostarlimab; Durva: durvalumab; Ipi: ipilimumab; Nivo: nivolumab; NSCLC: non-small cell lung cancer; Pem: pembrolizumab; Sint: sintilimab; Sugema: sugemalimab; Tisle: tislelizumab; Treme: tremelimumab.

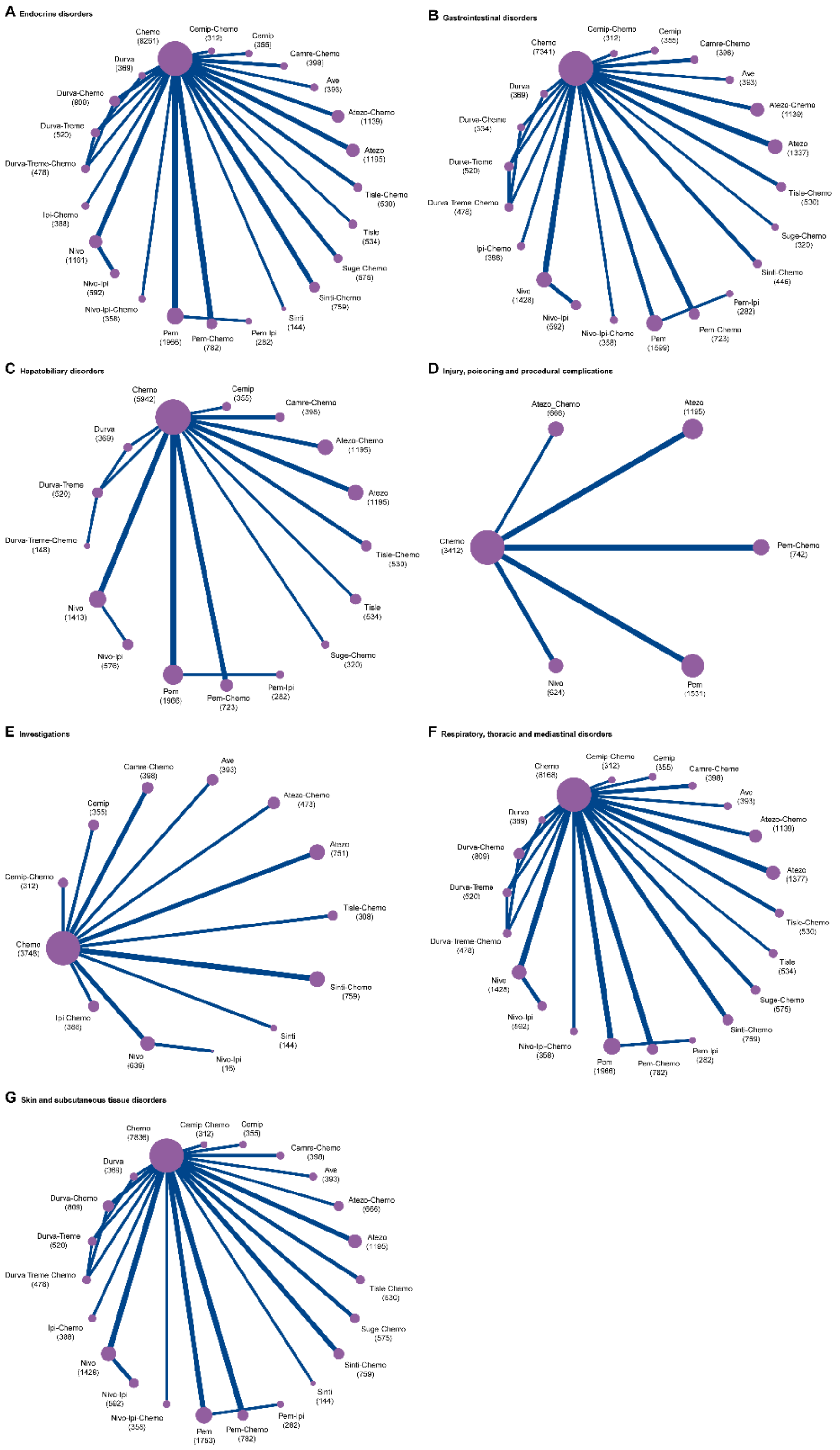

Supplementary Figure S3. Network plots of system organ classes specific immune-related adverse events of ICIs for NSCLC. (A) Endocrine disorders; (B) Gastrointestinal disorders; (C) Hepatobiliary disorders; (D) Injury, poisoning and procedural complications; (E) Investigations; (F) Respiratory, thoracic and mediastinal disorders; (G) Skin and subcutaneous tissue disorders. Atezo: atezolizumab; Ave: avelumab; Beva: bevacizumab; Camre: camrelizumab; Cemip: cemiplimab; Chemo, chemotherapy; Darat: daratumumab; Dostra, dostarlimab; Durva: durvalumab; Ipi: ipilimumab; Nivo: nivolumab; NSCLC: non-small cell lung cancer; Pem: pembrolizumab; Sint: sintilimab; Sugema: sugemalimab; Tisle: tislelizumab; Treme: tremelimumab.

(A) Blood and lymphatic system disorders

Rank 1	Rank 2	Rank 3	Rank 4	Rank 5	Rank 6	Rank 7	Rank 8	Rank 9	Rank 10	Rank 11	Rank 12	Rank 13	Rank 14	Rank 15	Rank 16	Rank 17	Rank 18	Rank 19	Rank 20	Rank 21	Rank 22	Rank 23	Rank 24	Rank 25
Sinti																								
0.32 (0.04, 1.32)	Cemip																							
0.23 (0.03, 1.07)	0.72 (0.31, 1.81)	Atezo-Chemo																						
0.22 (0.03, 0.97)	0.68 (0.30, 1.63)	0.94 (0.34, 2.53)	Durva-Treme																					
0.21 (0.03, 1.00)	0.64 (0.26, 1.75)	0.88 (0.31, 2.68)	0.95 (0.34, 2.78)	Ave																				
0.20 (0.03, 0.86)	0.61 (0.27, 1.42)	0.84 (0.31, 2.20)	0.90 (0.37, 2.16)	0.95 (0.33, 2.59)	Durva																			
0.16 (0.02, 0.61)	0.50 (0.28, 0.89)	0.70 (0.30, 1.44)	0.74 (0.34, 1.48)	0.79 (0.31, 1.70)	0.83 (0.39, 1.61)	Nivo																		
0.09 (0.01, 0.34)	0.26 (0.13, 0.51)	0.36 (0.15, 0.81)	0.39 (0.17, 0.85)	0.41 (0.15, 0.97)	0.43 (0.19, 0.92)	0.52 (0.32, 0.89)	Nivo-Ipi																	
0.07 (0.01, 0.30)	0.23 (0.11, 0.47)	0.32 (0.12, 0.74)	0.34 (0.14, 0.77)	0.36 (0.13, 0.88)	0.38 (0.16, 0.84)	0.45 (0.25, 0.83)	0.87 (0.43, 1.72)	Pem-Ipi																
0.07 (0.01, 0.27)	0.23 (0.13, 0.39)	0.32 (0.14, 0.63)	0.34 (0.16, 0.66)	0.36 (0.14, 0.76)	0.38 (0.18, 0.71)	0.45 (0.32, 0.65)	0.87 (0.52, 1.41)	1.00 (0.62, 1.60)	Pem															
0.04 (0.004, 0.22)	0.12 (0.03, 0.42)	0.16 (0.04, 0.64)	0.17 (0.04, 0.66)	0.18 (0.04, 0.74)	0.19 (0.05, 0.73)	0.23 (0.07, 0.79)	0.44 (0.12, 1.58)	0.51 (0.13, 1.88)	0.51 (0.15, 1.72)	Atezo-Darat														
0.05 (0.01, 0.17)	0.14 (0.08, 0.24)	0.19 (0.09, 0.39)	0.21 (0.10, 0.41)	0.22 (0.09, 0.47)	0.23 (0.11, 0.44)	0.28 (0.19, 0.40)	0.53 (0.32, 0.87)	0.61 (0.34, 1.10)	0.61 (0.44, 0.86)	1.20 (0.37, 4.00)	Atezo													
0.01 (0.001, 0.04)	0.03 (0.02, 0.06)	0.05 (0.02, 0.10)	0.05 (0.02, 0.10)	0.05 (0.02, 0.11)	0.05 (0.03, 0.11)	0.07 (0.04, 0.10)	0.13 (0.07, 0.22)	0.15 (0.08, 0.27)	0.15 (0.10, 0.21)	0.29 (0.08, 1.01)	0.24 (0.16, 0.35)	Nivo-Ipi-Chemo												
0.01 (0.001, 0.03)	0.03 (0.01, 0.05)	0.04 (0.02, 0.08)	0.04 (0.02, 0.08)	0.04 (0.02, 0.09)	0.04 (0.02, 0.09)	0.05 (0.03, 0.08)	0.10 (0.06, 0.17)	0.11 (0.06, 0.21)	0.11 (0.07, 0.17)	0.22 (0.06, 0.80)	0.18 (0.12, 0.28)	0.77 (0.49, 1.23)	Durva-Chemo											
0.004 (0.0005, 0.01)	0.01 (0.01, 0.02)	0.02 (0.01, 0.03)	0.02 (0.01, 0.03)	0.02 (0.01, 0.04)	0.02 (0.01, 0.03)	0.02 (0.02, 0.03)	0.04 (0.03, 0.07)	0.05 (0.03, 0.08)	0.05 (0.04, 0.06)	0.10 (0.03, 0.33)	0.08 (0.06, 0.10)	0.34 (0.25, 0.46)	0.44 (0.31, 0.62)	Chemo										
0.003 (0.0005, 0.01)	0.01 (0.01, 0.02)	0.01 (0.01, 0.03)	0.02 (0.01, 0.03)	0.02 (0.01, 0.04)	0.02 (0.01, 0.04)	0.02 (0.01, 0.04)	0.04 (0.02, 0.08)	0.05 (0.02, 0.09)	0.05 (0.03, 0.08)	0.09 (0.03, 0.34)	0.08 (0.05, 0.13)	0.32 (0.19, 0.55)	0.42 (0.24, 0.72)	0.95 (0.62, 1.46)	Pem-Chemo									
0.003 (0.0005, 0.01)	0.01 (0.01, 0.02)	0.01 (0.01, 0.03)	0.02 (0.01, 0.03)	0.02 (0.01, 0.04)	0.02 (0.01, 0.03)	0.02 (0.01, 0.03)	0.04 (0.02, 0.07)	0.04 (0.02, 0.08)	0.05 (0.03, 0.07)	0.09 (0.03, 0.31)	0.07 (0.05, 0.11)	0.31 (0.20, 0.48)	0.40 (0.25, 0.63)	0.91 (0.66, 1.24)	0.96 (0.56, 1.62)	Ipi-Chemo								
0.003 (0.0005, 0.01)	0.01 (0.01, 0.02)	0.01 (0.01, 0.03)	0.02 (0.01, 0.03)	0.02 (0.01, 0.03)	0.02 (0.01, 0.03)	0.02 (0.01, 0.03)	0.04 (0.02, 0.07)	0.04 (0.02, 0.08)	0.04 (0.03, 0.06)	0.09 (0.03, 0.31)	0.07 (0.05, 0.11)	0.31 (0.20, 0.47)	0.40 (0.25, 0.62)	0.90 (0.68, 1.20)	0.95 (0.57, 1.59)	1.00 (0.65, 1.52)	Sinti-Chemo							
0.003 (0.0004, 0.01)	0.01 (0.005, 0.02)	0.01 (0.01, 0.03)	0.01 (0.01, 0.03)	0.02 (0.01, 0.04)	0.02 (0.01, 0.03)	0.02 (0.01, 0.03)	0.04 (0.02, 0.07)	0.04 (0.02, 0.08)	0.04 (0.03, 0.07)	0.08 (0.02, 0.31)	0.07 (0.04, 0.11)	0.29 (0.17, 0.49)	0.37 (0.21, 0.65)	0.85 (0.54, 1.31)	0.90 (0.48, 1.65)	0.94 (0.54, 1.60)	0.94 (0.55, 1.59)	Tisle						
0.003 (0.0004, 0.01)	0.01 (0.005, 0.02)	0.01 (0.01, 0.02)	0.01 (0.01, 0.03)	0.01 (0.01, 0.03)	0.01 (0.01, 0.03)	0.02 (0.01, 0.03)	0.03 (0.02, 0.05)	0.04 (0.02, 0.07)	0.04 (0.03, 0.05)	0.07 (0.02, 0.26)	0.06 (0.04, 0.09)	0.26 (0.17, 0.39)	0.33 (0.21, 0.52)	0.75 (0.56, 1.01)	0.79 (0.47, 1.32)	0.83 (0.54, 1.27)	0.83 (0.55, 1.25)	0.88 (0.52, 1.51)	Camre-Chemo					
0.002 (0.0001, 0.02)	0.01 (0.0009, 0.03)	0.01 (0.001, 0.05)	0.01 (0.001, 0.05)	0.01 (0.001, 0.06)	0.01 (0.001, 0.06)	0.01 (0.002, 0.07)	0.02 (0.003, 0.13)	0.02 (0.004, 0.15)	0.02 (0.004, 0.15)	0.05 (0.01, 0.41)	0.04 (0.01, 0.24)	0.17 (0.03, 1.01)	0.22 (0.04, 1.30)	0.49 (0.08, 2.86)	0.52 (0.08, 3.18)	0.55 (0.09, 3.25)	0.55 (0.09, 3.26)	0.58 (0.09, 3.58)	0.66 (0.11, 3.90)	Tisle-Chemo				
0.002 (0.0003, 0.009)	0.01 (0.004, 0.01)	0.01 (0.004, 0.02)	0.01 (0.004, 0.02)	0.01 (0.004, 0.03)	0.01 (0.01, 0.02)	0.01 (0.01, 0.02)	0.03 (0.01, 0.05)	0.03 (0.02, 0.06)	0.03 (0.02, 0.05)	0.06 (0.02, 0.22)	0.05 (0.03, 0.08)	0.21 (0.12, 0.35)	0.27 (0.16, 0.47)	0.62 (0.41, 0.94)	0.65 (0.36, 1.19)	0.68 (0.40, 1.15)	0.68 (0.41, 1.14)	0.73 (0.40, 1.34)	0.82 (0.49, 1.38)	1.25 (0.20, 7.67)	Cemip-Chemo			
0.002 (0.0002, 0.01)	0.01 (0.002, 0.02)	0.01 (0.002, 0.03)	0.01 (0.003, 0.03)	0.01 (0.002, 0.03)	0.01 (0.003, 0.03)	0.01 (0.004, 0.03)	0.02 (0.01, 0.07)	0.03 (0.01, 0.08)	0.03 (0.01, 0.07)	0.05 (0.01, 0.24)	0.04 (0.02, 0.12)	0.18 (0.06, 0.49)	0.23 (0.08, 0.64)	0.52 (0.19, 1.38)	0.55 (0.22, 1.31)	0.58 (0.20, 1.60)	0.58 (0.20, 1.59)	0.62 (0.21, 1.79)	0.70 (0.25, 1.92)	1.06 (0.14, 7.95)	0.85 (0.28, 2.44)	Dostar-Chemo		
0.001 (0.0001, 0.006)	0.004 (0.001, 0.01)	0.01 (0.001, 0.02)	0.01 (0.002, 0.02)	0.01 (0.002, 0.02)	0.01 (0.002, 0.02)	0.01 (0.003, 0.02)	0.02 (0.005, 0.04)	0.02 (0.01, 0.05)	0.02 (0.01, 0.05)	0.03 (0.01, 0.16)	0.03 (0.01, 0.07)	0.12 (0.04, 0.32)	0.15 (0.05, 0.41)	0.35 (0.12, 0.88)	0.37 (0.12, 1.03)	0.39 (0.13, 1.03)	0.39 (0.13, 1.03)	0.41 (0.13, 1.16)	0.47 (0.15, 1.23)	0.70 (0.09, 5.20)	0.57 (0.18, 1.57)	(0.16, 2.63)	Suge-Chemo	
0.0002 (1E-05, 0.001)	0.001 (7E-05, 0.002)	0.001 (9E-05, 0.003)	0.001 (0.0001, 0.003)	0.001 (0.0001, 0.004)	0.001 (0.0001, 0.004)	0.001 (0.0002, 0.004)	0.002 (0.0003, 0.008)	0.002 (0.0003, 0.009)	0.002 (0.0003, 0.009)	0.005 (0.0005, 0.03)	0.004 (0.0006, 0.01)	0.02 (0.002, 0.06)	0.02 (0.003, 0.07)	0.05 (0.01, 0.17)	0.05 (0.01, 0.19)	0.05 (0.01, 0.20)	0.05 (0.01, 0.19)	0.06 (0.01, 0.22)	0.06 (0.01, 0.23)	0.09 (0.01, 0.86)	0.08 (0.01, 0.30)	0.09 (0.01, 0.47)	0.14 (0.02, 0.72)	Durva-Treme-Chemo

Treatment-related blood and lymphatic system disorders

(B) Endocrine disorders

Rank 1	Rank 2	Rank 3	Rank 4	Rank 5	Rank 6	Rank 7	Rank 8	Rank 9	Rank 10	Rank 11	Rank 12	Rank 13	Rank 14	Rank 15	Rank 16	Rank 17
Chemo																
**0.24 (0.13, 0.40)**	Suge-Chemo															
**0.24 (0.11, 0.50)**	1.00 (0.38, 2.53)	Atezo-Chemo														
**0.21 (0.05, 0.62)**	0.86 (0.18, 3.00)	0.85 (0.16, 3.42)	Cemip-Chemo													
**0.19 (0.09, 0.38)**	0.79 (0.31, 1.94)	0.79 (0.27, 2.29)	0.92 (0.23, 4.74)	Sinti-Chemo												
**0.15 (0.07, 0.30)**	0.63 (0.25, 1.56)	0.63 (0.22, 1.83)	0.74 (0.19, 3.80)	0.80 (0.28, 2.29)	Camre-Chemo											
**0.14 (0.06, 0.30)**	0.60 (0.21, 1.51)	0.59 (0.19, 1.77)	0.69 (0.17, 3.64)	0.75 (0.24, 2.21)	0.94 (0.30, 2.76)	Durva-Chemo										
**0.14 (0.06, 0.28)**	0.58 (0.21, 1.44)	0.57 (0.19, 1.69)	0.67 (0.17, 3.48)	0.72 (0.25, 2.11)	0.91 (0.31, 2.63)	0.97 (0.32, 3.00)	Tisle-Chemo									
**0.10 (0.003, 0.73)**	0.41 (0.01, 3.36)	0.41 (0.01, 3.62)	0.48 (0.01, 5.91)	0.52 (0.02, 4.52)	0.65 (0.02, 5.65)	0.70 (0.02, 6.27)	0.72 (0.02, 6.31)	Pem-Chemo								
**0.06 (0.02, 0.14)**	**0.26 (0.08, 0.71)**	**0.25 (0.08, 0.82)**	0.30 (0.07, 1.66)	0.32 (0.10, 1.03)	0.40 (0.12, 1.29)	**0.43 (0.24, 0.77)**	0.45 (0.13, 1.46)	0.62 (0.07, 18.86)	Durva-Treme-							
**0.03 (0.01, 0.07)**	**0.14 (0.05, 0.35)**	**0.13 (0.04, 0.41)**	**0.16 (0.04, 0.83)**	**0.17 (0.05, 0.51)**	**0.21 (0.07, 0.64)**	**0.23 (0.07, 0.73)**	**0.23 (0.07, 0.73)**	0.33 (0.04, 9.68)	0.53 (0.15, 1.81)	Pem						
**0.03 (0.004, 0.10)**	**0.12 (0.02, 0.49)**	**0.12 (0.02, 0.55)**	0.14 (0.02, 1.00)	**0.15 (0.02, 0.69)**	**0.19 (0.03, 0.86)**	**0.21 (0.03, 0.96)**	**0.21 (0.03, 0.97)**	0.29 (0.02, 9.89)	0.48 (0.06, 2.36)	0.91 (0.12, 4.40)	Nivo					
**0.02 (0.0008, 0.12)**	**0.09 (0.003, 0.55)**	**0.09 (0.003, 0.61)**	0.10 (0.003, 1.03)	**0.11 (0.004, 0.76)**	**0.14 (0.005, 0.95)**	0.15 (0.01, 1.05)	0.16 (0.01, 1.07)	0.21 (0.005, 8.95)	0.35 (0.01, 2.57)	0.67 (0.02, 4.77)	0.74 (0.02, 9.71)	Tisle				
**0.02 (0.01, 0.06)**	**0.10 (0.03, 0.29)**	**0.10 (0.03, 0.34)**	**0.12 (0.02, 0.66)**	**0.13 (0.04, 0.42)**	**0.16 (0.04, 0.52)**	**0.17 (0.05, 0.59)**	**0.17 (0.05, 0.60)**	0.24 (0.02, 7.43)	0.39 (0.10, 1.46)	0.74 (0.45, 1.20)	0.82 (0.16, 6.60)	1.11 (0.14, 33.59)	Pem-Ipi			
**0.02 (0.0006, 0.06)**	**0.06 (0.002, 0.40)**	**0.06 (0.002, 0.43)**	**0.07 (0.002, 0.73)**	**0.08 (0.003, 0.54)**	**0.10 (0.003, 0.68)**	**0.11 (0.004, 0.76)**	**0.11 (0.004, 0.77)**	0.15 (0.003, 6.32)	0.25 (0.01, 1.85)	0.47 (0.02, 3.45)	0.52 (0.02, 6.95)	0.70 (0.02, 27.95)	0.63 (0.02, 5.02)	Sinti		
**0.01 (0.002, 0.05)**	**0.06 (0.01, 0.25)**	**0.06 (0.01, 0.28)**	**0.07 (0.01, 0.50)**	**0.08 (0.01, 0.35)**	**0.10 (0.01, 0.44)**	**0.10 (0.01, 0.49)**	**0.11 (0.01, 0.49)**	0.15 (0.01, 4.99)	0.24 (0.03, 1.20)	0.46 (0.06, 2.22)	**0.51 (0.31, 0.81)**	0.69 (0.05, 22.76)	0.62 (0.08, 3.25)	0.98 (0.07, 32.2)	Nivo-Ipi	
**0.008 (0.0004, 0.05)**	**0.03 (0.001, 0.21)**	**0.03 (0.001, 0.23)**	**0.04 (0.001, 0.40)**	**0.04 (0.002, 0.29)**	**0.05 (0.002, 0.36)**	**0.06 (0.002, 0.40)**	**0.06 (0.002, 0.41)**	0.08 (0.002, 3.44)	**0.13 (0.01, 0.99)**	0.25 (0.01, 1.82)	0.28 (0.01, 3.65)	0.38 (0.01, 14.34)	0.34 (0.01, 2.66)	0.54 (0.01, 22.02)	0.55 (0.02, 7.28)	Nivo-Ipi-Chemo

Treatment-related endocrine disorders

(C) Gastrointestinal disorders

Rank 1	Rank 2	Rank 3	Rank 4	Rank 5	Rank 6	Rank 7	Rank 8	Rank 9	Rank 10	Rank 11	Rank 12	Rank 13	Rank 14	Rank 15	Rank 16	Rank 17	Rank 18	Rank 19	Rank 20	Rank 21	Rank 22	Rank 23	Rank 24
**Durva**																							
0.71 (0.47, 1.07)	**Nivo**																						
**0.61 (0.42, 0.88)**	0.86 (0.59, 1.25)	**Durva-Treme**																					
**0.58 (0.39, 0.87)**	0.82 (0.63, 1.08)	0.96 (0.66, 1.38)	**Pem**																				
**0.48 (0.29, 0.79)**	0.68 (0.45, 1.01)	0.79 (0.49, 1.26)	0.82 (0.56, 1.21)	**Cemip**																			
**0.37 (0.24, 0.57)**	**0.52 (0.40, 0.67)**	**0.61 (0.41, 0.90)**	**0.63 (0.47, 0.85)**	0.77 (0.50, 1.17)	**Nivo-Ipi**																		
**0.37 (0.21, 0.62)**	**0.52 (0.33, 0.80)**	0.60 (0.36, 1.00)	**0.63 (0.45, 0.88)**	0.77 (0.45, 1.29)	1.00 (0.63, 1.57)	**Pem-Ipi**																	
**0.36 (0.21, 0.61)**	**0.51 (0.34, 0.78)**	**0.60 (0.37, 0.97)**	**0.62 (0.41, 0.94)**	0.76 (0.46, 1.25)	0.98 (0.64, 1.53)	0.99 (0.58, 1.70)	**Ave**																
**0.28 (0.17, 0.47)**	**0.39 (0.26, 0.60)**	**0.46 (0.28, 0.75)**	**0.48 (0.32, 0.72)**	**0.59 (0.35, 0.97)**	0.76 (0.49, 1.18)	0.76 (0.45, 1.31)	0.77 (0.46, 1.30)	**Tisle**															
**0.19 (0.05, 0.92)**	0.27 (0.08, 1.26)	0.31 (0.09, 1.50)	0.32 (0.10, 1.53)	0.39 (0.11, 1.91)	0.51 (0.15, 2.44)	0.51 (0.15, 2.52)	0.52 (0.15, 2.55)	0.67 (0.19, 3.29)	**Sinti-Chemo**														
**0.22 (0.15, 0.33)**	**0.31 (0.24, 0.42)**	**0.37 (0.25, 0.53)**	**0.38 (0.29, 0.50)**	**0.46 (0.31, 0.69)**	**0.60 (0.44, 0.82)**	**0.61 (0.39, 0.94)**	**0.61 (0.40, 0.93)**	0.79 (0.52, 1.20)	1.18 (0.25, 3.94)	**Atezo**													
**0.13 (0.06, 0.26)**	**0.18 (0.09, 0.35)**	**0.21 (0.10, 0.42)**	**0.22 (0.11, 0.42)**	**0.27 (0.13, 0.55)**	**0.34 (0.18, 0.68)**	**0.35 (0.17, 0.73)**	**0.35 (0.17, 0.73)**	**0.45 (0.22, 0.95)**	0.67 (0.13, 2.61)	0.57 (0.30, 1.12)	**Sinti**												
**0.10 (0.04, 0.25)**	**0.14 (0.06, 0.33)**	**0.16 (0.06, 0.40)**	**0.17 (0.07, 0.40)**	**0.21 (0.08, 0.51)**	**0.27 (0.11, 0.65)**	**0.27 (0.10, 0.68)**	**0.27 (0.11, 0.69)**	**0.35 (0.14, 0.89)**	0.52 (0.09, 2.28)	0.44 (0.19, 1.01)	0.78 (0.27, 2.23)	**Atezo-Darat**											
**0.08 (0.05, 0.12)**	**0.11 (0.08, 0.15)**	**0.13 (0.09, 0.19)**	**0.13 (0.10, 0.18)**	**0.16 (0.11, 0.24)**	**0.21 (0.15, 0.29)**	**0.21 (0.14, 0.33)**	**0.21 (0.14, 0.33)**	**0.28 (0.18, 0.43)**	0.41 (0.09, 1.39)	**0.35 (0.26, 0.47)**	0.61 (0.31, 1.19)	0.79 (0.33, 1.93)	**Durva-Chemo**										
**0.08 (0.05, 0.12)**	**0.11 (0.07, 0.16)**	**0.12 (0.08, 0.20)**	**0.13 (0.09, 0.19)**	**0.16 (0.10, 0.26)**	**0.21 (0.14, 0.31)**	**0.21 (0.12, 0.35)**	**0.21 (0.13, 0.34)**	**0.27 (0.16, 0.45)**	0.40 (0.08, 1.39)	**0.34 (0.23, 0.51)**	0.60 (0.29, 1.21)	0.77 (0.31, 1.95)	0.98 (0.65, 1.46)	**Nivo-Ipi-Chemo**									
**0.07 (0.05, 0.10)**	**0.10 (0.08, 0.12)**	**0.12 (0.08, 0.16)**	**0.12 (0.10, 0.15)**	**0.15 (0.10, 0.21)**	**0.19 (0.15, 0.24)**	**0.19 (0.13, 0.29)**	**0.20 (0.13, 0.28)**	**0.25 (0.17, 0.37)**	0.38 (0.08, 1.24)	**0.32 (0.26, 0.39)**	0.56 (0.29, 1.04)	0.72 (0.31, 1.71)	0.91 (0.73, 1.14)	0.93 (0.67, 1.31)	**Chemo**								
**0.07 (0.04, 0.12)**	**0.10 (0.06, 0.15)**	**0.11 (0.07, 0.18)**	**0.12 (0.08, 0.18)**	**0.14 (0.08, 0.24)**	**0.18 (0.12, 0.29)**	**0.18 (0.11, 0.32)**	**0.19 (0.11, 0.32)**	**0.24 (0.14, 0.41)**	0.36 (0.07, 1.26)	**0.30 (0.20, 0.47)**	0.53 (0.25, 1.11)	0.68 (0.27, 1.77)	0.87 (0.55, 1.37)	0.89 (0.53, 1.49)	0.95 (0.64, 1.41)	**Suge-Chemo**							
**0.05 (0.03, 0.08)**	**0.07 (0.05, 0.10)**	**0.08 (0.05, 0.12)**	**0.08 (0.06, 0.12)**	**0.10 (0.06, 0.16)**	**0.13 (0.09, 0.19)**	**0.13 (0.08, 0.21)**	**0.13 (0.08, 0.21)**	**0.17 (0.11, 0.28)**	**0.26 (0.05, 0.87)**	**0.22 (0.15, 0.31)**	**0.38 (0.19, 0.76)**	0.49 (0.2, 1.22)	**0.62 (0.43, 0.90)**	**0.63 (0.41, 0.99)**	**0.68 (0.50, 0.91)**	0.71 (0.43, 1.16)	**Camre-Chemo**						
**0.05 (0.03, 0.08)**	**0.07 (0.05, 0.10)**	**0.08 (0.05, 0.12)**	**0.08 (0.06, 0.12)**	**0.10 (0.06, 0.16)**	**0.13 (0.09, 0.19)**	**0.13 (0.08, 0.21)**	**0.13 (0.08, 0.21)**	**0.17 (0.11, 0.27)**	**0.25 (0.05, 0.87)**	**0.22 (0.15, 0.31)**	**0.38 (0.19, 0.76)**	0.48 (0.20, 1.22)	**0.62 (0.45, 0.84)**	**0.63 (0.40, 0.99)**	**0.68 (0.50, 0.91)**	0.71 (0.43, 1.16)	1.00 (0.65, 1.52)	**Durva-Treme-**					
**0.05 (0.03, 0.07)**	**0.07 (0.05, 0.09)**	**0.08 (0.05, 0.12)**	**0.08 (0.06, 0.11)**	**0.10 (0.06, 0.15)**	**0.13 (0.09, 0.18)**	**0.13 (0.08, 0.21)**	**0.13 (0.08, 0.21)**	**0.17 (0.11, 0.27)**	**0.25 (0.05, 0.85)**	**0.21 (0.15, 0.30)**	**0.37 (0.19, 0.73)**	0.48 (0.20, 1.19)	**0.61 (0.43, 0.86)**	**0.62 (0.41, 0.96)**	**0.67 (0.51, 0.87)**	0.70 (0.43, 1.13)	0.98 (0.66, 1.47)	0.99 (0.66, 1.48)	**Atezo-Chemo**				
**0.05 (0.03, 0.08)**	**0.06 (0.04, 0.10)**	**0.07 (0.05, 0.12)**	**0.08 (0.05, 0.12)**	**0.09 (0.06, 0.16)**	**0.12 (0.08, 0.19)**	**0.12 (0.07, 0.21)**	**0.12 (0.07, 0.21)**	**0.16 (0.10, 0.27)**	**0.24 (0.05, 0.83)**	**0.20 (0.13, 0.31)**	**0.36 (0.17, 0.73)**	0.46 (0.18, 1.18)	**0.58 (0.38, 0.89)**	**0.60 (0.37, 0.98)**	**0.64 (0.45, 0.92)**	0.67 (0.39, 1.15)	0.94 (0.59, 1.50)	0.95 (0.59, 1.52)	0.96 (0.61, 1.51)	**Tisle-Chemo**			
**0.04 (0.02, 0.06)**	**0.05 (0.03, 0.08)**	**0.06 (0.04, 0.10)**	**0.06 (0.04, 0.10)**	**0.08 (0.04, 0.13)**	**0.10 (0.06, 0.16)**	**0.10 (0.06, 0.17)**	**0.10 (0.06, 0.17)**	**0.13 (0.07, 0.22)**	**0.19 (0.04, 0.68)**	**0.16 (0.10, 0.25)**	**0.28 (0.13, 0.60)**	**0.37 (0.14, 0.95)**	**0.47 (0.29, 0.73)**	**0.48 (0.28, 0.80)**	**0.51 (0.34, 0.76)**	**0.54 (0.31, 0.94)**	0.75 (0.46, 1.23)	0.76 (0.46, 1.25)	0.77 (0.47, 1.24)	0.80 (0.46, 1.36)	**Cemip-Chemo**		
**0.04 (0.02, 0.06)**	**0.05 (0.04, 0.07)**	**0.06 (0.04, 0.09)**	**0.06 (0.04, 0.08)**	**0.07 (0.05, 0.11)**	**0.10 (0.07, 0.14)**	**0.10 (0.06, 0.15)**	**0.10 (0.06, 0.15)**	**0.13 (0.08, 0.20)**	**0.19 (0.04, 0.63)**	**0.16 (0.11, 0.22)**	**0.28 (0.14, 0.55)**	**0.36 (0.15, 0.88)**	**0.45 (0.32, 0.64)**	**0.46 (0.30, 0.71)**	**0.50 (0.38, 0.65)**	**0.52 (0.32, 0.84)**	0.73 (0.49, 1.09)	0.73 (0.49, 1.10)	0.74 (0.51, 1.09)	0.78 (0.49, 1.22)	0.97 (0.6, 1.57)	**Ipi-Chemo**	
**0.02 (0.01, 0.04)**	**0.03 (0.02, 0.06)**	**0.04 (0.02, 0.07)**	**0.04 (0.02, 0.07)**	**0.04 (0.02, 0.09)**	**0.06 (0.03, 0.11)**	**0.06 (0.03, 0.12)**	**0.06 (0.03, 0.12)**	**0.08 (0.04, 0.15)**	**0.11 (0.02, 0.43)**	**0.10 (0.05, 0.18)**	**0.17 (0.07, 0.39)**	**0.22 (0.08, 0.61)**	**0.28 (0.15, 0.51)**	**0.28 (0.14, 0.55)**	**0.30 (0.17, 0.54)**	**0.32 (0.16, 0.64)**	**0.45 (0.23, 0.85)**	**0.45 (0.23, 0.86)**	**0.45 (0.23, 0.86)**	**0.47 (0.24, 0.93)**	0.59 (0.29, 1.19)	0.61 (0.32, 1.15)	**Pem-Chemo**

Treatment-related gastrointestinal disorders

(D) General disorders and administration site conditions

Rank 1	Rank 2	Rank 3	Rank 4	Rank 5	Rank 6	Rank 7	Rank 8	Rank 9	Rank 10	Rank 11	Rank 12	Rank 13	Rank 14	Rank 15	Rank 16	Rank 17	Rank 18	Rank 19	Rank 20	Rank 21	Rank 22	Rank 23	Rank 24	Rank 25
Cemip																								
0.89 (0.52, 1.48)	Nivo																							
0.86 (0.46, 1.55)	0.96 (0.66, 1.42)	Ave																						
0.85 (0.45, 1.58)	0.96 (0.63, 1.45)	0.99 (0.60, 1.66)	Durva																					
0.78 (0.39, 1.51)	0.87 (0.54, 1.42)	0.91 (0.51, 1.60)	0.91 (0.50, 1.64)	Tisle																				
0.64 (0.35, 1.13)	0.72 (0.50, 1.02)	0.74 (0.47, 1.18)	0.75 (0.50, 1.11)	0.82 (0.47, 1.42)	Durva-Treme																			
0.61 (0.36, 1.02)	**0.69 (0.54, 0.88)**	0.72 (0.48, 1.05)	0.72 (0.47, 1.09)	0.79 (0.48, 1.29)	0.96 (0.67, 1.37)	Pem																		
**0.55 (0.32, 0.92)**	**0.62 (0.47, 0.80)**	**0.64 (0.43, 0.95)**	**0.64 (0.42, 0.98)**	0.71 (0.43, 1.16)	0.86 (0.60, 1.24)	0.90 (0.68, 1.17)	Atezo																	
**0.50 (0.26, 0.92)**	**0.56 (0.37, 0.85)**	**0.58 (0.35, 0.97)**	**0.58 (0.34, 0.99)**	0.64 (0.35, 1.16)	0.78 (0.48, 1.27)	0.81 (0.58, 1.13)	0.91 (0.59, 1.39)	Pem-Ipi																
**0.50 (0.28, 0.86)**	**0.56 (0.43, 0.74)**	**0.58 (0.38, 0.89)**	**0.59 (0.37, 0.91)**	0.64 (0.38, 1.08)	0.78 (0.53, 1.16)	0.81 (0.60, 1.11)	0.91 (0.66, 1.25)	1.00 (0.64, 1.58)	Nivo-Ipi															
0.40 (0.14, 1.08)	0.45 (0.18, 1.09)	0.46 (0.18, 1.19)	0.47 (0.18, 1.21)	0.51 (0.19, 1.37)	0.62 (0.24, 1.57)	0.65 (0.26, 1.58)	0.72 (0.30, 1.69)	0.80 (0.30, 2.08)	0.80 (0.32, 1.98)	Atezo-Darat														
**0.38 (0.19, 0.78)**	**0.43 (0.25, 0.75)**	**0.45 (0.24, 0.84)**	**0.45 (0.24, 0.86)**	**0.50 (0.25, 0.99)**	0.60 (0.33, 1.11)	0.63 (0.36, 1.09)	0.70 (0.40, 1.23)	0.77 (0.41, 1.48)	0.77 (0.43, 1.38)	0.97 (0.35, 2.72)	Sinti													
**0.34 (0.13, 0.93)**	**0.38 (0.16, 0.94)**	0.40 (0.16, 1.03)	0.40 (0.16, 1.04)	0.44 (0.17, 1.19)	0.53 (0.22, 1.36)	0.56 (0.23, 1.37)	0.62 (0.26, 1.54)	0.69 (0.27, 1.79)	0.68 (0.28, 1.71)	0.86 (0.25, 2.99)	0.88 (0.33, 2.47)	Dostar-Chemo												
**0.29 (0.17, 0.47)**	**0.32 (0.27, 0.38)**	**0.34 (0.24, 0.47)**	**0.34 (0.23, 0.49)**	**0.37 (0.24, 0.59)**	**0.45 (0.33, 0.61)**	**0.47 (0.39, 0.56)**	**0.53 (0.43, 0.64)**	**0.58 (0.40, 0.85)**	**0.58 (0.45, 0.74)**	0.73 (0.30, 1.77)	0.75 (0.44, 1.27)	0.85 (0.35, 1.98)	Chemo											
**0.26 (0.15, 0.44)**	**0.29 (0.22, 0.38)**	**0.30 (0.20, 0.45)**	**0.30 (0.20, 0.46)**	**0.33 (0.20, 0.55)**	**0.41 (0.28, 0.58)**	**0.42 (0.32, 0.55)**	**0.47 (0.35, 0.63)**	**0.52 (0.34, 0.80)**	**0.52 (0.38, 0.71)**	0.65 (0.27, 1.62)	0.67 (0.38, 1.18)	0.76 (0.31, 1.81)	0.89 (0.73, 1.10)	Atezo-Chemo										
**0.24 (0.14, 0.42)**	**0.27 (0.21, 0.36)**	**0.28 (0.19, 0.43)**	**0.29 (0.19, 0.44)**	**0.31 (0.19, 0.52)**	**0.38 (0.27, 0.55)**	**0.40 (0.30, 0.53)**	**0.44 (0.33, 0.60)**	**0.49 (0.32, 0.76)**	**0.49 (0.35, 0.69)**	0.61 (0.25, 1.54)	0.63 (0.36, 1.12)	0.72 (0.29, 1.73)	0.84 (0.67, 1.06)	0.94 (0.69, 1.28)	Durva-Chemo									
**0.24 (0.14, 0.42)**	**0.27 (0.20, 0.37)**	**0.28 (0.18, 0.44)**	**0.28 (0.18, 0.45)**	**0.31 (0.19, 0.53)**	**0.38 (0.26, 0.57)**	**0.40 (0.29, 0.54)**	**0.44 (0.32, 0.61)**	**0.49 (0.31, 0.77)**	**0.49 (0.34, 0.70)**	0.61 (0.24, 1.55)	0.63 (0.35, 1.13)	0.71 (0.28, 1.73)	0.84 (0.65, 1.09)	0.94 (0.68, 1.31)	0.99 (0.70, 1.41)	Tisle-Chemo								
**0.23 (0.13, 0.40)**	**0.25 (0.18, 0.35)**	**0.26 (0.17, 0.41)**	**0.27 (0.17, 0.42)**	**0.29 (0.17, 0.50)**	**0.35 (0.23, 0.54)**	**0.37 (0.26, 0.52)**	**0.41 (0.29, 0.58)**	**0.46 (0.28, 0.73)**	**0.45 (0.31, 0.66)**	0.57 (0.23, 1.45)	0.59 (0.32, 1.07)	0.66 (0.26, 1.62)	0.78 (0.59, 1.04)	0.88 (0.62, 1.24)	0.93 (0.64, 1.34)	0.93 (0.63, 1.37)	Ipi-Chemo							
**0.22 (0.13, 0.38)**	**0.25 (0.18, 0.33)**	**0.26 (0.17, 0.39)**	**0.26 (0.17, 0.40)**	**0.28 (0.17, 0.48)**	**0.35 (0.24, 0.51)**	**0.36 (0.27, 0.49)**	**0.40 (0.29, 0.55)**	**0.44 (0.28, 0.69)**	**0.44 (0.31, 0.62)**	0.56 (0.22, 1.39)	0.57 (0.32, 1.02)	0.65 (0.26, 1.56)	**0.76 (0.60, 0.97)**	0.85 (0.62, 1.17)	0.90 (0.65, 1.26)	0.91 (0.64, 1.29)	0.98 (0.67, 1.42)	Sinti-Chemo						
**0.21 (0.12, 0.36)**	**0.23 (0.17, 0.32)**	**0.24 (0.16, 0.38)**	**0.24 (0.15, 0.39)**	**0.27 (0.16, 0.46)**	**0.33 (0.22, 0.49)**	**0.34 (0.24, 0.47)**	**0.38 (0.27, 0.53)**	**0.42 (0.26, 0.67)**	**0.42 (0.29, 0.60)**	0.52 (0.21, 1.33)	**0.54 (0.30, 0.98)**	0.61 (0.26, 1.36)	**0.72 (0.55, 0.95)**	0.80 (0.57, 1.13)	0.85 (0.59, 1.22)	0.86 (0.59, 1.25)	0.92 (0.62, 1.37)	0.94 (0.65, 1.36)	Pem-Chemo					
**0.20 (0.11, 0.38)**	**0.23 (0.15, 0.35)**	**0.24 (0.14, 0.40)**	**0.24 (0.14, 0.41)**	**0.26 (0.14, 0.48)**	**0.32 (0.19, 0.52)**	**0.33 (0.21, 0.51)**	**0.37 (0.23, 0.57)**	**0.41 (0.23, 0.70)**	**0.40 (0.25, 0.65)**	0.51 (0.19, 1.34)	0.52 (0.27, 1.01)	0.59 (0.22, 1.51)	0.70 (0.47, 1.04)	0.78 (0.50, 1.22)	0.83 (0.52, 1.31)	0.83 (0.51, 1.34)	0.89 (0.54, 1.46)	0.91 (0.57, 1.46)	0.97 (0.60, 1.57)	Suge-Chemo				
**0.19 (0.11, 0.34)**	**0.22 (0.16, 0.31)**	**0.23 (0.14, 0.36)**	**0.23 (0.14, 0.37)**	**0.25 (0.15, 0.43)**	**0.30 (0.20, 0.46)**	**0.32 (0.23, 0.45)**	**0.35 (0.25, 0.50)**	**0.39 (0.24, 0.63)**	**0.39 (0.27, 0.57)**	0.49 (0.19, 1.24)	**0.50 (0.28, 0.92)**	0.57 (0.22, 1.40)	**0.67 (0.50, 0.90)**	0.75 (0.53, 1.07)	0.80 (0.55, 1.15)	0.80 (0.54, 1.18)	0.86 (0.57, 1.29)	0.88 (0.61, 1.28)	0.94 (0.63, 1.39)	0.96 (0.59, 1.58)	Camre-Chemo			
**0.19 (0.10, 0.34)**	**0.21 (0.15, 0.30)**	**0.22 (0.14, 0.35)**	**0.22 (0.14, 0.36)**	**0.24 (0.14, 0.42)**	**0.30 (0.19, 0.46)**	**0.31 (0.21, 0.44)**	**0.34 (0.24, 0.50)**	**0.38 (0.23, 0.62)**	**0.38 (0.25, 0.57)**	0.48 (0.19, 1.22)	**0.49 (0.27, 0.91)**	0.55 (0.22, 1.37)	**0.65 (0.48, 0.90)**	0.73 (0.50, 1.06)	0.77 (0.52, 1.14)	0.78 (0.52, 1.17)	0.84 (0.54, 1.28)	0.86 (0.57, 1.27)	0.91 (0.60, 1.38)	0.94 (0.56, 1.56)	0.97 (0.63, 1.49)	Nivo-Ipi-Chemo		
**0.18 (0.08, 0.37)**	**0.20 (0.11, 0.35)**	**0.21 (0.10, 0.39)**	**0.21 (0.10, 0.40)**	**0.23 (0.11, 0.46)**	**0.28 (0.14, 0.52)**	**0.29 (0.16, 0.51)**	**0.32 (0.17, 0.58)**	**0.36 (0.18, 0.69)**	**0.36 (0.19, 0.65)**	0.45 (0.16, 1.27)	**0.46 (0.21, 0.98)**	0.52 (0.18, 1.43)	0.62 (0.34, 1.06)	0.69 (0.37, 1.23)	0.73 (0.39, 1.31)	0.73 (0.39, 1.33)	0.78 (0.41, 1.45)	0.80 (0.43, 1.45)	0.85 (0.45, 1.57)	0.88 (0.44, 1.73)	0.91 (0.48, 1.69)	0.94 (0.48, 1.76)	Cemip-Chemo	
**0.18 (0.10, 0.31)**	**0.20 (0.15, 0.28)**	**0.21 (0.13, 0.33)**	**0.21 (0.14, 0.33)**	**0.23 (0.14, 0.40)**	**0.28 (0.20, 0.40)**	**0.29 (0.21, 0.41)**	**0.33 (0.23, 0.46)**	**0.36 (0.23, 0.58)**	**0.36 (0.25, 0.53)**	0.45 (0.18, 1.15)	**0.47 (0.26, 0.85)**	0.53 (0.21, 1.29)	**0.62 (0.47, 0.83)**	**0.70 (0.49, 0.99)**	**0.74 (0.55, 0.99)**	0.74 (0.51, 1.09)	0.80 (0.54, 1.19)	0.82 (0.57, 1.18)	0.87 (0.59, 1.28)	0.89 (0.55, 1.46)	0.93 (0.62, 1.39)	0.95 (0.63, 1.46)	1.02 (0.55, 1.94)	Durva-Treme-Chemo

Treatment-related general disorders and administration site conditions

(E) Investigations

Rank 1	Rank 2	Rank 3	Rank 4	Rank 5	Rank 6	Rank 7	Rank 8	Rank 9	Rank 10	Rank 11	Rank 12	Rank 13	Rank 14	Rank 15	Rank 16	Rank 17	Rank 18	Rank 19	Rank 20	Rank 21
Atezo																				
0.24 (0.01, 2.07)	Nivo-Ipi																			
0.19 (0.01, 1.16)	0.80 (0.22, 2.22)	Tisle																		
0.18 (0.01, 1.31)	0.73 (0.17, 2.79)	0.92 (0.37, 2.64)	Ave																	
0.13 (0.005, 0.76)	0.53 (0.15, 1.40)	0.67 (0.39, 1.14)	0.73 (0.26, 1.72)	Nivo																
0.08 (0.003, 0.47)	0.34 (0.10, 0.87)	0.42 (0.26, 0.68)	0.46 (0.17, 1.05)	0.63 (0.43, 0.92)	Pem															
0.05 (0.002, 0.27)	0.19 (0.05, 0.51)	0.24 (0.14, 0.41)	0.26 (0.09, 0.62)	0.36 (0.23, 0.56)	0.56 (0.38, 0.84)	Cemip														
0.03 (0.001, 0.21)	0.14 (0.04, 0.40)	0.18 (0.09, 0.34)	0.19 (0.06, 0.49)	0.26 (0.14, 0.47)	0.42 (0.26, 0.65)	0.74 (0.4, 1.34)	Pem-Ipi													
0.02 (0.0007, 0.11)	0.08 (0.02, 0.21)	0.10 (0.06, 0.16)	0.11 (0.04, 0.25)	0.14 (0.09, 0.23)	0.23 (0.16, 0.34)	0.41 (0.26, 0.64)	0.55 (0.3, 1.01)	Durva-Treme-Chemo												
0.02 (0.001, 0.07)	0.06 (0.02, 0.16)	0.08 (0.05, 0.13)	0.09 (0.03, 0.20)	0.12 (0.08, 0.17)	0.19 (0.14, 0.25)	0.33 (0.22, 0.48)	0.45 (0.26, 0.78)	0.81 (0.55, 1.19)	Atezo-Chemo											
0.02 (0.001, 0.09)	0.06 (0.02, 0.17)	0.08 (0.05, 0.13)	0.09 (0.03, 0.20)	0.12 (0.08, 0.18)	0.19 (0.13, 0.28)	0.33 (0.21, 0.52)	0.45 (0.25, 0.83)	0.82 (0.59, 1.13)	1.00 (0.69, 1.47)	Durva-Chemo										
0.01 (0.0003, 0.24)	0.05 (0.004, 0.63)	0.07 (0.01, 0.69)	0.07 (0.01, 0.83)	0.10 (0.01, 1.02)	0.16 (0.02, 1.60)	0.29 (0.03, 2.88)	0.40 (0.04, 4.00)	0.72 (0.07, 7.13)	0.88 (0.09, 8.66)	0.88 (0.09, 8.69)	Camre-Chemo									
0.01 (0.0005, 0.07)	0.05 (0.02, 0.14)	0.07 (0.04, 0.10)	0.07 (0.03, 0.16)	0.10 (0.07, 0.14)	0.16 (0.13, 0.20)	0.29 (0.21, 0.40)	0.39 (0.24, 0.65)	0.71 (0.51, 0.97)	0.87 (0.71, 1.07)	0.86 (0.63, 1.18)	0.99 (0.10, 9.49)	Chemo								
0.01 (0.0003, 0.05)	0.03 (0.01, 0.09)	0.04 (0.03, 0.07)	0.05 (0.02, 0.11)	0.07 (0.04, 0.10)	0.10 (0.07, 0.15)	0.18 (0.12, 0.28)	0.25 (0.14, 0.45)	0.45 (0.30, 0.69)	0.56 (0.39, 0.79)	0.55 (0.36, 0.85)	0.63 (0.06, 6.20)	0.64 (0.48, 0.85)	Ipi-Chemo							
0.01 (0.0002, 0.10)	0.03 (0.004, 0.25)	0.04 (0.01, 0.27)	0.05 (0.01, 0.32)	0.06 (0.01, 0.39)	0.10 (0.02, 0.61)	0.18 (0.03, 1.09)	0.24 (0.04, 1.53)	0.44 (0.07, 2.67)	0.54 (0.09, 3.25)	0.54 (0.09, 3.29)	0.62 (0.03, 10.78)	0.63 (0.11, 3.70)	0.97 (0.16, 5.88)	Sinti-Chemo						
0.01 (0.0002, 0.04)	0.03 (0.01, 0.07)	0.03 (0.02, 0.06)	0.04 (0.01, 0.09)	0.05 (0.03, 0.08)	0.08 (0.05, 0.12)	0.14 (0.08, 0.22)	0.19 (0.10, 0.35)	0.34 (0.21, 0.55)	0.42 (0.28, 0.63)	0.42 (0.26, 0.67)	0.47 (0.05, 4.69)	0.48 (0.34, 0.69)	0.75 (0.48, 1.18)	0.77 (0.13, 4.70)	Nivo-Ipi-Chemo					
0.01 (0.0002, 0.03)	0.02 (0.01, 0.07)	0.03 (0.02, 0.06)	0.03 (0.01, 0.08)	0.04 (0.02, 0.08)	0.07 (0.04, 0.12)	0.12 (0.07, 0.22)	0.17 (0.08, 0.34)	0.31 (0.17, 0.54)	0.38 (0.22, 0.63)	0.37 (0.21, 0.66)	0.43 (0.04, 4.33)	0.43 (0.26, 0.69)	0.68 (0.38, 1.18)	0.69 (0.11, 4.34)	0.90 (0.49, 1.63)	Suge-Chemo				
0.01 (0.0002, 0.03)	0.02 (0.01, 0.06)	0.03 (0.02, 0.05)	0.03 (0.01, 0.08)	0.04 (0.02, 0.07)	0.07 (0.04, 0.11)	0.12 (0.07, 0.21)	0.16 (0.08, 0.32)	0.30 (0.17, 0.51)	0.36 (0.22, 0.59)	0.36 (0.21, 0.62)	0.41 (0.04, 4.12)	0.42 (0.27, 0.65)	0.65 (0.39, 1.09)	0.67 (0.11, 4.15)	0.87 (0.50, 1.52)	0.97 (0.51, 1.86)	Tisle-Chemo			
0.01 (0.0002, 0.03)	0.02 (0.005, 0.06)	0.02 (0.01, 0.06)	0.03 (0.01, 0.08)	0.04 (0.02, 0.08)	0.06 (0.03, 0.12)	0.10 (0.04, 0.23)	0.14 (0.06, 0.34)	0.25 (0.11, 0.56)	0.31 (0.14, 0.67)	0.31 (0.13, 0.69)	0.35 (0.03, 3.79)	0.36 (0.17, 0.75)	0.55 (0.25, 1.23)	0.57 (0.08, 3.88)	0.74 (0.32, 1.68)	0.82 (0.33, 2.01)	0.85 (0.35, 2.01)	Pem-Chemo		
0.003 (0.0001, 0.02)	0.01 (0.004, 0.04)	0.02 (0.01, 0.03)	0.02 (0.01, 0.05)	0.03 (0.01, 0.05)	0.04 (0.02, 0.07)	0.07 (0.04, 0.14)	0.10 (0.05, 0.21)	0.18 (0.10, 0.34)	0.23 (0.13, 0.40)	0.23 (0.12, 0.42)	0.26 (0.02, 2.62)	0.26 (0.15, 0.44)	0.41 (0.22, 0.74)	0.42 (0.07, 2.65)	0.54 (0.29, 1.02)	0.60 (0.29, 1.23)	0.62 (0.31, 1.23)	0.74 (0.29, 1.86)	Sinti	
0.002 (7E-05, 0.01)	0.01 (0.002, 0.02)	0.01 (0.01, 0.02)	0.01 (0.004, 0.03)	0.02 (0.01, 0.03)	0.03 (0.02, 0.04)	0.05 (0.03, 0.08)	0.06 (0.03, 0.13)	0.11 (0.06, 0.20)	0.14 (0.08, 0.23)	0.14 (0.08, 0.24)	0.16 (0.02, 1.58)	0.16 (0.10, 0.26)	0.25 (0.14, 0.43)	0.26 (0.04, 1.60)	0.33 (0.18, 0.60)	0.37 (0.19, 0.73)	0.38 (0.20, 0.73)	0.45 (0.18, 1.10)	0.61 (0.30, 1.25)	Cemip-Chemo

Treatment-related investigations

(F) Metabolism and nutrition disorders

Rank 1	Rank 2	Rank 3	Rank 4	Rank 5	Rank 6	Rank 7	Rank 8	Rank 9	Rank 10	Rank 11	Rank 12	Rank 13	Rank 14	Rank 15	Rank 16	Rank 17	Rank 18	Rank 19	Rank 20	Rank 21	Rank 22	Rank 23	Rank 24
Tisle																							
1.00 (0.49, 2.05)	Durva																						
0.95 (0.52, 1.74)	0.96 (0.49, 1.82)	Cemip																					
0.77 (0.45, 1.28)	0.77 (0.42, 1.35)	0.81 (0.52, 1.24)	Nivo																				
0.65 (0.39, 1.09)	0.66 (0.36, 1.15)	0.69 (0.44, 1.06)	0.85 (0.62, 1.17)	Pem																			
0.64 (0.34, 1.22)	0.64 (0.32, 1.28)	0.67 (0.38, 1.20)	0.83 (0.51, 1.38)	0.98 (0.60, 1.62)	Ave																		
0.56 (0.30, 1.01)	0.56 (0.31, 0.97)	0.59 (0.34, 0.99)	0.73 (0.47, 1.12)	0.85 (0.55, 1.32)	0.87 (0.49, 1.55)	Durva-Treme																	
0.43 (0.24, 0.75)	0.43 (0.23, 0.79)	0.45 (0.28, 0.73)	0.56 (0.39, 0.80)	0.66 (0.45, 0.96)	0.67 (0.39, 1.15)	0.77 (0.47, 1.25)	Nivo-Ipi																
0.38 (0.19, 0.76)	0.39 (0.18, 0.79)	0.40 (0.21, 0.75)	0.50 (0.29, 0.86)	0.59 (0.37, 0.92)	0.60 (0.31, 1.17)	0.69 (0.37, 1.28)	0.90 (0.50, 1.62)	Pem-Ipi															
0.38 (0.17, 0.85)	0.38 (0.16, 0.88)	0.40 (0.19, 0.84)	0.50 (0.25, 0.99)	0.58 (0.30, 1.16)	0.60 (0.27, 1.30)	0.69 (0.33, 1.45)	0.89 (0.44, 1.82)	1.00 (0.44, 2.26)	Sinti														
0.32 (0.19, 0.55)	0.33 (0.18, 0.57)	0.34 (0.22, 0.53)	0.42 (0.31, 0.58)	0.50 (0.36, 0.68)	0.51 (0.31, 0.83)	0.58 (0.37, 0.90)	0.76 (0.51, 1.11)	0.84 (0.49, 1.47)	0.85 (0.43, 1.67)	Pem-Chemo													
0.30 (0.17, 0.53)	0.31 (0.16, 0.55)	0.32 (0.20, 0.51)	0.40 (0.28, 0.57)	0.47 (0.32, 0.67)	0.48 (0.28, 0.80)	0.55 (0.35, 0.85)	0.71 (0.46, 1.09)	0.79 (0.45, 1.42)	0.80 (0.39, 1.61)	0.94 (0.65, 1.36)	Durva-Chemo												
0.27 (0.17, 0.43)	0.27 (0.16, 0.46)	0.29 (0.20, 0.41)	0.36 (0.28, 0.44)	0.42 (0.33, 0.52)	0.43 (0.27, 0.66)	0.49 (0.34, 0.71)	0.64 (0.47, 0.87)	0.71 (0.43, 1.18)	0.71 (0.37, 1.35)	0.84 (0.67, 1.06)	0.89 (0.67, 1.20)	Chemo											
0.27 (0.16, 0.45)	0.27 (0.15, 0.48)	0.29 (0.19, 0.44)	0.36 (0.26, 0.48)	0.42 (0.31, 0.57)	0.43 (0.26, 0.69)	0.49 (0.32, 0.75)	0.64 (0.44, 0.92)	0.71 (0.41, 1.23)	0.72 (0.36, 1.40)	0.84 (0.62, 1.15)	0.90 (0.63, 1.28)	1.00 (0.81, 1.23)	Atezo										
0.26 (0.14, 0.45)	0.26 (0.14, 0.46)	0.27 (0.16, 0.44)	0.34 (0.23, 0.50)	0.39 (0.27, 0.58)	0.40 (0.23, 0.69)	0.46 (0.31, 0.68)	0.60 (0.38, 0.94)	0.67 (0.37, 1.22)	0.67 (0.33, 1.38)	0.79 (0.54, 1.18)	0.84 (0.60, 1.20)	0.94 (0.69, 1.30)	0.94 (0.64, 1.38)	Durva-Treme-									
0.24 (0.13, 0.44)	0.24 (0.12, 0.46)	0.25 (0.15, 0.43)	0.32 (0.20, 0.49)	0.37 (0.24, 0.58)	0.38 (0.21, 0.67)	0.43 (0.26, 0.74)	0.57 (0.35, 0.92)	0.63 (0.34, 1.18)	0.63 (0.30, 1.33)	0.75 (0.48, 1.16)	0.79 (0.49, 1.28)	0.89 (0.61, 1.30)	0.89 (0.57, 1.37)	0.94 (0.57, 1.55)	Nivo-Ipi-Chemo								
0.23 (0.14, 0.39)	0.23 (0.13, 0.41)	0.24 (0.16, 0.37)	0.30 (0.22, 0.41)	0.35 (0.26, 0.48)	0.36 (0.22, 0.59)	0.42 (0.27, 0.64)	0.54 (0.37, 0.79)	0.60 (0.35, 1.04)	0.60 (0.31, 1.19)	0.71 (0.52, 0.98)	0.76 (0.53, 1.09)	0.85 (0.68, 1.06)	0.85 (0.62, 1.15)	0.90 (0.61, 1.32)	0.95 (0.61, 1.48)	Atezo-Chemo							
0.23 (0.13, 0.40)	0.23 (0.12, 0.42)	0.24 (0.15, 0.38)	0.30 (0.20, 0.43)	0.35 (0.24, 0.51)	0.36 (0.21, 0.60)	0.41 (0.25, 0.66)	0.53 (0.34, 0.81)	0.59 (0.33, 1.07)	0.59 (0.29, 1.20)	0.70 (0.48, 1.02)	0.75 (0.49, 1.13)	0.83 (0.62, 1.13)	0.83 (0.58, 1.20)	0.88 (0.57, 1.37)	0.94 (0.58, 1.52)	0.98 (0.68, 1.43)	Camre-Chemo						
0.21 (0.12, 0.38)	0.21 (0.11, 0.39)	0.22 (0.13, 0.37)	0.27 (0.18, 0.41)	0.32 (0.21, 0.49)	0.33 (0.18, 0.57)	0.38 (0.22, 0.63)	0.49 (0.30, 0.78)	0.54 (0.30, 1.01)	0.55 (0.26, 1.13)	0.65 (0.42, 0.98)	0.69 (0.44, 1.08)	0.77 (0.54, 1.09)	0.77 (0.51, 1.15)	0.81 (0.51, 1.31)	0.86 (0.51, 1.45)	0.91 (0.60, 1.37)	0.92 (0.58, 1.46)	Suge-Chemo					
0.20 (0.12, 0.33)	0.20 (0.11, 0.35)	0.21 (0.13, 0.32)	0.26 (0.19, 0.35)	0.30 (0.22, 0.42)	0.31 (0.19, 0.50)	0.35 (0.23, 0.55)	0.46 (0.31, 0.67)	0.51 (0.29, 0.89)	0.51 (0.26, 1.02)	0.61 (0.44, 0.84)	0.65 (0.44, 0.94)	0.72 (0.57, 0.91)	0.72 (0.53, 0.98)	0.76 (0.51, 1.14)	0.81 (0.52, 1.27)	0.85 (0.62, 1.18)	0.86 (0.59, 1.27)	0.94 (0.62, 1.43)	Sinti-Chemo				
0.15 (0.04, 0.46)	0.15 (0.04, 0.48)	0.15 (0.05, 0.47)	0.19 (0.06, 0.56)	0.23 (0.07, 0.66)	0.23 (0.07, 0.71)	0.26 (0.08, 0.80)	0.34 (0.11, 1.02)	0.38 (0.11, 1.22)	0.39 (0.11, 1.31)	0.46 (0.15, 1.32)	0.48 (0.15, 1.43)	0.54 (0.18, 1.54)	0.54 (0.18, 1.50)	0.57 (0.18, 1.70)	0.61 (0.19, 1.85)	0.64 (0.21, 1.85)	0.65 (0.21, 1.93)	0.70 (0.22, 2.13)	0.75 (0.24, 2.19)	Atezo-Darat			
0.18 (0.09, 0.33)	0.18 (0.09, 0.34)	0.19 (0.11, 0.32)	0.23 (0.14, 0.37)	0.27 (0.17, 0.43)	0.28 (0.15, 0.50)	0.32 (0.18, 0.55)	0.41 (0.24, 0.69)	0.46 (0.24, 0.88)	0.46 (0.21, 0.98)	0.54 (0.34, 0.87)	0.58 (0.35, 0.95)	0.65 (0.42, 0.97)	0.64 (0.40, 1.02)	0.68 (0.40, 1.15)	0.73 (0.41, 1.27)	0.76 (0.47, 1.21)	0.77 (0.46, 1.29)	0.84 (0.49, 1.45)	0.90 (0.55, 1.44)	1.19 (0.39, 3.89)	Ipi-Chemo		
0.16 (0.08, 0.30)	0.16 (0.08, 0.32)	0.17 (0.09, 0.30)	0.21 (0.13, 0.34)	0.25 (0.15, 0.40)	0.25 (0.13, 0.46)	0.29 (0.16, 0.51)	0.38 (0.22, 0.64)	0.42 (0.22, 0.81)	0.42 (0.19, 0.91)	0.50 (0.30, 0.80)	0.53 (0.31, 0.88)	0.59 (0.38, 0.90)	0.59 (0.36, 0.94)	0.63 (0.36, 1.07)	0.66 (0.37, 1.18)	0.70 (0.43, 1.13)	0.71 (0.42, 1.19)	0.77 (0.44, 1.34)	0.82 (0.50, 1.33)	1.09 (0.35, 3.56)	0.91 (0.5, 1.66)	Cemip-Chemo	
0.15 (0.08, 0.26)	0.15 (0.08, 0.27)	0.16 (0.09, 0.25)	0.19 (0.13, 0.28)	0.23 (0.15, 0.33)	0.23 (0.13, 0.40)	0.26 (0.16, 0.43)	0.34 (0.22, 0.54)	0.38 (0.21, 0.70)	0.39 (0.19, 0.79)	0.46 (0.31, 0.67)	0.48 (0.31, 0.75)	0.54 (0.39, 0.74)	0.54 (0.37, 0.79)	0.57 (0.36, 0.90)	0.61 (0.37, 1.00)	0.64 (0.43, 0.94)	0.65 (0.42, 1.01)	0.70 (0.44, 1.14)	0.75 (0.50, 1.12)	1.00 (0.34, 3.17)	0.84 (0.5, 1.42)	0.92 (0.54, 1.58)	Tisle-Chemo

Treatment-related metabolism and nutrition disorders

(G) Musculoskeletal and connective tissue disorders

Rank 1	Rank 2	Rank 3	Rank 4	Rank 5	Rank 6	Rank 7	Rank 8	Rank 9	Rank 10	Rank 11	Rank 12	Rank 13	Rank 14
**Ave**													
**0.30 (0.10, 0.77)**	**Nivo**												
**0.24 (0.08, 0.64)**	0.82 (0.41, 1.59)	**Pem-Ipi**											
**0.24 (0.08, 0.65)**	0.80 (0.39, 1.64)	0.98 (0.46, 2.10)	**Cemip**										
**0.22 (0.08, 0.52)**	0.73 (0.42, 1.24)	0.89 (0.60, 1.35)	0.92 (0.48, 1.72)	**Pem**									
**0.16 (0.06, 0.38)**	**0.54 (0.32, 0.90)**	0.67 (0.38, 1.17)	0.68 (0.37, 1.25)	0.75 (0.51, 1.09)	**Atezo**								
**0.13 (0.05, 0.31)**	**0.43 (0.24, 0.75)**	**0.53 (0.29, 0.97)**	0.54 (0.28, 1.03)	**0.59 (0.37, 0.92)**	0.79 (0.52, 1.20)	**Durva-Chemo**							
**0.12 (0.04, 0.27)**	**0.40 (0.25, 0.62)**	**0.49 (0.29, 0.81)**	**0.50 (0.28, 0.86)**	**0.55 (0.40, 0.74)**	**0.73 (0.57, 0.93)**	0.92 (0.66, 1.29)	**Chemo**						
**0.11 (0.04, 0.26)**	**0.37 (0.22, 0.62)**	**0.45 (0.25, 0.81)**	**0.46 (0.24, 0.86)**	**0.51 (0.34, 0.76)**	**0.68 (0.47, 0.98)**	0.86 (0.56, 1.33)	0.93 (0.70, 1.22)	**Tisle-Chemo**					
**0.11 (0.04, 0.27)**	**0.35 (0.18, 0.66)**	**0.43 (0.22, 0.85)**	**0.44 (0.21, 0.89)**	**0.48 (0.28, 0.83)**	0.64 (0.38, 1.08)	0.81 (0.46, 1.43)	0.88 (0.56, 1.39)	0.95 (0.55, 1.61)	**Camre-Chemo**				
**0.10 (0.03, 0.25)**	**0.33 (0.18, 0.59)**	**0.41 (0.21, 0.77)**	**0.41 (0.21, 0.82)**	**0.45 (0.28, 0.74)**	**0.61 (0.38, 0.96)**	0.77 (0.46, 1.28)	0.83 (0.56, 1.22)	0.89 (0.56, 1.44)	0.95 (0.52, 1.72)	**Ipi-Chemo**			
**0.08 (0.02, 0.25)**	**0.28 (0.12, 0.65)**	**0.35 (0.14, 0.83)**	**0.36 (0.14, 0.87)**	**0.39 (0.17, 0.84)**	0.52 (0.23, 1.10)	0.66 (0.29, 1.43)	0.71 (0.33, 1.44)	0.77 (0.34, 1.63)	0.81 (0.33, 1.88)	0.86 (0.36, 1.92)	**Cemip-Chemo**		
**0.08 (0.03, 0.19)**	**0.27 (0.15, 0.46)**	**0.33 (0.18, 0.59)**	**0.34 (0.17, 0.63)**	**0.37 (0.23, 0.57)**	**0.49 (0.33, 0.73)**	**0.62 (0.39, 0.99)**	**0.67 (0.49, 0.92)**	0.72 (0.47, 1.10)	0.76 (0.44, 1.33)	0.81 (0.49, 1.33)	0.94 (0.43, 2.16)	**Pem-Chemo**	
**0.05 (0.02, 0.12)**	**0.16 (0.08, 0.30)**	**0.20 (0.10, 0.39)**	**0.20 (0.10, 0.41)**	**0.22 (0.13, 0.37)**	**0.30 (0.18, 0.49)**	**0.37 (0.21, 0.65)**	**0.40 (0.25, 0.63)**	**0.43 (0.25, 0.73)**	**0.46 (0.24, 0.87)**	**0.49 (0.27, 0.88)**	0.57 (0.24, 1.37)	0.60 (0.34, 1.04)	**Atezo-Chemo**

Treatment-related musculoskeletal and connective tissue disorders

(H) Nervous system disorders

Rank 1	Rank 2	Rank 3	Rank 4	Rank 5	Rank 6	Rank 7	Rank 8	Rank 9	Rank 10	Rank 11	Rank 12
**Pem**											
0.33 (0.09, 1.13)	**Cemip**										
**0.30 (0.09, 0.86)**	0.90 (0.31, 2.42)	**Nivo**									
**0.15 (0.05, 0.36)**	0.44 (0.17, 1.02)	**0.49 (0.24, 0.96)**	**Atezo**								
**0.05 (0.02, 0.13)**	**0.16 (0.06, 0.36)**	**0.17 (0.09, 0.33)**	**0.36 (0.22, 0.57)**	**Ipi-Chemo**							
**0.04 (0.01, 0.10)**	**0.12 (0.04, 0.28)**	**0.13 (0.06, 0.26)**	**0.26 (0.15, 0.46)**	0.73 (0.43, 1.25)	**Camre-Chemo**						
**0.04 (0.01, 0.08)**	**0.11 (0.04, 0.23)**	**0.12 (0.06, 0.21)**	**0.25 (0.17, 0.35)**	**0.70 (0.51, 0.94)**	0.95 (0.61, 1.47)	**Chemo**					
**0.04 (0.01, 0.10)**	**0.11 (0.04, 0.28)**	**0.12 (0.05, 0.27)**	**0.24 (0.12, 0.49)**	0.68 (0.34, 1.33)	0.93 (0.44, 1.94)	0.98 (0.53, 1.78)	**Tisle-Chemo**				
**0.03 (0.01, 0.09)**	**0.10 (0.03, 0.25)**	**0.11 (0.05, 0.24)**	**0.22 (0.11, 0.42)**	0.62 (0.32, 1.16)	0.84 (0.41, 1.70)	0.89 (0.50, 1.53)	0.91 (0.40, 2.07)	**Cemip-Chemo**			
**0.03 (0.01, 0.08)**	**0.09 (0.03, 0.22)**	**0.10 (0.04, 0.21)**	**0.20 (0.10, 0.37)**	0.55 (0.29, 1.02)	0.75 (0.37, 1.49)	0.80 (0.46, 1.34)	0.81 (0.36, 1.81)	0.89 (0.41, 1.94)	**Durva-Chemo**		
**0.03 (0.01, 0.07)**	**0.08 (0.03, 0.19)**	**0.09 (0.04, 0.18)**	**0.19 (0.11, 0.31)**	**0.52 (0.32, 0.84)**	0.71 (0.40, 1.25)	0.75 (0.52, 1.08)	0.76 (0.38, 1.56)	0.84 (0.43, 1.65)	0.94 (0.50, 1.82)	**Pem-Chemo**	
**0.02 (0.01, 0.04)**	**0.06 (0.02, 0.12)**	**0.06 (0.03, 0.12)**	**0.13 (0.08, 0.20)**	**0.35 (0.23, 0.54)**	**0.48 (0.29, 0.81)**	**0.51 (0.38, 0.68)**	0.52 (0.27, 1.02)	0.57 (0.31, 1.08)	0.64 (0.35, 1.19)	0.68 (0.43, 1.09)	**Atezo-Chemo**

Treatment-related nervous system disorders

(I) Respiratory, thoracic and mediastinal disorders

Rank 1	Rank 2	Rank 3	Rank 4	Rank 5	Rank 6	Rank 7	Rank 8	Rank 9	Rank 10	Rank 11	Rank 12	Rank 13
**Pem-Chemo**												
0.87 (0.53, 1.41)	**Tisle-Chemo**											
**0.78 (0.62, 0.99)**	0.90 (0.59, 1.38)	**Chemo**										
**0.63 (0.46, 0.88)**	0.73 (0.45, 1.19)	0.81 (0.64, 1.03)	**Atezo**									
**0.56 (0.32, 0.97)**	0.65 (0.34, 1.24)	0.72 (0.44, 1.18)	0.89 (0.51, 1.52)	**Sinti-Chemo**								
**0.42 (0.20, 0.84)**	0.48 (0.21, 1.05)	0.53 (0.26, 1.03)	0.66 (0.31, 1.32)	0.74 (0.31, 1.69)	**Cemip**							
**0.26 (0.09, 0.65)**	**0.29 (0.10, 0.80)**	**0.33 (0.12, 0.80)**	0.40 (0.14, 1.02)	0.45 (0.14, 1.27)	0.61 (0.18, 1.92)	**Atezo-Chemo**						
**0.21 (0.13, 0.34)**	**0.24 (0.13, 0.44)**	**0.27 (0.17, 0.41)**	**0.33 (0.20, 0.54)**	**0.37 (0.19, 0.71)**	0.50 (0.23, 1.14)	0.81 (0.30, 2.51)	**Durva-Chemo**					
**0.15 (0.02, 0.59)**	**0.17 (0.02, 0.71)**	**0.20 (0.03, 0.74)**	**0.24 (0.03, 0.93)**	0.27 (0.04, 1.12)	0.36 (0.05, 1.66)	0.59 (0.07, 3.26)	0.73 (0.10, 3.01)	**Suge-Chemo**				
**0.09 (0.003, 0.67)**	**0.11 (0.004, 0.80)**	**0.12 (0.004, 0.84)**	0.15 (0.01, 1.05)	0.17 (0.01, 1.25)	0.22 (0.01, 1.81)	0.37 (0.01, 3.43)	0.45 (0.02, 3.35)	0.62 (0.02, 9.80)	**Nivo**			
**0.10 (0.03, 0.28)**	**0.11 (0.03, 0.34)**	**0.12 (0.04, 0.34)**	**0.15 (0.05, 0.41)**	**0.17 (0.05, 0.53)**	**0.23 (0.06, 0.81)**	0.38 (0.09, 1.63)	0.46 (0.13, 1.42)	0.64 (0.11, 5.85)	1.03 (0.10, 32.89)	**Atezo-Darat**		
**0.01 (0.0005, 0.07)**	**0.01 (0.0005, 0.08)**	**0.02 (0.0006, 0.09)**	**0.02 (0.0007, 0.11)**	**0.02 (0.0008, 0.13)**	**0.03 (0.001, 0.19)**	**0.05 (0.002, 0.37)**	**0.06 (0.002, 0.35)**	0.08 (0.002, 1.11)	0.13 (0.003, 5.34)	0.13 (0.005, 1.03)	**Pem**	
**0.01 (0.0004, 0.06)**	**0.01 (0.0004, 0.07)**	**0.01 (0.0005, 0.08)**	**0.02 (0.0006, 0.10)**	**0.02 (0.0007, 0.12)**	**0.03 (0.0009, 0.17)**	**0.04 (0.001, 0.32)**	**0.05 (0.002, 0.31)**	**0.07 (0.002, 0.98)**	0.11 (0.003, 4.66)	**0.11 (0.004, 0.92)**	0.86 (0.61, 1.20)	**Pem-Ipi**

Treatment-related respiratory, thoracic and mediastinal disorders

(J) Skin and subcutaneous tissue disorders

Rank 1	Rank 2	Rank 3	Rank 4	Rank 5	Rank 6	Rank 7	Rank 8	Rank 9	Rank 10	Rank 11	Rank 12	Rank 13	Rank 14	Rank 15	Rank 16	Rank 17	Rank 18	Rank 19	Rank 20	Rank 21	Rank 22	Rank 23	Rank 24
Ave																							
0.65 (0.02, 5.35)	Tisle																						
0.44 (0.01, 3.28)	0.66 (0.15, 2.71)	Atezo																					
0.02 (0.0009, 0.14)	0.04 (0.01, 0.10)	0.05 (0.02, 0.14)	Cemip																				
0.01 (0.0004, 0.06)	0.02 (0.005, 0.04)	0.02 (0.01, 0.06)	0.45 (0.27, 0.74)	Pem																			
0.01 (0.0004, 0.06)	0.02 (0.004, 0.04)	0.02 (0.01, 0.06)	0.42 (0.21, 0.85)	0.93 (0.54, 1.63)	Sinti																		
0.007 (0.0003, 0.04)	0.01 (0.003, 0.03)	0.02 (0.01, 0.04)	0.31 (0.17, 0.56)	0.69 (0.46, 1.03)	0.74 (0.39, 1.40)	Durva																	
0.007 (0.0003, 0.04)	0.01 (0.003, 0.03)	0.02 (0.01, 0.04)	0.30 (0.16, 0.54)	0.66 (0.47, 0.93)	0.71 (0.37, 1.36)	0.96 (0.57, 1.63)	Pem-Ipi																
0.006 (0.0002, 0.03)	0.009 (0.002, 0.02)	0.01 (0.004, 0.03)	0.24 (0.13, 0.43)	0.53 (0.34, 0.81)	0.57 (0.29, 1.08)	0.77 (0.45, 1.31)	0.80 (0.46, 1.38)	Cemip-Chemo															
0.006 (0.0002, 0.03)	0.009 (0.002, 0.02)	0.01 (0.004, 0.03)	0.23 (0.14, 0.39)	0.52 (0.39, 0.68)	0.56 (0.31, 0.97)	0.75 (0.49, 1.15)	0.78 (0.51, 1.21)	0.98 (0.63, 1.53)	Nivo														
0.005 (0.0002, 0.03)	0.008 (0.002, 0.02)	0.01 (0.004, 0.03)	0.21 (0.13, 0.33)	0.46 (0.38, 0.54)	0.49 (0.29, 0.83)	0.67 (0.46, 0.96)	0.69 (0.47, 1.02)	0.87 (0.59, 1.29)	0.88 (0.71, 1.10)	Chemo													
0.004 (0.0002, 0.02)	0.006 (0.002, 0.02)	0.009 (0.003, 0.02)	0.17 (0.09, 0.29)	0.37 (0.26, 0.52)	0.40 (0.22, 0.72)	0.54 (0.37, 0.76)	0.56 (0.34, 0.91)	0.70 (0.43, 1.15)	0.71 (0.49, 1.03)	0.81 (0.60, 1.09)	Durva-Treme												
0.003 (0.0001, 0.02)	0.005 (0.001, 0.01)	0.008 (0.003, 0.02)	0.14 (0.08, 0.24)	0.32 (0.25, 0.41)	0.34 (0.19, 0.59)	0.46 (0.31, 0.70)	0.48 (0.31, 0.74)	0.60 (0.39, 0.93)	0.61 (0.46, 0.82)	0.69 (0.57, 0.84)	0.86 (0.60, 1.23)	Atezo-Chemo											
0.003 (0.0001, 0.02)	0.004 (0.001, 0.01)	0.007 (0.002, 0.02)	0.12 (0.07, 0.22)	0.27 (0.18, 0.40)	0.29 (0.15, 0.55)	0.39 (0.23, 0.66)	0.41 (0.24, 0.69)	0.51 (0.30, 0.88)	0.52 (0.34, 0.80)	0.59 (0.41, 0.85)	0.73 (0.46, 1.17)	0.85 (0.56, 1.28)	Suge-Chemo										
0.003 (9E-05, 0.01)	0.004 (0.001, 0.01)	0.006 (0.002, 0.01)	0.11 (0.05, 0.20)	0.23 (0.14, 0.38)	0.25 (0.12, 0.50)	0.34 (0.19, 0.61)	0.35 (0.19, 0.64)	0.44 (0.24, 0.81)	0.45 (0.27, 0.74)	0.51 (0.32, 0.80)	0.63 (0.36, 1.09)	0.74 (0.44, 1.20)	0.86 (0.48, 1.55)	Sinti-Chemo									
0.002 (9E-05, 0.01)	0.004 (0.001, 0.01)	0.006 (0.002, 0.01)	0.10 (0.06, 0.18)	0.22 (0.15, 0.33)	0.24 (0.13, 0.45)	0.33 (0.20, 0.54)	0.34 (0.20, 0.56)	0.42 (0.25, 0.71)	0.43 (0.29, 0.64)	0.49 (0.34, 0.69)	0.60 (0.38, 0.95)	0.70 (0.47, 1.04)	0.82 (0.50, 1.36)	0.96 (0.54, 1.71)	Camre-Chemo								
0.002 (8E-05, 0.01)	0.003 (9E-04, 0.008)	0.005 (0.002, 0.01)	0.09 (0.05, 0.15)	0.19 (0.14, 0.27)	0.21 (0.11, 0.38)	0.28 (0.18, 0.44)	0.29 (0.18, 0.47)	0.37 (0.22, 0.60)	0.37 (0.26, 0.54)	0.42 (0.31, 0.57)	0.53 (0.36, 0.77)	0.61 (0.43, 0.87)	0.72 (0.45, 1.15)	0.83 (0.48, 1.45)	0.87 (0.55, 1.37)	Durva-Chemo							
0.002 (7E-05, 0.01)	0.003 (0.0009, 0.008)	0.005 (0.002, 0.01)	0.09 (0.05, 0.15)	0.19 (0.14, 0.26)	0.20 (0.11, 0.36)	0.28 (0.18, 0.43)	0.29 (0.18, 0.46)	0.36 (0.23, 0.57)	0.37 (0.26, 0.51)	0.42 (0.32, 0.53)	0.51 (0.35, 0.76)	0.60 (0.43, 0.82)	0.70 (0.45, 1.10)	0.81 (0.48, 1.39)	0.85 (0.56, 1.31)	0.98 (0.66, 1.45)	Pem-Chemo						
0.002 (7E-05, 0.01)	0.003 (0.0008, 0.008)	0.005 (0.001, 0.01)	0.08 (0.05, 0.14)	0.18 (0.13, 0.26)	0.20 (0.11, 0.36)	0.27 (0.17, 0.42)	0.28 (0.17, 0.46)	0.35 (0.21, 0.57)	0.35 (0.24, 0.52)	0.40 (0.29, 0.55)	0.50 (0.35, 0.70)	0.58 (0.40, 0.84)	0.67 (0.42, 1.10)	0.78 (0.45, 1.39)	0.82 (0.51, 1.31)	0.94 (0.68, 1.31)	0.96 (0.64, 1.46)	Durva-Treme-Chemo					
0.002 (7E-05, 0.01)	0.003 (0.0008, 0.008)	0.004 (0.001, 0.01)	0.08 (0.04, 0.14)	0.17 (0.12, 0.26)	0.19 (0.10, 0.35)	0.25 (0.15, 0.42)	0.26 (0.16, 0.45)	0.33 (0.19, 0.56)	0.34 (0.22, 0.51)	0.38 (0.26, 0.54)	0.47 (0.29, 0.75)	0.55 (0.36, 0.82)	0.64 (0.38, 1.08)	0.75 (0.42, 1.35)	0.78 (0.47, 1.28)	0.90 (0.56, 1.43)	0.92 (0.59, 1.43)	0.95 (0.59, 1.54)	Tisle-Chemo				
0.002 (6E-05, 0.009)	0.002 (0.0007, 0.006)	0.004 (0.001, 0.009)	0.07 (0.04, 0.11)	0.14 (0.10, 0.20)	0.16 (0.08, 0.28)	0.21 (0.13, 0.34)	0.22 (0.13, 0.36)	0.27 (0.17, 0.45)	0.28 (0.19, 0.40)	0.32 (0.23, 0.43)	0.39 (0.26, 0.60)	0.46 (0.32, 0.65)	0.53 (0.33, 0.86)	0.62 (0.36, 1.08)	0.65 (0.41, 1.03)	0.75 (0.49, 1.14)	0.76 (0.51, 1.13)	0.79 (0.51, 1.23)	0.83 (0.52, 1.33)	Ipi-Chemo			
0.001 (5E-05, 0.008)	0.002 (0.0006, 0.006)	0.003 (0.001, 0.008)	0.06 (0.03, 0.10)	0.14 (0.10, 0.19)	0.15 (0.08, 0.26)	0.20 (0.12, 0.31)	0.20 (0.13, 0.33)	0.26 (0.16, 0.42)	0.26 (0.19, 0.35)	0.30 (0.22, 0.39)	0.37 (0.24, 0.55)	0.43 (0.30, 0.60)	0.50 (0.32, 0.79)	0.58 (0.34, 1.00)	0.61 (0.39, 0.95)	0.70 (0.46, 1.05)	0.71 (0.49, 1.04)	0.74 (0.48, 1.13)	0.78 (0.49, 1.23)	0.94 (0.62, 1.42)	Nivo-Ipi		
0.0004 (1E-05, 0.003)	0.0006 (8E-05, 0.003)	0.0009 (0.0001, 0.004)	0.02 (0.003, 0.06)	0.04 (0.01, 0.13)	0.04 (0.01, 0.15)	0.05 (0.01, 0.19)	0.05 (0.01, 0.20)	0.07 (0.01, 0.25)	0.07 (0.01, 0.25)	0.08 (0.02, 0.27)	0.10 (0.02, 0.35)	0.11 (0.02, 0.40)	0.13 (0.03, 0.49)	0.16 (0.03, 0.58)	0.16 (0.03, 0.59)	0.19 (0.04, 0.67)	0.19 (0.04, 0.64)	0.20 (0.04, 0.71)	0.21 (0.04, 0.75)	0.25 (0.05, 0.90)	0.27 (0.05, 0.95)	Dostar-Chemo	
0.0004 (1E-05, 0.002)	0.0006 (0.0002, 0.002)	0.0009 (0.0003, 0.002)	0.02 (0.01, 0.03)	0.04 (0.02, 0.06)	0.04 (0.02, 0.08)	0.05 (0.03, 0.09)	0.05 (0.03, 0.10)	0.07 (0.04, 0.12)	0.07 (0.04, 0.11)	0.08 (0.05, 0.12)	0.10 (0.05, 0.17)	0.11 (0.06, 0.18)	0.13 (0.07, 0.24)	0.15 (0.08, 0.29)	0.16 (0.09, 0.28)	0.18 (0.10, 0.32)	0.19 (0.10, 0.32)	0.19 (0.11, 0.34)	0.20 (0.11, 0.37)	0.24 (0.14, 0.43)	0.26 (0.15, 0.45)	0.97 (0.25, 5.06)	Nivo-Ipi-Chemo

Treatment-related skin and subcutaneous tissue disorders

(K) Infections and infestations

Rank 1	Rank 2	Rank 3	Rank 4	Rank 5	Rank 6	Rank 7	Rank 8	Rank 9	Rank 10	Rank 11
**Ave**										
0.29 (0.03, 1.49)	**Cemip**									
0.24 (0.03, 1.37)	0.85 (0.23, 3.16)	**Sinti**								
**0.15 (0.02, 0.70)**	0.51 (0.16, 1.56)	0.60 (0.17, 2.02)	**Atezo**							
**0.12 (0.02, 0.60)**	0.43 (0.13, 1.34)	0.51 (0.14, 1.72)	0.84 (0.29, 2.43)	**Camre-Chemo**						
**0.11 (0.02, 0.43)**	**0.38 (0.15, 0.86)**	0.45 (0.16, 1.15)	0.74 (0.35, 1.54)	0.88 (0.41, 1.86)	**Chemo**					
**0.07 (0.01, 0.34)**	**0.25 (0.08, 0.73)**	**0.29 (0.08, 0.95)**	0.49 (0.17, 1.34)	0.58 (0.20, 1.60)	0.66 (0.31, 1.31)	**Suge-Chemo**				
**0.07 (0.01, 0.31)**	**0.25 (0.09, 0.66)**	**0.30 (0.10, 0.86)**	0.49 (0.2, 1.19)	0.58 (0.24, 1.43)	0.67 (0.41, 1.08)	1.01 (0.43, 2.48)	**Sinti-Chemo**			
**0.05 (0.003, 0.49)**	0.18 (0.02, 1.25)	0.21 (0.02, 1.55)	0.36 (0.04, 2.32)	0.42 (0.05, 2.77)	0.48 (0.06, 2.70)	0.74 (0.08, 4.77)	0.73 (0.08, 4.36)	**Atezo-Chemo**		
**0.07 (0.01, 0.28)**	**0.24 (0.09, 0.59)**	**0.28 (0.09, 0.78)**	0.46 (0.2, 1.06)	0.55 (0.23, 1.28)	**0.63 (0.42, 0.92)**	0.95 (0.43, 2.22)	0.94 (0.50, 1.76)	1.29 (0.22, 10.92)	**Durva-Chemo**	
**0.01 (0.0003, 0.20)**	**0.05 (0.002, 0.52)**	**0.06 (0.002, 0.65)**	**0.11 (0.004, 0.74)**	0.12 (0.004, 1.17)	0.14 (0.005, 1.17)	0.21 (0.01, 2.02)	0.21 (0.01, 1.87)	0.29 (0.01, 5.80)	0.23 (0.01, 1.94)	**Atezo-Darat**

Treatment-related infections and infestations

Supplementary Figure S4. Odds ratio (95% CrI) of system organ classes specific treatment-related adverse events associated with each treatment regimen. (A) Blood and lymphatic system disorders; (B) Endocrine disorders; (C) Gastrointestinal disorders; (D) General disorders and administration site conditions; (E) Investigations; (F) Metabolism and nutrition disorders; (G) Musculoskeletal and connective tissue disorders; (H) Nervous system disorders; (I) Respiratory, thoracic and mediastinal disorders; (J) Skin and subcutaneous tissue disorders; (K) Infections and infestations. Atezo: atezolizumab; Ave: avelumab; Beva: bevacizumab; Camre: camrelizumab; Cemip: cemiplimab; Chemo, chemotherapy; Darat: daratumumab; Dostra, dostarlimab; Durva: durvalumab; Ipi: ipilimumab; Nivo: nivolumab; NSCLC: non-small cell lung cancer; Pem: pembrolizumab; Sint: sintilimab; Sugema: sugemalimab; Tisle: tislelizumab; Treme: tremelimumab.

(A) Endocrine disorders

Rank 1	Rank 2	Rank 3	Rank 4	Rank 5	Rank 6	Rank 7	Rank 8	Rank 9	Rank 10	Rank 11	Rank 12	Rank 13	Rank 14	Rank 15	Rank 16	Rank 17	Rank 18
Durva																	
0.98 (0.32, 3.24)	Atezo																
0.97 (0.37, 2.59)	0.99 (0.33, 2.79)	Durva-Chemo															
0.98 (0.26, 3.54)	0.99 (0.32, 2.81)	1.01 (0.29, 3.35)	Tisle-Chemo														
0.68 (0.25, 2.04)	0.68 (0.33, 1.52)	0.70 (0.28, 1.89)	0.69 (0.28, 1.97)	Pem													
0.62 (0.11, 2.77)	0.63 (0.13, 2.28)	0.64 (0.12, 2.65)	0.64 (0.12, 2.70)	0.92 (0.20, 2.96)	Cemip												
**0.51 (0.31, 0.83)**	0.52 (0.17, 1.49)	0.53 (0.22, 1.23)	0.52 (0.15, 1.82)	0.75 (0.27, 1.89)	0.82 (0.19, 4.38)	Durva-Treme											
0.49 (0.24, 1.02)	0.50 (0.16, 1.46)	0.51 (0.23, 1.07)	0.50 (0.15, 1.78)	0.73 (0.26, 1.86)	0.79 (0.19, 4.28)	0.97 (0.56, 1.69)	Durva-Treme-Chemo										
0.44 (0.15, 1.42)	0.44 (0.19, 1.10)	0.45 (0.16, 1.33)	0.45 (0.16, 1.38)	0.64 (0.41, 1.00)	0.70 (0.20, 3.45)	0.86 (0.31, 2.64)	0.89 (0.31, 2.70)	Pem-Ipi									
0.37 (0.07, 1.62)	0.38 (0.08, 1.31)	0.38 (0.07, 1.54)	0.38 (0.07, 1.58)	0.55 (0.12, 1.70)	0.60 (0.10, 3.63)	0.73 (0.14, 3.03)	0.75 (0.14, 3.12)	0.86 (0.18, 2.88)	Nivo								
0.38 (0.10, 1.44)	0.39 (0.12, 1.14)	0.40 (0.11, 1.36)	0.39 (0.11, 1.39)	0.57 (0.18, 1.44)	0.62 (0.14, 3.36)	0.76 (0.20, 2.67)	0.78 (0.21, 2.73)	0.89 (0.27, 2.48)	1.04 (0.24, 5.58)	Atezo-Chemo							
0.25 (0.01, 1.87)	0.25 (0.01, 1.61)	0.26 (0.01, 1.81)	0.26 (0.01, 1.86)	0.37 (0.01, 2.17)	0.40 (0.01, 3.93)	0.49 (0.02, 3.52)	0.50 (0.02, 3.62)	0.57 (0.02, 3.58)	0.67 (0.02, 6.57)	0.65 (0.02, 4.95)	Cemip-Chemo						
0.24 (0.01, 1.87)	0.25 (0.01, 1.64)	0.25 (0.01, 1.81)	0.25 (0.01, 1.87)	0.37 (0.01, 2.18)	0.39 (0.01, 3.94)	0.48 (0.02, 3.54)	0.50 (0.02, 3.66)	0.56 (0.02, 3.61)	0.66 (0.02, 6.51)	0.64 (0.02, 4.95)	1.00 (0.02, 40.03)	Tisle					
0.20 (0.02, 1.00)	**0.20 (0.03, 0.84)**	**0.20 (0.03, 0.96)**	**0.20 (0.03, 0.99)**	0.29 (0.04, 1.10)	0.32 (0.04, 2.23)	0.39 (0.05, 1.89)	0.40 (0.05, 1.94)	0.45 (0.06, 1.85)	0.53 (0.06, 3.66)	0.51 (0.06, 2.65)	0.79 (0.06, 27.04)	0.80 (0.06, 28.06)	Camre-Chemo				
0.17 (0.01, 1.29)	0.17 (0.01, 1.12)	0.17 (0.01, 1.28)	0.17 (0.01, 1.27)	0.25 (0.01, 1.50)	0.27 (0.01, 2.70)	0.33 (0.01, 2.46)	0.34 (0.01, 2.54)	0.39 (0.01, 2.47)	0.46 (0.01, 4.54)	0.44 (0.01, 3.39)	0.68 (0.02, 28.15)	0.69 (0.02, 28.85)	0.86 (0.03, 11.4)	Ave			
0.16 (0.01, 1.26)	0.16 (0.01, 1.09)	0.16 (0.01, 1.23)	0.16 (0.01, 1.24)	0.24 (0.01, 1.47)	0.26 (0.01, 2.57)	0.31 (0.01, 2.37)	0.32 (0.01, 2.45)	0.37 (0.01, 2.40)	0.43 (0.01, 4.32)	0.42 (0.01, 3.27)	0.64 (0.02, 26.65)	0.64 (0.02, 28.31)	0.81 (0.02, 10.96)	0.95 (0.02, 40.65)	Sinti		
**0.18 (0.03, 0.83)**	**0.19 (0.04, 0.68)**	**0.19 (0.03, 0.80)**	**0.19 (0.03, 0.82)**	**0.27 (0.06, 0.88)**	0.30 (0.05, 1.86)	0.36 (0.07, 1.56)	0.37 (0.07, 1.60)	0.42 (0.08, 1.49)	**0.49 (0.35, 0.70)**	0.48 (0.09, 2.17)	0.74 (0.07, 24.43)	0.75 (0.07, 24.13)	0.93 (0.13, 8.23)	1.09 (0.11, 34.94)	1.15 (0.11, 36.93)	Nivo-Ipi	
**0.06 (0.002, 0.45)**	**0.06 (0.002, 0.39)**	**0.06 (0.002, 0.44)**	**0.06 (0.002, 0.44)**	**0.09 (0.003, 0.52)**	**0.09 (0.003, 0.94)**	**0.12 (0.004, 0.85)**	**0.12 (0.004, 0.80)**	**0.14 (0.005, 0.86)**	0.16 (0.005, 1.56)	0.15 (0.005, 1.17)	0.24 (0.01, 9.86)	0.24 (0.01, 9.75)	0.30 (0.01, 3.96)	0.34 (0.01, 14.78)	0.37 (0.01, 14.72)	0.32 (0.01, 3.24)	Nivo-Ipi-Chemo

Immune-related endocrine disorders

(B) Gastrointestinal disorders

Rank 1	Rank 2	Rank 3	Rank 4	Rank 5	Rank 6	Rank 7	Rank 8	Rank 9	Rank 10	Rank 11	Rank 12	Rank 13	Rank 14	Rank 15	Rank 16	Rank 17	Rank 18	Rank 19	Rank 20	Rank 21
Nivo																				
**0.64 (0.45, 0.92)**	Nivo-Ipi																			
1.06 (0.03, 41.02)	1.65 (0.04, 63.99)	Cemip-Chemo																		
**0.52 (0.38, 0.69)**	0.80 (0.50, 1.29)	0.49 (0.01, 19.03)	Chemo																	
**0.36 (0.13, 0.93)**	0.56 (0.19, 1.55)	0.34 (0.01, 14.69)	0.70 (0.26, 1.71)	Sinti-Chemo																
0.43 (0.01, 5.69)	0.67 (0.02, 9.12)	0.38 (0.003, 35.30)	0.84 (0.03, 10.86)	1.20 (0.03, 18.81)	Suge-Chemo															
**0.16 (0.02, 0.66)**	0.25 (0.03, 1.08)	0.14 (0.002, 7.40)	0.31 (0.04, 1.23)	0.44 (0.05, 2.46)	0.36 (0.01, 15.10)	Tisle-Chemo														
**0.15 (0.05, 0.38)**	**0.24 (0.08, 0.63)**	0.14 (0.003, 6.10)	**0.29 (0.11, 0.69)**	0.42 (0.11, 1.56)	0.35 (0.02, 12.12)	0.95 (0.17, 8.15)	Pem-Chemo													
**0.15 (0.09, 0.23)**	**0.23 (0.12, 0.41)**	0.14 (0.004, 5.48)	**0.28 (0.19, 0.41)**	0.40 (0.15, 1.16)	0.34 (0.03, 10.94)	0.91 (0.22, 6.79)	0.96 (0.37, 2.82)	Ipi-Chemo												
**0.14 (0.04, 0.42)**	**0.22 (0.06, 0.70)**	0.13 (0.003, 6.13)	**0.28 (0.08, 0.78)**	0.40 (0.08, 1.67)	0.33 (0.02, 12.08)	0.91 (0.14, 8.34)	0.95 (0.20, 4.04)	0.99 (0.26, 2.99)	Atezo											
0.14 (0.005, 1.37)	0.22 (0.01, 2.20)	0.13 (0.001, 9.56)	0.28 (0.01, 2.60)	0.40 (0.01, 4.60)	0.33 (0.01, 19.75)	0.91 (0.02, 17.76)	0.95 (0.03, 11.14)	0.99 (0.03, 9.55)	1.00 (0.03, 13.03)	Ave										
**0.13 (0.03, 0.42)**	**0.20 (0.04, 0.69)**	0.12 (0.002, 5.59)	**0.26 (0.06, 0.78)**	0.36 (0.06, 1.63)	0.30 (0.02, 11.33)	0.82 (0.11, 7.87)	0.86 (0.15, 3.96)	0.90 (0.19, 2.96)	0.91 (0.15, 4.97)	0.90 (0.06, 31.35)	Atezo-Chemo									
**0.10 (0.003, 0.82)**	0.16 (0.01, 1.34)	0.09 (0.0007, 6.20)	0.20 (0.01, 1.55)	0.28 (0.01, 2.84)	0.23 (0.004, 12.86)	0.64 (0.02, 11.07)	0.66 (0.02, 6.75)	0.69 (0.02, 5.75)	0.69 (0.02, 8.07)	0.69 (0.01, 35.89)	0.77 (0.02, 10.12)	Cemip								
**0.09 (0.01, 0.54)**	**0.13 (0.01, 0.87)**	0.08 (0.001, 4.62)	0.17 (0.02, 1.01)	0.24 (0.02, 1.88)	0.20 (0.01, 9.57)	0.55 (0.04, 7.71)	0.56 (0.05, 4.52)	0.59 (0.06, 3.74)	0.60 (0.05, 5.43)	0.60 (0.03, 25.61)	0.66 (0.06, 6.89)	0.87 (0.04, 37.05)	Durva-Chemo							
**0.06 (0.01, 0.28)**	**0.10 (0.01, 0.46)**	0.06 (0.001, 2.97)	**0.12 (0.02, 0.53)**	0.17 (0.02, 1.03)	0.14 (0.01, 6.11)	0.39 (0.03, 4.53)	0.41 (0.05, 2.49)	0.43 (0.05, 1.98)	0.43 (0.05, 3.07)	0.43 (0.02, 16.39)	0.48 (0.05, 3.91)	0.62 (0.03, 24.07)	0.73 (0.14, 3.32)	Durva						
**0.07 (0.01, 0.26)**	**0.10 (0.01, 0.42)**	0.06 (0.001, 3.00)	**0.13 (0.02, 0.48)**	**0.18 (0.02, 0.97)**	0.15 (0.01, 6.15)	0.42 (0.04, 4.46)	0.44 (0.05, 2.35)	0.46 (0.06, 1.82)	0.46 (0.05, 2.9)	0.45 (0.02, 16.58)	0.51 (0.05, 3.77)	0.65 (0.04, 24.02)	0.76 (0.06, 10.04)	1.06 (0.09, 11.86)	Pem					
**0.05 (0.01, 0.18)**	**0.07 (0.01, 0.29)**	0.04 (0.0007, 2.09)	**0.09 (0.01, 0.34)**	**0.13 (0.01, 0.67)**	0.10 (0.004, 4.28)	0.29 (0.03, 3.11)	0.30 (0.04, 1.63)	0.32 (0.04, 1.27)	0.32 (0.04, 2.02)	0.32 (0.02, 11.68)	0.35 (0.04, 2.61)	0.45 (0.03, 16.55)	0.53 (0.04, 6.95)	0.73 (0.06, 8.36)	0.69 (0.06, 7.2)	Camre-Chemo				
**0.02 (0.003, 0.09)**	**0.04 (0.005, 0.15)**	0.02 (0.0004, 1.06)	**0.05 (0.01, 0.17)**	**0.06 (0.01, 0.35)**	0.05 (0.002, 2.14)	0.15 (0.01, 1.56)	**0.15 (0.02, 0.83)**	**0.16 (0.02, 0.65)**	0.16 (0.02, 1.04)	0.16 (0.01, 5.81)	0.18 (0.02, 1.35)	0.23 (0.01, 8.39)	**0.27 (0.07, 0.79)**	0.37 (0.12, 1.05)	0.35 (0.03, 3.73)	0.50 (0.05, 5.52)	Durva-Treme-Chemo			
**0.02 (0.002, 0.07)**	**0.03 (0.004, 0.11)**	**0.02 (0.0003, 0.81)**	**0.04 (0.005, 0.13)**	**0.05 (0.01, 0.26)**	0.04 (0.002, 1.64)	0.11 (0.01, 1.19)	**0.12 (0.01, 0.62)**	**0.12 (0.02, 0.48)**	**0.12 (0.01, 0.78)**	0.12 (0.01, 4.47)	0.14 (0.01, 1.01)	0.18 (0.01, 6.46)	**0.21 (0.05, 0.76)**	**0.29 (0.13, 0.60)**	0.27 (0.03, 2.83)	0.39 (0.04, 4.16)	0.78 (0.37, 1.62)	Durva-Treme		
**0.01 (0.0007, 0.05)**	**0.01 (0.001, 0.08)**	**0.01 (0.0001, 0.44)**	**0.02 (0.001, 0.10)**	**0.02 (0.002, 0.18)**	**0.02 (0.0006, 0.91)**	**0.05 (0.003, 0.73)**	**0.05 (0.004, 0.43)**	**0.06 (0.005, 0.36)**	**0.06 (0.004, 0.52)**	0.06 (0.002, 2.49)	**0.06 (0.004, 0.65)**	0.08 (0.003, 3.56)	0.09 (0.005, 1.61)	0.13 (0.01, 1.95)	**0.13 (0.03, 0.39)**	0.18 (0.01, 2.53)	0.35 (0.02, 5.01)	0.46 (0.03, 6.3)	Pem-Ipi	
**0.01 (0.0003, 0.04)**	**0.01 (0.0004, 0.07)**	**0.01 (5E-05, 0.41)**	**0.01 (0.0005, 0.08)**	**0.02 (0.0007, 0.16)**	**0.02 (0.0003, 0.80)**	**0.05 (0.001, 0.66)**	**0.05 (0.002, 0.38)**	**0.05 (0.002, 0.31)**	**0.05 (0.002, 0.46)**	0.05 (0.001, 2.25)	**0.06 (0.002, 0.57)**	0.07 (0.002, 3.18)	0.08 (0.002, 1.47)	0.12 (0.003, 1.77)	0.11 (0.003, 1.54)	0.16 (0.005, 2.22)	0.32 (0.01, 4.57)	0.42 (0.01, 5.74)	0.90 (0.02, 17.98)	Nivo-Ipi-Chemo

Immune-related gastrointestinal disorders

(C) Hepatobiliary disorders

Rank 1	Rank 2	Rank 3	Rank 4	Rank 5	Rank 6	Rank 7	Rank 8	Rank 9	Rank 10	Rank 11	Rank 12	Rank 13	Rank 14	Rank 15	Rank 16
**Chemo**															
0.80 (0.48, 1.33)	**Nivo**														
1.06 (0.03, 40.94)	1.33 (0.03, 52.68)	**Durva**													
**0.53 (0.38, 0.74)**	0.67 (0.36, 1.23)	0.50 (0.01, 19.13)	**Atezo-Chemo**												
**0.51 (0.27, 0.97)**	**0.64 (0.43, 0.94)**	0.48 (0.01, 18.87)	0.96 (0.46, 1.98)	**Nivo-Ipi**											
**0.54 (0.02, 5.02)**	0.67 (0.02, 6.67)	0.47 (0.004, 35.26)	1.01 (0.03, 9.68)	1.05 (0.03, 10.78)	**Tisle**										
**0.43 (0.28, 0.65)**	0.53 (0.27, 1.03)	0.40 (0.01, 15.29)	0.80 (0.46, 1.38)	0.83 (0.39, 1.81)	0.79 (0.08, 24.75)	**Atezo**									
0.34 (0.05, 1.39)	0.42 (0.05, 1.91)	0.30 (0.01, 15.16)	0.64 (0.08, 2.73)	0.66 (0.08, 3.18)	0.62 (0.03, 23.92)	0.79 (0.10, 3.49)	**Tisle-Chemo**								
**0.35 (0.12, 0.89)**	0.44 (0.14, 1.27)	0.33 (0.01, 13.58)	0.66 (0.22, 1.79)	0.69 (0.20, 2.16)	0.66 (0.06, 22.18)	0.83 (0.26, 2.30)	1.05 (0.17, 9.58)	**Pem-Chemo**							
0.30 (0.01, 2.19)	0.37 (0.01, 2.95)	0.25 (0.002, 16.75)	0.56 (0.02, 4.27)	0.58 (0.02, 4.81)	0.54 (0.01, 27.03)	0.70 (0.02, 5.46)	0.88 (0.02, 15.00)	0.84 (0.03, 8.28)	**Suge-Chemo**						
0.27 (0.01, 2.50)	0.33 (0.01, 3.34)	0.23 (0.002, 17.59)	0.50 (0.02, 4.82)	0.52 (0.02, 5.38)	0.50 (0.01, 27.91)	0.62 (0.02, 6.11)	0.80 (0.02, 15.99)	0.76 (0.02, 9.15)	0.92 (0.02, 51.03)	**Cemip**					
0.25 (0.04, 1.39)	0.32 (0.05, 1.87)	0.24 (0.004, 12.78)	0.48 (0.07, 2.70)	0.50 (0.07, 3.05)	0.48 (0.03, 20.21)	0.59 (0.09, 3.44)	0.77 (0.07, 10.17)	0.72 (0.09, 5.36)	0.89 (0.06, 36.56)	0.97 (0.05, 41.46)	**Pem-Ipi**				
**0.26 (0.07, 0.71)**	0.32 (0.08, 1.01)	0.24 (0.01, 10.34)	0.48 (0.13, 1.42)	0.50 (0.12, 1.70)	0.47 (0.04, 16.26)	0.60 (0.16, 1.83)	0.76 (0.11, 7.14)	0.72 (0.15, 3.20)	0.87 (0.08, 29.33)	0.95 (0.07, 32.78)	1.00 (0.26, 3.74)	**Pem**			
**0.08 (0.003, 0.53)**	**0.10 (0.004, 0.72)**	**0.08 (0.003, 0.50)**	0.16 (0.01, 1.03)	0.16 (0.01, 1.18)	0.15 (0.003, 7.16)	0.19 (0.01, 1.31)	0.24 (0.01, 3.80)	0.23 (0.01, 2.03)	0.28 (0.01, 12.78)	0.30 (0.01, 14.25)	0.31 (0.01, 4.54)	0.32 (0.01, 3.14)	**Durva-Treme**		
**0.04 (0.001, 0.39)**	**0.05 (0.002, 0.52)**	**0.04 (0.001, 0.37)**	**0.08 (0.002, 0.75)**	**0.08 (0.002, 0.84)**	0.08 (0.001, 4.30)	**0.10 (0.003, 0.95)**	0.12 (0.003, 2.45)	0.12 (0.003, 1.41)	0.14 (0.003, 7.70)	0.15 (0.003, 8.58)	0.17 (0.004, 2.98)	0.16 (0.005, 2.15)	0.52 (0.15, 1.57)	**Durva-Treme-**	
**0.03 (0.004, 0.10)**	**0.04 (0.005, 0.14)**	0.03 (0.0004, 1.21)	**0.05 (0.01, 0.20)**	**0.05 (0.01, 0.23)**	0.05 (0.003, 1.93)	**0.07 (0.01, 0.25)**	**0.08 (0.01, 0.89)**	**0.08 (0.01, 0.42)**	0.09 (0.01, 3.43)	0.10 (0.01, 3.89)	0.11 (0.01, 1.03)	**0.11 (0.01, 0.67)**	0.34 (0.02, 11.4)	0.67 (0.04, 26.22)	**Camre-Chemo**

Immune-related hepatobiliary disorders

(D) Injury, poisoning and procedural complications

Rank 1	Rank 2	Rank 3	Rank 4	Rank 5	Rank 6
**Pem**					
0.57 (0.20, 1.62)	**Nivo**				
**0.37 (0.23, 0.59)**	0.65 (0.26, 1.63)	**Chemo**			
**0.23 (0.10, 0.52)**	0.4 (0.13, 1.25)	0.62 (0.31, 1.19)	**Pem-Chemo**		
**0.09 (0.02, 0.33)**	**0.16 (0.03, 0.73)**	**0.25 (0.05, 0.81)**	0.4 (0.08, 1.59)	**Atezo**	
**0.04 (0.002, 0.26)**	**0.07 (0.002, 0.54)**	**0.11 (0.004, 0.66)**	0.18 (0.01, 1.22)	0.44 (0.01, 4.62)	**Atezo-Chemo**

Immune-related injury, poisoning and procedural complications

(E) Investigations

Rank 1	Rank 2	Rank 3	Rank 4	Rank 5	Rank 6	Rank 7	Rank 8	Rank 9	Rank 10	Rank 11	Rank 12	Rank 13
Chemo												
0.98 (0.54, 1.73)	Atezo-Chemo											
0.73 (0.51, 1.03)	0.74 (0.38, 1.47)	Sinti-Chemo										
**0.40 (0.27, 0.59)**	**0.41 (0.21, 0.83)**	**0.55 (0.33, 0.92)**	Nivo									
0.27 (0.01, 2.48)	0.27 (0.01, 2.73)	0.37 (0.01, 3.51)	0.66 (0.02, 6.36)	Cemip								
**0.21 (0.07, 0.50)**	**0.21 (0.06, 0.62)**	**0.28 (0.09, 0.74)**	0.52 (0.16, 1.35)	0.77 (0.06, 26.78)	Tisle-Chemo							
0.17 (0.005, 2.48)	0.17 (0.005, 2.67)	0.23 (0.01, 3.50)	0.41 (0.01, 5.95)	0.64 (0.01, 47.25)	0.80 (0.02, 14.94)	Nivo-Ipi						
**0.18 (0.10, 0.32)**	**0.19 (0.08, 0.42)**	**0.25 (0.13, 0.48)**	**0.45 (0.23, 0.89)**	0.69 (0.07, 22.08)	0.88 (0.30, 3.01)	1.11 (0.07, 40.24)	Ipi-Chemo					
**0.07 (0.003, 0.42)**	**0.07 (0.003, 0.48)**	**0.09 (0.004, 0.60)**	0.17 (0.01, 1.10)	0.25 (0.01, 12.28)	0.33 (0.01, 2.85)	0.39 (0.01, 21.89)	0.37 (0.01, 2.57)	Ave				
**0.06 (0.002, 0.32)**	**0.06 (0.002, 0.36)**	**0.08 (0.003, 0.45)**	**0.14 (0.01, 0.83)**	0.20 (0.004, 9.59)	0.27 (0.01, 2.17)	0.31 (0.01, 16.73)	0.30 (0.01, 1.94)	0.81 (0.02, 31.51)	Cemip-Chemo			
**0.02 (0.0009, 0.12)**	**0.02 (0.0009, 0.14)**	**0.03 (0.001, 0.17)**	**0.05 (0.002, 0.32)**	0.08 (0.002, 3.66)	**0.10 (0.004, 0.83)**	0.12 (0.002, 6.68)	**0.12 (0.005, 0.74)**	0.31 (0.01, 12.20)	0.39 (0.01, 15.23)	Sinti		
**0.01 (0.002, 0.05)**	**0.01 (0.002, 0.06)**	**0.02 (0.003, 0.07)**	**0.03 (0.005, 0.12)**	0.05 (0.003, 1.89)	**0.07 (0.01, 0.35)**	0.08 (0.003, 3.48)	**0.08 (0.01, 0.30)**	0.20 (0.01, 6.37)	0.25 (0.02, 7.85)	0.64 (0.05, 18.67)	Atezo	
**0.01 (0.002, 0.04)**	**0.01 (0.001, 0.05)**	**0.02 (0.002, 0.06)**	**0.03 (0.004, 0.10)**	0.04 (0.002, 1.57)	**0.05 (0.01, 0.28)**	0.06 (0.002, 2.81)	**0.06 (0.01, 0.24)**	0.16 (0.01, 5.01)	0.20 (0.01, 6.29)	0.51 (0.04, 15.44)	0.80 (0.08, 8.15)	Camre-Chemo

Immune-related investigations

(F) Respiratory, thoracic and mediastinal disorders

Rank 1	Rank 2	Rank 3	Rank 4	Rank 5	Rank 6	Rank 7	Rank 8	Rank 9	Rank 10	Rank 11	Rank 12	Rank 13	Rank 14	Rank 15	Rank 16	Rank 17	Rank 18	Rank 19	Rank 20	Rank 21
**Chemo**																				
0.75 (0.27, 2.10)	**Durva**																			
**0.48 (0.27, 0.80)**	0.64 (0.21, 1.89)	**Durva-Chemo**																		
**0.38 (0.18, 0.75)**	0.51 (0.14, 1.74)	0.80 (0.32, 1.94)	**Suge-Chemo**																	
**0.31 (0.16, 0.54)**	0.41 (0.12, 1.32)	0.64 (0.28, 1.43)	0.81 (0.32, 2.11)	**Pem-Chemo**																
**0.27 (0.13, 0.57)**	0.37 (0.12, 1.05)	0.57 (0.27, 1.22)	0.72 (0.26, 2.07)	0.89 (0.34, 2.36)	**Durva-Treme-**															
**0.25 (0.07, 0.66)**	0.32 (0.06, 1.38)	0.52 (0.13, 1.63)	0.64 (0.15, 2.29)	0.80 (0.20, 2.60)	0.90 (0.21, 3.20)	**Sinti-Chemo**														
**0.23 (0.10, 0.48)**	**0.31 (0.13, 0.66)**	0.48 (0.20, 1.12)	0.60 (0.21, 1.74)	0.75 (0.28, 1.96)	0.84 (0.38, 1.82)	0.94 (0.26, 4.00)	**Durva-Treme**													
0.25 (0.01, 1.73)	0.32 (0.01, 3.02)	0.51 (0.02, 3.95)	0.64 (0.02, 5.31)	0.80 (0.03, 6.26)	0.89 (0.03, 7.45)	1.00 (0.03, 10.39)	1.06 (0.03, 8.97)	**Cemip-Chemo**												
**0.19 (0.07, 0.43)**	**0.25 (0.06, 0.94)**	0.40 (0.13, 1.07)	0.50 (0.15, 1.54)	0.63 (0.20, 1.73)	0.70 (0.21, 2.14)	0.78 (0.19, 3.49)	0.84 (0.25, 2.61)	0.78 (0.09, 24.83)	**Tisle-Chemo**											
**0.19 (0.07, 0.43)**	**0.25 (0.06, 0.94)**	0.40 (0.13, 1.08)	0.50 (0.15, 1.53)	0.62 (0.20, 1.74)	0.70 (0.20, 2.14)	0.78 (0.19, 3.49)	0.83 (0.25, 2.62)	0.78 (0.09, 24.91)	0.99 (0.28, 3.53)	**Atezo**										
**0.18 (0.08, 0.36)**	**0.24 (0.06, 0.82)**	**0.38 (0.14, 0.92)**	0.47 (0.16, 1.33)	0.58 (0.22, 1.48)	0.66 (0.22, 1.85)	0.73 (0.20, 3.07)	0.78 (0.26, 2.26)	0.73 (0.09, 22.90)	0.93 (0.30, 3.08)	0.94 (0.30, 3.11)	**Atezo-Chemo**									
**0.14 (0.05, 0.31)**	**0.18 (0.04, 0.67)**	**0.29 (0.10, 0.77)**	0.36 (0.11, 1.10)	0.45 (0.14, 1.24)	0.50 (0.15, 1.53)	0.56 (0.14, 2.52)	0.60 (0.18, 1.88)	0.56 (0.06, 17.79)	0.72 (0.20, 2.53)	0.72 (0.20, 2.57)	0.77 (0.23, 2.41)	**Nivo**								
**0.13 (0.04, 0.33)**	**0.17 (0.04, 0.69)**	**0.27 (0.08, 0.80)**	0.34 (0.10, 1.14)	0.42 (0.12, 1.30)	0.47 (0.13, 1.59)	0.53 (0.12, 2.56)	0.57 (0.15, 1.93)	0.53 (0.06, 17.18)	0.68 (0.18, 2.60)	0.68 (0.18, 2.62)	0.73 (0.20, 2.50)	0.94 (0.59, 1.50)	**Nivo-Ipi**							
**0.13 (0.07, 0.22)**	**0.17 (0.05, 0.54)**	**0.27 (0.12, 0.58)**	**0.34 (0.14, 0.86)**	**0.42 (0.18, 0.95)**	0.47 (0.18, 1.20)	0.52 (0.16, 2.06)	0.56 (0.22, 1.47)	0.52 (0.07, 15.84)	0.67 (0.24, 2.03)	0.67 (0.24, 2.06)	0.72 (0.29, 1.90)	0.93 (0.34, 2.84)	0.99 (0.33, 3.29)	**Pem**						
**0.09 (0.003, 0.60)**	0.12 (0.004, 1.06)	0.19 (0.01, 1.38)	0.24 (0.01, 1.88)	0.30 (0.01, 2.20)	0.33 (0.01, 2.60)	0.37 (0.01, 3.64)	0.40 (0.01, 3.15)	0.37 (0.01, 17.20)	0.47 (0.02, 4.05)	0.48 (0.02, 4.07)	0.51 (0.02, 4.02)	0.66 (0.02, 5.69)	0.70 (0.02, 6.37)	0.71 (0.02, 5.19)	**Cemip**					
**0.05 (0.002, 0.31)**	**0.07 (0.002, 0.56)**	**0.11 (0.004, 0.73)**	**0.14 (0.01, 0.97)**	0.17 (0.01, 1.15)	0.19 (0.01, 1.37)	0.21 (0.01, 1.94)	0.23 (0.01, 1.66)	0.21 (0.01, 9.31)	0.27 (0.01, 2.13)	0.28 (0.01, 2.16)	0.29 (0.01, 2.13)	0.38 (0.01, 2.99)	0.40 (0.01, 3.37)	0.41 (0.02, 2.71)	0.57 (0.01, 24.36)	**Ave**				
**0.04 (0.001, 0.25)**	**0.06 (0.002, 0.45)**	**0.09 (0.004, 0.58)**	**0.12 (0.004, 0.78)**	**0.14 (0.01, 0.92)**	0.16 (0.01, 1.09)	0.18 (0.01, 1.57)	0.19 (0.01, 1.31)	0.18 (0.004, 7.75)	0.23 (0.01, 1.70)	0.23 (0.01, 1.73)	0.25 (0.01, 1.69)	0.32 (0.01, 2.38)	0.34 (0.01, 2.67)	0.35 (0.01, 2.17)	0.48 (0.01, 19.39)	0.84 (0.02, 32.56)	**Tisle**			
**0.05 (0.02, 0.11)**	**0.06 (0.02, 0.24)**	**0.10 (0.04, 0.27)**	**0.13 (0.04, 0.39)**	**0.16 (0.06, 0.45)**	**0.18 (0.06, 0.55)**	**0.20 (0.05, 0.89)**	**0.21 (0.07, 0.67)**	0.20 (0.02, 6.26)	**0.25 (0.08, 0.90)**	**0.26 (0.08, 0.91)**	**0.27 (0.09, 0.86)**	0.35 (0.11, 1.26)	0.37 (0.10, 1.45)	**0.38 (0.20, 0.70)**	0.54 (0.07, 16.14)	0.93 (0.12, 25.73)	1.10 (0.15, 29.17)	**Pem-Ipi**		
**0.03 (0.001, 0.19)**	**0.04 (0.001, 0.34)**	**0.07 (0.002, 0.43)**	**0.08 (0.003, 0.59)**	**0.10 (0.004, 0.69)**	**0.12 (0.005, 0.82)**	0.13 (0.004, 1.17)	**0.14 (0.005, 0.99)**	0.13 (0.003, 5.87)	0.17 (0.01, 1.28)	0.17 (0.01, 1.29)	0.18 (0.01, 1.27)	0.23 (0.01, 1.78)	0.25 (0.01, 2.01)	0.25 (0.01, 1.63)	0.35 (0.01, 14.79)	0.61 (0.01, 23.66)	0.73 (0.02, 27.30)	0.66 (0.02, 4.85)	**Nivo-Ipi-Chemo**	
**0.04 (0.01, 0.12)**	**0.05 (0.01, 0.24)**	**0.07 (0.01, 0.30)**	**0.09 (0.01, 0.41)**	**0.12 (0.02, 0.47)**	**0.13 (0.02, 0.57)**	**0.14 (0.02, 0.87)**	**0.15 (0.02, 0.69)**	0.14 (0.01, 5.19)	**0.18 (0.02, 0.90)**	**0.19 (0.02, 0.91)**	**0.20 (0.03, 0.89)**	0.26 (0.03, 1.26)	0.27 (0.03, 1.44)	0.28 (0.04, 1.12)	0.38 (0.03, 13.15)	0.67 (0.05, 21.10)	0.80 (0.06, 23.31)	0.72 (0.09, 3.44)	1.10 (0.08, 36.23)	**Camre-Chemo**

Immune-related respiratory, thoracic and mediastinal disorders

(G) Skin and subcutaneous tissue disorders.

Rank 1	Rank 2	Rank 3	Rank 4	Rank 5	Rank 6	Rank 7	Rank 8	Rank 9	Rank 10	Rank 11	Rank 12	Rank 13	Rank 14	Rank 15	Rank 16	Rank 17	Rank 18	Rank 19	Rank 20	Rank 21	Rank 22
**Chemo**																					
0.57 (0.28, 1.09)	**Tisle-Chemo**																				
0.57 (0.23, 1.29)	0.99 (0.32, 2.96)	**Pem-Chemo**																			
0.52 (0.18, 1.60)	0.93 (0.26, 3.46)	0.94 (0.24, 3.92)	**Durva**																		
**0.48 (0.27, 0.79)**	0.83 (0.36, 2.01)	0.84 (0.31, 2.40)	0.90 (0.26, 3.01)	**Sinti-Chemo**																	
**0.47 (0.34, 0.63)**	0.82 (0.40, 1.78)	0.83 (0.34, 2.18)	0.89 (0.28, 2.76)	0.98 (0.54, 1.84)	**Ipi-Chemo**																
**0.43 (0.18, 0.98)**	0.76 (0.25, 2.25)	0.77 (0.23, 2.62)	0.82 (0.25, 2.57)	0.91 (0.33, 2.43)	0.93 (0.36, 2.22)	**Durva-Chemo**															
**0.42 (0.29, 0.61)**	0.74 (0.35, 1.65)	0.74 (0.30, 2.00)	0.80 (0.25, 2.53)	0.88 (0.47, 1.70)	0.90 (0.55, 1.46)	0.97 (0.39, 2.52)	**Atezo-Chemo**														
**0.37 (0.28, 0.48)**	0.65 (0.32, 1.40)	0.65 (0.27, 1.70)	0.70 (0.22, 2.15)	0.77 (0.43, 1.42)	0.79 (0.52, 1.19)	0.85 (0.36, 2.13)	0.87 (0.55, 1.40)	**Nivo**													
0.18 (0.01, 1.18)	0.32 (0.01, 2.39)	0.32 (0.01, 2.64)	0.34 (0.01, 3.10)	0.38 (0.01, 2.73)	0.39 (0.01, 2.62)	0.41 (0.01, 3.39)	0.43 (0.01, 2.93)	0.50 (0.02, 3.29)	**Cemip-Chemo**												
**0.20 (0.13, 0.29)**	**0.34 (0.16, 0.78)**	**0.35 (0.14, 0.94)**	0.37 (0.11, 1.19)	**0.41 (0.21, 0.81)**	**0.42 (0.25, 0.70)**	0.45 (0.18, 1.19)	**0.47 (0.27, 0.81)**	**0.53 (0.40, 0.71)**	1.08 (0.16, 31.25)	**Nivo-Ipi**											
**0.18 (0.08, 0.37)**	**0.31 (0.11, 0.86)**	0.31 (0.10, 1.01)	**0.34 (0.13, 0.77)**	**0.37 (0.14, 0.93)**	**0.38 (0.16, 0.84)**	**0.41 (0.18, 0.90)**	**0.42 (0.17, 0.96)**	0.48 (0.20, 1.05)	0.98 (0.12, 29.50)	0.90 (0.36, 2.09)	**Durva-Treme**										
0.15 (0.01, 1.13)	0.27 (0.01, 2.25)	0.27 (0.01, 2.48)	0.28 (0.01, 2.90)	0.32 (0.01, 2.58)	0.33 (0.01, 2.49)	0.35 (0.01, 3.17)	0.36 (0.01, 2.80)	0.42 (0.01, 3.13)	0.85 (0.02, 38.94)	0.78 (0.03, 6.04)	0.86 (0.03, 7.60)	**Cemip**									
**0.16 (0.05, 0.43)**	**0.29 (0.07, 0.96)**	0.29 (0.06, 1.10)	0.30 (0.06, 1.35)	0.34 (0.09, 1.05)	**0.35 (0.10, 0.97)**	0.37 (0.08, 1.40)	0.39 (0.10, 1.11)	0.44 (0.12, 1.22)	0.89 (0.09, 28.47)	0.83 (0.22, 2.39)	0.92 (0.22, 3.30)	1.06 (0.10, 36.27)	**Pem**								
**0.15 (0.10, 0.22)**	**0.27 (0.12, 0.60)**	**0.27 (0.11, 0.73)**	**0.29 (0.09, 0.92)**	**0.32 (0.17, 0.62)**	**0.33 (0.20, 0.53)**	**0.35 (0.14, 0.92)**	**0.36 (0.21, 0.62)**	**0.41 (0.25, 0.66)**	0.84 (0.12, 24.28)	0.78 (0.44, 1.36)	0.86 (0.37, 2.13)	1.00 (0.13, 30.82)	0.94 (0.33, 3.48)	**Atezo**							
**0.14 (0.06, 0.28)**	**0.24 (0.09, 0.66)**	**0.24 (0.08, 0.77)**	**0.26 (0.09, 0.67)**	**0.29 (0.11, 0.71)**	**0.30 (0.13, 0.64)**	**0.32 (0.16, 0.63)**	**0.33 (0.14, 0.73)**	**0.37 (0.16, 0.80)**	0.76 (0.10, 22.67)	0.70 (0.29, 1.58)	0.78 (0.49, 1.23)	0.91 (0.10, 28.95)	0.85 (0.24, 3.56)	0.90 (0.38, 2.03)	**Durva-Treme-Chemo**						
**0.09 (0.01, 0.31)**	**0.15 (0.02, 0.66)**	**0.15 (0.02, 0.74)**	**0.16 (0.02, 0.89)**	**0.18 (0.02, 0.73)**	**0.19 (0.03, 0.69)**	**0.20 (0.02, 0.95)**	**0.21 (0.03, 0.77)**	**0.24 (0.03, 0.86)**	0.47 (0.03, 16.27)	0.44 (0.06, 1.68)	0.49 (0.06, 2.24)	0.56 (0.04, 20.97)	0.53 (0.06, 3.18)	0.57 (0.08, 2.17)	0.62 (0.08, 2.82)	**Suge-Chemo**					
**0.08 (0.02, 0.35)**	**0.15 (0.03, 0.72)**	**0.15 (0.02, 0.80)**	**0.16 (0.02, 0.96)**	**0.18 (0.03, 0.81)**	**0.18 (0.03, 0.77)**	0.19 (0.03, 1.03)	**0.20 (0.04, 0.86)**	**0.23 (0.04, 0.97)**	0.47 (0.04, 16.75)	0.43 (0.08, 1.87)	0.47 (0.08, 2.45)	0.56 (0.04, 20.99)	0.52 (0.17, 1.42)	0.55 (0.10, 2.41)	0.61 (0.10, 3.09)	0.99 (0.12, 10.49)	**Pem-Ipi**				
**0.03 (0.001, 0.16)**	**0.05 (0.002, 0.33)**	**0.05 (0.002, 0.37)**	**0.05 (0.001, 0.43)**	**0.06 (0.002, 0.37)**	**0.06 (0.002, 0.36)**	**0.06 (0.002, 0.47)**	**0.07 (0.002, 0.40)**	**0.08 (0.003, 0.45)**	0.15 (0.004, 6.42)	**0.15 (0.01, 0.87)**	0.16 (0.01, 1.12)	0.18 (0.004, 7.96)	0.17 (0.01, 1.52)	0.19 (0.01, 1.12)	0.21 (0.01, 1.42)	0.33 (0.01, 4.47)	0.33 (0.01, 3.84)	**Ave**			
**0.03 (0.004, 0.10)**	**0.05 (0.01, 0.22)**	**0.05 (0.01, 0.25)**	**0.06 (0.01, 0.30)**	**0.06 (0.01, 0.25)**	**0.06 (0.01, 0.23)**	**0.07 (0.01, 0.32)**	**0.07 (0.01, 0.26)**	**0.08 (0.01, 0.29)**	0.16 (0.01, 5.59)	**0.15 (0.02, 0.57)**	**0.17 (0.02, 0.75)**	0.19 (0.01, 7.09)	0.18 (0.02, 1.09)	**0.20 (0.03, 0.73)**	**0.22 (0.03, 0.96)**	0.34 (0.03, 3.39)	0.35 (0.03, 2.83)	1.04 (0.08, 36.07)	**Camre-Chemo**		
**0.02 (0.0006, 0.09)**	**0.03 (0.001, 0.19)**	**0.03 (0.001, 0.21)**	**0.03 (0.001, 0.25)**	**0.03 (0.001, 0.21)**	**0.03 (0.001, 0.20)**	**0.04 (0.001, 0.27)**	**0.04 (0.001, 0.23)**	**0.04 (0.002, 0.26)**	0.09 (0.002, 3.74)	**0.08 (0.003, 0.50)**	**0.09 (0.003, 0.64)**	0.10 (0.002, 4.66)	**0.10 (0.003, 0.86)**	**0.11 (0.004, 0.64)**	**0.12 (0.004, 0.81)**	0.19 (0.01, 2.52)	0.19 (0.01, 2.18)	0.57 (0.01, 23.93)	0.55 (0.02, 7.40)	**Sinti**	
**0.01 (0.0004, 0.05)**	**0.02 (0.0006, 0.11)**	**0.02 (0.0006, 0.12)**	**0.02 (0.0006, 0.14)**	**0.02 (0.001, 0.12)**	**0.02 (0.001, 0.12)**	**0.02 (0.001, 0.16)**	**0.02 (0.001, 0.13)**	**0.03 (0.001, 0.15)**	0.05 (0.001, 2.18)	**0.05 (0.002, 0.29)**	**0.05 (0.002, 0.37)**	0.06 (0.001, 2.72)	**0.06 (0.002, 0.50)**	**0.06 (0.002, 0.37)**	**0.07 (0.003, 0.47)**	0.11 (0.003, 1.48)	0.11 (0.003, 1.28)	0.34 (0.01, 13.99)	0.32 (0.01, 4.25)	0.59 (0.01, 23.52)	**Nivo-Ipi-Chemo**

Immune-related skin and subcutaneous tissue disorders

Supplementary Figure S5. Odds ratio (95% CrI) of system organ classes specific immune-related adverse events associated with each treatment regimen. (A) Endocrine disorders; (B) Gastrointestinal disorders; (C) Hepatobiliary disorders; (D) Injury, poisoning and procedural complications; (E) Investigations; (F) Respiratory, thoracic and mediastinal disorders; (G) Skin and subcutaneous tissue disorders. Atezo: atezolizumab; Ave: avelumab; Beva: bevacizumab; Camre: camrelizumab; Cemip: cemiplimab; Chemo, chemotherapy; Darat: daratumumab; Dostra, dostarlimab; Durva: durvalumab; Ipi: ipilimumab; Nivo: nivolumab; NSCLC: non-small cell lung cancer; Pem: pembrolizumab; Sint: sintilimab; Sugema: sugemalimab; Tisle: tislelizumab; Treme: tremelimumab.

(A) Blood and lymphatic system disorders

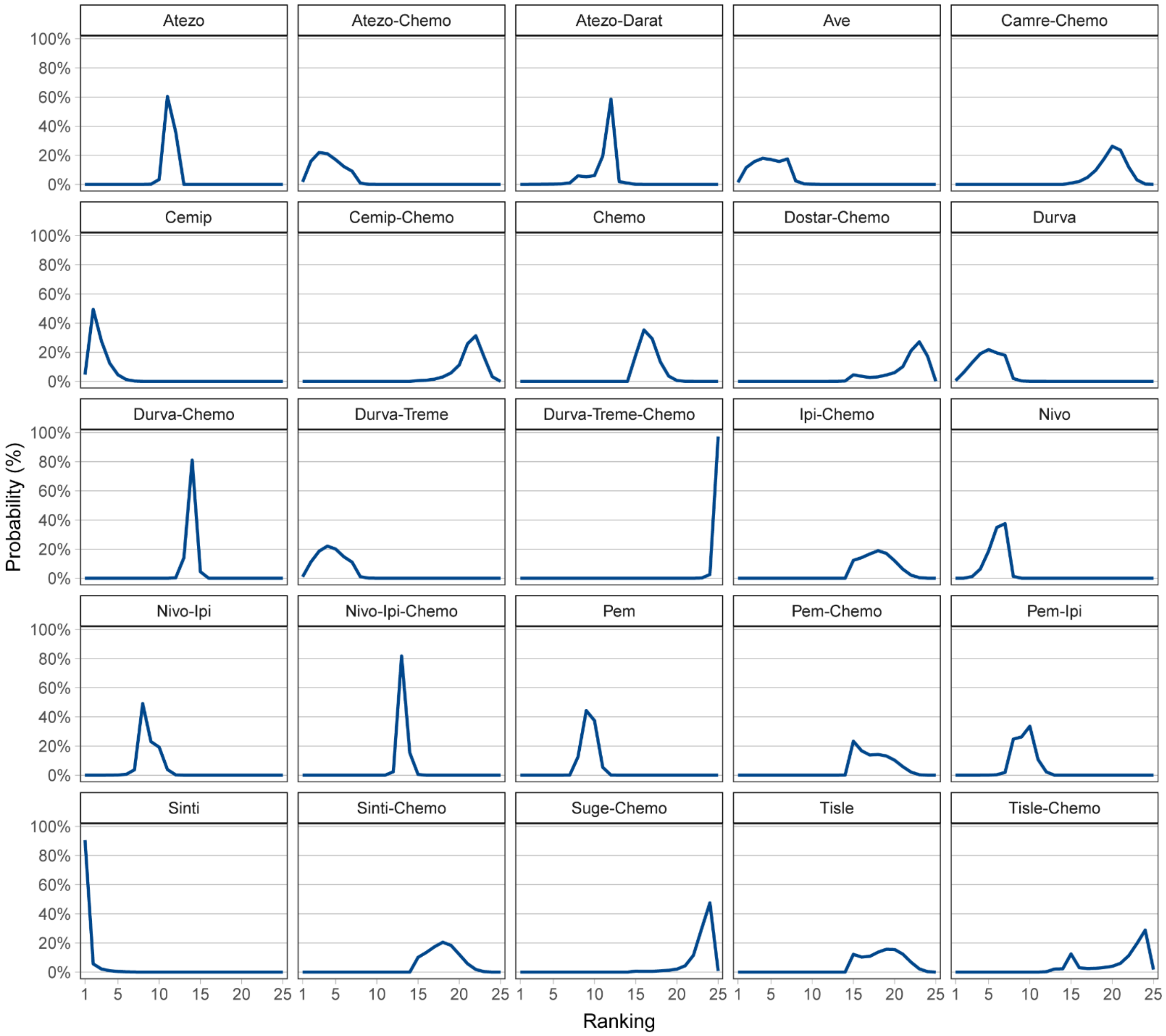

(B) Endocrine disorders

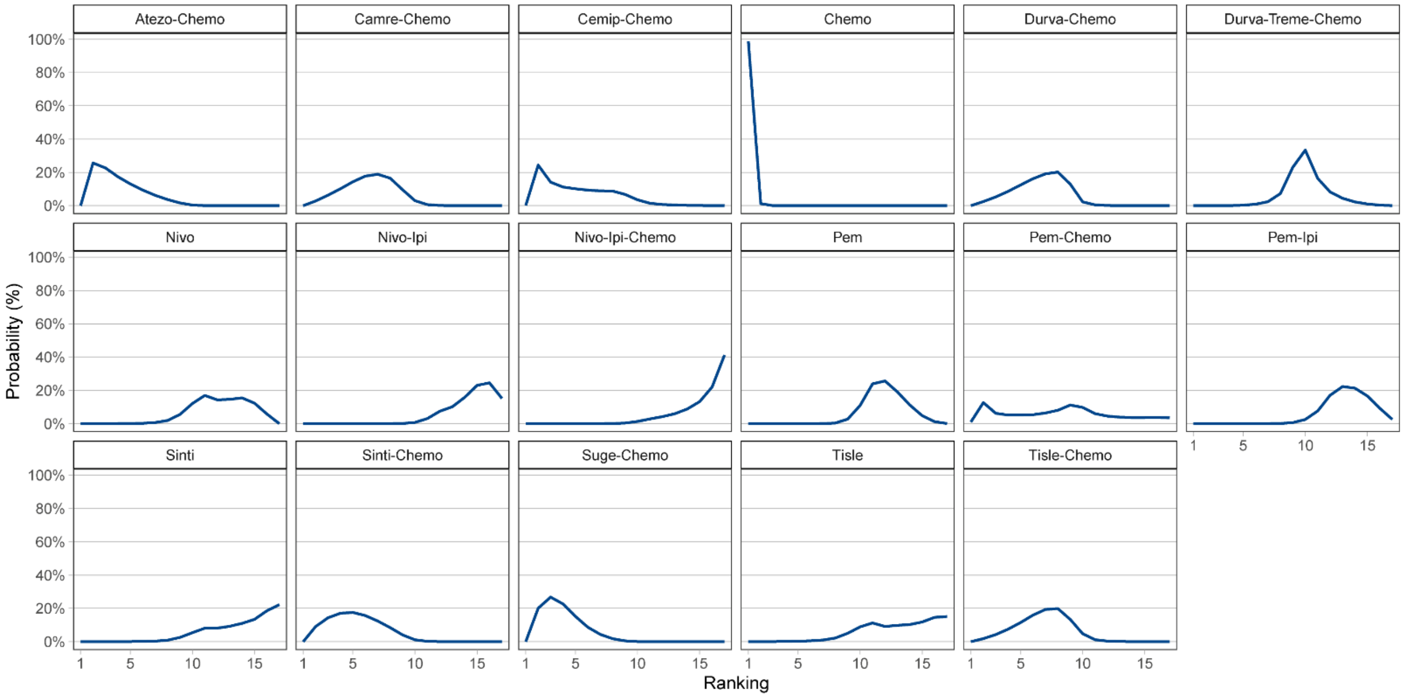

(C) Gastrointestinal disorders

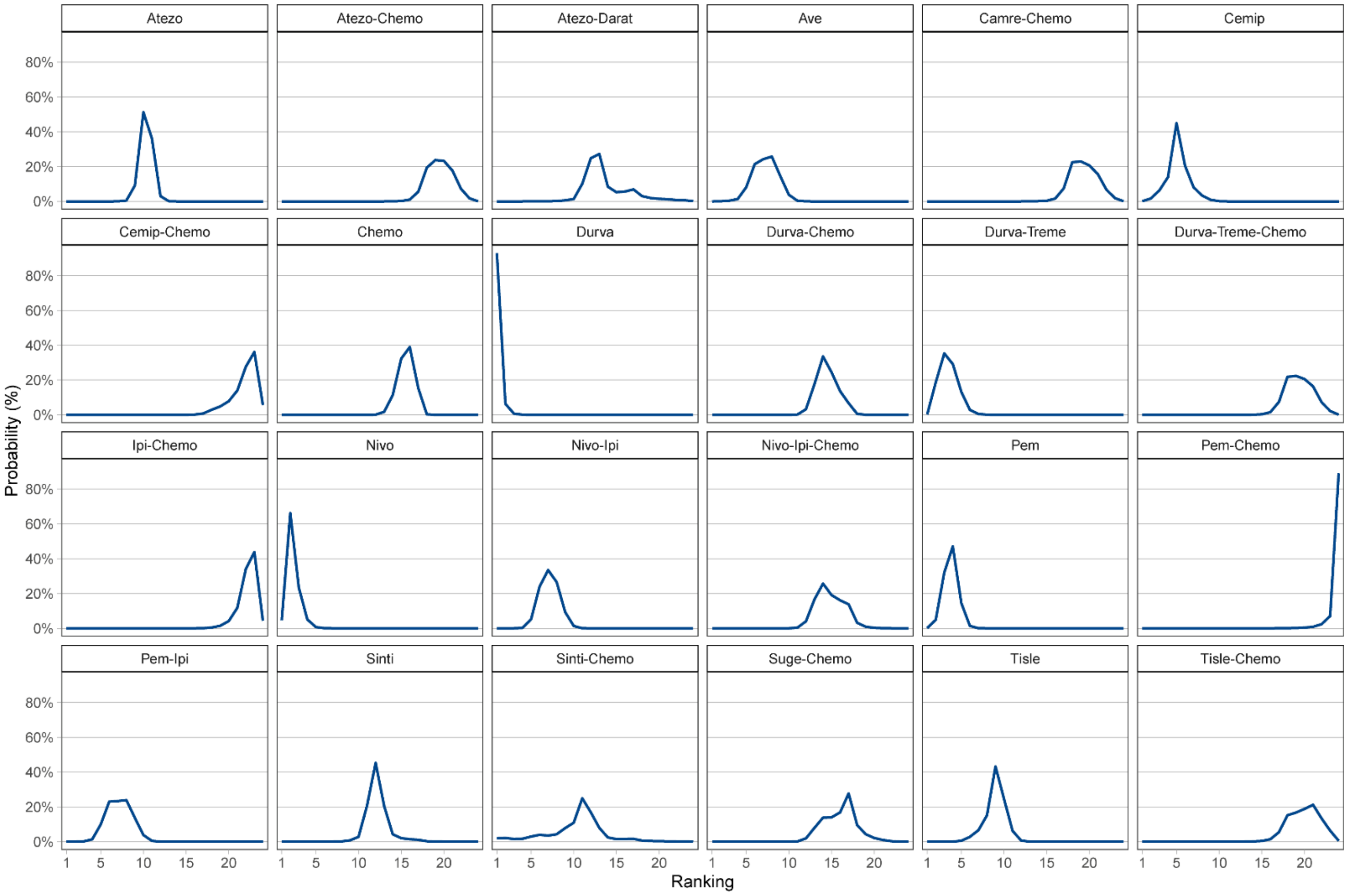

(D) General disorders and administration site conditions

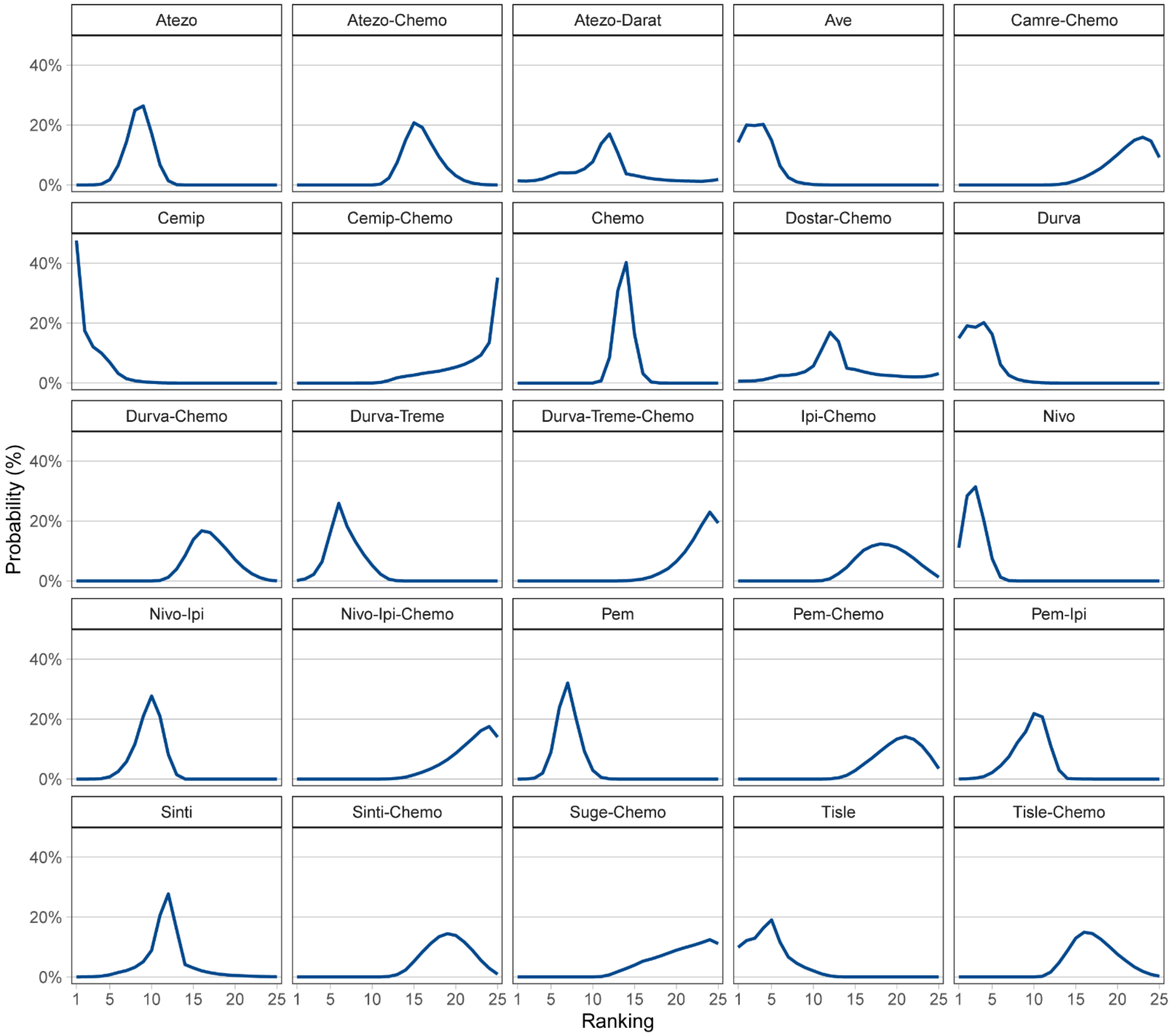

(E) Investigations

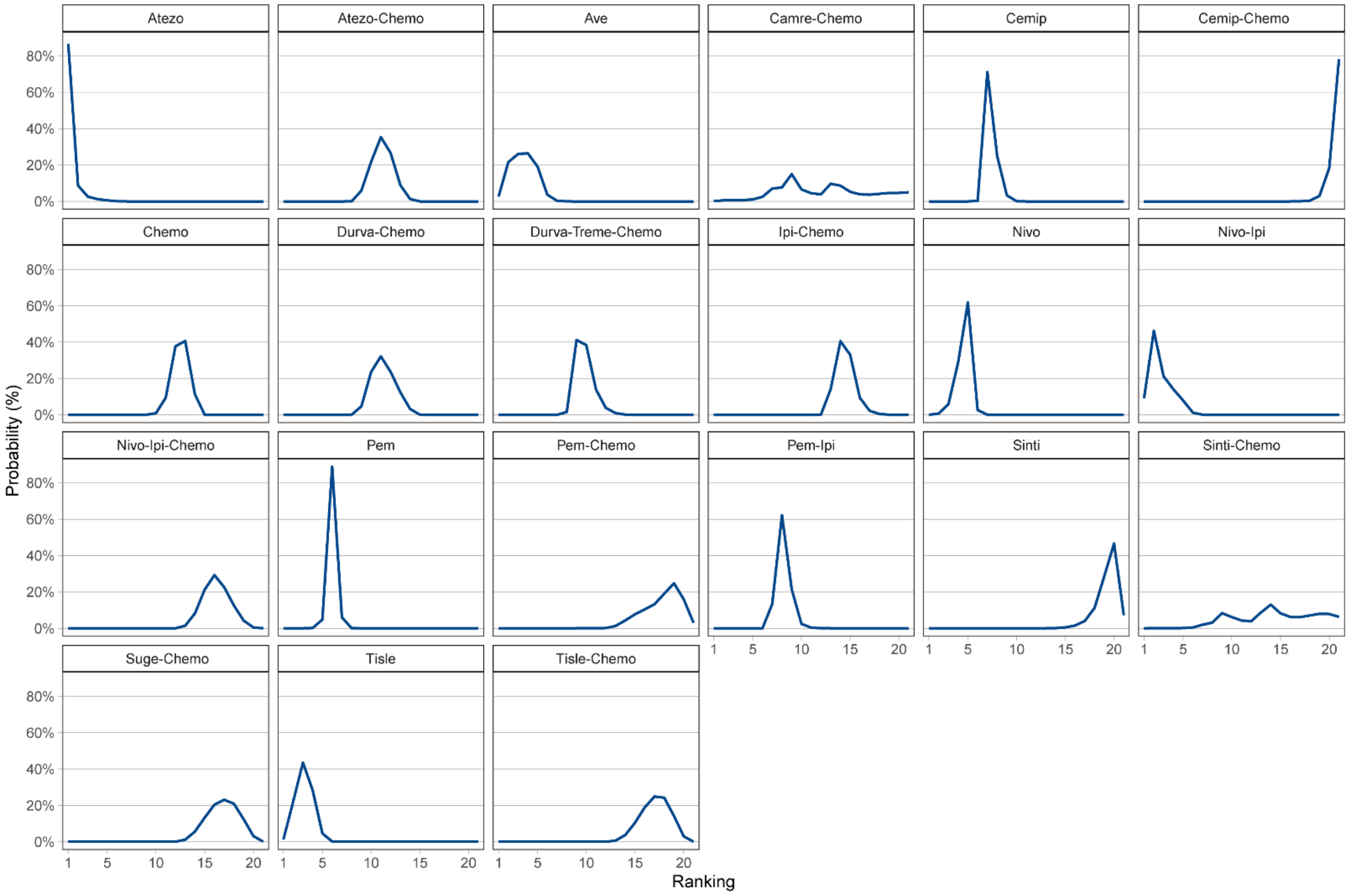

(F) Metabolism and nutrition disorders

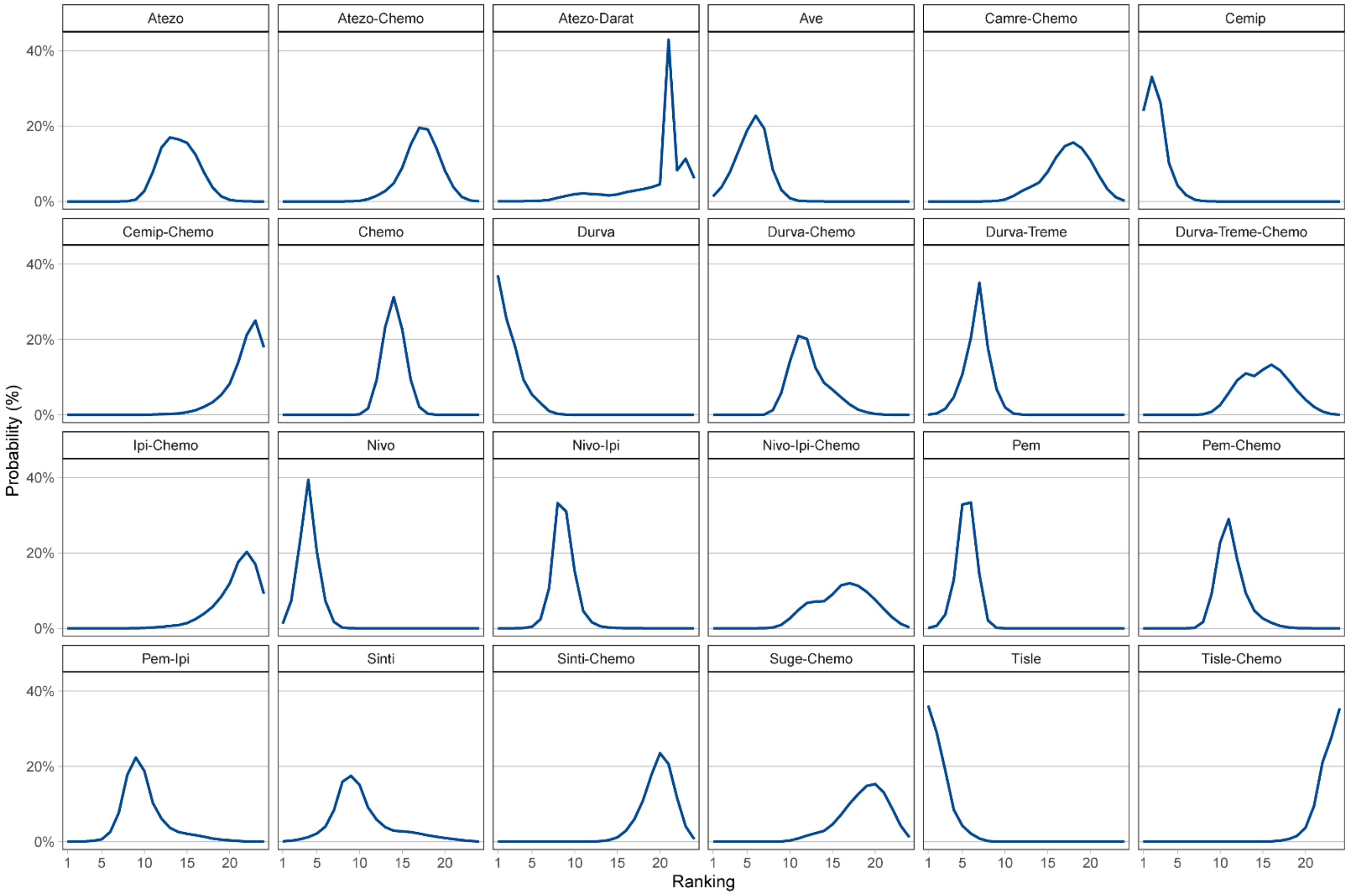

(G) Musculoskeletal and connective tissue disorders

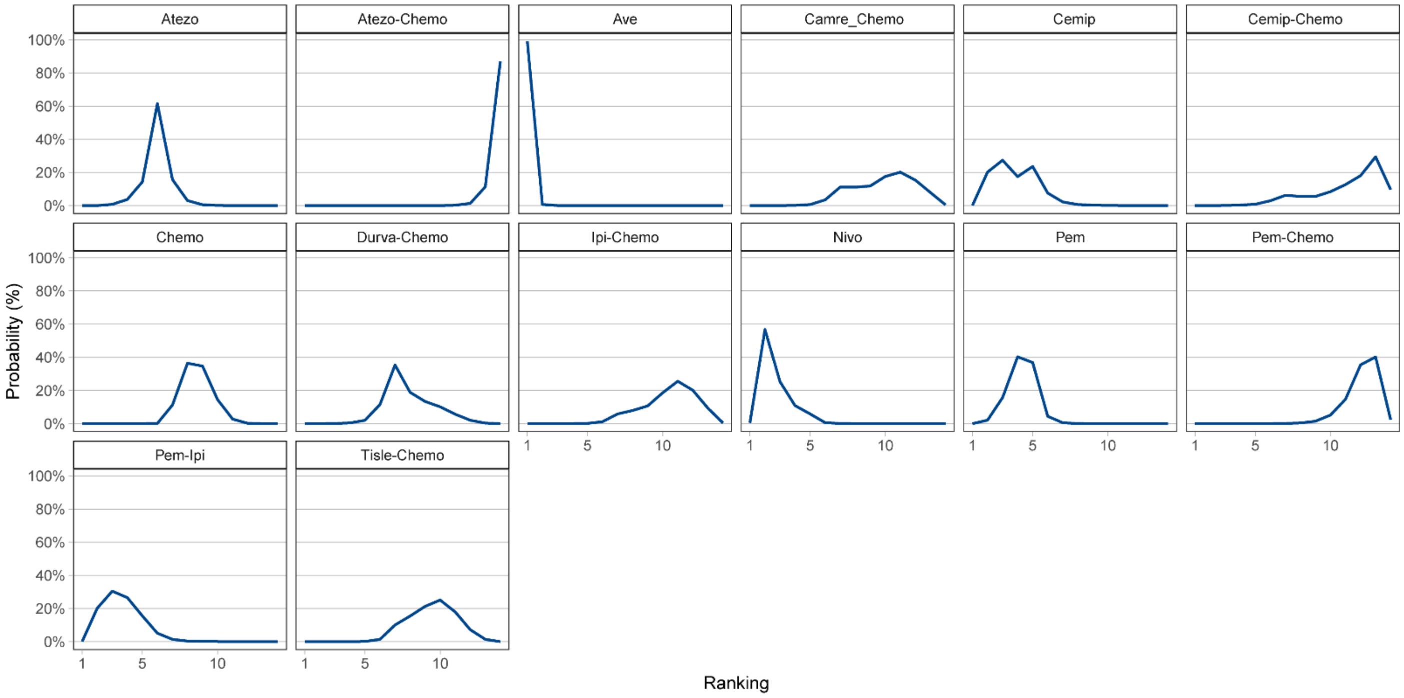

(H) Nervous system disorders

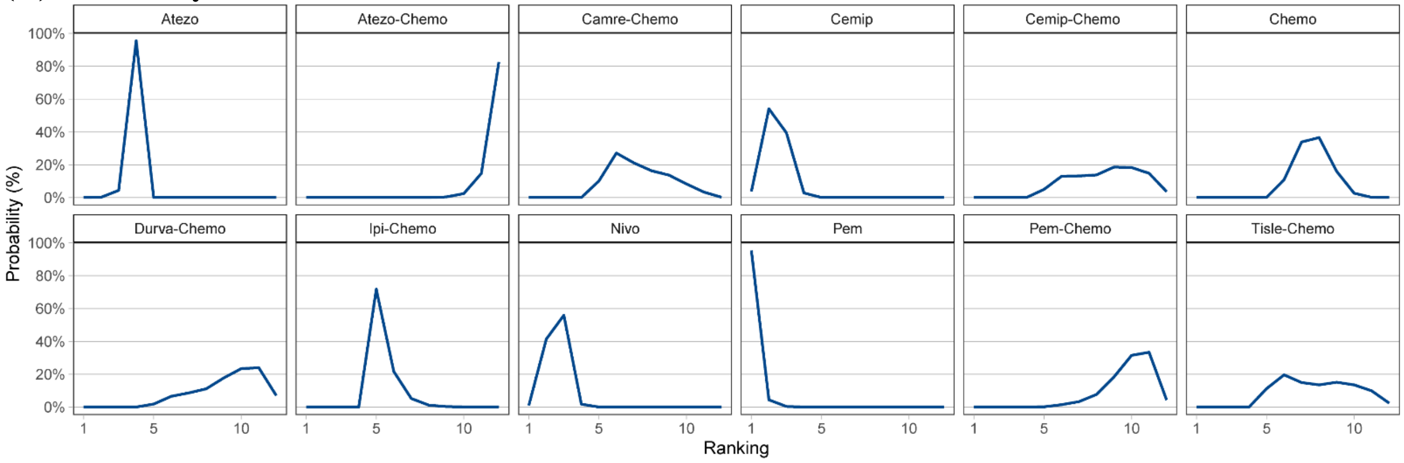

(I) Respiratory, thoracic and mediastinal disorders

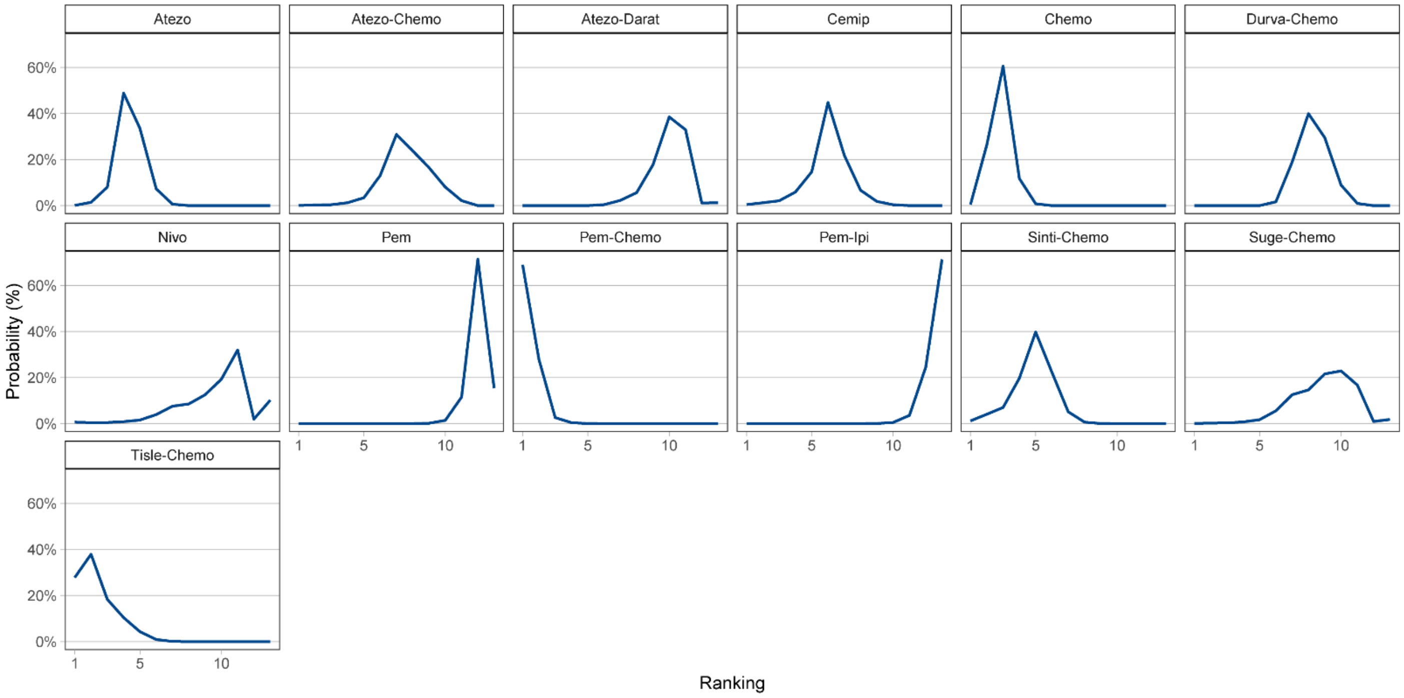

(J) Skin and subcutaneous tissue disorders

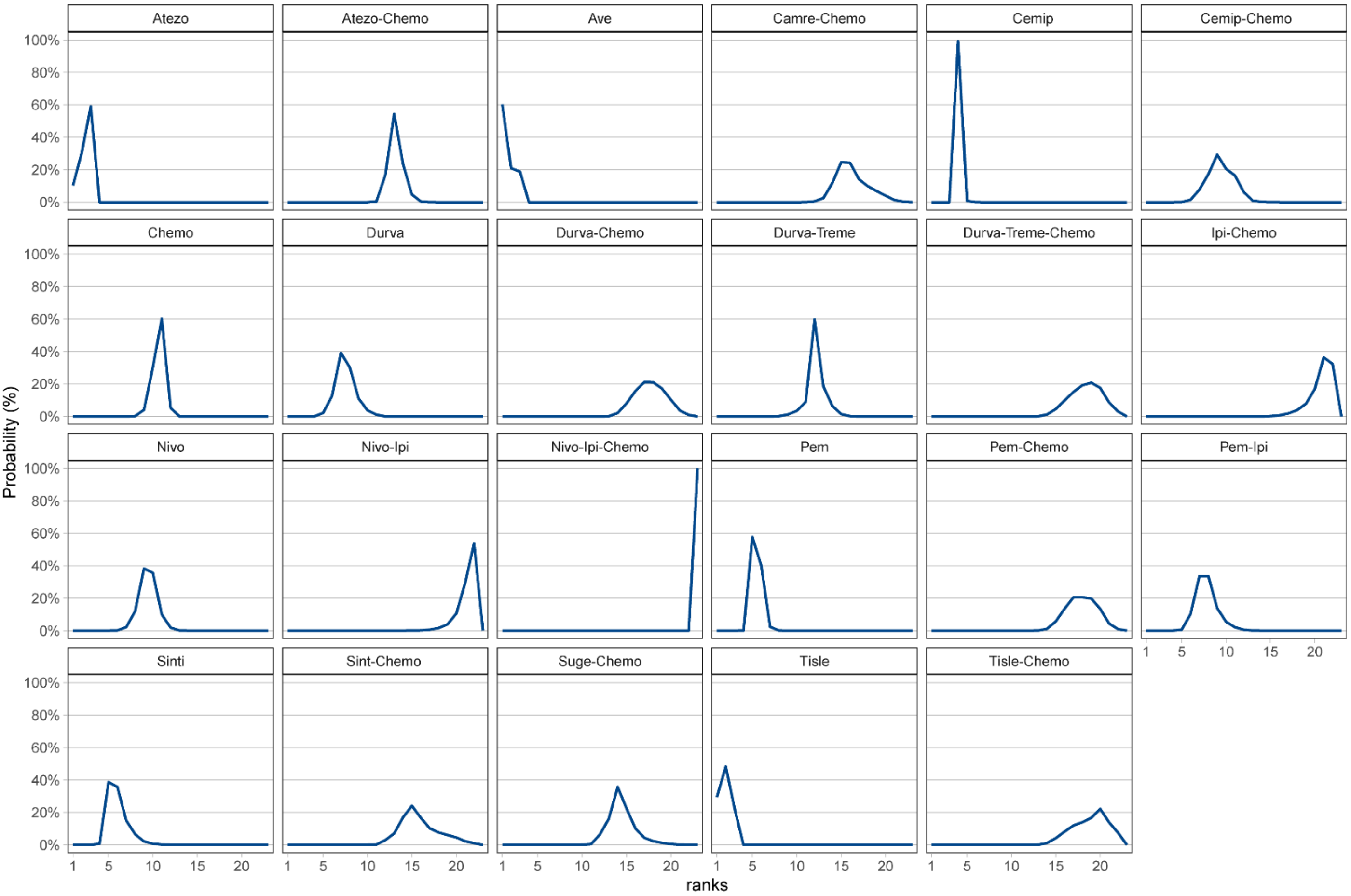

(K) Infections and infestations

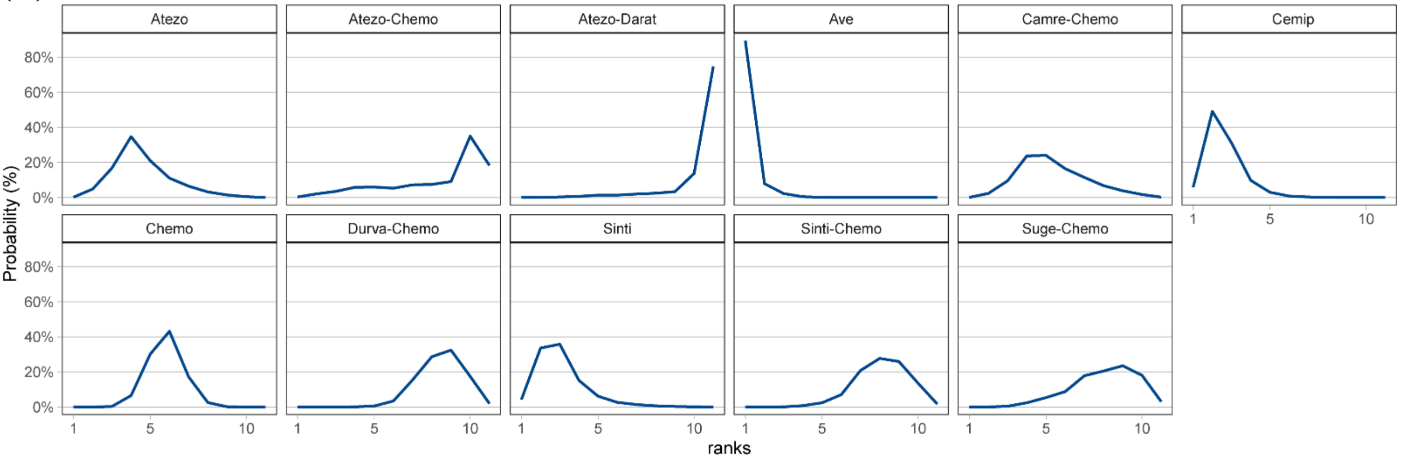

Supplementary Figure S6. Ranking of the probability of being the best treatment regimen in system organ classes specific treatment-related adverse events. (A) Blood and lymphatic system disorders; (B) Endocrine disorders; (C) Gastrointestinal disorders; (D) General disorders and administration site conditions; (E) Investigations; (F) Metabolism and nutrition disorders; (G) Musculoskeletal and connective tissue disorders; (H) Nervous system disorders; (I) Respiratory, thoracic and mediastinal disorders; (J) Skin and subcutaneous tissue disorders; (K) Infections and infestations. Atezo: atezolizumab; Ave: avelumab; Beva: bevacizumab; Camre: camrelizumab; Cemip: cemiplimab; Chemo, chemotherapy; Darat: daratumumab; Dostra, dostarlimab; Durva: durvalumab; Ipi: ipilimumab; Nivo: nivolumab; NSCLC: non-small cell lung cancer; Pem: pembrolizumab; Sint: sintilimab; Sugema: sugemalimab; Tisle: tislelizumab; Treme: tremelimumab.

(A) Endocrine disorders

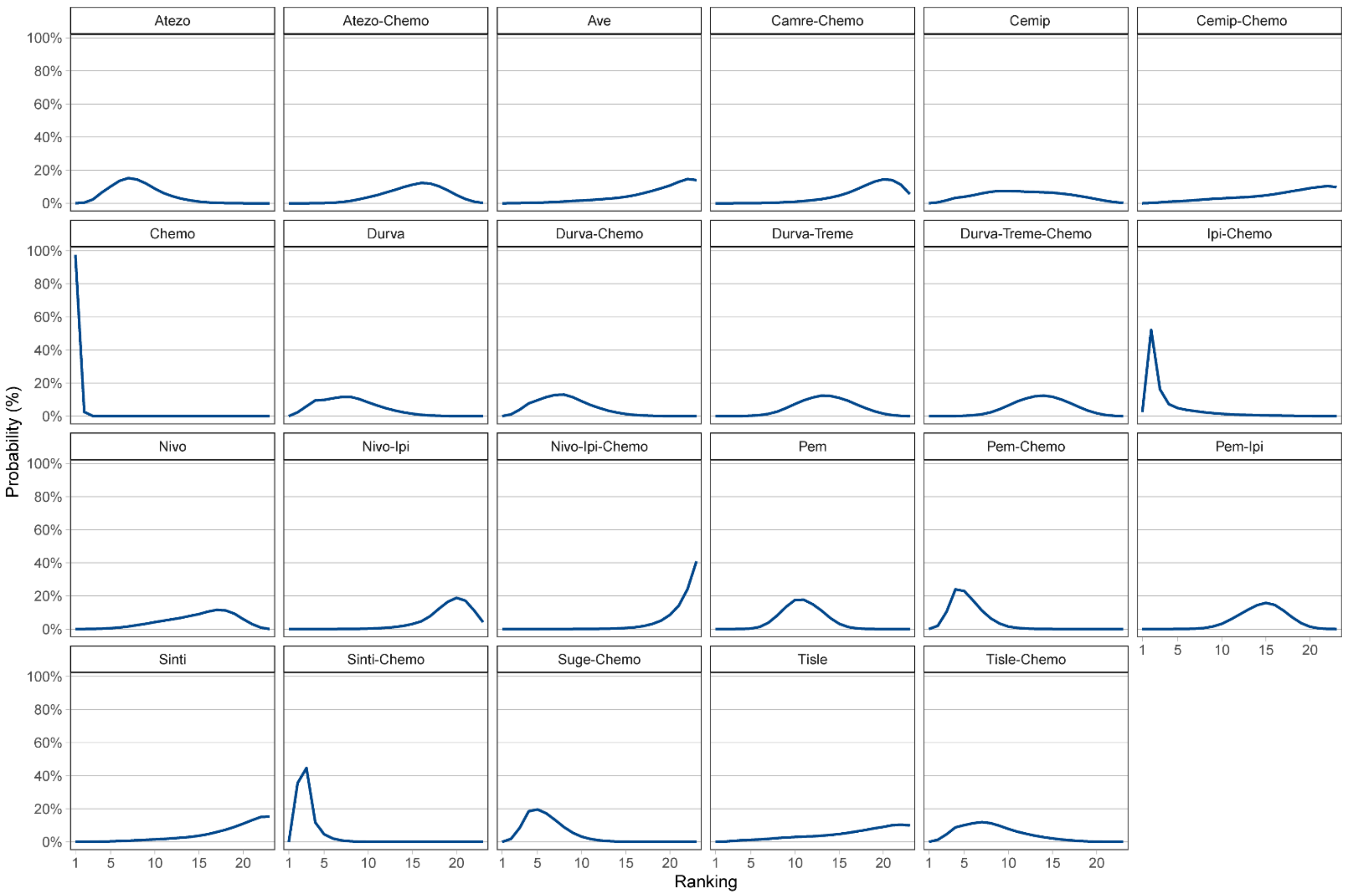

(B) Gastrointestinal disorders

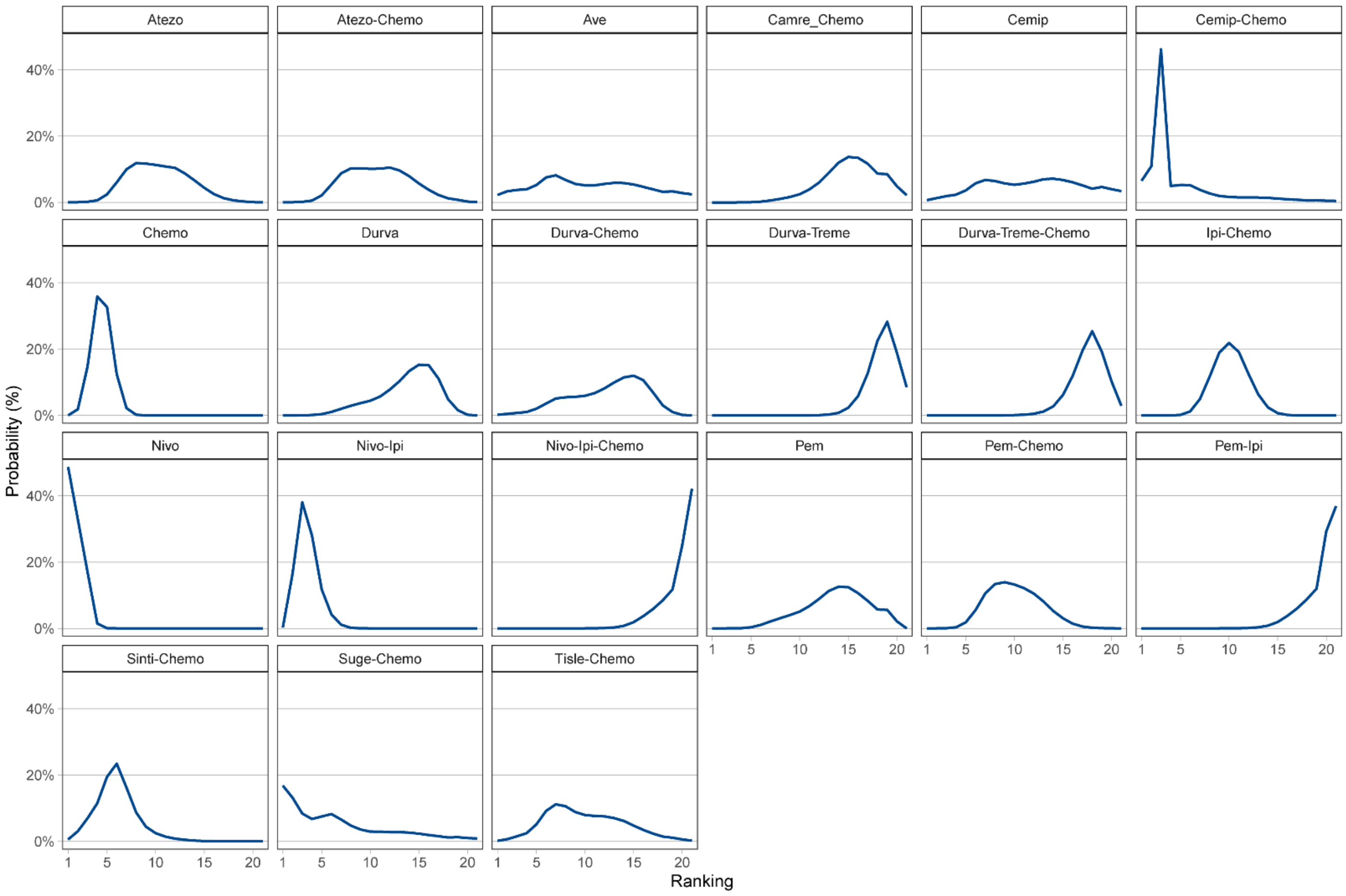

(C) Hepatobiliary disorders

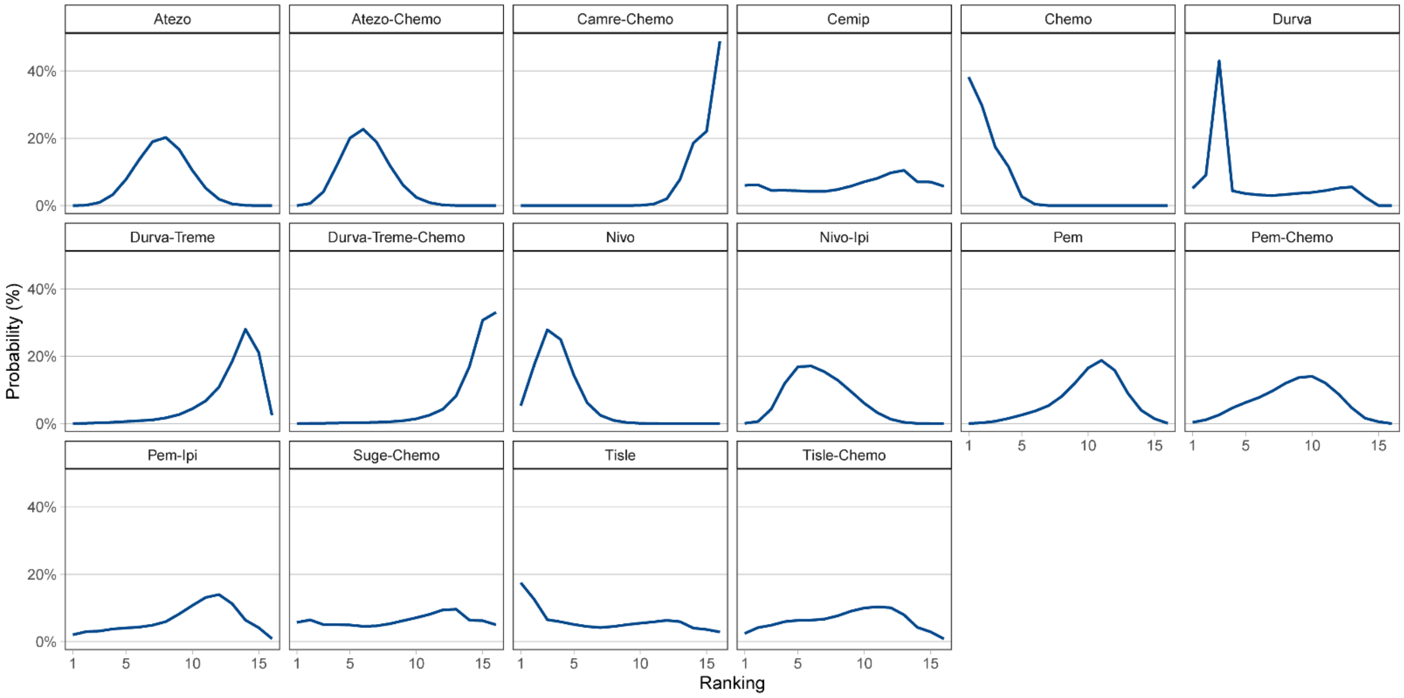

(D) Injury, poisoning and procedural complications

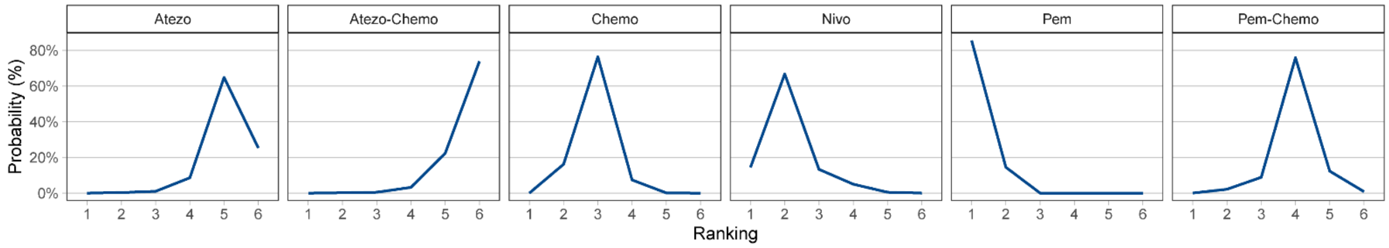

(E) Investigations

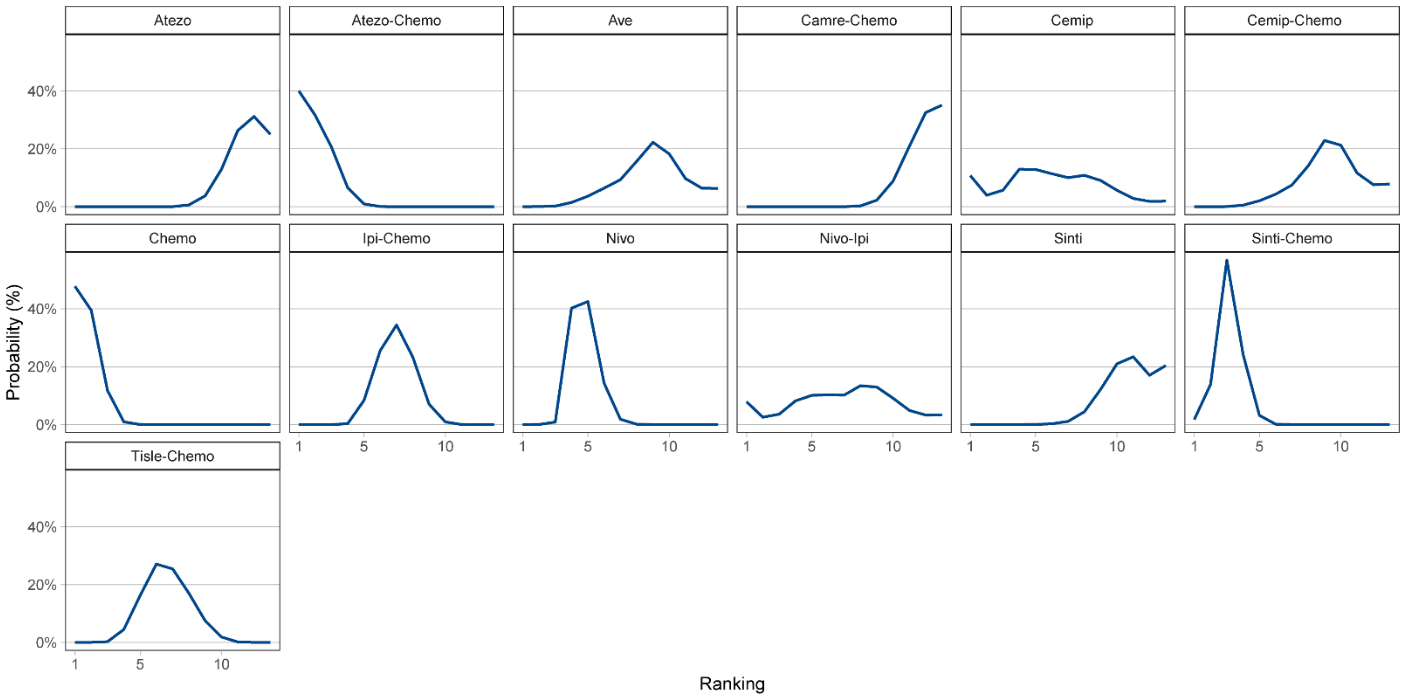

(F) Respiratory, thoracic and mediastinal disorders

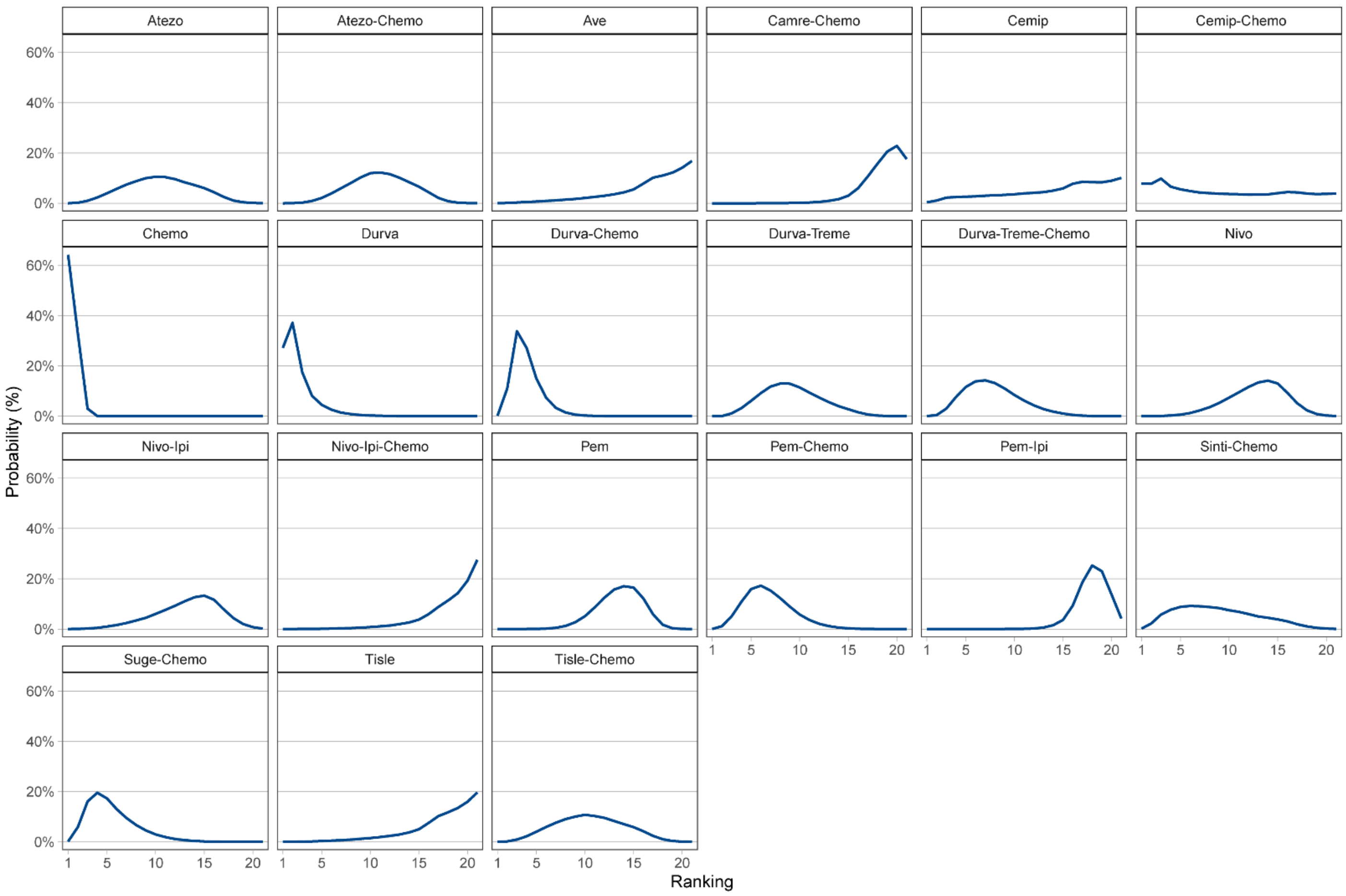

(G) Skin and subcutaneous tissue disorders

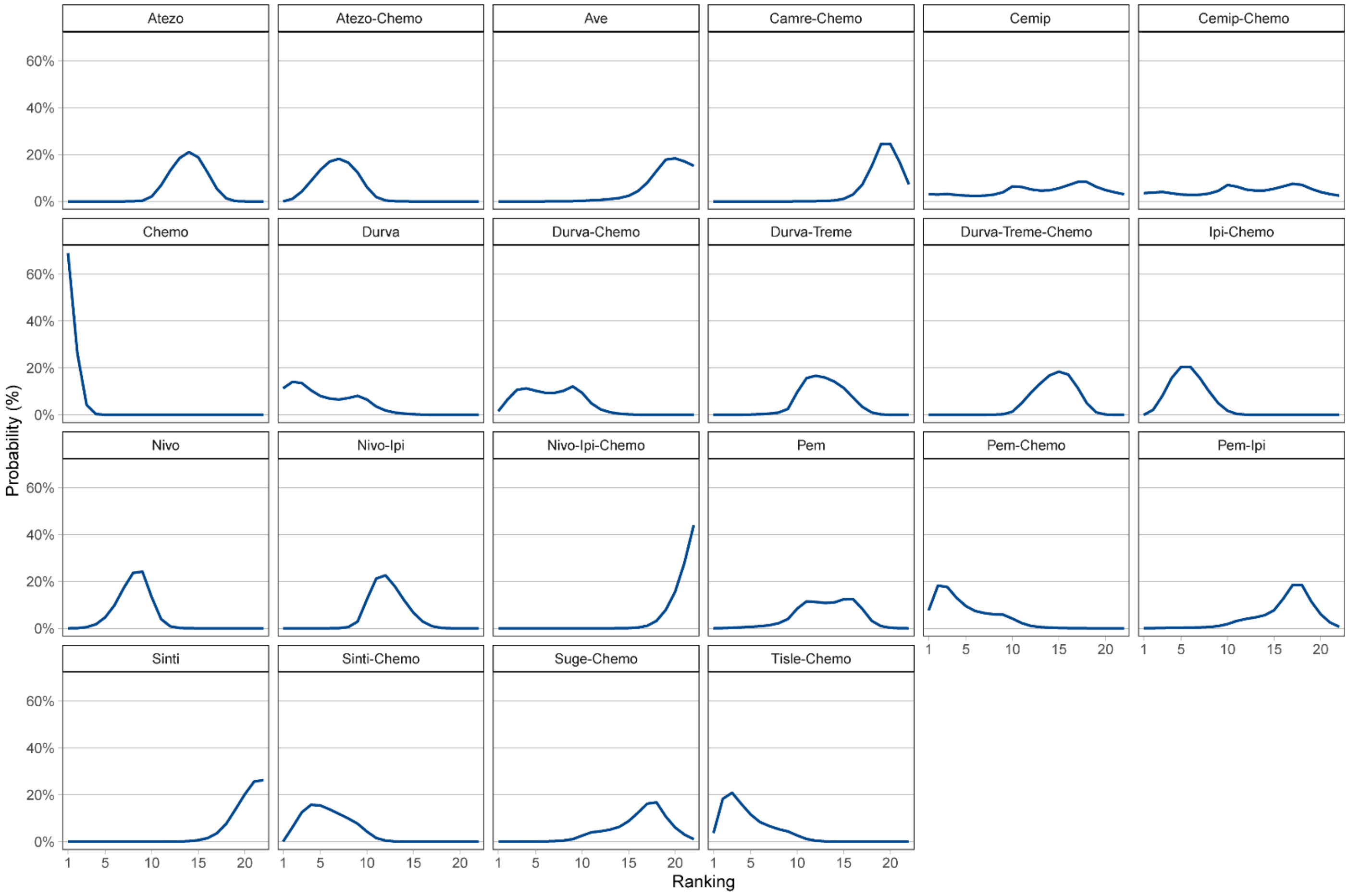

Supplementary Figure S7. Ranking of the probability of being the best treatment regimen in system organ classes specific immune-related adverse events. (A) Endocrine disorders; (B) Gastrointestinal disorders; (C) Hepatobiliary disorders; (D) Injury, poisoning and procedural complications; (E) Investigations; (F) Respiratory, thoracic and mediastinal disorders; (G) Skin and subcutaneous tissue disorders. Atezo: atezolizumab; Ave: avelumab; Beva: bevacizumab; Camre: camrelizumab; Cemip: cemiplimab; Chemo, chemotherapy; Darat: daratumumab; Dostra, dostarlimab; Durva: durvalumab; Ipi: ipilimumab; Nivo: nivolumab; NSCLC: non-small cell lung cancer; Pem: pembrolizumab; Sint: sintilimab; Sugema: sugemalimab; Tisle: tislelizumab; Treme: tremelimumab.

(A) any 3-5 treatment-related adverse events

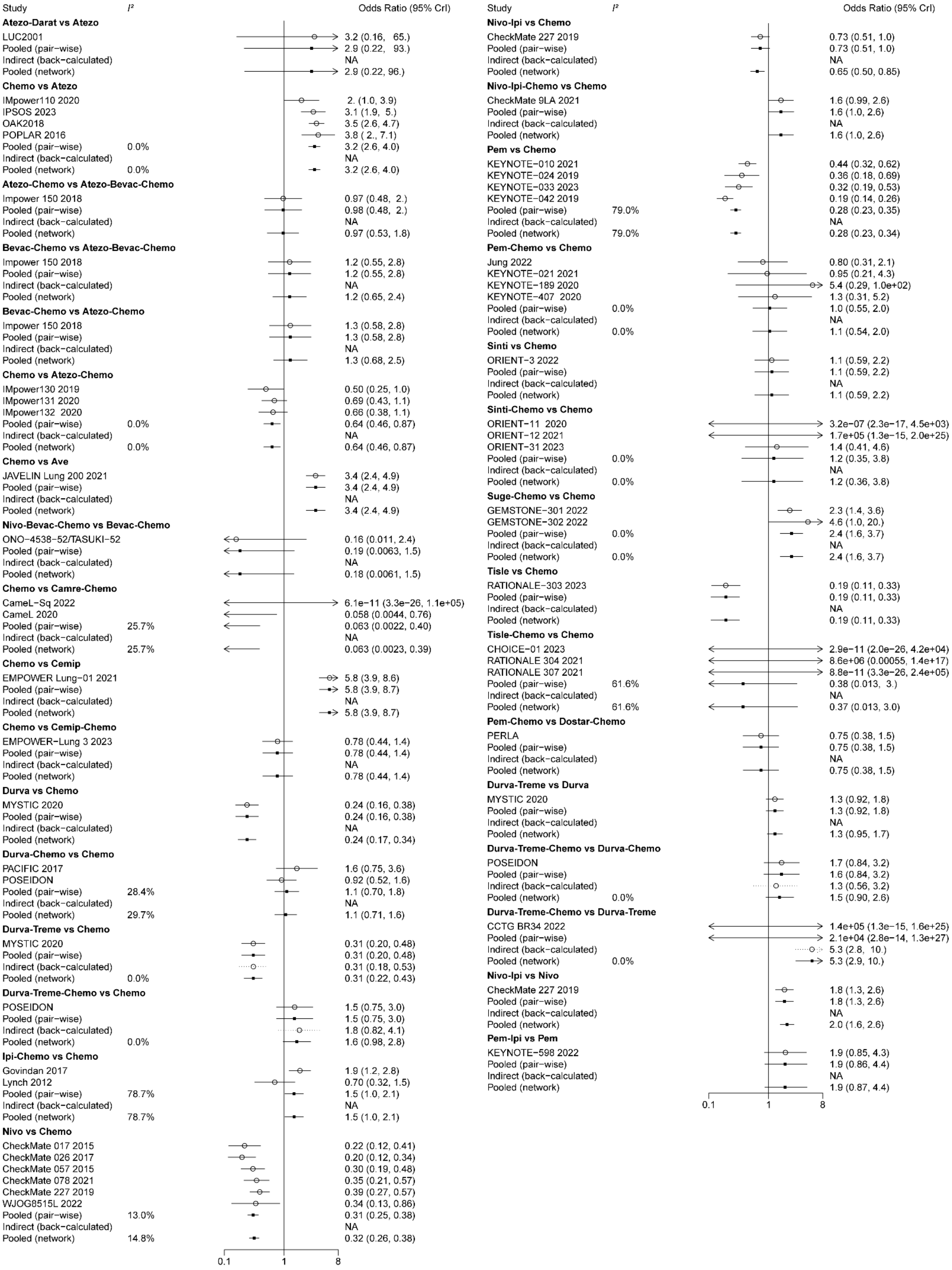

(B) grades 3-5 treatment-related adverse events

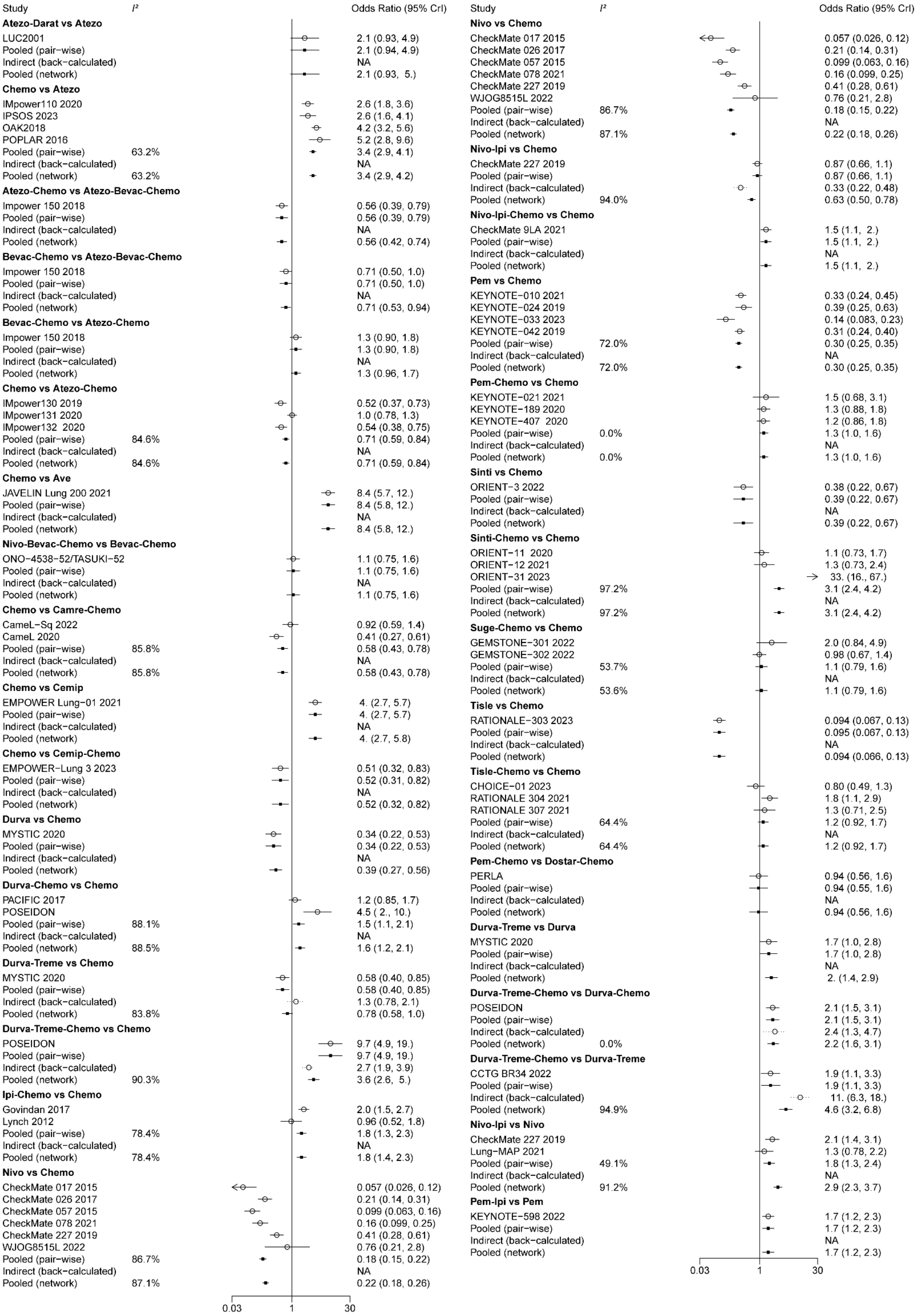

(C) any 3-5 immune-related adverse events

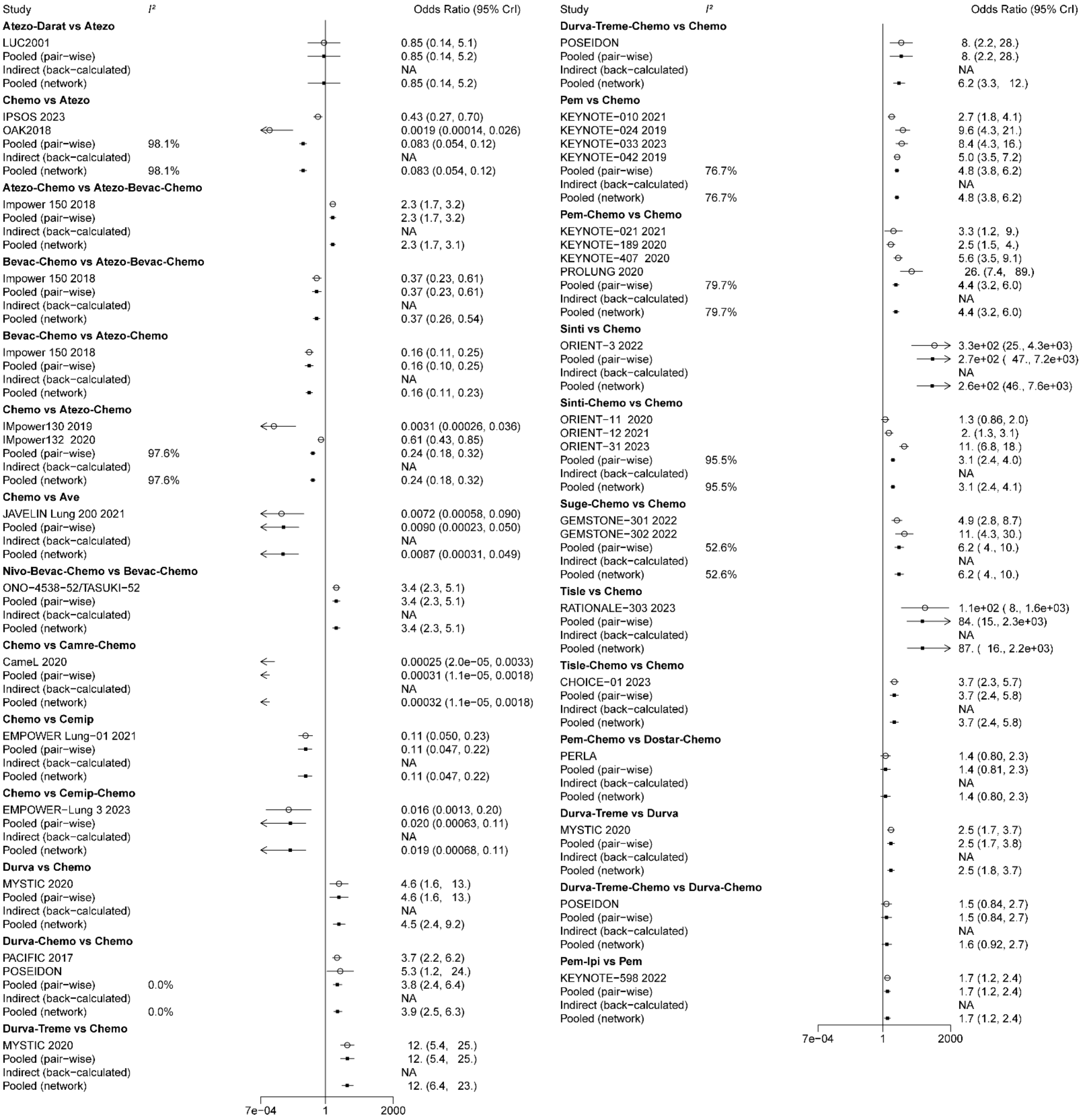

(D) grade 3-5 immune-related adverse events

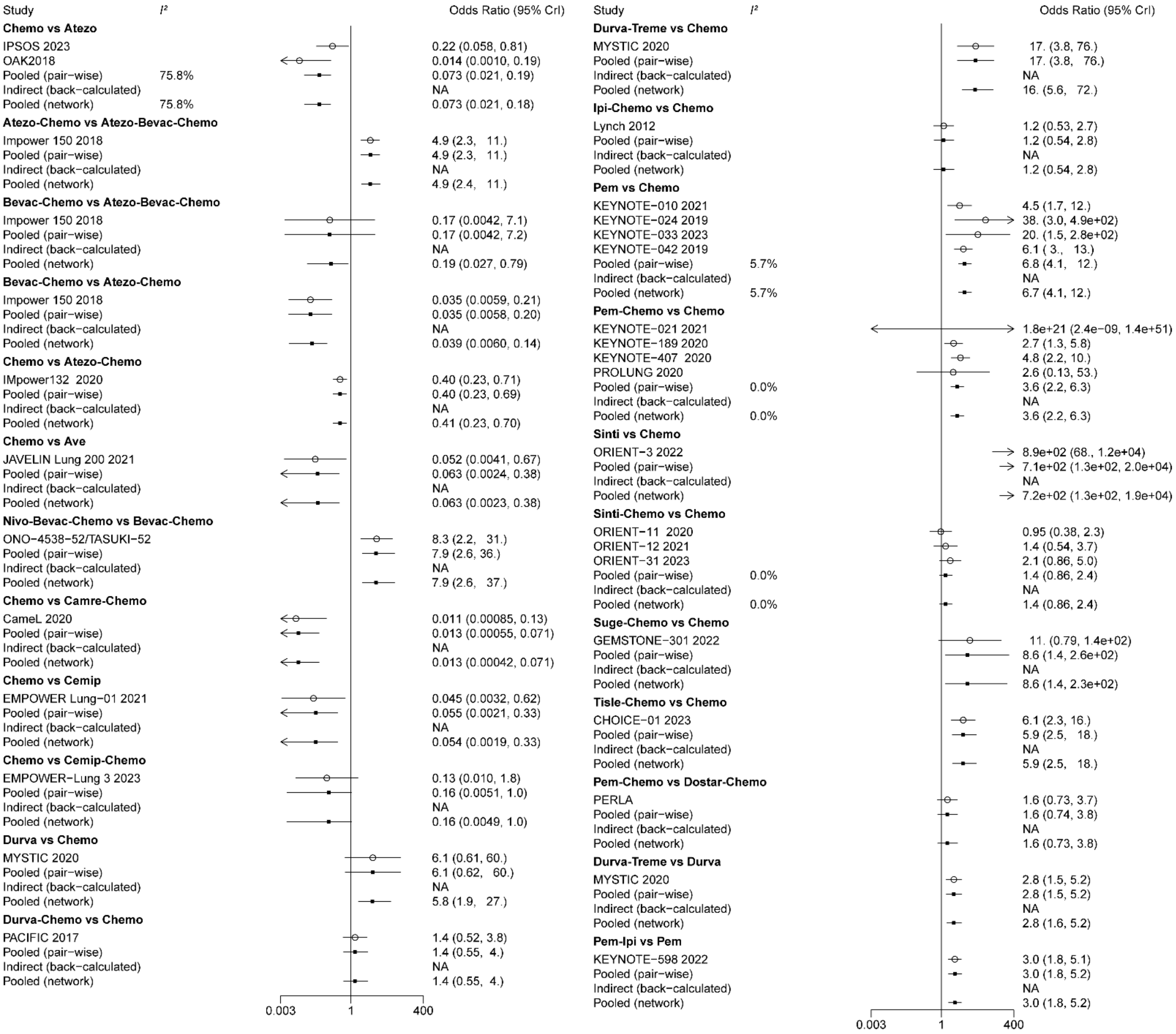

Supplementary Figure S8. Heterogeneity and inconsistency analysis of network meta-analysis results.

HLGT

Anaemias nonhaemolytic and marrow depression
Coagulopathies and bleeding diatheses (excl thrombocytopenic)
Haematological disorders NEC
Haemolyses and related conditions
Platelet disorders
Spleen, lymphatic and reticuloendothelial system disorders
White blood cell disorders
Cardiac arrhythmias
Heart failures
Myocardial disorders
Pericardial disorders
Hearing disorders
Adrenal gland disorders
Endocrine and glandular disorders NEC
Hypothalamus and pituitary gland disorders
Thyroid gland disorders
Ocular haemorrhages and vascular disorders NEC
Ocular infections, irritations and inflammations
Ocular neuromuscular disorders
Retina, choroid and vitreous haemorrhages and vascular disorders
Abdominal hernias and other abdominal wall conditions
Exocrine pancreas conditions
Gastrointestinal conditions NEC

Gastrointestinal haemorrhages NEC
Gastrointestinal inflammatory conditions
Gastrointestinal ulceration and perforation
Oral soft tissue conditions
Body temperature conditions
Fatal outcomes
General system disorders NEC
Therapeutic and nontherapeutic effects (excl toxicity)
Bile duct disorders
Hepatic and hepatobiliary disorders
Immune disorders NEC
Immunodeficiency syndromes
Bacterial infectious disorders
Fungal infectious disorders
Infections − pathogen unspecified
Mycobacterial infectious disorders
Viral infectious disorders
Bone and joint injuries
Injuries NEC
Injuries by physical agents
Medication errors and other product use errors and issues
Overdoses and underdoses NEC
Procedural related injuries and complications NEC

Cardiac and vascular investigations (excl enzyme tests)
Cytogenetic investigations and genetic analyses
Endocrine investigations (incl sex hormones)
Enzyme investigations NEC
Haematology investigations (incl blood groups)
Hepatobiliary investigations
Investigations, imaging and histopathology procedures NEC
Metabolic, nutritional and blood gas investigations
Microbiology and serology investigations
Physical examination and organ system status topics
Renal and urinary tract investigations and urinalyses
Respiratory and pulmonary investigations (excl blood gases)
Water, electrolyte and mineral investigations
Diabetic complications
Glucose metabolism disorders (incl diabetes mellitus)
Connective tissue disorders (excl congenital)
Joint disorders
Muscle disorders
Musculoskeletal and connective tissue disorders NEC
Synovial and bursal disorders
Tendon, ligament and cartilage disorders
Gastrointestinal neoplasms malignant and unspecified
Lymphomas NEC

Metastases
Miscellaneous and site unspecified neoplasms malignant and unspecified
Neoplasm related morbidities
Renal and urinary tract neoplasms malignant and unspecified
Respiratory and mediastinal neoplasms malignant and unspecified
Central nervous system infections and inflammations
Central nervous system vascular disorders
Cranial nerve disorders (excl neoplasms)
Demyelinating disorders
Encephalopathies
Mental impairment disorders
Movement disorders (incl parkinsonism)
Neurological disorders NEC
Neuromuscular disorders
Peripheral neuropathies
Anxiety disorders and symptoms
Mood disorders and disturbances NEC
Personality disorders and disturbances in behaviour
Psychiatric and behavioural symptoms NEC
Suicidal and self−injurious behaviours NEC
Bladder and bladder neck disorders (excl calculi)
Nephropathies
Prostatic disorders (excl infections and inflammations)

Bronchial disorders (excl neoplasms)
Lower respiratory tract disorders (excl obstruction and infection)
Pleural disorders
Pulmonary vascular disorders
Respiratory disorders NEC
Respiratory tract signs and symptoms
Upper respiratory tract disorders (excl infections)
Epidermal and dermal conditions
Pigmentation disorders
Skin and subcutaneous tissue disorders NEC
Skin vascular abnormalities
Cardiac therapeutic procedures
Haematological and lymphoid tissue therapeutic procedures
Hepatobiliary therapeutic procedures
Nervous system, skull and spine therapeutic procedures
Renal and urinary tract therapeutic procedures
Respiratory tract therapeutic procedures
Therapeutic procedures and supportive care NEC
Vascular therapeutic procedures
Embolism and thrombosis
Vascular haemorrhagic disorders
Vascular infections and inflammations

HLT

Marrow depression and hypoplastic anaemias
Coagulopathies
Haematological disorders
Anaemias haemolytic immune
Platelet disorders NEC
Thrombocytopenias
Lymphatic system disorders NEC
Eosinophilic disorders
Neutropenias
White blood cell abnormal findings NEC
Cardiac conduction disorders
Rate and rhythm disorders NEC
Heart failures NEC
Noninfectious myocarditis
Noninfectious pericarditis
Pericardial disorders NEC
Hearing losses
Adrenal cortical hypofunctions
Adrenal gland disorders NEC
Endocrine disorders NEC
Anterior pituitary hyperfunction
Anterior pituitary hypofunction
Hypothalamic and pituitary disorders NEC
Acute and chronic thyroiditis
Thyroid disorders NEC
Thyroid hyperfunction disorders
Thyroid hypofunction disorders
Optic nerve bleeding and vascular disorders
Iris and uveal tract infections, irritations and inflammations
Retinal, choroid and vitreous infections and inflammations
Eyelid movement disorders
Ocular nerve and muscle disorders
Retinopathies NEC
Inguinal hernias
Acute and chronic pancreatitis
Pancreatic disorders NEC
Gastrointestinal disorders NEC
Gastric and oesophageal haemorrhages
Non−site specific gastrointestinal haemorrhages
Colitis (excl infective)

Gastritis (excl infective)
Gastrointestinal inflammatory disorders NEC
Oesophageal ulcers and perforation
Oral soft tissue signs and symptoms
Stomatitis and ulceration
Body temperature altered
Death and sudden death
Adverse effect absent
Feelings and sensations NEC
General signs and symptoms NEC
Pain and discomfort NEC
Interactions
Therapeutic and nontherapeutic responses
Bile duct infections and inflammations
Structural and other bile duct disorders
Hepatic and hepatobiliary disorders NEC
Hepatic enzymes and function abnormalities
Hepatocellular damage and hepatitis NEC
Immune and associated conditions NEC
Transplant rejections
Immunodeficiency disorders NEC
Bacterial infections NEC
Corynebacteria infections
Aspergillus infections
Pneumocystis infections
Abdominal and gastrointestinal infections
Breast infections
Central nervous system and spinal infections
Dental and oral soft tissue infections
Hepatobiliary and spleen infections
Infections NEC
Lower respiratory tract and lung infections
Atypical mycobacterial infections
Tuberculous infections
Coronavirus infections
Cytomegaloviral infections
Herpes viral infections
Influenza viral infections
Fractures and dislocations NEC
Limb fractures and dislocations

Abdominal and gastrointestinal injuries NEC
Radiation injuries
Medication errors, product use errors and issues NEC
Product administration errors and issues
Product prescribing errors and issues
Product storage errors and issues in the product use system
Overdoses NEC
Underdoses NEC
Non−site specific procedural complications
Cardiac imaging procedures
Cell marker analyses
Pituitary analyses anterior
Thyroid analyses
Skeletal and cardiac muscle analyses
Tissue enzyme analyses NEC
Red blood cell analyses
White blood cell analyses
Hepatobiliary function diagnostic procedures
Investigations NEC
Blood gas and acid base analyses
Carbohydrate tolerance analyses (incl diabetes)
Virus identification and serology
Physical examination procedures and organ system status
Renal function analyses
Respiratory tract and thoracic histopathology procedures
Mineral and electrolyte analyses
Diabetic complications NEC
Diabetes mellitus (incl subtypes)
Connective tissue disorders NEC
Lupus erythematosus (incl subtypes)
Arthropathies NEC
Joint related disorders NEC
Joint related signs and symptoms
Psoriatic arthropathies
Rheumatoid arthropathies
Muscle infections and inflammations
Muscle tone abnormalities
Myopathies
Musculoskeletal and connective tissue conditions NEC
Musculoskeletal and connective tissue pain and discomfort

Synovial disorders
Tendon disorders
Colorectal neoplasms malignant
Lymphomas unspecified NEC
Metastases to specified sites
Metastases to unknown and unspecified sites
Neoplasms malignant site unspecified NEC
Neoplasms unspecified malignancy and site unspecified NEC
Oncologic complications and emergencies
Bladder neoplasms malignant
Urinary tract neoplasms malignant NEC
Non−small cell neoplasms malignant of the respiratory tract cell type specified
Respiratory tract and pleural neoplasms malignant cell type unspecified NEC
Encephalitis NEC
Central nervous system haemorrhages and cerebrovascular accidents
Eye movement disorders
Optic nerve disorders NEC
Demyelinating disorders NEC
Multiple sclerosis acute and progressive
Encephalopathies NEC
Encephalopathies toxic and metabolic
Dementia (excl Alzheimer's type)
Paralysis and paresis (excl cranial nerve)
Coordination and balance disturbances
Nervous system disorders NEC
Neuromuscular junction dysfunction
Peripheral neuropathies NEC
Panic attacks and disorders
Emotional and mood disturbances NEC
Behaviour and socialisation disturbances
Abnormal behaviour NEC
Suicidal and self−injurious behaviour
Bladder infections and inflammations
Myoneurogenic bladder disorders
Glomerulonephritis and nephrotic syndrome
Nephritis NEC
Nephropathies and tubular disorders NEC
Prostatic signs, symptoms and disorders NEC
Bronchial conditions NEC
Bronchospasm and obstruction

Lower respiratory tract inflammatory and immunologic conditions
Parenchymal lung disorders NEC
Pleural conditions NEC
Pleural infections and inflammations
Pneumothorax and pleural effusions NEC
Pulmonary thrombotic and embolic conditions
Breathing abnormalities
Conditions associated with abnormal gas exchange
Respiratory failures (excl neonatal)
Respiratory tract disorders NEC
Lower respiratory tract signs and symptoms
Laryngeal spasm, oedema and obstruction
Paranasal sinus disorders (excl infections and neoplasms)
Tracheal disorders (excl infections and neoplasms)
Bullous conditions
Connective tissue disorders
Dermatitis and eczema
Papulosquamous conditions
Psoriatic conditions
Rashes, eruptions and exanthems NEC
Hypopigmentation disorders
Panniculitides
Skin and subcutaneous tissue ulcerations
Purpura and related conditions
Cardiac device therapeutic procedures
Cardiac therapeutic procedures NEC
Blood and blood product treatment
Biliary tract and gallbladder therapeutic procedures
Skull and brain therapeutic procedures
Renal therapeutic procedures
Chest wall and mediastinal therapeutic procedures
Pleural therapeutic procedures
Tracheal therapeutic procedures
Radiotherapies site unspecified
Therapeutic procedures NEC
Venous therapeutic procedures
Peripheral embolism and thrombosis
Haemorrhages NEC
Arterial infections and inflammations

Supplementary Figure S9. HLGT and HLT legend of **Fig. 5**. HLGT, high level group term; HLT, high level term.

Supplementary Table S1. Characteristics of included randomized controlled trials for NSCLC.

Trial (Phase, Design)	Source (Year)	Registered ID (Randomization)	Sample Size (Median Age/y)	Stage (Male/Female)	Histology	Line of treatment	Treatment	
							Experiment	Control
CheckMate 9LA (III, open-label)	Lancet Onco 2021	NCT03215706 (1:1)	361/358 (65/65)	IV (215/504)	NSCLC	1L	Nivolumab 350 mg Q3W plus ipilimumab 1 mg/kg Q6W plus chemotherapy (carboplatin AUC 6 or cisplatin 75 mg/m^2 plus pemetrexed 500 mg/m^2 Q3W)	Chemotherapy (carboplatin AUC 6 or cisplatin 75 mg/m^2 plus pemetrexed 500 mg/m^2 Q3W)
CheckMate 017 (III, open-label)	NEJM 2015	NCT01642004 (1:1)	135/137 (62/64)	IIIB-IV (208/64)	Squamous NSCLC	2L+	Nivolumab 3 mg/kg Q2W	Chemotherapy (docetaxel 75 mg/m^2 Q3W)
CheckMate 026 (III, open-label)	NEJM 2017	NCT02041533 (1:1)	271/270 (63/65)	IV (332/209)	NSCLC	1L	Nivolumab 3 mg/kg Q2W	Chemotherapy (investigator's choice of platinum doublet chemotherapy Q3W)
CheckMate 057 (III, open-label)	NEJM 2015	NCT01673867 (1:1)	292/290 (61/64)	IIIB-IV (319/263)	Nonsquamous NSCLC	2L+	Nivolumab 3 mg/kg Q2W	Chemotherapy (docetaxel 75 mg/m^2 Q3W)
CheckMate 078 (III, open-label)	Lung Cancer 2021	NCT02613507 (2:1)	338/166 (60/60)	IIIB-IV (397/107)	NSCLC	2L+	Nivolumab 3 mg/kg Q2W	Chemotherapy (docetaxel 75 mg/m^2 Q3W)
CheckMate 227 (III, open-label)	NEJM 2018	NCT02477826 (1:1)	583/583 (64/64)	IV (778/388)	NSCLC	1L	Arm 1: Nivolumab 3 mg/kg Q2W plus ipilimumab 1 mg/kg Q6W<br>Arm 2: Nivolumab 240 mg Q2W	Chemotherapy (carboplatin AUC 6 or cisplatin 75 mg/m^2 plus pemetrexed 500 mg/m^2 Q3W)

EMPOWER Lung-01 (III, open-label)	Lung Cancer 2021	NCT03088540 (1:1)	356/354 (63/64)	IV (606/104)	NSCLC	1L	Cemiplimab 350 mg Q3W	platinum-doublet chemotherapy
IMpower110 (III, open-label)	NEJM 2020	NCT02409342 (1:1)	277/277 (64/65)	IV (389/165)	NSCLC	1L	Atezolizumab 1200 mg Q3W	Chemotherapy (carboplatin AUC 6 or cisplatin 75 mg/m^2 plus pemetrexed 500 mg/m^2 or gemcitabine 1000 mg/m^2 Q3W)
IMpower130 (III, open-label)	Lancet Onco 2019	NCT02367781 (2:1)	451/228 (64/65)	IV (400/279)	Nonsquamous NSCLC	1L	Atezolizumab 1200 mg Q3W plus chemotherapy (carboplatin AUC 6 Q3W plus nab-paclitaxel 100 mg/m^2 QW)	Chemotherapy (carboplatin AUC 6 Q3W plus nab-paclitaxel 100 mg/m^2 QW)
IMpower131 (III, open-label)	JTO 2020	NCT02367794 (1:1:1)	338/343/340 (66/65/65)	IV (835/186)	Squamous NSCLC	1L	Atezolizumab 1200 mg Q3W plus chemotherapy (carboplatin AUC 6 Q3W plus nab-paclitaxel 100 mg/m^2 QW)	Chemotherapy (carboplatin AUC 6 Q3W plus nab-paclitaxel 100 mg/m^2 QW)
IMpower132 (III, open-label)	JTO 2020	NCT02657434 (1:1)	292/286 (64/63)	IV (384/194)	Nonsquamous NSCLC	1L	Atezolizumab 1200 mg Q3W plus chemotherapy (carboplatin AUC 6 or cisplatin 75 mg/m^2 plus pemetrexed 500 mg/m^2 Q3W)	Chemotherapy (carboplatin AUC 6 or cisplatin 75 mg/m^2 plus pemetrexed 500 mg/m^2 Q3W)

IMpower150 (III, open-label)	NEJM 2018	NCT02366143 (1:1:1)	402/400/400 (63/63/63)	IV (720/482)	Nonsquamous NSCLC	1L	Arm 1: Atezolizumab 1200 mg Q3W plus bevacizumab 15 mg/kg Q3W plus chemotherapy (carboplatin AUC 6 plus paclitaxel 200 mg/m^2 Q3W)<br>Arm 2: Atezolizumab 1200 mg Q3W plus chemotherapy (carboplatin AUC 6 plus paclitaxel 200 mg/m^2 Q3W)	Bevacizumab 15 mg/kg Q3W plus chemotherapy (carboplatin AUC 6 plus pemetrexed 200 mg/m^2 Q3W)
JAVELIN Lung 200 (III, open-label)	JTO 2021	NCT02576574 (1:1)	396/396 (64/63)	IIIB–IV (524/250)	NSCLC	2L+	Avelumab 10 mg/kg Q2W	Chemotherapy (docetaxel 75 mg/m^2 Q3W)
KEYNOTE-010 (II/III, open-label)	JTO 2021	NCT01905657 (2:1)	690/343 (63/62)	IIIB–IV (634/399)	NSCLC	2L+	Pembrolizumab 2 mg/kg or 10 mg/kg Q3W	Chemotherapy (docetaxel 75 mg/m^2 Q3W)
KEYNOTE-021 (II, open-label)	JTO 2021	NCT02039674 (1:1)	60/63 (62.5/63.2)	IIIB–IV (48/75)	Nonsquamous NSCLC	1L	Pembrolizumab 200 mg Q3W plus chemotherapy (carboplatin AUC 5 plus pemetrexed 500 mg/m^2 Q3W)	Chemotherapy (carboplatin AUC 5 plus pemetrexed 500 mg/m^2 Q3W)
KEYNOTE-024 (III, open-label)	ASCO 2019	NCT02142738 (1:1)	154/151 (64.5/66.0)	IV (187/118)	NSCLC	1L	Pembrolizumab 200 mg Q3W	investigator’s choice of platinum-based chemotherapy

KEYNOTE-033 (III, open-label)	Annals of Oncology 2020	NCT02864394 (1:1)	213/212 (60.6/61.0)	IIIB–IV (321/104)	NSCLC	2L+	Pembrolizumab 2 mg/kg Q3W	Chemotherapy (docetaxel 75 mg/m^2 Q3W)
KEYNOTE-042 (III, open-label)	Lancet 2019	NCT02220894 (1:1)	637/637 (63.0/63.0)	NR (902/372)	NSCLC	1L	Pembrolizumab 200 mg Q3W	Chemotherapy (carboplatin AUC 5-6 plus paclitaxel 200 mg/m^2 or pemetrexed 500 mg/m^2 Q3W)
KEYNOTE-189 (III, double-blind)	ASCO 2020	NCT02578680 (2:1)	410/206 (65/63.5)	IV (363/253)	Nonsquamous NSCLC	1L	Pembrolizumab 200 mg Q3W plus PBC (carboplatin AUC 5 or cisplatin 75 mg/m^2 plus pemetrexed 500 mg/m^2 Q3W)	PBC (carboplatin AUC 5 or cisplatin 75 mg/m^2 plus pemetrexed 500 mg/m^2 Q3W)
KEYNOTE-407 (III, double-blind)	JTO 2020	NCT02775435 (1:1)	278/281 (65/65)	IV (455/104)	Squamous NSCLC	1L	Pembrolizumab 200 mg Q3W plus chemotherapy (carboplatin AUC 6 plus paclitaxel 200 mg/m^2 Q3W)	Chemotherapy (carboplatin AUC 6 plus paclitaxel 200 mg/m^2 Q3W)
KEYNOTE-598 (III, double-blind)	ASCO 2021	NCT03302234 (1:1)	284/284 (64/65)	IV (393/375)	NSCLC	1L	Ipilimumab 1 mg/kg Q6W plus pembrolizumab 200 mg Q3W	Pembrolizumab 200 mg Q3W
MYSTIC (III, open-label)	Jama Onco 2020	NCT02453282 (1:1:1)	374/372/372 (64/65/64.5)	IV (772/346)	NSCLC	1L	Arm 1: Durvalumab 20 mg/kg plus tremelimumab 1 mg/kg Q4W<br>Arm 2: Durvalumab 20 mg/kg Q4W	Chemotherapy (carboplatin AUC 6 or cisplatin 75 mg/m^2 plus pemetrexed 500 mg/m^2 Q3W)

OAK (III, open-label)	JTO 2018	NCT02008227 (1:1)	425/425 (63/64)	IIIB-IV (520/330)	NSCLC	2L+	Atezolizumab 1200 mg Q3W	Docetaxel 75 mg/m² Q3W
PACIFIC (III, double-blind)	NEJM 2017	NCT02125461 (2:1)	476/237 (64/64)	III (500/213)	NSCLC	2L+	Durvalumab 10 mg/kg Q2W plus PBC	PBC
POPLAR (II, open-label)	Lancet 2016	NCT01903993 (1:1)	144/143 (62/62)	NR (169/118)	NSCLC	2L+	Atezolizumab 1200 mg Q3W	Docetaxel 75 mg/m² Q3W
Govindan 2017 (III, double-blind)	ASCO 2017	NCT01285609 (1:1)	388/361 (64/64)	IV (635/114)	Squamous NSCLC	1L	Ipilimumab 10 mg/kg plus carboplatin AUC 6 plus paclitaxel 175 mg/m^2 Q3W	Chemotherapy (carboplatin AUC 6 plus paclitaxel 175 mg/m2 Q3W)
CameL (III, open-label)	Lancet Respir Med 2020	NCT03134872 (1:1)	205/207 (59/61)	IIIB-IV (295/117)	Nonsquamous NSCLC	1L	Camrelizumab 200 mg Q3W plus chemotherapy (carboplatin AUC 5 plus pemetrexed 500 mg/m^2 Q3W)	Chemotherapy (carboplatin AUC 5 plus pemetrexed 500 mg/m^2 Q3W)
CameL-Sq (III, double-blind)	JTO 2022	NCT03668496 (1:1)	193/196 (64/62)	IIIB-IV (359/30)	Squamous NSCLC	1L	Camrelizumab 200 mg Q3W plus chemotherapy (carboplatin AUC 5 plus paclitaxel 175 mg/m^2 Q3W)	Chemotherapy (carboplatin AUC 5 plus paclitaxel 175 mg/m^2 Q3W)
RATIONALE 304 (III, open-label)	JTO 2021	NCT03663205 (2:1)	223/111 (60/61)	IIIB–IV (247/87)	Nonsquamous NSCLC	1L	Tislelizumab 200 mg plus chemotherapy (carboplatin AUC 5 or cisplatin 75 mg/m^2 plus pemetrexed 500 mg/m2 Q3W)	Chemotherapy (carboplatin AUC 5 or cisplatin 75 mg/m^2 plus pemetrexed 500 mg/m^2 Q3W)

ORIENT-11(III, double-blind)	JTO 2021	NCT03607539 (2:1)	266/131 (61/61)	IIIB–IV (303/94)	Nonsquamous NSCLC	1L	Sintilimab 200 mg plus chemotherapy (carboplatin AUC 5 or cisplatin 75 mg/m^2 plus pemetrexed 500 mg/m^2 Q3W)	Chemotherapy (carboplatin AUC 5 or cisplatin 75 mg/m^2 plus pemetrexed 500 mg/m^2 Q3W)
ORIENT-12 (III, double-blind)	JTO 2021	NCT03629925 (1:1)	179/178 (64/62)	IIIB–IV (327/30)	Squamous NSCLC	1L	Sintilimab 200 mg plus chemotherapy (gemcitabine 1 g/m2, d 1, 8 plus carboplatin AUC 5, d 1 or cisplatin 75 mg/m^2 Q3W)	Chemotherapy (gemcitabine 1 g/m2, d 1, 8 plus carboplatin AUC 5, d 1 or cisplatin 75 mg/m^2 Q3W)
Lynch 2012 (II, double-blind)	ASCO 2012	NR (2:1)	138/66 (60/62)	IIIB-IV (151/53)	NSCLC	1L	Ipilimumab 10 mg/kg plus carboplatin AUC 6 plus paclitaxel 175 mg/m^2 Q3W	Chemotherapy (carboplatin AUC 6 plus paclitaxel 175 mg/m^2 Q3W)
RATIONALE 307 (III, open-label)	JAMA Onco 2021	NCT03594747 (1:1:1)	120/119/121 (60/63/62)	IIIB–IV (330/30)	Squamous NSCLC	1L	Arm 1: Tislelizumab 200 mg plus chemotherapy (carboplatin AUC 5 plus paclitaxel 175 mg/m^2 Q3W) Arm 2: Tislelizumab 200 mg plus chemotherapy (carboplatin AUC 5 Q3W plus nab-paclitaxel 100 mg/m^2 QW)	Chemotherapy (carboplatin AUC 5 plus paclitaxel 175 mg/m^2 Q3W)
CCTG BR34 (III, open-label)	JTO 2022	NCT03057106 (1:1)	151/150 (65/63)	IV (162/139)	NSCLC	1L	Durvalumab 1500 mg plus tremelimumab 75 mg Q4W plus chemotherapy (carboplatin AUC 6 or cisplatin 75 mg/m^2 plus pemetrexed 500 mg/m^2 Q3W)	Durvalumab 1500 mg plus tremelimumab 75 mg Q4W

Lung-MAP (III, open-label)	JAMA Onco 2021	NCT02785952 (1:1)	125/127 (67.5/68.1)	IV (169/83)	Squamous NSCLC	2L+	Nivolumab 3 mg/kg Q2W plus ipilimumab 1 mg/kg Q6W	Nivolumab 3 mg/kg Q2W
GEMSTONE-301 (III, double-blind)	Lancet Oncol 2022	NCT03728556 (2:1)	255/126 (61/60)	III (351/30)	NSCLC	2L+	Sugemalimab 1200 mg Q3W plus PBC	PBC
GEMSTONE-302 (III, double-blind)	Lancet Oncol 2022	NCT03789604 (2:1)	320/159 (62/64)	IV (383/96)	NSCLC	1L	Sugemalimab 1200 mg plus chemotherapy (carboplatin AUC 5 plus pemetrexed 500 mg/m^2 or paclitaxel 175 mg/m² Q3W)	Chemotherapy (carboplatin AUC 5 plus pemetrexed 500 mg/m2 or paclitaxel 175 mg/m² Q3W)
ONO-4538-52/TASUKI-52 (III, double-blind)	ESMO 2021	NCT03117049 (1:1)	275/275 (66/66)	IIIB–IV (411/139)	Nonsquamous NSCLC	1L	Nivolumab 360 mg plus chemotherapy (carboplatin AUC 6 plus paclitaxel 175 mg/m² plus bevacizumab 15 mg/kg Q3W)	Chemotherapy (carboplatin AUC 6 plus paclitaxel 175 mg/m² plus bevacizumab 15 mg/kg Q3W)
LUC2001 (II, open-label)	JTO 2020	NCT03023423 (1:1)	46/46 (65.5/61)	IIIB–IV (66/26)	NSCLC	2L+	Daratumumab 16 mg/kg plus atezolizumab 1200 mg Q3W	Atezolizumab 1200 mg Q3W
PROLUNG (II, open-label)	JAMA Onco 2020	NCT02574598 (1:1)	40/38 (50.1/62.1)	NR (32/46)	NSCLC	2L+	Pembrolizumab 200 mg plus docetaxel 75 mg/m² Q3W	Docetaxel 75 mg/m² Q3W

EMPOWER-Lung 3 (III, double-blind)	JTO 2023	NCT03409614 (2:1)	312/154 (63/63)	IIIB/C-IV(391/75)	NSCLC	1L	Cemiplimab 350 mg Q3W plus chemotherapy (platinum-doublet chemotherapy Q3W)	Chemotherapy (platinum-doublet chemotherapy Q3W)
WJOG8515L (II, open-label)	Clin Cancer Res 2022	jRCTs051180133 (1:1)	52/50 (70.5/67)	IIIB-IV (43/59)	NSCLC	1L	Nivolumab 3 mg/kg Q2W	Chemotherapy (pemetrexed 500 mg/m^2 plus carboplatin AUC 6 Q3W)
Jung 2022 (II, double-blind)	Clin Cancer Res 2022	NR (1:1)	47/51 (63/64)	NR (80/18)	NSCLC	1L	Pembrolizumab 200 mg Q3W plus chemotherapy (investigator's decision)	Chemotherapy (investigator's decision)
Lai 2022 (II, open-label)	JTO Clin Res Rep 2022	NCT03091491 (1:1)	15/16 (70.7/59.1)	IIIB-IV (19/12)	NSCLC	2L	Nivolumab 3 mg/kg Q2W	Nivolumab 3 mg/kg Q2W plus ipilimumab 1 mg/kg Q6W
ORIENT-3 (III, open-label)	Cancer Commun 2022	NCT03150875 (1:1)	145/135 (61/60)	IIIB/IIIC/IV (258/22)	Squamous NSCLC	2L	Sintilimab 200 mg Q3W	Docetaxel 75 mg/m^2 Q3W
RATIONALE-303 (III, open-label)	JTO 2023	NCT03358875 (2:1)	535/270 (61/61)	NR (622/183)	NSCLC	2L	Tislelizumab 200 mg Q3W	Docetaxel 75 mg/m^2 Q3W

CHOICE-01 (III, double-blind)	JCO 2023	NCT03856411 (2:1)	309/156 (63/61)	IIIB-IVB (377/88)	NSCLC	1L	Toripalimab 240 mg Q3W plus chemotherapy (nabpaclitaxel 100 mg/m^2 plus carboplatin AUC 5 (or pemetrexed 500 mg/m^2 plus cisplatin 75 mg/m^2 or carboplatin AUC 5)	Chemotherapy (nabpaclitaxel 100 mg/m^2 plus carboplatin AUC 5 (or pemetrexed 500 mg/m^2 plus cisplatin 75 mg/m^2 or carboplatin AUC 5)
ORIENT-31 (III, open-label)	Lancet Respir Med 2023	NCT03802240 (1:1:1)	158/158/160 (58.5/57.5/56)	IIIB-IV (194/282)	NSCLC	2L	Arm 1: Sintilimab 200 mg Q3W plus chemotherapy (IBI305 15 mg/kg plus pemetrexed 500 mg plus cisplatin 75 mg/m^2) Q3W<br>Arm 1: Sintilimab 200 mg Q3W plus chemotherapy (pemetrexed 500 mg plus cisplatin 75 mg/m^2) Q3W	Chemotherapy (pemetrexed 500 mg plus cisplatin 75 mg/m^2) Q3W
POSEIDON (III, open-label)	JTO2023	NCT03164616 (1:1:1)	338/338/337 (63/64.5/64)	IVA-IVB (770/243)	NSCLC	1L	Arm 1: Tremelimumab 75 mg plus durvalumab 1,500 mg plus chemotherapy Q3W<br>Arm 2: Durvalumab 1500 mg plus chemotherapy Q3W	Chemotherapy Q3W
IPSOS (III, open-label)	Lancet 2023	NCT03191786 (2:1)	302/151 (75/75)	IIIB/IV (328/125)	NSCLC	1L	Atezolizumab 1200 mg Q3W	Single-agent chemotherapy
PERLA (II, double-blind)	Nat Commun 2023	NCT04581824 (1:1)	121/122 (64/65)	I-IV (162/81)	Nonsquamous NSCLC	1L	Dostarlimab 500mg Q3W plus chemotherapy (pemetrexed 500mg/m^2 plus cisplatin 75mg/m^2 or carboplatin AUC 5)	Pembrolizumab 200 mg Q3W plus chemotherapy (pemetrexed 500mg/m^2 plus cisplatin 75mg/m^2 or carboplatin AUC 5)

Supplementary Table S2. Risk of bias assessment of the included studies.

Study	D1	D2	D3	D4	D5	Overall
CheckMate 9LA	Low	Low	Low	Low	Low	Low
CheckMate 017	Some concerns	Low	Low	Low	Some concerns	Some concerns
CheckMate 026	Some concerns	Low	Low	Low	Low	Some concerns
CheckMate 057	Some concerns	Low	Low	Low	Some concerns	Some concerns
CheckMate 078	Low	Low	Low	Low	Low	Low
CheckMate 227	Low	Low	Low	Low	Low	Low
EMPOWER Lung-01	Low	Low	Low	Low	Low	Low
IMpower110	Low	Low	Low	Low	Low	Low
IMpower130	Low	Low	Low	Low	Low	Low
IMpower131	Low	Low	Low	Low	Low	Low
IMpower132	Low	Low	Low	Low	Low	Low
IMpower150	Low	Low	Low	Low	Low	Low
JAVELIN Lung 200	Low	Low	Low	Low	Low	Low
KEYNOTE-010	Low	Low	Low	Low	Low	Low
KEYNOTE-021	Low	Low	Low	Low	Low	Low
KEYNOTE-024	Some concerns	Low	Low	Low	Low	Some concerns
KEYNOTE-033	Low	Low	Low	Low	Low	Low
KEYNOTE-042	Low	Low	Low	Low	Low	Low
KEYNOTE-189	Low	Low	Low	Low	Low	Low
KEYNOTE-407	Low	Low	Low	Low	Low	Low
KEYNOTE-598	Low	Low	Low	Low	Low	Low
MYSTIC	Low	Low	Low	Low	Low	Low
OAK	Low	Low	Low	Low	Low	Low
PACIFIC	Low	Low	Low	Low	Low	Low
POPLAR	Low	Some concerns	Low	Low	Low	Some concerns
Govindan 2017	Low	Some concerns	Low	Low	Low	Some concerns
CameL	Low	Some concerns	Low	Low	Low	Some concerns
CameL-Sq	Low	Low	Low	Low	Low	Low
RATIONALE 304	Low	Some concerns	Low	Low	Low	Some concerns
ORIENT-11	Low	Low	Low	Low	Low	Low
ORIENT-12	Low	Some concerns	Low	Low	Low	Some concerns
Lynch 2012	Low	Some concerns	Low	Low	Low	Some concerns
RATIONALE 307	Low	Low	Low	Low	Low	Low
CCTG BR34	Low	Some concerns	Low	Low	Low	Some concerns
Lung-MAP	Low	Some concerns	Low	Low	Low	Some concerns
GEMSTONE-301	Low	Low	Low	Low	Low	Low
GEMSTONE-302	Low	Low	Low	Low	Low	Low
ONO-4538-52/TASUKI-52	Low	Some concerns	Low	Low	Low	Some concerns
LUC2001	Low	Some concerns	Low	Low	Low	Some concerns
PROLUNG	Low	Some concerns	Low	Low	Low	Some concerns
EMPOWER-Lung 3	Low	Low	Low	Low	Low	Low
WJOG8515L	Low	Some concerns	Low	Low	Some concerns	Some concerns
Jung 2022	Low	Some concerns	Low	Low	Some concerns	Some concerns
Lai 2022	Low	Some concerns	Low	Low	Low	Some concerns
ORIENT-3	Low	Low	Low	Low	Low	Low
RATIONALE-303	Low	Low	Low	Low	Low	Low
CHOICE-01	Low	Low	Low	Low	Low	Low
ORIENT-31	Low	Low	Low	Low	Low	Low
POSEIDON	Low	Low	Low	Low	Low	Low
IPSOS	Low	Low	Low	Low	Low	Low
PERLA	Low	Low	Low	Low	Low	Low

D1: Bias due to randomisation.

D2: Bias due to deviations from intended intervention.

D3: Bias due to missing data.

D4: Bias due to outcome measurement.

D5: Bias due to selection of reported result.

Supplementary Table S3. Characteristics of reports with ICI-related adverse events in NSCLC patients.

Clinical characteristics	Primary suspect (n = 23,941)
**Gender**	
Male	14,685 (68%)
Female	6,866 (32%)
Missing	2,390
**Age [Median (IQR)]**	67 (59, 74)
Missing	5,251
**Age group**	
18–64	7,405 (40%)
65–75	6,912 (37%)
≥75	4,373 (23%)
Missing	5,251
**Country**	
United States of America	4,499 (19%)
Australia	416 (1.7%)
Canada	536 (2.2%)
United Kingdom of Great Britain and Northern Ireland	297 (1.2%)
France	984 (4.1%)
Germany	1,002 (4.2%)
Italy	531 (2.2%)
Japan	9,392 (39%)
Other country	6,284 (26%)
**Received year**	
2013	12 (<0.1%)
2014	46 (0.2%)
2015	547 (2.3%)
2016	1,766 (7.4%)
2017	2,648 (11%)
2018	3,158 (13%)
2019	4,036 (17%)
2020	3,032 (13%)
2021	2,947 (12%)
2022	3,038 (13%)
2023	2,711 (11%)
**Case priority**	
Direct	161 (0.7%)
Expedited	22,343 (93%)
Non-expedited	1,437 (6.0%)
**Reporter type**	
Consumer	5,396 (23%)

Healthcare professional	18,008 (77%)
Lawyer	3 (<0.1%)
Missing	534
**Treatment strategy**	
Atezo	1,861 (7.8%)
Atezo-Chemo	1,758 (7.3%)
Atezo-Ipi	13 (<0.1%)
Atezo-Ipi-Treme	2 (<0.1%)
Atezo-Ipi-Chemo	2 (<0.1%)
Ave	66 (0.3%)
Ave-chemo	7 (<0.1%)
Cemip	50 (0.2%)
Cemip-Chemo	18 (<0.1%)
Cemip-Ipi	4 (<0.1%)
Cemip-Ipi-Chemo	6 (<0.1%)
Dostar	5 (<0.1%)
Dostar-Chemo	25 (0.1%)
Durva	2,802 (12%)
Durva-Chemo	177 (0.7%)
Durva-Ipi	1 (<0.1%)
Durva-Ipi-Chemo	1 (<0.1%)
Durva-Treme	215 (0.9%)
Durva-Treme-Chemo	128 (0.5%)
Ipi	22 (<0.1%)
ipi-Chemo	76 (0.3%)
Nivo	7,695 (32%)
Nivo-Chemo	597 (2.5%)
Nivo-Ipi	1,839 (7.7%)
Nivo-Ipi-Chemo	947 (4.0%)
Nivo-Treme	3 (<0.1%)
Pem	3,896 (16%)
Pem-Chemo	1,709 (7.1%)
Pem-Ipi	12 (<0.1%)
Pem-Ipi-Chemo	4 (<0.1%)

Supplementary Table S4. The case number of different adverse events in NSCLC cases receiving ICI treatment in FAERS database.

**Adverse Event (preferred term)**	**Frequency**	**Percentage (%)**
Malignant neoplasm progression	3337	5.271%
Death	1978	3.124%
Pneumonitis	1093	1.726%
Pneumonia	1087	1.717%
Pyrexia	1042	1.646%
Interstitial lung disease	1025	1.619%
Diarrhoea	927	1.464%
Dyspnoea	803	1.268%
Radiation pneumonitis	800	1.264%
Rash	797	1.259%
Fatigue	612	0.967%
Decreased appetite	594	0.938%
Nausea	528	0.834%
Off label use	516	0.815%
Anaemia	494	0.780%
Hypothyroidism	477	0.753%
Colitis	474	0.749%
Asthenia	445	0.703%
Febrile neutropenia	437	0.690%
Pleural effusion	422	0.667%
Hepatic function abnormal	395	0.624%
Malaise	386	0.610%
Vomiting	378	0.597%
Respiratory failure	358	0.565%
Lung disorder	357	0.564%
Platelet count decreased	352	0.556%
Arthralgia	349	0.551%
Acute kidney injury	345	0.545%
Cough	324	0.512%
Neutrophil count decreased	323	0.510%
Pruritus	320	0.505%
Adrenal insufficiency	319	0.504%
General physical health deterioration	317	0.501%
Renal impairment	302	0.477%
Neutropenia	293	0.463%
Thrombocytopenia	288	0.455%
Sepsis	276	0.436%
Pain	272	0.430%
Liver disorder	262	0.414%
Constipation	243	0.384%
Non-small cell lung cancer	231	0.365%
Metastases to central nervous system	226	0.357%
White blood cell count decreased	225	0.355%
Immune-mediated lung disease	221	0.349%
Haemoptysis	220	0.347%

Weight decreased	218	0.344%
Pulmonary embolism	212	0.335%
Pneumothorax	210	0.332%
Headache	207	0.327%
Immune-mediated enterocolitis	206	0.325%
Cardiac failure	205	0.324%
Pericardial effusion	205	0.324%
Dehydration	203	0.321%
Neuropathy peripheral	202	0.319%
Hyperthyroidism	200	0.316%
Aspartate aminotransferase increased	198	0.313%
Hyponatraemia	198	0.313%
Myocarditis	196	0.310%
Disease progression	193	0.305%
Alanine aminotransferase increased	191	0.302%
Cerebral infarction	187	0.295%
Drug ineffective	186	0.294%
Immune-mediated hepatic disorder	185	0.292%
Fall	184	0.291%
Pulmonary toxicity	183	0.289%
Myalgia	182	0.287%
Infusion related reaction	180	0.284%
Atrial fibrillation	179	0.283%
Dizziness	171	0.270%
Back pain	169	0.267%
Renal failure	161	0.254%
Infection	160	0.253%
Stomatitis	160	0.253%
Adverse event	160	0.253%
Hepatitis	156	0.246%
Cholangitis	154	0.243%
Muscular weakness	153	0.242%
Hypotension	152	0.240%
Hypoxia	148	0.234%
Chest pain	143	0.226%
Inappropriate schedule of product administration	143	0.226%
Myositis	139	0.220%
Abdominal pain	138	0.218%
Arthritis	135	0.213%
Cytokine release syndrome	135	0.213%
Blood creatinine increased	134	0.212%
Diabetes mellitus	134	0.212%
Pneumonia bacterial	134	0.212%
Skin disorder	133	0.210%
Type 1 diabetes mellitus	133	0.210%
Chronic obstructive pulmonary disease	130	0.205%
Intentional product use issue	128	0.202%
Pain in extremity	127	0.201%
Pneumonia aspiration	126	0.199%

Renal disorder	126	0.199%
Drug-induced liver injury	124	0.196%
Adrenocorticotropic hormone deficiency	123	0.194%
C-reactive protein increased	121	0.191%
Enterocolitis	121	0.191%
Hypokalaemia	121	0.191%
Oedema peripheral	120	0.190%
Encephalitis	118	0.186%
Seizure	118	0.186%
Hypoaesthesia	117	0.185%
Hypophysitis	117	0.185%
Prescribed underdose	117	0.185%
COVID-19	117	0.185%
Myasthenia gravis	116	0.183%
Diabetic ketoacidosis	114	0.180%
Rheumatoid arthritis	112	0.177%
Immune-mediated adverse reaction	112	0.177%
Hypopituitarism	111	0.175%
Myelosuppression	110	0.174%
Myocardial infarction	110	0.174%
Urinary tract infection	110	0.174%
Thyroid disorder	109	0.172%
Confusional state	108	0.171%
Dysphagia	107	0.169%
Erythema	107	0.169%
Tubulointerstitial nephritis	106	0.167%
Hypertension	104	0.164%
Thyroiditis	104	0.164%
Gait disturbance	103	0.163%
Cholangitis sclerosing	102	0.161%
Pancytopenia	102	0.161%
Pemphigoid	102	0.161%
Chills	101	0.160%
Fulminant type 1 diabetes mellitus	99	0.156%
Tumour pseudoprogression	99	0.156%
Immune thrombocytopenia	99	0.156%
Condition aggravated	98	0.155%
Herpes zoster	98	0.155%
Septic shock	98	0.155%
Cerebrovascular accident	95	0.150%
Delirium	95	0.150%
Altered state of consciousness	94	0.148%
Drug eruption	94	0.148%
Stevens-Johnson syndrome	94	0.148%
Disseminated intravascular coagulation	92	0.145%
Immune-mediated dermatitis	92	0.145%
Hyperglycaemia	90	0.142%
Product use issue	90	0.142%
Erythema multiforme	89	0.141%

Nephritis	88	0.139%
Gamma-glutamyltransferase increased	87	0.137%
Pancreatitis	87	0.137%
Hepatotoxicity	86	0.136%
Peripheral swelling	86	0.136%
Abdominal pain upper	85	0.134%
Blood creatine phosphokinase increased	85	0.134%
Hyperkalaemia	85	0.134%
Organising pneumonia	83	0.131%
Leukopenia	82	0.130%
Nasopharyngitis	82	0.130%
Alopecia	81	0.128%
Lymphocyte count decreased	81	0.128%
Pulmonary oedema	81	0.128%
Acute respiratory failure	80	0.126%
Secondary adrenocortical insufficiency	80	0.126%
Hepatic enzyme increased	80	0.126%
Immune-mediated hepatitis	80	0.126%
Rash maculo-papular	78	0.123%
Multiple organ dysfunction syndrome	78	0.123%
Cardiac tamponade	77	0.122%
Oedema	77	0.122%
Weight increased	77	0.122%
Autoimmune hepatitis	76	0.120%
Hepatic failure	76	0.120%
Deep vein thrombosis	76	0.120%
Toxicity to various agents	76	0.120%
Metastases to bone	75	0.118%
Blood alkaline phosphatase increased	75	0.118%
Hypercalcaemia	72	0.114%
Somnolence	71	0.112%
Pneumocystis jirovecii pneumonia	71	0.112%
Hypersensitivity	70	0.111%
Lymphadenopathy	70	0.111%
Pericarditis	70	0.111%
Dermatitis	69	0.109%
Musculoskeletal pain	69	0.109%
Thrombosis	69	0.109%
Haemophagocytic lymphohistiocytosis	68	0.107%
Insomnia	67	0.106%
Brain oedema	67	0.106%
Adrenal disorder	66	0.104%
Ascites	65	0.103%
Mucosal inflammation	64	0.101%
Productive cough	64	0.101%
Psoriasis	64	0.101%
Product use in unapproved indication	64	0.101%
Bronchitis	63	0.100%
Cardiac arrest	63	0.100%

Cholecystitis	63	0.100%
Metastases to liver	63	0.100%
Urticaria	62	0.098%
Anxiety	61	0.096%
Syncope	61	0.096%
Toxic epidermal necrolysis	61	0.096%
Inflammation	61	0.096%
Transfusion	61	0.096%
Depression	60	0.095%
Non-small cell lung cancer recurrent	60	0.095%
Acute myocardial infarction	59	0.093%
Encephalopathy	59	0.093%
Bone pain	58	0.092%
Epistaxis	58	0.092%
Haematochezia	58	0.092%
Ileus	58	0.092%
Rhabdomyolysis	58	0.092%
Immune-mediated myocarditis	58	0.092%
Dry skin	57	0.090%
Meningitis	57	0.090%
Anaphylactic reaction	56	0.088%
Colitis ulcerative	56	0.088%
Gastrointestinal haemorrhage	56	0.088%
Haemoglobin decreased	56	0.088%
No adverse event	56	0.088%
Asthma	55	0.087%
Depressed level of consciousness	55	0.087%
Oxygen saturation decreased	55	0.087%
Pulmonary haemorrhage	55	0.087%
Sudden death	55	0.087%
Bone marrow failure	55	0.087%
Blood thyroid stimulating hormone increased	54	0.085%
Loss of consciousness	54	0.085%
Paraesthesia	54	0.085%
Rash pruritic	54	0.085%
Respiratory distress	54	0.085%
Metastases to meninges	54	0.085%
Skin toxicity	53	0.084%
Cellulitis	52	0.082%
Cytopenia	52	0.082%
Tachycardia	51	0.081%
Blood bilirubin increased	50	0.079%
Dyspnoea exertional	50	0.079%
Hypoalbuminaemia	50	0.079%
Prescribed overdose	50	0.079%
Liver function test increased	50	0.079%
Acute respiratory distress syndrome	49	0.077%
Eczema	49	0.077%
Feeling abnormal	49	0.077%

Lower respiratory tract infection	49	0.077%
Skin exfoliation	49	0.077%
Immune-mediated hypothyroidism	49	0.077%
Product dose omission issue	49	0.077%
Arrhythmia	48	0.076%
Cardio-respiratory arrest	48	0.076%
Eosinophil count increased	48	0.076%
Hypothalamo-pituitary disorder	48	0.076%
Peritonitis	48	0.076%
Vision blurred	48	0.076%
Jaundice	47	0.074%
Melaena	47	0.074%
Peripheral sensory neuropathy	47	0.074%
Visual impairment	47	0.074%
Cardiac disorder	47	0.074%
Pulmonary fibrosis	46	0.073%
Cerebral haemorrhage	45	0.071%
Influenza	45	0.071%
Malignant pleural effusion	45	0.071%
Polyarthritis	45	0.071%
White blood cell count increased	45	0.071%
Infectious pleural effusion	45	0.071%
Blood pressure decreased	44	0.069%
Neoplasm	44	0.069%
Proteinuria	44	0.069%
Thrombophlebitis migrans	44	0.069%
Tremor	44	0.069%
Respiratory tract infection	44	0.069%
Blood glucose increased	43	0.068%
Dysphonia	43	0.068%
Enteritis	43	0.068%
Gastritis	43	0.068%
Influenza like illness	43	0.068%
Memory impairment	43	0.068%
Lymphangiosis carcinomatosa	43	0.068%
Transaminases increased	43	0.068%
Haemorrhage	43	0.068%
Neoplasm progression	43	0.068%
Chest discomfort	42	0.066%
Diverticulitis	42	0.066%
Dry mouth	42	0.066%
Pleurisy	42	0.066%
Anaphylactic shock	41	0.065%
Cardiac failure congestive	41	0.065%
Embolism	41	0.065%
Atrioventricular block complete	40	0.063%
Epilepsy	40	0.063%
Gastric ulcer	40	0.063%
Joint swelling	40	0.063%

Sjogren's syndrome	40	0.063%
Discomfort	39	0.062%
Nervous system disorder	39	0.062%
Pulmonary tuberculosis	39	0.062%
Swelling	39	0.062%
Inappropriate antidiuretic hormone secretion	39	0.062%
Atelectasis	38	0.060%
Cholestasis	38	0.060%
Diplopia	38	0.060%
Movement disorder	38	0.060%
Muscle spasms	38	0.060%
Oesophagitis	38	0.060%
Type 2 diabetes mellitus	38	0.060%
Oropharyngeal pain	38	0.060%
Amylase increased	37	0.058%
Guillain-Barre syndrome	37	0.058%
Lung infiltration	37	0.058%
Cognitive disorder	37	0.058%
Therapy partial responder	37	0.058%
Agranulocytosis	36	0.057%
Cardiomyopathy	36	0.057%
Intestinal obstruction	36	0.057%
Myopathy	36	0.057%
Neck pain	36	0.057%
Cancer pain	36	0.057%
Autoimmune colitis	36	0.057%
Blister	35	0.055%
Blood lactate dehydrogenase increased	35	0.055%
Gait inability	35	0.055%
Intestinal perforation	35	0.055%
Lipase increased	35	0.055%
Metastases to lymph nodes	35	0.055%
Immune-mediated adrenal insufficiency	35	0.055%
Eyelid ptosis	34	0.054%
Fracture	34	0.054%
Hypoglycaemia	34	0.054%
Large intestine perforation	34	0.054%
Urinary retention	34	0.054%
Autoimmune haemolytic anaemia	34	0.054%
Gastrointestinal disorder	33	0.052%
Haematuria	33	0.052%
Laboratory test abnormal	33	0.052%
Neoplasm malignant	33	0.052%
Optic neuritis	33	0.052%
Polymyalgia rheumatica	33	0.052%
Surgery	33	0.052%
Performance status decreased	33	0.052%
Immune-mediated renal disorder	33	0.052%
Abdominal discomfort	32	0.051%

Lung abscess	32	0.051%
Wheezing	32	0.051%
Balance disorder	32	0.051%
Ischaemic stroke	32	0.051%
Dysgeusia	31	0.049%
Emphysema	31	0.049%
Facial paralysis	31	0.049%
Gastrointestinal perforation	31	0.049%
Musculoskeletal stiffness	31	0.049%
Stress cardiomyopathy	31	0.049%
Taste disorder	31	0.049%
Blood pressure increased	30	0.047%
Bradycardia	30	0.047%
Cytomegalovirus infection	30	0.047%
Eosinophilia	30	0.047%
Oral candidiasis	30	0.047%
Metastasis	30	0.047%
Blood thyroid stimulating hormone decreased	29	0.046%
Feeling cold	29	0.046%
Flushing	29	0.046%
Metastases to lung	29	0.046%
Upper respiratory tract infection	29	0.046%
Immune-mediated arthritis	29	0.046%
Cardiac failure acute	28	0.044%
Cataract	28	0.044%
Disorientation	28	0.044%
Gastroenteritis	28	0.044%
Myelodysplastic syndrome	28	0.044%
Nephrotic syndrome	28	0.044%
Supraventricular tachycardia	28	0.044%
Thyroid function test abnormal	28	0.044%
Lung neoplasm malignant	28	0.044%
Immune-mediated encephalitis	28	0.044%
COVID-19 pneumonia	28	0.044%
Bronchopulmonary aspergillosis	27	0.043%
Dermatitis acneiform	27	0.043%
Dyspepsia	27	0.043%
Hyperhidrosis	27	0.043%
Hospitalisation	27	0.043%
Pulmonary mass	27	0.043%
Underdose	27	0.043%
Ill-defined disorder	27	0.043%
Hiccups	26	0.041%
Metastases to adrenals	26	0.041%
Nephropathy toxic	26	0.041%
Pollakiuria	26	0.041%
Shock	26	0.041%
Skin reaction	26	0.041%
Tuberculosis	26	0.041%

Tumour lysis syndrome	26	0.041%
Mental status changes	26	0.041%
Musculoskeletal chest pain	26	0.041%
Central nervous system lesion	26	0.041%
Eastern Cooperative Oncology Group performance status worsened	26	0.041%
Aphasia	25	0.039%
Aspiration pleural cavity	25	0.039%
Atrial flutter	25	0.039%
Cystitis	25	0.039%
Endocrine disorder	25	0.039%
Heart rate increased	25	0.039%
Polyneuropathy	25	0.039%
Uveitis	25	0.039%
Vasculitis	25	0.039%
Encephalitis autoimmune	25	0.039%
Immune-mediated myositis	25	0.039%
Haematemesis	24	0.038%
Hemiparesis	24	0.038%
Leukocytosis	24	0.038%
Pancreatitis acute	24	0.038%
Rash erythematous	24	0.038%
Transient ischaemic attack	24	0.038%
Vertigo	24	0.038%
Autoimmune thyroiditis	24	0.038%
Toxic skin eruption	24	0.038%
Aspiration	23	0.036%
Blood corticotrophin decreased	23	0.036%
Coma	23	0.036%
Dementia	23	0.036%
Eating disorder	23	0.036%
Hypocalcaemia	23	0.036%
Lethargy	23	0.036%
Liver function test abnormal	23	0.036%
Night sweats	23	0.036%
Rhinorrhoea	23	0.036%
Skin ulcer	23	0.036%
Ventricular fibrillation	23	0.036%
Tumour haemorrhage	23	0.036%
Staphylococcal infection	23	0.036%
Liver injury	23	0.036%
Abdominal distension	22	0.035%
Carcinoembryonic antigen increased	22	0.035%
Hallucination	22	0.035%
Paralysis	22	0.035%
Pericarditis malignant	22	0.035%
Sleep disorder	22	0.035%
Small intestinal perforation	22	0.035%
Spinal compression fracture	22	0.035%
Contusion	22	0.035%

Feeding disorder	22	0.035%
Device related infection	22	0.035%
Chronic kidney disease	22	0.035%
Silent thyroiditis	22	0.035%
Immune-mediated cholangitis	22	0.035%
Cardiogenic shock	21	0.033%
Circulatory collapse	21	0.033%
Cortisol decreased	21	0.033%
Femur fracture	21	0.033%
Metabolic acidosis	21	0.033%
Palpitations	21	0.033%
Psoriatic arthropathy	21	0.033%
Respiratory disorder	21	0.033%
Superior vena cava syndrome	21	0.033%
Therapeutic response unexpected	21	0.033%
Tumour pain	21	0.033%
Acute coronary syndrome	21	0.033%
Drug intolerance	21	0.033%
Aspergillus infection	21	0.033%
Immune-mediated thyroiditis	21	0.033%
Appendicitis	20	0.032%
Cachexia	20	0.032%
Cholecystitis acute	20	0.032%
Duodenal ulcer	20	0.032%
Gastrooesophageal reflux disease	20	0.032%
Neurotoxicity	20	0.032%
Pulmonary alveolar haemorrhage	20	0.032%
Rectal haemorrhage	20	0.032%
Ventricular tachycardia	20	0.032%
Cytomegalovirus enterocolitis	20	0.032%
Tumour associated fever	20	0.032%
Troponin increased	20	0.032%
General physical condition abnormal	20	0.032%
Adverse drug reaction	20	0.032%
Autoimmune myocarditis	20	0.032%
Rheumatic disorder	20	0.032%
Tumour hyperprogression	20	0.032%
Hypersensitivity pneumonitis	20	0.032%
Angina pectoris	19	0.030%
Deafness	19	0.030%
Dermatomyositis	19	0.030%
Drug hypersensitivity	19	0.030%
Face oedema	19	0.030%
Generalised oedema	19	0.030%
Mouth ulceration	19	0.030%
Peripheral motor neuropathy	19	0.030%
Polymyositis	19	0.030%
Thirst	19	0.030%
Therapy non-responder	19	0.030%

Bacterial infection	19	0.030%
Hypophagia	19	0.030%
Lung opacity	19	0.030%
Immune-mediated pancreatitis	19	0.030%
Amnesia	18	0.028%
Ataxia	18	0.028%
Blindness	18	0.028%
Clostridium difficile colitis	18	0.028%
Conjunctivitis	18	0.028%
Dysarthria	18	0.028%
Dysuria	18	0.028%
Osteomyelitis	18	0.028%
Rib fracture	18	0.028%
Skin discolouration	18	0.028%
Therapeutic response decreased	18	0.028%
Dermatitis psoriasiform	18	0.028%
Autoimmune disorder	18	0.028%
Adrenocortical insufficiency acute	17	0.027%
Blood sodium decreased	17	0.027%
Colon cancer	17	0.027%
Coronary artery disease	17	0.027%
Depressed mood	17	0.027%
Electrolyte imbalance	17	0.027%
Eye pain	17	0.027%
Haemoglobin abnormal	17	0.027%
Lacrimation increased	17	0.027%
Lichen planus	17	0.027%
Neuralgia	17	0.027%
Speech disorder	17	0.027%
Hepatic cytolysis	17	0.027%
Lower gastrointestinal haemorrhage	17	0.027%
Coronavirus infection	17	0.027%
Lichenoid keratosis	17	0.027%
Autoimmune pancreatitis	17	0.027%
Arthropathy	16	0.025%
Atrioventricular block	16	0.025%
Bacteraemia	16	0.025%
Blood potassium decreased	16	0.025%
Cardiomegaly	16	0.025%
Fungal infection	16	0.025%
Hyperthermia	16	0.025%
Hyperuricaemia	16	0.025%
Ileus paralytic	16	0.025%
Meningitis aseptic	16	0.025%
Osteonecrosis	16	0.025%
Pulmonary hypertension	16	0.025%
Sialoadenitis	16	0.025%
Sinus tachycardia	16	0.025%
Skin lesion	16	0.025%

Thoracic cavity drainage	16	0.025%
Gastrointestinal toxicity	16	0.025%
Immune-mediated nephritis	16	0.025%
Immune-mediated hyperthyroidism	16	0.025%
Aplastic anaemia	15	0.024%
Asphyxia	15	0.024%
Bladder cancer	15	0.024%
Bronchospasm	15	0.024%
Decreased activity	15	0.024%
Duodenitis	15	0.024%
Haemolytic anaemia	15	0.024%
Hypersomnia	15	0.024%
Hypomagnesaemia	15	0.024%
Immune system disorder	15	0.024%
Liver abscess	15	0.024%
Oesophageal stenosis	15	0.024%
Paronychia	15	0.024%
Peripheral ischaemia	15	0.024%
Purpura	15	0.024%
Rash macular	15	0.024%
Red blood cell count decreased	15	0.024%
Sinusitis	15	0.024%
Spinal fracture	15	0.024%
Swelling face	15	0.024%
Tinnitus	15	0.024%
Upper gastrointestinal haemorrhage	15	0.024%
Mobility decreased	15	0.024%
Fibrin D dimer increased	14	0.022%
Frequent bowel movements	14	0.022%
Gastric haemorrhage	14	0.022%
Hepatitis acute	14	0.022%
Hepatocellular injury	14	0.022%
Hip fracture	14	0.022%
Lung adenocarcinoma	14	0.022%
Ocular hyperaemia	14	0.022%
Palmar-plantar erythrodysaesthesia syndrome	14	0.022%
Pathological fracture	14	0.022%
Pleurodesis	14	0.022%
Skin infection	14	0.022%
Small intestinal obstruction	14	0.022%
Radiation oesophagitis	14	0.022%
Systemic inflammatory response syndrome	14	0.022%
Clostridium difficile infection	14	0.022%
Enteritis infectious	14	0.022%
Non-small cell lung cancer metastatic	14	0.022%
Drug resistance	14	0.022%
Aortic arteriosclerosis	14	0.022%
Hospice care	14	0.022%
Illness	14	0.022%

Body temperature increased	13	0.021%
Burning sensation	13	0.021%
Cholelithiasis	13	0.021%
Dermatitis bullous	13	0.021%
Dermatitis exfoliative generalised	13	0.021%
Drug interaction	13	0.021%
Dry eye	13	0.021%
Eosinophilic pneumonia	13	0.021%
Failure to thrive	13	0.021%
Femoral neck fracture	13	0.021%
Gastric cancer	13	0.021%
Hydronephrosis	13	0.021%
IgA nephropathy	13	0.021%
Increased appetite	13	0.021%
Lymphocyte count abnormal	13	0.021%
Myasthenic syndrome	13	0.021%
Overdose	13	0.021%
Pharyngitis	13	0.021%
Platelet transfusion	13	0.021%
Presyncope	13	0.021%
Pseudomembranous colitis	13	0.021%
Pulmonary congestion	13	0.021%
Restlessness	13	0.021%
Systemic lupus erythematosus	13	0.021%
Visual acuity reduced	13	0.021%
Dysstasia	13	0.021%
Intervertebral disc protrusion	13	0.021%
Cytokine storm	13	0.021%
Pulmonary sepsis	13	0.021%
Secretion discharge	13	0.021%
Tumour necrosis	13	0.021%
Neutrophil count abnormal	13	0.021%
Limbic encephalitis	13	0.021%
Acute myeloid leukaemia	12	0.019%
Aortic dissection	12	0.019%
Aplasia pure red cell	12	0.019%
Blood albumin decreased	12	0.019%
Blood potassium increased	12	0.019%
Blood thyroid stimulating hormone abnormal	12	0.019%
Bronchial obstruction	12	0.019%
Cerebral ischaemia	12	0.019%
Cholangitis acute	12	0.019%
Cystitis haemorrhagic	12	0.019%
Deafness neurosensory	12	0.019%
Diabetes mellitus inadequate control	12	0.019%
Endocarditis	12	0.019%
Feeling hot	12	0.019%
Haemorrhoids	12	0.019%
Lung consolidation	12	0.019%

Myocardial ischaemia	12	0.019%
Neutrophil count increased	12	0.019%
Recurrent cancer	12	0.019%
Spinal osteoarthritis	12	0.019%
Hydrothorax	12	0.019%
Urosepsis	12	0.019%
Physical deconditioning	12	0.019%
Hot flush	12	0.019%
Brain neoplasm	12	0.019%
Haematotoxicity	12	0.019%
Malnutrition	12	0.019%
Venous thrombosis limb	12	0.019%
Incorrect dose administered	12	0.019%
Osteonecrosis of jaw	12	0.019%
Loss of personal independence in daily activities	12	0.019%
Immune-mediated gastritis	12	0.019%
Immune-mediated hypophysitis	12	0.019%
Acidosis	11	0.017%
Arteriosclerosis	11	0.017%
Blood urea increased	11	0.017%
Breast cancer	11	0.017%
Bronchiectasis	11	0.017%
Colitis ischaemic	11	0.017%
Decubitus ulcer	11	0.017%
Diabetes insipidus	11	0.017%
Disturbance in attention	11	0.017%
Eye disorder	11	0.017%
Haematoma	11	0.017%
Incontinence	11	0.017%
Ketoacidosis	11	0.017%
Lymphopenia	11	0.017%
Mass	11	0.017%
Metastases to spine	11	0.017%
Nephrolithiasis	11	0.017%
Oesophageal candidiasis	11	0.017%
Ophthalmoplegia	11	0.017%
Osteoarthritis	11	0.017%
Pallor	11	0.017%
Platelet disorder	11	0.017%
Pneumothorax spontaneous	11	0.017%
Retroperitoneal fibrosis	11	0.017%
Spinal cord compression	11	0.017%
Squamous cell carcinoma	11	0.017%
Subdural haematoma	11	0.017%
Tachypnoea	11	0.017%
Urinary incontinence	11	0.017%
Vitiligo	11	0.017%
Neutropenic sepsis	11	0.017%
Lumbar vertebral fracture	11	0.017%

Ejection fraction decreased	11	0.017%
Tracheal stenosis	11	0.017%
Cerebral disorder	11	0.017%
Oesophagobronchial fistula	11	0.017%
Blood test abnormal	11	0.017%
White blood cell disorder	11	0.017%
Atypical mycobacterial infection	11	0.017%
Lymphocytic hypophysitis	11	0.017%
Radiation interaction	11	0.017%
Myocardial necrosis marker increased	11	0.017%
Sinus node dysfunction	11	0.017%
Immune-mediated neuropathy	11	0.017%
Cardiac dysfunction	11	0.017%
Therapeutic product effect incomplete	11	0.017%
SARS-CoV-2 test positive	11	0.017%
Acute hepatic failure	10	0.016%
Addison's disease	10	0.016%
Ageusia	10	0.016%
Aspartate aminotransferase abnormal	10	0.016%
Blood calcium increased	10	0.016%
Bronchial fistula	10	0.016%
Bronchitis chronic	10	0.016%
Cardiovascular disorder	10	0.016%
Cheilitis	10	0.016%
Chronic gastritis	10	0.016%
Dizziness postural	10	0.016%
Duodenal ulcer haemorrhage	10	0.016%
Embolism venous	10	0.016%
Gastritis haemorrhagic	10	0.016%
Gastrointestinal necrosis	10	0.016%
Hepatic steatosis	10	0.016%
Hepatitis fulminant	10	0.016%
Hypophosphataemia	10	0.016%
Myelitis	10	0.016%
Odynophagia	10	0.016%
Pemphigus	10	0.016%
Pericardial drainage	10	0.016%
Proctitis	10	0.016%
Radiculopathy	10	0.016%
Rash pustular	10	0.016%
Renal tubular necrosis	10	0.016%
Thrombotic thrombocytopenic purpura	10	0.016%
Thyrotoxic crisis	10	0.016%
Musculoskeletal disorder	10	0.016%
Bile duct stenosis	10	0.016%
Bronchopleural fistula	10	0.016%
Neurological symptom	10	0.016%
Obstructive airways disorder	10	0.016%
Hepatobiliary disease	10	0.016%

Therapy cessation	10	0.016%
Hypertransaminasaemia	10	0.016%
Spinal pain	10	0.016%
Prerenal failure	10	0.016%
Candida infection	10	0.016%
Autoimmune nephritis	10	0.016%
Vogt-Koyanagi-Harada disease	10	0.016%
Central hypothyroidism	10	0.016%
Red blood cell transfusion	10	0.016%
Agitation	9	0.014%
Alanine aminotransferase abnormal	9	0.014%
Angioedema	9	0.014%
Bile duct stone	9	0.014%
Duodenal perforation	9	0.014%
Empyema	9	0.014%
Enterocolitis haemorrhagic	9	0.014%
Eosinophilic fasciitis	9	0.014%
Giant cell arteritis	9	0.014%
Glomerulonephritis membranous	9	0.014%
Gynaecomastia	9	0.014%
Haemorrhage intracranial	9	0.014%
Heart rate decreased	9	0.014%
Henoch-Schonlein purpura	9	0.014%
Hepatitis C	9	0.014%
Hyperbilirubinaemia	9	0.014%
Hypertensive crisis	9	0.014%
Hypokinesia	9	0.014%
Lip swelling	9	0.014%
Lymphadenopathy mediastinal	9	0.014%
Oral pain	9	0.014%
Oxygen saturation abnormal	9	0.014%
Panniculitis	9	0.014%
Paralysis recurrent laryngeal nerve	9	0.014%
Petechiae	9	0.014%
Pyelonephritis	9	0.014%
Respiratory arrest	9	0.014%
Sarcoidosis	9	0.014%
Scleroderma	9	0.014%
Splenic infarction	9	0.014%
Stress	9	0.014%
Systemic candida	9	0.014%
Venous thrombosis	9	0.014%
Hypoacusis	9	0.014%
Bedridden	9	0.014%
Lung cancer metastatic	9	0.014%
Cardiopulmonary failure	9	0.014%
Blood alkaline phosphatase abnormal	9	0.014%
Sputum retention	9	0.014%
Mental disorder	9	0.014%

Metastatic neoplasm	9	0.014%
Motor dysfunction	9	0.014%
Immunodeficiency	9	0.014%
Diverticular perforation	9	0.014%
Lung neoplasm	9	0.014%
Pigmentation disorder	9	0.014%
Oral mucosa erosion	9	0.014%
Oesophageal fistula	9	0.014%
Staphylococcus test positive	9	0.014%
Posterior reversible encephalopathy syndrome	9	0.014%
Intestinal metastasis	9	0.014%
Steroid diabetes	9	0.014%
Congestive hepatopathy	9	0.014%
Immune-mediated encephalopathy	9	0.014%
Abscess	8	0.013%
Basal cell carcinoma	8	0.013%
Benign prostatic hyperplasia	8	0.013%
Bronchiolitis	8	0.013%
Chromaturia	8	0.013%
Coagulopathy	8	0.013%
Diverticulum intestinal haemorrhagic	8	0.013%
Embolic stroke	8	0.013%
Erysipelas	8	0.013%
Fluid retention	8	0.013%
Folliculitis	8	0.013%
Glaucoma	8	0.013%
Gout	8	0.013%
Blood urine present	8	0.013%
Haemolysis	8	0.013%
Hair colour changes	8	0.013%
Heart rate irregular	8	0.013%
Hepatic cirrhosis	8	0.013%
Hepatic cyst	8	0.013%
Hepatitis toxic	8	0.013%
Hepatomegaly	8	0.013%
Hypercapnia	8	0.013%
Hypernatraemia	8	0.013%
Hyperpyrexia	8	0.013%
Hypovolaemic shock	8	0.013%
Idiopathic pulmonary fibrosis	8	0.013%
Intestinal ischaemia	8	0.013%
Irritability	8	0.013%
Lymphoedema	8	0.013%
Lymphoma	8	0.013%
Metastases to pleura	8	0.013%
Multiple sclerosis	8	0.013%
Nasal congestion	8	0.013%
Pain of skin	8	0.013%
Pancreatitis chronic	8	0.013%

Pulmonary artery thrombosis	8	0.013%
Radiotherapy	8	0.013%
Rash vesicular	8	0.013%
Road traffic accident	8	0.013%
Sciatica	8	0.013%
Skin erosion	8	0.013%
Sputum discoloured	8	0.013%
Synovitis	8	0.013%
Viral infection	8	0.013%
Vocal cord paralysis	8	0.013%
Tumour marker increased	8	0.013%
Pericardial effusion malignant	8	0.013%
Localised oedema	8	0.013%
Lacunar infarction	8	0.013%
Cytomegalovirus test positive	8	0.013%
Febrile infection	8	0.013%
Thyroxine free abnormal	8	0.013%
Staphylococcal sepsis	8	0.013%
Subacute cutaneous lupus erythematosus	8	0.013%
Troponin I increased	8	0.013%
Pulmonary necrosis	8	0.013%
Angiopathy	8	0.013%
Diffuse alveolar damage	8	0.013%
Appetite disorder	8	0.013%
Biliary tract disorder	8	0.013%
Limb discomfort	8	0.013%
Sudden hearing loss	8	0.013%
Renal function test abnormal	8	0.013%
Lower limb fracture	8	0.013%
Biliary tract infection	8	0.013%
Seronegative arthritis	8	0.013%
Oral herpes	8	0.013%
Radiation necrosis	8	0.013%
Lung cyst	8	0.013%
Paraneoplastic neurological syndrome	8	0.013%
Pancreatic failure	8	0.013%
Large intestine infection	8	0.013%
Immune-mediated cystitis	8	0.013%
Alveolitis	7	0.011%
Anuria	7	0.011%
Aphonia	7	0.011%
Appendicitis perforated	7	0.011%
Arteriosclerosis coronary artery	7	0.011%
Basedow's disease	7	0.011%
Blood calcium decreased	7	0.011%
Blood glucose decreased	7	0.011%
Bone disorder	7	0.011%
Cardiac pacemaker insertion	7	0.011%
Cerebral atrophy	7	0.011%

Chronic myeloid leukaemia	7	0.011%
Chronic respiratory failure	7	0.011%
Completed suicide	7	0.011%
Cyanosis	7	0.011%
Ear pain	7	0.011%
Faeces discoloured	7	0.011%
Gastric ulcer haemorrhage	7	0.011%
Glomerular filtration rate decreased	7	0.011%
Glomerulonephritis rapidly progressive	7	0.011%
Granulocytopenia	7	0.011%
Haemothorax	7	0.011%
Hemiplegia	7	0.011%
Hepatitis E	7	0.011%
Hiatus hernia	7	0.011%
Humerus fracture	7	0.011%
Hypertriglyceridaemia	7	0.011%
Impaired healing	7	0.011%
Injury	7	0.011%
Jaundice cholestatic	7	0.011%
Joint stiffness	7	0.011%
Large intestinal ulcer	7	0.011%
Lung carcinoma cell type unspecified recurrent	7	0.011%
Lymphadenitis	7	0.011%
Lymphangitis	7	0.011%
Mastitis	7	0.011%
Myelitis transverse	7	0.011%
Opportunistic infection	7	0.011%
Oral discomfort	7	0.011%
Orthostatic hypotension	7	0.011%
Osteoporosis	7	0.011%
Pancreatic atrophy	7	0.011%
Pancreatic disorder	7	0.011%
Paraplegia	7	0.011%
Parotitis	7	0.011%
Pelvic pain	7	0.011%
Photosensitivity reaction	7	0.011%
Pneumonia viral	7	0.011%
Postoperative wound infection	7	0.011%
Rash papular	7	0.011%
Renal cyst	7	0.011%
Retinal detachment	7	0.011%
Retinopathy	7	0.011%
Rhinitis	7	0.011%
Right ventricular failure	7	0.011%
Subarachnoid haemorrhage	7	0.011%
Thrombophlebitis	7	0.011%
Stent placement	7	0.011%
Cardiotoxicity	7	0.011%
Lupus-like syndrome	7	0.011%

Escherichia urinary tract infection	7	0.011%
Primary adrenal insufficiency	7	0.011%
Small intestinal haemorrhage	7	0.011%
Central venous catheterisation	7	0.011%
Haemorrhoidal haemorrhage	7	0.011%
Autoimmune neutropenia	7	0.011%
Pneumatosis intestinalis	7	0.011%
Biliary dilatation	7	0.011%
Troponin T increased	7	0.011%
Enterocolitis infectious	7	0.011%
Paraneoplastic syndrome	7	0.011%
Urine output decreased	7	0.011%
Prostate cancer	7	0.011%
Bladder disorder	7	0.011%
Escherichia infection	7	0.011%
Gastrointestinal motility disorder	7	0.011%
Spinal disorder	7	0.011%
Abnormal behaviour	7	0.011%
Pseudomonas infection	7	0.011%
Urinary tract obstruction	7	0.011%
Demyelinating polyneuropathy	7	0.011%
Peripheral arterial occlusive disease	7	0.011%
Hepatic enzyme abnormal	7	0.011%
Myasthenia gravis crisis	7	0.011%
Circumstance or information capable of leading to medication error	7	0.011%
Breath sounds abnormal	7	0.011%
Bronchial disorder	7	0.011%
Enterocolitis bacterial	7	0.011%
Bronchial haemorrhage	7	0.011%
Treatment failure	7	0.011%
Skin mass	7	0.011%
Neurological decompensation	7	0.011%
Creatinine renal clearance abnormal	7	0.011%
Autoimmune arthritis	7	0.011%
Drug reaction with eosinophilia and systemic symptoms	7	0.011%
Wrong technique in product usage process	7	0.011%
Autoimmune hypothyroidism	7	0.011%
Product storage error	7	0.011%
Vascular device infection	7	0.011%
Abdominal pain lower	6	0.009%
Atypical pneumonia	6	0.009%
Back disorder	6	0.009%
Blood chloride decreased	6	0.009%
Blood creatinine abnormal	6	0.009%
Blood glucose abnormal	6	0.009%
Blood sodium abnormal	6	0.009%
Blood uric acid increased	6	0.009%
Brain abscess	6	0.009%
Breast pain	6	0.009%

Bronchostenosis	6	0.009%
Bursitis	6	0.009%
Capillary leak syndrome	6	0.009%
Chest X-ray abnormal	6	0.009%
Clavicle fracture	6	0.009%
Creatinine renal clearance decreased	6	0.009%
Cystitis interstitial	6	0.009%
Dyskinesia	6	0.009%
Dyslalia	6	0.009%
Eye irritation	6	0.009%
Eyelid oedema	6	0.009%
Fistula	6	0.009%
Flatulence	6	0.009%
Full blood count abnormal	6	0.009%
Gastrointestinal infection	6	0.009%
Gingival pain	6	0.009%
Glycosylated haemoglobin increased	6	0.009%
Goitre	6	0.009%
Granuloma	6	0.009%
Hallucination, visual	6	0.009%
Head injury	6	0.009%
Hepatitis B	6	0.009%
Herpes simplex	6	0.009%
Hydrocephalus	6	0.009%
Hypotonia	6	0.009%
Hypovolaemia	6	0.009%
Inguinal hernia	6	0.009%
Intracranial pressure increased	6	0.009%
Iron deficiency anaemia	6	0.009%
Lactic acidosis	6	0.009%
Localised infection	6	0.009%
Lymphocyte count increased	6	0.009%
Metastases to kidney	6	0.009%
Monoparesis	6	0.009%
Monoplegia	6	0.009%
Muscle atrophy	6	0.009%
Nephropathy	6	0.009%
Nocturia	6	0.009%
Non-small cell lung cancer stage IV	6	0.009%
Oesophageal carcinoma	6	0.009%
Oesophageal perforation	6	0.009%
Optic ischaemic neuropathy	6	0.009%
Osteolysis	6	0.009%
Pain in jaw	6	0.009%
Phlebitis	6	0.009%
Pneumonitis chemical	6	0.009%
Renal tubular disorder	6	0.009%
Respiratory acidosis	6	0.009%
Respiratory depression	6	0.009%

Scar	6	0.009%
Second primary malignancy	6	0.009%
Sensory disturbance	6	0.009%
Sneezing	6	0.009%
Squamous cell carcinoma of skin	6	0.009%
Swollen tongue	6	0.009%
Throat irritation	6	0.009%
Thrombotic microangiopathy	6	0.009%
Tooth abscess	6	0.009%
Tracheo-oesophageal fistula	6	0.009%
Transplant rejection	6	0.009%
Unresponsive to stimuli	6	0.009%
Energy increased	6	0.009%
Cytomegalovirus colitis	6	0.009%
Tachyarrhythmia	6	0.009%
Left ventricular dysfunction	6	0.009%
Pneumomediastinum	6	0.009%
Subileus	6	0.009%
Chest tube insertion	6	0.009%
Anti-neutrophil cytoplasmic antibody positive vasculitis	6	0.009%
Staphylococcal bacteraemia	6	0.009%
Mechanical ileus	6	0.009%
Prostatomegaly	6	0.009%
Epidermolysis	6	0.009%
Febrile bone marrow aplasia	6	0.009%
Pachymeningitis	6	0.009%
Nodule	6	0.009%
Gastric disorder	6	0.009%
Infective exacerbation of chronic obstructive airways disease	6	0.009%
Computerised tomogram thorax abnormal	6	0.009%
Gastrointestinal oedema	6	0.009%
Serositis	6	0.009%
Limb injury	6	0.009%
Oral fungal infection	6	0.009%
Parkinson's disease	6	0.009%
Blood disorder	6	0.009%
Diabetic nephropathy	6	0.009%
Adrenalitis	6	0.009%
Cholecystitis infective	6	0.009%
Cell marker increased	6	0.009%
Exfoliative rash	6	0.009%
Infusion site extravasation	6	0.009%
Malignant transformation	6	0.009%
Cardiovascular insufficiency	6	0.009%
Oral disorder	6	0.009%
Acute lung injury	6	0.009%
Substance-induced psychotic disorder	6	0.009%
Noninfective encephalitis	6	0.009%
Gastrointestinal wall thickening	6	0.009%

Immune-mediated endocrinopathy	6	0.009%
Tumour cavitation	6	0.009%
Incorrect product administration duration	6	0.009%
Biliary obstruction	6	0.009%
Suspected COVID-19	6	0.009%
Immune-mediated cytopenia	6	0.009%
Acquired tracheo-oesophageal fistula	5	0.008%
Adenocarcinoma	5	0.008%
Affective disorder	5	0.008%
Aggression	5	0.008%
Aortic aneurysm	5	0.008%
Blood creatine increased	5	0.008%
Blood magnesium decreased	5	0.008%
Bundle branch block left	5	0.008%
Bundle branch block right	5	0.008%
Cardiac failure chronic	5	0.008%
Carotid artery stenosis	5	0.008%
Carpal tunnel syndrome	5	0.008%
Cerebral thrombosis	5	0.008%
Cerebrovascular disorder	5	0.008%
Cutaneous vasculitis	5	0.008%
Dermatitis exfoliative	5	0.008%
Diverticulum	5	0.008%
Diverticulum intestinal	5	0.008%
Dyspnoea at rest	5	0.008%
Ear disorder	5	0.008%
Electrocardiogram QT prolonged	5	0.008%
Escherichia sepsis	5	0.008%
Extravasation	5	0.008%
Eye discharge	5	0.008%
Full blood count decreased	5	0.008%
Gallbladder disorder	5	0.008%
Gastric perforation	5	0.008%
Gastritis erosive	5	0.008%
Gastrointestinal pain	5	0.008%
Gingivitis	5	0.008%
Glomerulonephritis	5	0.008%
Groin pain	5	0.008%
Haemorrhagic stroke	5	0.008%
Hyperaesthesia	5	0.008%
Hypoparathyroidism	5	0.008%
Hypothermia	5	0.008%
Jejunal perforation	5	0.008%
Joint dislocation	5	0.008%
Kidney infection	5	0.008%
Mental impairment	5	0.008%
Micturition urgency	5	0.008%
Muscle disorder	5	0.008%
Nail discolouration	5	0.008%

Necrosis	5	0.008%
Neurogenic bladder	5	0.008%
Oesophageal ulcer	5	0.008%
Oliguria	5	0.008%
Oral mucosal blistering	5	0.008%
Oral mucosal eruption	5	0.008%
Papilloedema	5	0.008%
Papule	5	0.008%
Paresis	5	0.008%
Peripheral coldness	5	0.008%
Photophobia	5	0.008%
Pneumonia cytomegaloviral	5	0.008%
Pneumonia staphylococcal	5	0.008%
Portal vein thrombosis	5	0.008%
Primary hypothyroidism	5	0.008%
Prostatitis	5	0.008%
Pulmonary thrombosis	5	0.008%
Pustular psoriasis	5	0.008%
Radiation fibrosis - lung	5	0.008%
Retching	5	0.008%
Scab	5	0.008%
Scratch	5	0.008%
Serous retinal detachment	5	0.008%
Sinus bradycardia	5	0.008%
Sinus congestion	5	0.008%
Small cell lung cancer	5	0.008%
Small intestine ulcer	5	0.008%
Subcutaneous emphysema	5	0.008%
Tendonitis	5	0.008%
Thrombotic stroke	5	0.008%
Tooth disorder	5	0.008%
Toxic encephalopathy	5	0.008%
Transitional cell carcinoma	5	0.008%
Tumour embolism	5	0.008%
Ventricular extrasystoles	5	0.008%
Viral upper respiratory tract infection	5	0.008%
Vitreous floaters	5	0.008%
Wound infection	5	0.008%
Anal abscess	5	0.008%
Ocular myasthenia	5	0.008%
Thoracic vertebral fracture	5	0.008%
Pulmonary cavitation	5	0.008%
Enterobacter infection	5	0.008%
Wound	5	0.008%
Deafness bilateral	5	0.008%
Musculoskeletal discomfort	5	0.008%
Tri-iodothyronine free decreased	5	0.008%
Fluid intake reduced	5	0.008%
Therapeutic aspiration	5	0.008%

Colitis microscopic	5	0.008%
Hypoaesthesia oral	5	0.008%
Paraesthesia oral	5	0.008%
Post procedural complication	5	0.008%
Blood bilirubin abnormal	5	0.008%
Skin laceration	5	0.008%
Protein-losing gastroenteropathy	5	0.008%
Postrenal failure	5	0.008%
Dialysis	5	0.008%
Eye movement disorder	5	0.008%
Pelvic fracture	5	0.008%
Gastric mucosal lesion	5	0.008%
Mediastinal disorder	5	0.008%
Upper limb fracture	5	0.008%
Pulmonary function test abnormal	5	0.008%
Bone lesion	5	0.008%
Neoplasm recurrence	5	0.008%
Pancreatic enzymes increased	5	0.008%
Psychotic disorder	5	0.008%
Sinus disorder	5	0.008%
Non-cardiac chest pain	5	0.008%
Poor quality sleep	5	0.008%
Arterial occlusive disease	5	0.008%
Effusion	5	0.008%
Thyroid hormones increased	5	0.008%
Endotracheal intubation	5	0.008%
Brain injury	5	0.008%
Tumour compression	5	0.008%
C-reactive protein abnormal	5	0.008%
Traumatic lung injury	5	0.008%
Acquired gene mutation	5	0.008%
EGFR gene mutation	5	0.008%
Peripheral artery thrombosis	5	0.008%
Adrenal gland cancer	5	0.008%
Pulmonary pain	5	0.008%
Intentional product misuse	5	0.008%
Stenosis	5	0.008%
Anal incontinence	5	0.008%
Systemic scleroderma	5	0.008%
Oxygen therapy	5	0.008%
Central nervous system vasculitis	5	0.008%
Spinal stenosis	5	0.008%
Immune-mediated uveitis	5	0.008%
Abscess drainage	4	0.006%
Acute abdomen	4	0.006%
Acute pulmonary oedema	4	0.006%
Anorectal disorder	4	0.006%
Antiphospholipid syndrome	4	0.006%
Aortic stenosis	4	0.006%

Apathy	4	0.006%
Aptyalism	4	0.006%
Arterial thrombosis	4	0.006%
Atrial tachycardia	4	0.006%
Biliary colic	4	0.006%
Blood cholesterol increased	4	0.006%
Blood creatinine decreased	4	0.006%
Blood lactic acid increased	4	0.006%
Blood potassium abnormal	4	0.006%
Cerebellar ataxia	4	0.006%
Cerebellar infarction	4	0.006%
Cerebellar syndrome	4	0.006%
Choking	4	0.006%
Cold type haemolytic anaemia	4	0.006%
Compression fracture	4	0.006%
Computerised tomogram abnormal	4	0.006%
Cortisol abnormal	4	0.006%
Craniotomy	4	0.006%
Crohn's disease	4	0.006%
Crying	4	0.006%
Demyelination	4	0.006%
Dermatitis contact	4	0.006%
Diabetic coma	4	0.006%
Diarrhoea haemorrhagic	4	0.006%
Eczema asteatotic	4	0.006%
Enanthema	4	0.006%
Eructation	4	0.006%
Erythema nodosum	4	0.006%
Erythema of eyelid	4	0.006%
Exophthalmos	4	0.006%
Eye haemorrhage	4	0.006%
Eye inflammation	4	0.006%
Facial pain	4	0.006%
Fear	4	0.006%
Fungal skin infection	4	0.006%
Gastroenteritis viral	4	0.006%
Generalised tonic-clonic seizure	4	0.006%
Gingival swelling	4	0.006%
Glossitis	4	0.006%
Goodpasture's syndrome	4	0.006%
Haemorrhage subcutaneous	4	0.006%
Hair texture abnormal	4	0.006%
Hepatic encephalopathy	4	0.006%
Hernia	4	0.006%
Hypersensitivity vasculitis	4	0.006%
Hypertrichosis	4	0.006%
International normalised ratio increased	4	0.006%
Intracranial aneurysm	4	0.006%
Iron deficiency	4	0.006%

Joint effusion	4	0.006%
Keratitis	4	0.006%
Labile blood pressure	4	0.006%
Leukaemia	4	0.006%
Leukoencephalopathy	4	0.006%
Ligament sprain	4	0.006%
Lung adenocarcinoma recurrent	4	0.006%
Macular degeneration	4	0.006%
Malignant ascites	4	0.006%
Megacolon	4	0.006%
Mesenteric artery thrombosis	4	0.006%
Metastases to skin	4	0.006%
Mood altered	4	0.006%
Mood swings	4	0.006%
Nail disorder	4	0.006%
Nerve compression	4	0.006%
Nervousness	4	0.006%
Neuritis	4	0.006%
Neurodermatitis	4	0.006%
Normocytic anaemia	4	0.006%
Oculomucocutaneous syndrome	4	0.006%
Otitis media	4	0.006%
Pancreatic carcinoma	4	0.006%
Panic attack	4	0.006%
Parkinsonism	4	0.006%
Parosmia	4	0.006%
Pericardial excision	4	0.006%
Pericarditis constrictive	4	0.006%
Plasmapheresis	4	0.006%
Pneumonia klebsiella	4	0.006%
Pneumonia pneumococcal	4	0.006%
Pre-existing condition improved	4	0.006%
Progressive multifocal leukoencephalopathy	4	0.006%
Radiation injury	4	0.006%
Raynaud's phenomenon	4	0.006%
Rectal ulcer	4	0.006%
Renal haemorrhage	4	0.006%
Renal infarct	4	0.006%
Renal pain	4	0.006%
Retinal artery occlusion	4	0.006%
Retinal haemorrhage	4	0.006%
Retinal vasculitis	4	0.006%
Rotator cuff syndrome	4	0.006%
Sensory loss	4	0.006%
Serum ferritin increased	4	0.006%
Skin irritation	4	0.006%
Status epilepticus	4	0.006%
Stridor	4	0.006%
Thrombocytosis	4	0.006%

Thyroiditis subacute	4	0.006%
Toothache	4	0.006%
Tri-iodothyronine decreased	4	0.006%
VIth nerve paralysis	4	0.006%
White blood cell count abnormal	4	0.006%
Lip disorder	4	0.006%
Atrial thrombosis	4	0.006%
Poor venous access	4	0.006%
Deafness unilateral	4	0.006%
Haematocrit abnormal	4	0.006%
Treatment noncompliance	4	0.006%
Subclavian vein thrombosis	4	0.006%
Miller Fisher syndrome	4	0.006%
Upper respiratory tract inflammation	4	0.006%
Metastases to muscle	4	0.006%
Shock haemorrhagic	4	0.006%
Hypophonesis	4	0.006%
Platelet count increased	4	0.006%
Metastases to chest wall	4	0.006%
Ocular discomfort	4	0.006%
Eosinophil percentage increased	4	0.006%
Respiratory tract congestion	4	0.006%
Lymphatic disorder	4	0.006%
Large intestinal haemorrhage	4	0.006%
Gastrointestinal tube insertion	4	0.006%
Protein urine present	4	0.006%
Organ failure	4	0.006%
Adrenal mass	4	0.006%
Brain natriuretic peptide increased	4	0.006%
Urinary tract infection bacterial	4	0.006%
Vanishing bile duct syndrome	4	0.006%
Thyroxine free increased	4	0.006%
Faecaloma	4	0.006%
Tracheomalacia	4	0.006%
Temperature intolerance	4	0.006%
Procedural complication	4	0.006%
Mycobacterium avium complex infection	4	0.006%
Hepatitis B reactivation	4	0.006%
Bicytopenia	4	0.006%
Intestinal haemorrhage	4	0.006%
Extremity necrosis	4	0.006%
Embolic cerebral infarction	4	0.006%
Ischaemia	4	0.006%
Optic neuropathy	4	0.006%
Peripheral embolism	4	0.006%
Pneumonia fungal	4	0.006%
Facial nerve disorder	4	0.006%
Erectile dysfunction	4	0.006%
Pulmonary radiation injury	4	0.006%

Renal injury	4	0.006%
Hepatic lesion	4	0.006%
Large intestinal obstruction	4	0.006%
Endocarditis noninfective	4	0.006%
Gallbladder enlargement	4	0.006%
Hyperamylasaemia	4	0.006%
Acute left ventricular failure	4	0.006%
Hyperglycaemic hyperosmolar nonketotic syndrome	4	0.006%
Incorrect drug administration rate	4	0.006%
Procedural pain	4	0.006%
Phrenic nerve paralysis	4	0.006%
Tracheal fistula	4	0.006%
Mixed liver injury	4	0.006%
Superinfection bacterial	4	0.006%
Contrast media allergy	4	0.006%
Procalcitonin increased	4	0.006%
Vasogenic cerebral oedema	4	0.006%
Heat illness	4	0.006%
Respiratory tract inflammation	4	0.006%
Influenza A virus test positive	4	0.006%
Coronavirus test positive	4	0.006%
Glucocorticoid deficiency	4	0.006%
Faeces soft	4	0.006%
Cutaneous symptom	4	0.006%
Autoimmune dermatitis	4	0.006%
Complication associated with device	4	0.006%
Critical illness	4	0.006%
Insurance issue	4	0.006%
Lower gastrointestinal perforation	4	0.006%
Autoimmune thyroid disorder	4	0.006%
Product prescribing issue	4	0.006%
Mucosal disorder	4	0.006%
Biliary catheter insertion	4	0.006%
Loss of therapeutic response	4	0.006%
Myocardial injury	4	0.006%
Abdominal tenderness	3	0.005%
Abnormal faeces	3	0.005%
Accident	3	0.005%
Accidental overdose	3	0.005%
Acne	3	0.005%
Activated partial thromboplastin time prolonged	3	0.005%
Acute sinusitis	3	0.005%
Adrenal adenoma	3	0.005%
Adrenal haemorrhage	3	0.005%
Anger	3	0.005%
Angina unstable	3	0.005%
Angular cheilitis	3	0.005%
Anosmia	3	0.005%
Aortic valve incompetence	3	0.005%

Arthritis reactive	3	0.005%
Atrioventricular block second degree	3	0.005%
Azotaemia	3	0.005%
Blood corticotrophin abnormal	3	0.005%
Blood creatine phosphokinase MB increased	3	0.005%
Blood pressure abnormal	3	0.005%
Blood sodium increased	3	0.005%
Blood triglycerides increased	3	0.005%
Brain stem haemorrhage	3	0.005%
Cardiac hypertrophy	3	0.005%
Catheterisation cardiac	3	0.005%
Cerebellar haemorrhage	3	0.005%
Cerebral artery embolism	3	0.005%
Coeliac disease	3	0.005%
Cold sweat	3	0.005%
Coma hepatic	3	0.005%
Conduction disorder	3	0.005%
Conjunctival haemorrhage	3	0.005%
Coordination abnormal	3	0.005%
Coronary artery occlusion	3	0.005%
Coronary artery stenosis	3	0.005%
Cranial nerve palsies multiple	3	0.005%
Cushing's syndrome	3	0.005%
Cutaneous sarcoidosis	3	0.005%
Cytomegalovirus hepatitis	3	0.005%
Diabetic ketoacidotic hyperglycaemic coma	3	0.005%
Diabetic ketosis	3	0.005%
Diabetic neuropathy	3	0.005%
Diffuse large B-cell lymphoma	3	0.005%
Dysaesthesia	3	0.005%
Electrocardiogram ST segment elevation	3	0.005%
Embolism arterial	3	0.005%
Emotional disorder	3	0.005%
Epididymitis	3	0.005%
Epstein-Barr virus infection	3	0.005%
Euphoric mood	3	0.005%
Extrasystoles	3	0.005%
Eye swelling	3	0.005%
Feeling jittery	3	0.005%
Flank pain	3	0.005%
Galactorrhoea	3	0.005%
Gangrene	3	0.005%
Gastric ulcer perforation	3	0.005%
Gastrointestinal carcinoma	3	0.005%
Haemoglobin increased	3	0.005%
Haemorrhagic disorder	3	0.005%
Hair growth abnormal	3	0.005%
Heart rate abnormal	3	0.005%
Hemianopia	3	0.005%

Hemianopia homonymous	3	0.005%
Hepatic haemorrhage	3	0.005%
Hepatic pain	3	0.005%
Hepatitis cholestatic	3	0.005%
Hepatitis viral	3	0.005%
Hunger	3	0.005%
Hyperplastic cholecystopathy	3	0.005%
Hyperprolactinaemia	3	0.005%
Hyperventilation	3	0.005%
Hypochloraemia	3	0.005%
IIIrd nerve paralysis	3	0.005%
Inflammatory bowel disease	3	0.005%
Injection site pain	3	0.005%
Intraocular pressure increased	3	0.005%
Intussusception	3	0.005%
Ischaemic hepatitis	3	0.005%
Ketosis	3	0.005%
Kidney transplant rejection	3	0.005%
Labyrinthitis	3	0.005%
Laryngeal oedema	3	0.005%
Laryngitis	3	0.005%
Left ventricular failure	3	0.005%
Leukaemoid reaction	3	0.005%
Leukoderma	3	0.005%
Leukoplakia oral	3	0.005%
Lung lobectomy	3	0.005%
Mean cell volume abnormal	3	0.005%
Mediastinum neoplasm	3	0.005%
Meningoencephalitis herpetic	3	0.005%
Migraine	3	0.005%
Mitral valve incompetence	3	0.005%
Mouth haemorrhage	3	0.005%
Mucous stools	3	0.005%
Multiple fractures	3	0.005%
Myoclonus	3	0.005%
Nephritis allergic	3	0.005%
Neutrophilia	3	0.005%
Non-cardiogenic pulmonary oedema	3	0.005%
Nosocomial infection	3	0.005%
Nystagmus	3	0.005%
Obesity	3	0.005%
Oesophageal obstruction	3	0.005%
Onycholysis	3	0.005%
Oral lichen planus	3	0.005%
Otitis externa	3	0.005%
Panic disorder	3	0.005%
Pericardial haemorrhage	3	0.005%
Periorbital oedema	3	0.005%
Personality change	3	0.005%

Pharyngeal ulceration	3	0.005%
Plasma cell myeloma	3	0.005%
Pleural thickening	3	0.005%
Pneumonia pseudomonal	3	0.005%
Pneumothorax traumatic	3	0.005%
Positron emission tomogram abnormal	3	0.005%
Prinzmetal angina	3	0.005%
Prostatic disorder	3	0.005%
Protein total decreased	3	0.005%
Pulmonary sarcoidosis	3	0.005%
Pulmonary venous thrombosis	3	0.005%
Pulse abnormal	3	0.005%
Purpura fulminans	3	0.005%
Purulent discharge	3	0.005%
Pyelonephritis acute	3	0.005%
Renal arteriosclerosis	3	0.005%
Renal colic	3	0.005%
Respiration abnormal	3	0.005%
Respiratory tract haemorrhage	3	0.005%
Retinal oedema	3	0.005%
Retinal vein occlusion	3	0.005%
Retroperitoneal haemorrhage	3	0.005%
Rhinitis allergic	3	0.005%
Screaming	3	0.005%
Shock symptom	3	0.005%
Skin atrophy	3	0.005%
Skin hyperpigmentation	3	0.005%
Skin necrosis	3	0.005%
Splenic rupture	3	0.005%
Sputum increased	3	0.005%
Steatorrhoea	3	0.005%
Sternal fracture	3	0.005%
Subcutaneous abscess	3	0.005%
Suicide attempt	3	0.005%
Superinfection	3	0.005%
Synovial cyst	3	0.005%
Tachycardia paroxysmal	3	0.005%
Tenderness	3	0.005%
Tendon rupture	3	0.005%
Tenosynovitis	3	0.005%
Tongue disorder	3	0.005%
Tracheal obstruction	3	0.005%
Trigeminal neuralgia	3	0.005%
Trismus	3	0.005%
Tuberculous pleurisy	3	0.005%
Urinary bladder haemorrhage	3	0.005%
Urinary tract infection enterococcal	3	0.005%
Vaginal haemorrhage	3	0.005%
Vena cava thrombosis	3	0.005%

Vitamin D deficiency	3	0.005%
Wernicke's encephalopathy	3	0.005%
Wrist fracture	3	0.005%
Kidney enlargement	3	0.005%
Intracardiac thrombus	3	0.005%
Oral infection	3	0.005%
Tooth infection	3	0.005%
Ischaemic cardiomyopathy	3	0.005%
Weight fluctuation	3	0.005%
Cytomegalovirus enteritis	3	0.005%
Pancreatic mass	3	0.005%
Antiacetylcholine receptor antibody positive	3	0.005%
Onychomadesis	3	0.005%
Sudden cardiac death	3	0.005%
Appendiceal abscess	3	0.005%
Blood HIV RNA increased	3	0.005%
Nephrostomy	3	0.005%
Sputum purulent	3	0.005%
Duodenal stenosis	3	0.005%
Tumour ulceration	3	0.005%
Ventricular hypokinesia	3	0.005%
Post procedural haemorrhage	3	0.005%
Chylothorax	3	0.005%
Facial paresis	3	0.005%
Exercise tolerance decreased	3	0.005%
Large intestine polyp	3	0.005%
Metastases to bone marrow	3	0.005%
Metastases to oesophagus	3	0.005%
Metastases to peritoneum	3	0.005%
Adrenomegaly	3	0.005%
Eye pruritus	3	0.005%
VIth nerve disorder	3	0.005%
Vascular occlusion	3	0.005%
Therapeutic procedure	3	0.005%
Bacterial sepsis	3	0.005%
Cardiac ventricular thrombosis	3	0.005%
Laryngeal discomfort	3	0.005%
Escherichia bacteraemia	3	0.005%
Skin burning sensation	3	0.005%
Pancreatic enlargement	3	0.005%
Pericarditis tuberculous	3	0.005%
Congestive cardiomyopathy	3	0.005%
Abdominal infection	3	0.005%
Catheter site infection	3	0.005%
Hepatic infection	3	0.005%
Chronic inflammatory demyelinating polyradiculoneuropathy	3	0.005%
Infected neoplasm	3	0.005%
Thyroid mass	3	0.005%
Ventricular dysfunction	3	0.005%

Palliative care	3	0.005%
Antinuclear antibody positive	3	0.005%
Recall phenomenon	3	0.005%
Abdominal abscess	3	0.005%
Arterial haemorrhage	3	0.005%
Corynebacterium infection	3	0.005%
Intra-abdominal haemorrhage	3	0.005%
Malignant peritoneal neoplasm	3	0.005%
Oesophageal disorder	3	0.005%
Partial seizures	3	0.005%
Pericardial disease	3	0.005%
Pleural infection	3	0.005%
Poisoning	3	0.005%
Streptococcal infection	3	0.005%
Tumour excision	3	0.005%
Intervertebral disc disorder	3	0.005%
Pituitary tumour benign	3	0.005%
Radiotherapy to brain	3	0.005%
Ulcer haemorrhage	3	0.005%
Bone marrow disorder	3	0.005%
Disease recurrence	3	0.005%
Gastrointestinal obstruction	3	0.005%
Herpes ophthalmic	3	0.005%
Hyperlipidaemia	3	0.005%
Perforated ulcer	3	0.005%
Microangiopathy	3	0.005%
Mycobacterial infection	3	0.005%
Soft tissue infection	3	0.005%
Tooth fracture	3	0.005%
Grip strength decreased	3	0.005%
Thoracic haemorrhage	3	0.005%
Carditis	3	0.005%
Diffuse panbronchiolitis	3	0.005%
Infective spondylitis	3	0.005%
Endocrine toxicity	3	0.005%
Cystitis noninfective	3	0.005%
Brachiocephalic vein thrombosis	3	0.005%
Bowel movement irregularity	3	0.005%
Coagulation test abnormal	3	0.005%
Cataract operation	3	0.005%
Gastrointestinal inflammation	3	0.005%
Breakthrough pain	3	0.005%
Antinuclear antibody increased	3	0.005%
Skin abrasion	3	0.005%
Loose tooth	3	0.005%
Pharyngeal inflammation	3	0.005%
Pleural fistula	3	0.005%
Paraneoplastic dermatomyositis	3	0.005%
Post procedural pneumonia	3	0.005%

Acute polyneuropathy	3	0.005%
Acute interstitial pneumonitis	3	0.005%
Adverse reaction	3	0.005%
Disease complication	3	0.005%
Oncologic complication	3	0.005%
Cholestatic liver injury	3	0.005%
Oropharyngeal discomfort	3	0.005%
Langerhans' cell histiocytosis	3	0.005%
H1N1 influenza	3	0.005%
Autoimmune neuropathy	3	0.005%
Aspergillus test positive	3	0.005%
Upper-airway cough syndrome	3	0.005%
Metastases to pelvis	3	0.005%
Seizure like phenomena	3	0.005%
Procedural haemorrhage	3	0.005%
Anaplastic lymphoma kinase gene mutation	3	0.005%
White matter lesion	3	0.005%
Necrotising myositis	3	0.005%
Coating in mouth	3	0.005%
Triple negative breast cancer	3	0.005%
Thyroid hormones decreased	3	0.005%
Tumour obstruction	3	0.005%
End stage renal disease	3	0.005%
Vascular access site infection	3	0.005%
Product availability issue	3	0.005%
Product supply issue	3	0.005%
Neuromyelitis optica spectrum disorder	3	0.005%
Pulmonary artery occlusion	3	0.005%
Drug effective for unapproved indication	3	0.005%
Primary biliary cholangitis	3	0.005%
Gallbladder rupture	3	0.005%
Incorrect route of product administration	3	0.005%
Product administration error	3	0.005%
Product prescribing error	3	0.005%
Therapeutic product effect decreased	3	0.005%
Blood loss anaemia	3	0.005%
Autoimmune cholangitis	3	0.005%
Diverticulitis intestinal perforated	3	0.005%
Immobilisation syndrome	3	0.005%
Coronavirus pneumonia	3	0.005%
Superficial vein thrombosis	3	0.005%
Abdominal adhesions	2	0.003%
Abnormal dreams	2	0.003%
Abscess oral	2	0.003%
Actinic keratosis	2	0.003%
Acute promyelocytic leukaemia	2	0.003%
Adult T-cell lymphoma/leukaemia	2	0.003%
Akathisia	2	0.003%
Alcoholic liver disease	2	0.003%

Alopecia areata	2	0.003%
Amaurosis	2	0.003%
Anal fistula	2	0.003%
Anaphylactoid reaction	2	0.003%
Ankle fracture	2	0.003%
Anoxia	2	0.003%
Aortic aneurysm rupture	2	0.003%
Aortic thrombosis	2	0.003%
Aortitis	2	0.003%
Aphthous ulcer	2	0.003%
Appendicectomy	2	0.003%
Arteriospasm coronary	2	0.003%
Aspartate aminotransferase decreased	2	0.003%
Atonic seizures	2	0.003%
Autonomic nervous system imbalance	2	0.003%
Axonal neuropathy	2	0.003%
B-cell small lymphocytic lymphoma	2	0.003%
Balanoposthitis	2	0.003%
Behaviour disorder	2	0.003%
Behcet's syndrome	2	0.003%
Bell's palsy	2	0.003%
Bile duct cancer	2	0.003%
Bladder catheterisation	2	0.003%
Blepharitis	2	0.003%
Blindness transient	2	0.003%
Blindness unilateral	2	0.003%
Blood calcium abnormal	2	0.003%
Blood creatine abnormal	2	0.003%
Blood creatine phosphokinase abnormal	2	0.003%
Blood fibrinogen increased	2	0.003%
Blood immunoglobulin E increased	2	0.003%
Blood iron decreased	2	0.003%
Blood lactate dehydrogenase abnormal	2	0.003%
Blood prolactin increased	2	0.003%
Blood testosterone decreased	2	0.003%
Body temperature decreased	2	0.003%
Bone cancer	2	0.003%
Brain herniation	2	0.003%
Bronchial carcinoma	2	0.003%
Burkitt's lymphoma	2	0.003%
Calcium deficiency	2	0.003%
Cardiac murmur	2	0.003%
Cardioversion	2	0.003%
CD4 lymphocytes decreased	2	0.003%
Cerebral artery occlusion	2	0.003%
Cerebral artery thrombosis	2	0.003%
Choroidal detachment	2	0.003%
Chronic hepatitis	2	0.003%
Chronic sinusitis	2	0.003%

Coccydynia	2	0.003%
Colostomy	2	0.003%
Corneal opacity	2	0.003%
Corneal perforation	2	0.003%
Cryoglobulinaemia	2	0.003%
CSF lymphocyte count abnormal	2	0.003%
Cutaneous T-cell lymphoma	2	0.003%
Decreased immune responsiveness	2	0.003%
Dental caries	2	0.003%
Dermatitis allergic	2	0.003%
Device breakage	2	0.003%
Diabetic hyperglycaemic coma	2	0.003%
Diplegia	2	0.003%
Disability	2	0.003%
Dressler's syndrome	2	0.003%
Drooling	2	0.003%
Drug dependence	2	0.003%
Ear infection	2	0.003%
Endoscopic retrograde cholangiopancreatography	2	0.003%
Epiglottitis	2	0.003%
Euthanasia	2	0.003%
Exostosis	2	0.003%
Foot fracture	2	0.003%
Gallbladder cancer	2	0.003%
Gingival disorder	2	0.003%
Glioma	2	0.003%
Glomerular filtration rate abnormal	2	0.003%
Graft versus host disease	2	0.003%
Granulocyte count increased	2	0.003%
Granuloma annulare	2	0.003%
Haemangioma of liver	2	0.003%
Haematocrit decreased	2	0.003%
Haemodialysis	2	0.003%
Hallucination, auditory	2	0.003%
Hallucinations, mixed	2	0.003%
Hepatic atrophy	2	0.003%
Hepatic haematoma	2	0.003%
Hepatitis A antibody positive	2	0.003%
Hepatorenal failure	2	0.003%
Hepatosplenomegaly	2	0.003%
Hilar lymphadenopathy	2	0.003%
Hordeolum	2	0.003%
Horner's syndrome	2	0.003%
Hypercoagulation	2	0.003%
Hyperkeratosis	2	0.003%
Hyperphagia	2	0.003%
Hypertensive heart disease	2	0.003%
Hypertrophic osteoarthropathy	2	0.003%
Hypogammaglobulinaemia	2	0.003%

Hypoproteinaemia	2	0.003%
Hypoventilation	2	0.003%
Illusion	2	0.003%
Immobile	2	0.003%
Immunisation reaction	2	0.003%
Impaired gastric emptying	2	0.003%
Injection site extravasation	2	0.003%
Injection site reaction	2	0.003%
Intestinal infarction	2	0.003%
Intracranial tumour haemorrhage	2	0.003%
Iridocyclitis	2	0.003%
Irritable bowel syndrome	2	0.003%
Jugular vein thrombosis	2	0.003%
Laparotomy	2	0.003%
Laryngeal pain	2	0.003%
Libido decreased	2	0.003%
Lichenification	2	0.003%
Listless	2	0.003%
Livedo reticularis	2	0.003%
Loss of control of legs	2	0.003%
Lumbar spinal stenosis	2	0.003%
Lung squamous cell carcinoma recurrent	2	0.003%
Lymph node pain	2	0.003%
Lymph node tuberculosis	2	0.003%
Lymphangioma	2	0.003%
Malabsorption	2	0.003%
Malignant melanoma	2	0.003%
Malignant neoplasm of eye	2	0.003%
Malignant neoplasm of renal pelvis	2	0.003%
Marasmus	2	0.003%
Mediastinitis	2	0.003%
Meningism	2	0.003%
Meningitis tuberculous	2	0.003%
Middle insomnia	2	0.003%
Motion sickness	2	0.003%
Muscle necrosis	2	0.003%
Muscle twitching	2	0.003%
Myocardial necrosis	2	0.003%
Neuromyopathy	2	0.003%
Neutrophil toxic granulation present	2	0.003%
Nipple pain	2	0.003%
Nocardiosis	2	0.003%
Non-small cell lung cancer stage III	2	0.003%
Oesophageal pain	2	0.003%
Oesophageal spasm	2	0.003%
Onychomycosis	2	0.003%
Osteochondrosis	2	0.003%
Ototoxicity	2	0.003%
Ovarian cyst	2	0.003%

Paranoia	2	0.003%
Parathyroid disorder	2	0.003%
Parotid gland enlargement	2	0.003%
Pathogen resistance	2	0.003%
Pelvic venous thrombosis	2	0.003%
Penile ulceration	2	0.003%
Peptic ulcer	2	0.003%
Perioral dermatitis	2	0.003%
Peripheral T-cell lymphoma unspecified	2	0.003%
Peroneal nerve palsy	2	0.003%
Pharyngeal cancer	2	0.003%
Pharyngeal oedema	2	0.003%
Platelet count abnormal	2	0.003%
Pleuritic pain	2	0.003%
Pneumoconiosis	2	0.003%
Pneumonia influenzal	2	0.003%
Pneumonia legionella	2	0.003%
Polyarteritis nodosa	2	0.003%
Polycythaemia	2	0.003%
Polycythaemia vera	2	0.003%
Polyneuropathy in malignant disease	2	0.003%
Polyuria	2	0.003%
Poor peripheral circulation	2	0.003%
Post herpetic neuralgia	2	0.003%
Proctitis ulcerative	2	0.003%
Prostatic specific antigen increased	2	0.003%
Protein urine	2	0.003%
Psychomotor hyperactivity	2	0.003%
Pulmonary granuloma	2	0.003%
Pulmonary infarction	2	0.003%
Quadriplegia	2	0.003%
Radiation myelopathy	2	0.003%
Radius fracture	2	0.003%
Rales	2	0.003%
Rash morbilliform	2	0.003%
Rectal cancer	2	0.003%
Renal cancer	2	0.003%
Renal tubular acidosis	2	0.003%
Renal vasculitis	2	0.003%
Retinitis	2	0.003%
Ruptured cerebral aneurysm	2	0.003%
Salivary hypersecretion	2	0.003%
Scapula fracture	2	0.003%
Seborrhoeic dermatitis	2	0.003%
Secondary amyloidosis	2	0.003%
Serotonin syndrome	2	0.003%
Skin depigmentation	2	0.003%
Skin fissures	2	0.003%
Skin hypopigmentation	2	0.003%

Skin papilloma	2	0.003%
Sluggishness	2	0.003%
Small cell lung cancer recurrent	2	0.003%
Small intestinal resection	2	0.003%
Snoring	2	0.003%
Spinal cord injury	2	0.003%
Splenic haemorrhage	2	0.003%
Splenomegaly	2	0.003%
Spondylitic myelopathy	2	0.003%
Starvation	2	0.003%
Strabismus	2	0.003%
Subdural haemorrhage	2	0.003%
Suffocation feeling	2	0.003%
Suicidal ideation	2	0.003%
Supraventricular extrasystoles	2	0.003%
Swelling of eyelid	2	0.003%
Tendon injury	2	0.003%
Teratoma	2	0.003%
Thrombolysis	2	0.003%
Thrombophlebitis septic	2	0.003%
Tonic convulsion	2	0.003%
Toxic optic neuropathy	2	0.003%
Tracheitis	2	0.003%
Tracheostomy	2	0.003%
Tricuspid valve incompetence	2	0.003%
Ureteric obstruction	2	0.003%
Ureteric stenosis	2	0.003%
Urinary tract disorder	2	0.003%
Urine flow decreased	2	0.003%
Uterine cancer	2	0.003%
Uterine leiomyoma	2	0.003%
Varicella	2	0.003%
Vein disorder	2	0.003%
Ventricular arrhythmia	2	0.003%
Vesical fistula	2	0.003%
Vestibular neuronitis	2	0.003%
Visual field defect	2	0.003%
Vitamin B12 deficiency	2	0.003%
Vitreous opacities	2	0.003%
Vitritis	2	0.003%
Vocal cord dysfunction	2	0.003%
Volvulus	2	0.003%
Wound dehiscence	2	0.003%
Pneumoperitoneum	2	0.003%
Cardiomyopathy acute	2	0.003%
Multiple sclerosis relapse	2	0.003%
Endothelial dysfunction	2	0.003%
Subdural hygroma	2	0.003%
Hypereosinophilic syndrome	2	0.003%

Restrictive pulmonary disease	2	0.003%
Terminal state	2	0.003%
Pituitary enlargement	2	0.003%
Joint range of motion decreased	2	0.003%
Onychoclasis	2	0.003%
Seasonal allergy	2	0.003%
Rectal abscess	2	0.003%
Lymphadenectomy	2	0.003%
Gastrostomy	2	0.003%
Encephalitis brain stem	2	0.003%
Cytomegalovirus gastritis	2	0.003%
Chapped lips	2	0.003%
Emotional distress	2	0.003%
Neck mass	2	0.003%
Vocal cord paresis	2	0.003%
Food aversion	2	0.003%
Muscle fatigue	2	0.003%
Fungal oesophagitis	2	0.003%
Metastases to heart	2	0.003%
Pituitary haemorrhage	2	0.003%
Bradyarrhythmia	2	0.003%
Blood glucose fluctuation	2	0.003%
Medical device implantation	2	0.003%
Blood bilirubin decreased	2	0.003%
Palmoplantar pustulosis	2	0.003%
Cervical radiculopathy	2	0.003%
Ureteral stent insertion	2	0.003%
Face injury	2	0.003%
Cold agglutinins	2	0.003%
Hip surgery	2	0.003%
Gastrointestinal stromal tumour	2	0.003%
Rhinalgia	2	0.003%
Conjunctival hyperaemia	2	0.003%
Metastases to spleen	2	0.003%
Bile duct stent insertion	2	0.003%
Haemodynamic instability	2	0.003%
Gastrointestinal hypomotility	2	0.003%
Ear discomfort	2	0.003%
Impaired insulin secretion	2	0.003%
Neuroendocrine tumour	2	0.003%
Ocular vascular disorder	2	0.003%
Brain natriuretic peptide abnormal	2	0.003%
Bronchopulmonary disease	2	0.003%
Tracheal disorder	2	0.003%
Urine protein/creatinine ratio increased	2	0.003%
Thermal burn	2	0.003%
Vascular rupture	2	0.003%
Wound complication	2	0.003%
Acquired haemophilia	2	0.003%

Brain tumour operation	2	0.003%
Eosinophilic cellulitis	2	0.003%
Tri-iodothyronine free increased	2	0.003%
Tri-iodothyronine free abnormal	2	0.003%
Evans syndrome	2	0.003%
Pneumococcal sepsis	2	0.003%
Depressive symptom	2	0.003%
Klebsiella sepsis	2	0.003%
Affect lability	2	0.003%
Helicobacter infection	2	0.003%
Arterial fibrosis	2	0.003%
Peripheral paralysis	2	0.003%
Vertebral column mass	2	0.003%
Lipase abnormal	2	0.003%
Splenic lesion	2	0.003%
Mean platelet volume decreased	2	0.003%
Haemorrhage urinary tract	2	0.003%
Varices oesophageal	2	0.003%
Food craving	2	0.003%
Cutaneous lupus erythematosus	2	0.003%
Psoas abscess	2	0.003%
Periorbital swelling	2	0.003%
Gastric infection	2	0.003%
Peripheral sensorimotor neuropathy	2	0.003%
Bronchial oedema	2	0.003%
Neurological examination abnormal	2	0.003%
Near drowning	2	0.003%
Gastrointestinal mucosal disorder	2	0.003%
Pharyngeal erythema	2	0.003%
Hepatic mass	2	0.003%
Urine odour abnormal	2	0.003%
Biliary tract dilation procedure	2	0.003%
Cell death	2	0.003%
Lower respiratory tract inflammation	2	0.003%
Therapeutic embolisation	2	0.003%
Peripheral artery aneurysm	2	0.003%
Peripheral artery occlusion	2	0.003%
Urine ketone body present	2	0.003%
Anxiety disorder	2	0.003%
Biliary sepsis	2	0.003%
Upper respiratory tract infection bacterial	2	0.003%
Rheumatoid factor increased	2	0.003%
Abdominal sepsis	2	0.003%
Administration site pain	2	0.003%
Multi-organ disorder	2	0.003%
Dyslipidaemia	2	0.003%
Ocular icterus	2	0.003%
Bronchioloalveolar carcinoma	2	0.003%
Cerebrovascular insufficiency	2	0.003%

Cytomegalovirus viraemia	2	0.003%
Superior vena cava occlusion	2	0.003%
Acute hepatitis B	2	0.003%
Delirium febrile	2	0.003%
Epidermal necrosis	2	0.003%
Bacterial test positive	2	0.003%
Fungal test positive	2	0.003%
Blood alkaline phosphatase decreased	2	0.003%
Adhesion	2	0.003%
Anti-thyroid antibody positive	2	0.003%
Gastrointestinal erosion	2	0.003%
Intervertebral discitis	2	0.003%
Ischaemic cerebral infarction	2	0.003%
Aortic rupture	2	0.003%
Sputum abnormal	2	0.003%
Blood electrolytes abnormal	2	0.003%
Connective tissue disorder	2	0.003%
Cranial nerve disorder	2	0.003%
Hormone level abnormal	2	0.003%
Infarction	2	0.003%
Large intestinal ulcer haemorrhage	2	0.003%
Monocyte count abnormal	2	0.003%
Mucosal erosion	2	0.003%
Nail infection	2	0.003%
Optic nerve disorder	2	0.003%
Pelvic neoplasm	2	0.003%
Skeletal injury	2	0.003%
Spondylitis	2	0.003%
Traumatic intracranial haemorrhage	2	0.003%
Cardiac valve disease	2	0.003%
Corneal disorder	2	0.003%
Feeling of body temperature change	2	0.003%
Scleral disorder	2	0.003%
Polyp	2	0.003%
Red blood cell abnormality	2	0.003%
Adrenal neoplasm	2	0.003%
Bronchitis bacterial	2	0.003%
Occult blood positive	2	0.003%
Salivary gland disorder	2	0.003%
Immunosuppression	2	0.003%
Lymphocyte morphology abnormal	2	0.003%
Peritonitis bacterial	2	0.003%
Respiratory gas exchange disorder	2	0.003%
Metabolic encephalopathy	2	0.003%
Metastases to soft tissue	2	0.003%
Nasal disorder	2	0.003%
Small intestinal stenosis	2	0.003%
Paranasal cyst	2	0.003%
Unevaluable event	2	0.003%

Enterovesical fistula	2	0.003%
Corneal endotheliitis	2	0.003%
Foaming at mouth	2	0.003%
Bladder tamponade	2	0.003%
Neutropenic colitis	2	0.003%
Mesenteric panniculitis	2	0.003%
Brain stem syndrome	2	0.003%
Acarodermatitis	2	0.003%
Herpes zoster oticus	2	0.003%
Radiation skin injury	2	0.003%
Vertebroplasty	2	0.003%
Vital functions abnormal	2	0.003%
Tumour invasion	2	0.003%
KL-6 increased	2	0.003%
Device occlusion	2	0.003%
Pancreatic duct dilatation	2	0.003%
Joint instability	2	0.003%
Ulcerative keratitis	2	0.003%
Herpes zoster disseminated	2	0.003%
Polychondritis	2	0.003%
Wound infection bacterial	2	0.003%
Laryngeal haemorrhage	2	0.003%
Central nervous system necrosis	2	0.003%
Pulmonary fistula	2	0.003%
Continuous haemodiafiltration	2	0.003%
Latent autoimmune diabetes in adults	2	0.003%
Corneal leukoma	2	0.003%
Carotid arteriosclerosis	2	0.003%
Mechanical ventilation	2	0.003%
Haemostasis	2	0.003%
Renal cell carcinoma	2	0.003%
Radiation fibrosis	2	0.003%
Butterfly rash	2	0.003%
Tumour thrombosis	2	0.003%
Near death experience	2	0.003%
Anti-cyclic citrullinated peptide antibody positive	2	0.003%
Antisynthetase syndrome	2	0.003%
Histiocytic necrotising lymphadenitis	2	0.003%
Henoch-Schonlein purpura nephritis	2	0.003%
Stent malfunction	2	0.003%
Clostridium test positive	2	0.003%
Escherichia test positive	2	0.003%
Candida test positive	2	0.003%
Product distribution issue	2	0.003%
Functional gastrointestinal disorder	2	0.003%
Dropped head syndrome	2	0.003%
Granulomatosis with polyangiitis	2	0.003%
Abdominal lymphadenopathy	2	0.003%
Ischaemic enteritis	2	0.003%

Herpes zoster meningitis	2	0.003%
Therapy change	2	0.003%
Ophthalmic vein thrombosis	2	0.003%
Cancer surgery	2	0.003%
Vein collapse	2	0.003%
Granulomatous dermatitis	2	0.003%
Peripheral venous disease	2	0.003%
Bone marrow infiltration	2	0.003%
Internal haemorrhage	2	0.003%
Mouth swelling	2	0.003%
Lower respiratory tract congestion	2	0.003%
Varicella zoster virus infection	2	0.003%
Cytomegalovirus gastrointestinal ulcer	2	0.003%
Autoimmune encephalopathy	2	0.003%
Tongue fungal infection	2	0.003%
Administration site extravasation	2	0.003%
Product leakage	2	0.003%
Nasal pruritus	2	0.003%
Inability to afford medication	2	0.003%
Perforation	2	0.003%
Acquired amegakaryocytic thrombocytopenia	2	0.003%
Jugular vein occlusion	2	0.003%
Product preparation error	2	0.003%
Kidney congestion	2	0.003%
Transitional cell carcinoma recurrent	2	0.003%
Immunoglobulin G4 related disease	2	0.003%
Device related thrombosis	2	0.003%
Intracranial mass	2	0.003%
Frustration tolerance decreased	2	0.003%
Procalcitonin abnormal	2	0.003%
Tongue discomfort	2	0.003%
Somatic symptom disorder	2	0.003%
Drug-device incompatibility	2	0.003%
Metastases to spinal cord	2	0.003%
Idiopathic interstitial pneumonia	2	0.003%
Intra-abdominal fluid collection	2	0.003%
Oesophageal prosthesis insertion	2	0.003%
Cystic lung disease	2	0.003%
Renal tubular injury	2	0.003%
Defaecation disorder	2	0.003%
Lip erythema	2	0.003%
Eosinophilic pleural effusion	2	0.003%
Autoimmune anaemia	2	0.003%
Trichoglossia	2	0.003%
Product preparation issue	2	0.003%
Autoimmune enteropathy	2	0.003%
Suspected product quality issue	2	0.003%
Therapeutic product effect delayed	2	0.003%
Autoimmune myositis	2	0.003%

SARS-CoV-2 test negative	2	0.003%
Disseminated varicella zoster virus infection	2	0.003%
Immune-mediated oesophagitis	2	0.003%
Positive airway pressure therapy	2	0.003%
Superficial inflammatory dermatosis	2	0.003%
Colorectal adenoma	2	0.003%
Abdominal mass	1	0.002%
Abdominal wall abscess	1	0.002%
Abnormal loss of weight	1	0.002%
Abscess intestinal	1	0.002%
Acetabulum fracture	1	0.002%
Acidosis hyperchloraemic	1	0.002%
Acrochordon	1	0.002%
Actinomycosis	1	0.002%
Actinomycotic pulmonary infection	1	0.002%
Acupuncture	1	0.002%
Acute febrile neutrophilic dermatosis	1	0.002%
Acute leukaemia	1	0.002%
Acute lymphocytic leukaemia	1	0.002%
Acute psychosis	1	0.002%
Adenocarcinoma of colon	1	0.002%
Adenosquamous cell lung cancer	1	0.002%
Adrenal atrophy	1	0.002%
Adrenal suppression	1	0.002%
Agraphia	1	0.002%
Alanine aminotransferase	1	0.002%
Alanine aminotransferase decreased	1	0.002%
Alkalosis	1	0.002%
Alpha 1 foetoprotein increased	1	0.002%
Amaurosis fugax	1	0.002%
Ammonia abnormal	1	0.002%
Ammonia increased	1	0.002%
Amoebic colitis	1	0.002%
Amylase	1	0.002%
Amylase decreased	1	0.002%
Amyloidosis	1	0.002%
Anaemia macrocytic	1	0.002%
Anaemia of chronic disease	1	0.002%
Anaemia vitamin B12 deficiency	1	0.002%
Anaplastic thyroid cancer	1	0.002%
Aneurysm	1	0.002%
Angiocentric lymphoma	1	0.002%
Angioplasty	1	0.002%
Angle closure glaucoma	1	0.002%
Animal scratch	1	0.002%
Anisocoria	1	0.002%
Ankylosing spondylitis	1	0.002%
Antinuclear antibody	1	0.002%
Aortic valve stenosis	1	0.002%

Aplasia	1	0.002%
Apnoea	1	0.002%
Areflexia	1	0.002%
Arterial injury	1	0.002%
Arteritis coronary	1	0.002%
Arthropod bite	1	0.002%
Aspergilloma	1	0.002%
Asterixis	1	0.002%
Asthenopia	1	0.002%
Atonic urinary bladder	1	0.002%
Atrioventricular block first degree	1	0.002%
Auditory disorder	1	0.002%
Axillary vein thrombosis	1	0.002%
B-cell lymphoma	1	0.002%
Back injury	1	0.002%
Benign mediastinal neoplasm	1	0.002%
Benign neoplasm of adrenal gland	1	0.002%
Bilirubin conjugated increased	1	0.002%
Biopsy kidney	1	0.002%
Biopsy liver	1	0.002%
Bladder cancer recurrent	1	0.002%
Bladder diverticulum	1	0.002%
Bladder neoplasm	1	0.002%
Bladder pain	1	0.002%
Bladder papilloma	1	0.002%
Bladder transitional cell carcinoma	1	0.002%
Blood aldosterone increased	1	0.002%
Blood antidiuretic hormone abnormal	1	0.002%
Blood antidiuretic hormone increased	1	0.002%
Blood bicarbonate increased	1	0.002%
Blood calcitonin	1	0.002%
Blood chloride abnormal	1	0.002%
Blood chloride increased	1	0.002%
Blood corticotrophin increased	1	0.002%
Blood creatine decreased	1	0.002%
Blood culture positive	1	0.002%
Blood follicle stimulating hormone increased	1	0.002%
Blood immunoglobulin G decreased	1	0.002%
Blood iron abnormal	1	0.002%
Blood lactic acid decreased	1	0.002%
Blood luteinising hormone increased	1	0.002%
Blood osmolarity decreased	1	0.002%
Blood parathyroid hormone abnormal	1	0.002%
Blood pressure fluctuation	1	0.002%
Blood pressure systolic abnormal	1	0.002%
Blood pressure systolic decreased	1	0.002%
Blood pressure systolic increased	1	0.002%
Blood prolactin abnormal	1	0.002%
Blood urea abnormal	1	0.002%

Blood urine	1	0.002%
Blood zinc decreased	1	0.002%
Body mass index decreased	1	0.002%
Body temperature	1	0.002%
Bone neoplasm	1	0.002%
Brain hypoxia	1	0.002%
Brain neoplasm malignant	1	0.002%
Brain stem infarction	1	0.002%
Breast haemorrhage	1	0.002%
Breast tenderness	1	0.002%
Breath odour	1	0.002%
Bronchopulmonary aspergillosis allergic	1	0.002%
Bronchopulmonary dysplasia	1	0.002%
Bronchoscopy	1	0.002%
Bundle branch block	1	0.002%
Burning sensation mucosal	1	0.002%
C-reactive protein	1	0.002%
Calculus prostatic	1	0.002%
Calculus urinary	1	0.002%
Campylobacter gastroenteritis	1	0.002%
Carbon dioxide decreased	1	0.002%
Carbon dioxide increased	1	0.002%
Cardiac failure high output	1	0.002%
Carotid artery thrombosis	1	0.002%
Cataract cortical	1	0.002%
Cataract nuclear	1	0.002%
Catatonia	1	0.002%
Central nervous system neoplasm	1	0.002%
Cerebellar atrophy	1	0.002%
Cerebral venous thrombosis	1	0.002%
Cerebrospinal fluid leakage	1	0.002%
Cervical spinal stenosis	1	0.002%
Cervicobrachial syndrome	1	0.002%
Choking sensation	1	0.002%
Cholangiocarcinoma	1	0.002%
Cholinergic syndrome	1	0.002%
Chorea	1	0.002%
Chorioretinitis	1	0.002%
Choroid plexus papilloma	1	0.002%
Choroiditis	1	0.002%
Chronic hepatitis C	1	0.002%
Chronic lymphocytic leukaemia	1	0.002%
Chronic lymphocytic leukaemia recurrent	1	0.002%
Circadian rhythm sleep disorder	1	0.002%
Claustrophobia	1	0.002%
CNS ventriculitis	1	0.002%
Coagulation time prolonged	1	0.002%
Cold agglutinins positive	1	0.002%
Collagen disorder	1	0.002%

Colonoscopy abnormal	1	0.002%
Colony stimulating factor therapy	1	0.002%
Compartment syndrome	1	0.002%
Compulsions	1	0.002%
Concussion	1	0.002%
Confabulation	1	0.002%
Congenital anomaly	1	0.002%
Congenital diaphragmatic hernia	1	0.002%
Conjunctivitis allergic	1	0.002%
Contrast media reaction	1	0.002%
Coombs positive haemolytic anaemia	1	0.002%
Corneal degeneration	1	0.002%
Corneal epithelium defect	1	0.002%
Corneal erosion	1	0.002%
Cortisol increased	1	0.002%
Cranioplasty	1	0.002%
Creatinine renal clearance increased	1	0.002%
Creutzfeldt-Jakob disease	1	0.002%
CSF cell count increased	1	0.002%
CSF protein increased	1	0.002%
Cyst	1	0.002%
Dacryoadenitis acquired	1	0.002%
Defaecation urgency	1	0.002%
Delirium tremens	1	0.002%
Delusion	1	0.002%
Delusional disorder, unspecified type	1	0.002%
Dementia Alzheimer's type	1	0.002%
Dental necrosis	1	0.002%
Dermabrasion	1	0.002%
Dermatitis atopic	1	0.002%
Diabetic retinopathy	1	0.002%
Diaphragm muscle weakness	1	0.002%
Diaphragmatic paralysis	1	0.002%
Diet refusal	1	0.002%
Differential white blood cell count abnormal	1	0.002%
Diverticular fistula	1	0.002%
Drowning	1	0.002%
Drug level increased	1	0.002%
Dumping syndrome	1	0.002%
Duodenal ulcer perforation	1	0.002%
Dupuytren's contracture	1	0.002%
Dupuytren's contracture operation	1	0.002%
Dysmetria	1	0.002%
Dysphoria	1	0.002%
Ear haemorrhage	1	0.002%
Ear swelling	1	0.002%
Ecchymosis	1	0.002%
Ectopic antidiuretic hormone secretion	1	0.002%
Eczema nummular	1	0.002%

Ejection fraction abnormal	1	0.002%
Electrocardiogram abnormal	1	0.002%
Electrocardiogram QRS complex prolonged	1	0.002%
Electrocochleogram abnormal	1	0.002%
Electroencephalogram abnormal	1	0.002%
Empty sella syndrome	1	0.002%
Encephalitis viral	1	0.002%
Endometrial adenocarcinoma	1	0.002%
Endophthalmitis	1	0.002%
Enterococcal bacteraemia	1	0.002%
Enuresis	1	0.002%
Eosinophil count normal	1	0.002%
Eosinophilic myocarditis	1	0.002%
Epidermolysis bullosa	1	0.002%
Epiglottic oedema	1	0.002%
Episcleritis	1	0.002%
Erythropenia	1	0.002%
Essential thrombocythaemia	1	0.002%
Exsanguination	1	0.002%
Extensor plantar response	1	0.002%
Extrapyramidal disorder	1	0.002%
Extravasation blood	1	0.002%
Eyelid sensory disorder	1	0.002%
Facial bones fracture	1	0.002%
Factor VIII deficiency	1	0.002%
Faeces hard	1	0.002%
Faeces pale	1	0.002%
Feeling drunk	1	0.002%
Fibrin degradation products increased	1	0.002%
Fibrosis	1	0.002%
Finger amputation	1	0.002%
Folate deficiency	1	0.002%
Food allergy	1	0.002%
Food poisoning	1	0.002%
Forced expiratory volume decreased	1	0.002%
Foreign body aspiration	1	0.002%
Foster-Kennedy Syndrome	1	0.002%
Fractured sacrum	1	0.002%
Gallbladder oedema	1	0.002%
Gamma-glutamyltransferase decreased	1	0.002%
Gastric mucosal hypertrophy	1	0.002%
Gastroduodenal ulcer	1	0.002%
Gastroenteritis radiation	1	0.002%
Gastroenteritis rotavirus	1	0.002%
Gastrointestinal arteriovenous malformation	1	0.002%
Genital herpes	1	0.002%
Genital ulceration	1	0.002%
Gingival bleeding	1	0.002%
Gingival hypertrophy	1	0.002%

Glomerular filtration rate increased	1	0.002%
Glomerular vascular disorder	1	0.002%
Glomerulonephritis acute	1	0.002%
Glomerulonephritis minimal lesion	1	0.002%
Glomerulonephritis proliferative	1	0.002%
Glycosuria	1	0.002%
Glucose urine present	1	0.002%
Guttate psoriasis	1	0.002%
Haemangioma	1	0.002%
Haemochromatosis	1	0.002%
Haemolytic uraemic syndrome	1	0.002%
Haemoperitoneum	1	0.002%
Haemosiderosis	1	0.002%
Hand-foot-and-mouth disease	1	0.002%
Hand fracture	1	0.002%
Head discomfort	1	0.002%
Heat exhaustion	1	0.002%
Heat stroke	1	0.002%
Hepatic fibrosis	1	0.002%
Hepatic rupture	1	0.002%
Hepatitis alcoholic	1	0.002%
Hepatitis D	1	0.002%
Hepatorenal syndrome	1	0.002%
Herpes virus infection	1	0.002%
Hidradenitis	1	0.002%
High density lipoprotein decreased	1	0.002%
Hip arthroplasty	1	0.002%
Histiocytosis	1	0.002%
HIV infection	1	0.002%
Human T-cell lymphotropic virus type I infection	1	0.002%
Hyperacusis	1	0.002%
Hyperadrenocorticism	1	0.002%
Hyperaemia	1	0.002%
Hyperaldosteronism	1	0.002%
Hyperammonaemia	1	0.002%
Hypercalcaemia of malignancy	1	0.002%
Hyperchloraemia	1	0.002%
Hyperchlorhydria	1	0.002%
Hypercholesterolaemia	1	0.002%
Hypermagnesaemia	1	0.002%
Hypermetabolism	1	0.002%
Hyperosmolar state	1	0.002%
Hyperparathyroidism	1	0.002%
Hyperparathyroidism secondary	1	0.002%
Hyperplasia	1	0.002%
Hyperplasia adrenal	1	0.002%
Hyperreflexia	1	0.002%
Hypertensive encephalopathy	1	0.002%
Hypertrophic cardiomyopathy	1	0.002%

Hypervolaemia	1	0.002%
Hypoglycaemic coma	1	0.002%
Hyponatraemic syndrome	1	0.002%
Hyporeflexia	1	0.002%
Hysterectomy	1	0.002%
Ichthyosis	1	0.002%
Ileal gangrene	1	0.002%
Ileal perforation	1	0.002%
Ileostomy	1	0.002%
Ilium fracture	1	0.002%
Impetigo	1	0.002%
Inappropriate affect	1	0.002%
Incarcerated inguinal hernia	1	0.002%
Incisional hernia	1	0.002%
Incoherent	1	0.002%
Infection parasitic	1	0.002%
Infectious mononucleosis	1	0.002%
Infective myositis	1	0.002%
Inguinal hernia, obstructive	1	0.002%
Initial insomnia	1	0.002%
Injection site haemorrhage	1	0.002%
Injection site rash	1	0.002%
Intensive care	1	0.002%
Intentional self-injury	1	0.002%
International normalised ratio decreased	1	0.002%
Intervertebral disc displacement	1	0.002%
Intestinal dilatation	1	0.002%
Intestinal fistula	1	0.002%
Intestinal pseudo-obstruction	1	0.002%
Intraventricular haemorrhage	1	0.002%
Iritis	1	0.002%
Jaw fracture	1	0.002%
Jejunal ulcer	1	0.002%
Jejunostomy	1	0.002%
Judgement impaired	1	0.002%
Kaposi's sarcoma	1	0.002%
Keratoacanthoma	1	0.002%
Kidney fibrosis	1	0.002%
Lacrimation disorder	1	0.002%
Leg amputation	1	0.002%
Leriche syndrome	1	0.002%
Lichen nitidus	1	0.002%
Life support	1	0.002%
Linear IgA disease	1	0.002%
Lip discolouration	1	0.002%
Lip dry	1	0.002%
Lip oedema	1	0.002%
Lip pain	1	0.002%
Lip ulceration	1	0.002%

Lipids increased	1	0.002%
Lipoma	1	0.002%
Listeriosis	1	0.002%
Lividity	1	0.002%
Local reaction	1	0.002%
Lumbar puncture	1	0.002%
Lumbar puncture abnormal	1	0.002%
Lupus nephritis	1	0.002%
Lymph node fibrosis	1	0.002%
Lymphocytosis	1	0.002%
Macular oedema	1	0.002%
Malignant hypertension	1	0.002%
Mallet finger	1	0.002%
Mania	1	0.002%
Marrow hyperplasia	1	0.002%
Mastication disorder	1	0.002%
Mastoiditis	1	0.002%
Mean cell haemoglobin increased	1	0.002%
Mediastinal abscess	1	0.002%
Mediastinal fibrosis	1	0.002%
Mediastinal mass	1	0.002%
Medication error	1	0.002%
Melanocytic naevus	1	0.002%
Meningioma	1	0.002%
Meningitis listeria	1	0.002%
Metabolic alkalosis	1	0.002%
Metastases to abdominal cavity	1	0.002%
Metastases to neck	1	0.002%
Metastases to the mediastinum	1	0.002%
Microcytic anaemia	1	0.002%
Micturition disorder	1	0.002%
Mitral valve prolapse	1	0.002%
Mitral valve stenosis	1	0.002%
Mixed connective tissue disease	1	0.002%
Monocyte count increased	1	0.002%
Mononeuritis	1	0.002%
Mononeuropathy multiplex	1	0.002%
Motor neurone disease	1	0.002%
Mucocutaneous ulceration	1	0.002%
Mucosal ulceration	1	0.002%
Muscle haemorrhage	1	0.002%
Muscle rigidity	1	0.002%
Myelofibrosis	1	0.002%
Myelopathy	1	0.002%
Myopathy endocrine	1	0.002%
Myopathy toxic	1	0.002%
Myopia	1	0.002%
Nail dystrophy	1	0.002%
Nail psoriasis	1	0.002%

Nasal inflammation	1	0.002%
Nasal obstruction	1	0.002%
Nasal polyps	1	0.002%
Nasal septum deviation	1	0.002%
Nephrectomy	1	0.002%
Nephrogenic diabetes insipidus	1	0.002%
Nephrosclerosis	1	0.002%
Nerve root injury cervical	1	0.002%
Neuralgic amyotrophy	1	0.002%
Neuritis cranial	1	0.002%
Neuro-ophthalmological test abnormal	1	0.002%
Neuroendocrine carcinoma of the skin	1	0.002%
Neuroleptic malignant syndrome	1	0.002%
Neurosis	1	0.002%
Neurosyphilis	1	0.002%
Nodal arrhythmia	1	0.002%
Nodal rhythm	1	0.002%
Non-Hodgkin's lymphoma	1	0.002%
Normochromic normocytic anaemia	1	0.002%
Obliterative bronchiolitis	1	0.002%
Ocular hypertension	1	0.002%
Oesophageal haemorrhage	1	0.002%
Oesophageal ulcer haemorrhage	1	0.002%
Oesophageal varices haemorrhage	1	0.002%
Ophthalmic herpes zoster	1	0.002%
Optic atrophy	1	0.002%
Orbital oedema	1	0.002%
Organic brain syndrome	1	0.002%
Oropharyngeal spasm	1	0.002%
Orthopnoea	1	0.002%
Osteitis	1	0.002%
Osteomyelitis acute	1	0.002%
Osteomyelitis chronic	1	0.002%
Osteoporotic fracture	1	0.002%
Ovarian haematoma	1	0.002%
Overweight	1	0.002%
Oxygen saturation increased	1	0.002%
Painful respiration	1	0.002%
Palmar erythema	1	0.002%
Pancreatic carcinoma recurrent	1	0.002%
Pancreatic cyst	1	0.002%
Pancreatitis necrotising	1	0.002%
Panic reaction	1	0.002%
Papillary thyroid cancer	1	0.002%
Papillitis	1	0.002%
Paraparesis	1	0.002%
Parathyroid tumour benign	1	0.002%
Parotid abscess	1	0.002%
PCO2 increased	1	0.002%

Penile swelling	1	0.002%
Periarthritis	1	0.002%
Pericarditis adhesive	1	0.002%
Periodontitis	1	0.002%
Peripheral nerve injury	1	0.002%
Peripheral vascular disorder	1	0.002%
Peritonsillar abscess	1	0.002%
Personality change due to a general medical condition	1	0.002%
Personality disorder	1	0.002%
Petit mal epilepsy	1	0.002%
Pharyngitis streptococcal	1	0.002%
Phimosis	1	0.002%
Pituitary tumour	1	0.002%
Pityriasis rubra pilaris	1	0.002%
Pleural calcification	1	0.002%
Pleural mesothelioma	1	0.002%
Pneumaturia	1	0.002%
Pneumonectomy	1	0.002%
Pneumonia escherichia	1	0.002%
Pneumonia haemophilus	1	0.002%
Pneumonia streptococcal	1	0.002%
Polydipsia	1	0.002%
Polyserositis	1	0.002%
Posture abnormal	1	0.002%
Poverty of speech	1	0.002%
Proctitis haemorrhagic	1	0.002%
Prostate cancer metastatic	1	0.002%
Prostatic specific antigen decreased	1	0.002%
Protein total abnormal	1	0.002%
Protein total normal	1	0.002%
Prurigo	1	0.002%
Psychomotor retardation	1	0.002%
Pulmonary artery aneurysm	1	0.002%
Pulmonary valve incompetence	1	0.002%
Pulpitis dental	1	0.002%
Pustule	1	0.002%
Pyelitis	1	0.002%
Pyoderma gangrenosum	1	0.002%
Pyuria	1	0.002%
Radial nerve palsy	1	0.002%
Radiation associated pain	1	0.002%
Reading disorder	1	0.002%
Rectal ulcer haemorrhage	1	0.002%
Red blood cell count abnormal	1	0.002%
Relapsing fever	1	0.002%
Renal aneurysm	1	0.002%
Renal artery thrombosis	1	0.002%
Renal tubular atrophy	1	0.002%
Respiratory alkalosis	1	0.002%

Respiratory rate increased	1	0.002%
Retinal pigment epitheliopathy	1	0.002%
Retinal tear	1	0.002%
Retinal vascular disorder	1	0.002%
Retinopathy hypertensive	1	0.002%
Retrograde amnesia	1	0.002%
Rheumatic fever	1	0.002%
Rocky mountain spotted fever	1	0.002%
Rosacea	1	0.002%
Rubber sensitivity	1	0.002%
Salivary gland calculus	1	0.002%
Salmonellosis	1	0.002%
Salpingo-oophorectomy bilateral	1	0.002%
Sarcoma	1	0.002%
Scintillating scotoma	1	0.002%
Scleritis	1	0.002%
Scleroderma-like reaction	1	0.002%
Sclerotherapy	1	0.002%
Scoliosis	1	0.002%
Scrotal irritation	1	0.002%
Scrotal oedema	1	0.002%
Scrotal swelling	1	0.002%
Seborrhoeic keratosis	1	0.002%
Sedation	1	0.002%
Sensation of blood flow	1	0.002%
Sense of oppression	1	0.002%
Septic embolus	1	0.002%
Shunt occlusion	1	0.002%
Silicosis	1	0.002%
Sinus headache	1	0.002%
Sinus pain	1	0.002%
Skin cancer	1	0.002%
Skin warm	1	0.002%
Sleep disorder due to general medical condition, insomnia type	1	0.002%
Small cell carcinoma	1	0.002%
Social avoidant behaviour	1	0.002%
Solar dermatitis	1	0.002%
Spinal cord injury cervical	1	0.002%
Spinal laminectomy	1	0.002%
Splenectomy	1	0.002%
Squamous cell carcinoma of lung	1	0.002%
Staring	1	0.002%
Stasis dermatitis	1	0.002%
Still's disease	1	0.002%
Stupor	1	0.002%
Subcutaneous haematoma	1	0.002%
T-cell lymphoma	1	0.002%
Teeth brittle	1	0.002%
Tendon disorder	1	0.002%

Tension	1	0.002%
Tension headache	1	0.002%
Testicular pain	1	0.002%
Testicular swelling	1	0.002%
Therapeutic response increased	1	0.002%
Thrombectomy	1	0.002%
Thrombocytopenic purpura	1	0.002%
Thrombosis mesenteric vessel	1	0.002%
Thymoma	1	0.002%
Thyroid function test	1	0.002%
Thyroiditis acute	1	0.002%
Thyroiditis chronic	1	0.002%
Thyroxine decreased	1	0.002%
Tobacco abuse	1	0.002%
Toe operation	1	0.002%
Tongue coated	1	0.002%
Tongue discolouration	1	0.002%
Tongue neoplasm malignant stage unspecified	1	0.002%
Tooth deposit	1	0.002%
Tooth discolouration	1	0.002%
Tooth injury	1	0.002%
Tooth loss	1	0.002%
Total lung capacity abnormal	1	0.002%
Toxic nodular goitre	1	0.002%
Toxic shock syndrome	1	0.002%
Toxic shock syndrome streptococcal	1	0.002%
Tracheal cancer	1	0.002%
Tracheal deviation	1	0.002%
Tracheobronchitis	1	0.002%
Tri-iodothyronine increased	1	0.002%
Trichorrhexis	1	0.002%
Trigger finger	1	0.002%
Tumour flare	1	0.002%
Type I hypersensitivity	1	0.002%
Ulcer	1	0.002%
Ulnar nerve injury	1	0.002%
Urethral pain	1	0.002%
Urethritis	1	0.002%
Urinary casts	1	0.002%
Urinary hesitation	1	0.002%
Urine abnormality	1	0.002%
Uterine haemorrhage	1	0.002%
Vaginal infection	1	0.002%
Vaginal ulceration	1	0.002%
Vascular pain	1	0.002%
Vascular purpura	1	0.002%
Vasodilatation	1	0.002%
Vena cava injury	1	0.002%
Venous injury	1	0.002%

Vertebrobasilar insufficiency	1	0.002%
Vertigo positional	1	0.002%
Vestibular disorder	1	0.002%
Viral myocarditis	1	0.002%
Viral pericarditis	1	0.002%
Vital capacity abnormal	1	0.002%
Vitamin B12 increased	1	0.002%
Vitamin B6 deficiency	1	0.002%
Vitreous haemorrhage	1	0.002%
Vomiting projectile	1	0.002%
Waldenstrom's macroglobulinaemia recurrent	1	0.002%
Weight	1	0.002%
White blood cells urine positive	1	0.002%
X-ray abnormal	1	0.002%
Xanthopsia	1	0.002%
Xerophthalmia	1	0.002%
Xerosis	1	0.002%
Yellow skin	1	0.002%
Fibromyalgia	1	0.002%
Intestinal polyp	1	0.002%
Wound secretion	1	0.002%
Pericardial fibrosis	1	0.002%
Pneumopericardium	1	0.002%
Axillary pain	1	0.002%
Acute generalised exanthematous pustulosis	1	0.002%
Underweight	1	0.002%
Cytomegalovirus chorioretinitis	1	0.002%
Vertebral lesion	1	0.002%
Pityriasis lichenoides et varioliformis acuta	1	0.002%
Streptococcal sepsis	1	0.002%
Carotid artery occlusion	1	0.002%
Vascular pseudoaneurysm	1	0.002%
Axillary mass	1	0.002%
Vascular graft occlusion	1	0.002%
Oesophagitis ulcerative	1	0.002%
Anaemia of malignant disease	1	0.002%
Allergic sinusitis	1	0.002%
Visual brightness	1	0.002%
Red blood cell sedimentation rate increased	1	0.002%
Red blood cell sedimentation rate decreased	1	0.002%
Thermal burns of eye	1	0.002%
Lipodystrophy acquired	1	0.002%
Mouth injury	1	0.002%
Lip haemorrhage	1	0.002%
Gingival pruritus	1	0.002%
Lip blister	1	0.002%
Gingival ulceration	1	0.002%
Precancerous skin lesion	1	0.002%
Blood phosphorus decreased	1	0.002%

Gastrointestinal fungal infection	1	0.002%
Traumatic fracture	1	0.002%
Anal haemorrhage	1	0.002%
Douglas' abscess	1	0.002%
Quadriparesis	1	0.002%
Gallbladder polyp	1	0.002%
Metastases to pancreas	1	0.002%
Pubic pain	1	0.002%
Silent myocardial infarction	1	0.002%
Left ventricular hypertrophy	1	0.002%
Muscle tightness	1	0.002%
Breast discomfort	1	0.002%
Dry gangrene	1	0.002%
Intracranial hypotension	1	0.002%
Bradyphrenia	1	0.002%
Abulia	1	0.002%
Renal cancer metastatic	1	0.002%
Muscle strain	1	0.002%
Consciousness fluctuating	1	0.002%
Mediastinal cyst	1	0.002%
Anal inflammation	1	0.002%
Right ventricular hypertrophy	1	0.002%
Coronary angioplasty	1	0.002%
Electrocardiogram T wave abnormal	1	0.002%
Chronic gastrointestinal bleeding	1	0.002%
Carotid artery dissection	1	0.002%
Granulocytes maturation arrest	1	0.002%
Anastomotic leak	1	0.002%
Peripancreatic fluid collection	1	0.002%
Abscess limb	1	0.002%
Anorectal operation	1	0.002%
Prostate infection	1	0.002%
Red blood cells urine	1	0.002%
Band neutrophil count increased	1	0.002%
Urine leukocyte esterase positive	1	0.002%
Lymph gland infection	1	0.002%
Decreased ventricular afterload	1	0.002%
Polymerase chain reaction	1	0.002%
Radiation hepatitis	1	0.002%
Streptococcal bacteraemia	1	0.002%
Oral surgery	1	0.002%
Endodontic procedure	1	0.002%
Catheter site haemorrhage	1	0.002%
Drug ineffective for unapproved indication	1	0.002%
Madarosis	1	0.002%
Dyschezia	1	0.002%
Ureteritis	1	0.002%
SAPHO syndrome	1	0.002%
Wound haemorrhage	1	0.002%

Allergic cystitis	1	0.002%
Dysentery	1	0.002%
Gastrointestinal tract adenoma	1	0.002%
Metastases to retroperitoneum	1	0.002%
Metastases to trachea	1	0.002%
Bone marrow oedema	1	0.002%
Blood beta-D-glucan increased	1	0.002%
Acinetobacter infection	1	0.002%
Fusarium infection	1	0.002%
Glomerulonephropathy	1	0.002%
Cortical laminar necrosis	1	0.002%
Lip erosion	1	0.002%
Eyelid margin crusting	1	0.002%
Eye oedema	1	0.002%
Monocyte percentage increased	1	0.002%
Bronchial neoplasm	1	0.002%
Catheter site erythema	1	0.002%
Hepatopulmonary syndrome	1	0.002%
Urinary occult blood positive	1	0.002%
Systemic mycosis	1	0.002%
Dilatation intrahepatic duct acquired	1	0.002%
Gastric hypomotility	1	0.002%
Paranasal sinus discomfort	1	0.002%
Electrocardiogram repolarisation abnormality	1	0.002%
Large intestinal ulcer perforation	1	0.002%
Small intestinal ulcer perforation	1	0.002%
Detachment of retinal pigment epithelium	1	0.002%
Incisional drainage	1	0.002%
Gastric volvulus	1	0.002%
Fanconi syndrome acquired	1	0.002%
Vascular shunt	1	0.002%
Thoracic operation	1	0.002%
Adenocarcinoma pancreas	1	0.002%
Drug tolerance	1	0.002%
Drug tolerance decreased	1	0.002%
Perirectal abscess	1	0.002%
Cardiac flutter	1	0.002%
Nerve injury	1	0.002%
Catheter placement	1	0.002%
Crystal urine	1	0.002%
Epigastric discomfort	1	0.002%
Candida pneumonia	1	0.002%
Candida sepsis	1	0.002%
Therapeutic response delayed	1	0.002%
Arteriovenous graft thrombosis	1	0.002%
Splenic neoplasm malignancy unspecified	1	0.002%
Walking disability	1	0.002%
Non-alcoholic steatohepatitis	1	0.002%
Amimia	1	0.002%

Skin swelling	1	0.002%
Polypectomy	1	0.002%
Clonic convulsion	1	0.002%
Trichosporon infection	1	0.002%
Traumatic haemorrhage	1	0.002%
Arthritis bacterial	1	0.002%
Abscess neck	1	0.002%
Bronchopneumopathy	1	0.002%
Extraocular muscle disorder	1	0.002%
IIIrd nerve disorder	1	0.002%
Limb deformity	1	0.002%
Infusion site phlebitis	1	0.002%
Infusion site pruritus	1	0.002%
Sigmoidectomy	1	0.002%
CSF white blood cell count increased	1	0.002%
CSF white blood cell count decreased	1	0.002%
Sepsis syndrome	1	0.002%
Red cell distribution width abnormal	1	0.002%
Haemorrhagic tumour necrosis	1	0.002%
Listeria encephalitis	1	0.002%
Stenotrophomonas infection	1	0.002%
Skin candida	1	0.002%
Superior mesenteric artery syndrome	1	0.002%
Small intestine carcinoma	1	0.002%
Intestinal resection	1	0.002%
IIIrd nerve paresis	1	0.002%
Cardiac discomfort	1	0.002%
Dental discomfort	1	0.002%
Enterococcal sepsis	1	0.002%
Disorder of globe	1	0.002%
Regressive behaviour	1	0.002%
Increased viscosity of bronchial secretion	1	0.002%
Pharyngeal lesion	1	0.002%
Throat lesion	1	0.002%
Corneal transplant	1	0.002%
Myoclonic epilepsy	1	0.002%
Mucosal discolouration	1	0.002%
Infusion site reaction	1	0.002%
Vascular neoplasm	1	0.002%
Lip injury	1	0.002%
Rectal cancer metastatic	1	0.002%
Breast cancer metastatic	1	0.002%
Thyroxine free decreased	1	0.002%
Necrotising oesophagitis	1	0.002%
Pneumonia necrotising	1	0.002%
Haematoma muscle	1	0.002%
Adenomyosis	1	0.002%
Emphysematous cystitis	1	0.002%
Joint tuberculosis	1	0.002%

Growth hormone deficiency	1	0.002%
Phlebitis infective	1	0.002%
Emphysematous cholecystitis	1	0.002%
Gaze palsy	1	0.002%
Body height decreased	1	0.002%
Device failure	1	0.002%
Alcoholic pancreatitis	1	0.002%
Enterostomy	1	0.002%
Pyopneumothorax	1	0.002%
Spinal cord paralysis	1	0.002%
Neuroendocrine carcinoma	1	0.002%
Daydreaming	1	0.002%
Intervertebral disc operation	1	0.002%
Bronchial polyp	1	0.002%
Rehabilitation therapy	1	0.002%
Dependence on respirator	1	0.002%
Post procedural diarrhoea	1	0.002%
Choledocholithotomy	1	0.002%
Bipolar disorder	1	0.002%
Joint arthroplasty	1	0.002%
Infective thrombosis	1	0.002%
Metabolic disorder	1	0.002%
Neutrophilic dermatosis	1	0.002%
Nephrogenic anaemia	1	0.002%
Asteatosis	1	0.002%
Eosinophil percentage abnormal	1	0.002%
Neutrophil percentage abnormal	1	0.002%
Subendocardial ischaemia	1	0.002%
Pulseless electrical activity	1	0.002%
Clostridium colitis	1	0.002%
Dysplasia	1	0.002%
Parakeratosis	1	0.002%
Nuchal rigidity	1	0.002%
Ballismus	1	0.002%
Anti-platelet antibody positive	1	0.002%
Hypoperfusion	1	0.002%
Pulmonary hilum mass	1	0.002%
Oesophageal infection	1	0.002%
Enterobacter bacteraemia	1	0.002%
Fungal sepsis	1	0.002%
Pseudomonal sepsis	1	0.002%
Serratia sepsis	1	0.002%
Haemobilia	1	0.002%
Extradural neoplasm	1	0.002%
Intestinal mass	1	0.002%
Gastrointestinal ischaemia	1	0.002%
Biloma	1	0.002%
Blue toe syndrome	1	0.002%
Cardiac massage	1	0.002%

Oxygen consumption decreased	1	0.002%
Oxygen consumption increased	1	0.002%
Acquired diaphragmatic eventration	1	0.002%
Early satiety	1	0.002%
Adrenal haematoma	1	0.002%
Sedative therapy	1	0.002%
Pleuropericarditis	1	0.002%
Parophthalmia	1	0.002%
Wound infection staphylococcal	1	0.002%
Skin neoplasm excision	1	0.002%
Wound infection pseudomonas	1	0.002%
Abdominal cavity drainage	1	0.002%
Lung hyperinflation	1	0.002%
Secondary hypogonadism	1	0.002%
Stroke in evolution	1	0.002%
CSF test abnormal	1	0.002%
Anti-GAD antibody positive	1	0.002%
Haemorrhagic ascites	1	0.002%
Infusion site rash	1	0.002%
Liver function test	1	0.002%
Antineutrophil cytoplasmic antibody increased	1	0.002%
Hypergastrinaemia	1	0.002%
Skin neoplasm bleeding	1	0.002%
Endocrine ophthalmopathy	1	0.002%
Therapeutic product ineffective	1	0.002%
Hepatic enzyme decreased	1	0.002%
Myxoedema coma	1	0.002%
Joint injury	1	0.002%
Choroidal neovascularisation	1	0.002%
General symptom	1	0.002%
Adenovirus infection	1	0.002%
Appendix disorder	1	0.002%
Arterial disorder	1	0.002%
Arthritis infective	1	0.002%
Cardiac myxoma	1	0.002%
Cardiac neoplasm malignant	1	0.002%
Cardiac operation	1	0.002%
Central nervous system infection	1	0.002%
Clostridial infection	1	0.002%
Colon neoplasm	1	0.002%
Communication disorder	1	0.002%
Cranial nerve injury	1	0.002%
Enterococcal infection	1	0.002%
Eye injury	1	0.002%
Ocular toxicity	1	0.002%
Ligament disorder	1	0.002%
Limb operation	1	0.002%
Lymphoproliferative disorder	1	0.002%
Intervertebral disc degeneration	1	0.002%

Investigation abnormal	1	0.002%
Jaw disorder	1	0.002%
Joint lock	1	0.002%
Legionella infection	1	0.002%
Mantle cell lymphoma	1	0.002%
Mucosal haemorrhage	1	0.002%
Peritoneal disorder	1	0.002%
Peritoneal neoplasm	1	0.002%
Pleural neoplasm	1	0.002%
Pneumococcal infection	1	0.002%
Skin injury	1	0.002%
Skull fracture	1	0.002%
Soft tissue disorder	1	0.002%
Spinal cord disorder	1	0.002%
Victim of crime	1	0.002%
Gastrointestinal ulcer	1	0.002%
Postoperative wound complication	1	0.002%
Proteus infection	1	0.002%
Post procedural fistula	1	0.002%
Rhinovirus infection	1	0.002%
Salivary gland neoplasm	1	0.002%
Serratia infection	1	0.002%
Lip and/or oral cavity cancer	1	0.002%
Skin degenerative disorder	1	0.002%
Radiotherapy to lung	1	0.002%
Sensation of foreign body	1	0.002%
Mass excision	1	0.002%
Troponin	1	0.002%
Aortic valve disease	1	0.002%
Cardiac fibrillation	1	0.002%
Respiratory syncytial virus infection	1	0.002%
Aortic surgery	1	0.002%
Artery dissection	1	0.002%
Autonomic neuropathy	1	0.002%
Brain operation	1	0.002%
Cerebellar tumour	1	0.002%
Chondropathy	1	0.002%
Colectomy	1	0.002%
Conjunctivitis bacterial	1	0.002%
Deformity thorax	1	0.002%
Dental operation	1	0.002%
Enterocolitis viral	1	0.002%
Fat necrosis	1	0.002%
Obstruction	1	0.002%
Occult blood	1	0.002%
Oesophageal neoplasm	1	0.002%
Pancreatic neoplasm	1	0.002%
Paracentesis	1	0.002%
Parainfluenzae virus infection	1	0.002%

Pulmonary function test decreased	1	0.002%
Scan adrenal gland abnormal	1	0.002%
Lymph nodes scan abnormal	1	0.002%
Fracture treatment	1	0.002%
Gamma radiation therapy	1	0.002%
Gastrectomy	1	0.002%
Glomerulosclerosis	1	0.002%
Hernia repair	1	0.002%
Immune agglutinins	1	0.002%
Lung infiltration malignant	1	0.002%
Lung operation	1	0.002%
Lymphocytic infiltration	1	0.002%
Pharyngeal operation	1	0.002%
Physical examination abnormal	1	0.002%
Respiratory tract infection viral	1	0.002%
Syphilis	1	0.002%
Thyroidectomy	1	0.002%
Tooth extraction	1	0.002%
Vascular access complication	1	0.002%
Vitamin B12 abnormal	1	0.002%
Vitamin D decreased	1	0.002%
Moraxella infection	1	0.002%
Shoulder operation	1	0.002%
Spinal cord neoplasm	1	0.002%
Urinary tract infection pseudomonal	1	0.002%
Urinary tract infection staphylococcal	1	0.002%
Heparin-induced thrombocytopenia	1	0.002%
Increased bronchial secretion	1	0.002%
Erosive duodenitis	1	0.002%
Tracheal haemorrhage	1	0.002%
Scleroderma renal crisis	1	0.002%
Spider vein	1	0.002%
Increased upper airway secretion	1	0.002%
Urethritis noninfective	1	0.002%
Cyclic vomiting syndrome	1	0.002%
Facial spasm	1	0.002%
Listeria sepsis	1	0.002%
Post procedural inflammation	1	0.002%
Anaphylactoid shock	1	0.002%
Post procedural haematoma	1	0.002%
Insulin C-peptide abnormal	1	0.002%
Interleukin level increased	1	0.002%
Metamorphopsia	1	0.002%
Charles Bonnet syndrome	1	0.002%
Venous stent insertion	1	0.002%
Gene mutation identification test positive	1	0.002%
Body temperature fluctuation	1	0.002%
Allergic respiratory disease	1	0.002%
Spondylolisthesis	1	0.002%

Metastatic squamous cell carcinoma	1	0.002%
Lung neoplasm surgery	1	0.002%
Radiotherapy to lymph nodes	1	0.002%
Hippocampal sclerosis	1	0.002%
Pyramidal tract syndrome	1	0.002%
Post procedural oedema	1	0.002%
Cerebral artery stenosis	1	0.002%
Uraemic gastropathy	1	0.002%
Accidental death	1	0.002%
Life expectancy shortened	1	0.002%
Implant site pain	1	0.002%
Renal ischaemia	1	0.002%
Sleep disorder due to a general medical condition	1	0.002%
Metastatic gastric cancer	1	0.002%
Orthostatic intolerance	1	0.002%
Vascular stent thrombosis	1	0.002%
Multiple system atrophy	1	0.002%
Penile erosion	1	0.002%
Iatrogenic infection	1	0.002%
Eosinophilic oesophagitis	1	0.002%
Skin haemorrhage	1	0.002%
Human papilloma virus test positive	1	0.002%
Muscle swelling	1	0.002%
Lip exfoliation	1	0.002%
Oral mucosal exfoliation	1	0.002%
Corneal exfoliation	1	0.002%
Cell marker	1	0.002%
Cranial operation	1	0.002%
Gene mutation	1	0.002%
Iliac artery occlusion	1	0.002%
Portal venous gas	1	0.002%
Breath sounds	1	0.002%
Mycobacterium abscessus infection	1	0.002%
Pus in stool	1	0.002%
Vulvovaginal mycotic infection	1	0.002%
Pulmonary arterial hypertension	1	0.002%
Age-related macular degeneration	1	0.002%
Matrix metalloproteinase-3 increased	1	0.002%
Tumour perforation	1	0.002%
Head titubation	1	0.002%
Thromboembolectomy	1	0.002%
Rectal fissure	1	0.002%
Necrotising retinitis	1	0.002%
Nutritional condition abnormal	1	0.002%
Immune reconstitution inflammatory syndrome	1	0.002%
Herpes simplex pneumonia	1	0.002%
Latent tuberculosis	1	0.002%
Gastrointestinal endoscopic therapy	1	0.002%
Pancreatitis viral	1	0.002%

Cystitis bacterial	1	0.002%
Acrokeratosis paraneoplastica	1	0.002%
Cancer in remission	1	0.002%
Post procedural hypothyroidism	1	0.002%
Pelvic fluid collection	1	0.002%
Brachial plexopathy	1	0.002%
Infusion site discolouration	1	0.002%
Infusion site paraesthesia	1	0.002%
Cerebral arteriosclerosis	1	0.002%
Eosinophilic bronchitis	1	0.002%
Percutaneous coronary intervention	1	0.002%
Stoma site inflammation	1	0.002%
Nephritic syndrome	1	0.002%
Peripheral nerve infection	1	0.002%
Soft tissue necrosis	1	0.002%
Fistula of small intestine	1	0.002%
Vessel puncture site haematoma	1	0.002%
Pneumobilia	1	0.002%
Hyponatraemic encephalopathy	1	0.002%
Bone contusion	1	0.002%
Interleukin-2 receptor increased	1	0.002%
Therapy interrupted	1	0.002%
Staphylococcal skin infection	1	0.002%
Testicular oedema	1	0.002%
Stomach mass	1	0.002%
Bronchial secretion retention	1	0.002%
Aorto-oesophageal fistula	1	0.002%
Alveolar osteitis	1	0.002%
Retroperitoneal lymphadenopathy	1	0.002%
Basal ganglia haemorrhage	1	0.002%
Monocyte percentage abnormal	1	0.002%
Post procedural infection	1	0.002%
Left atrial dilatation	1	0.002%
Surfactant protein increased	1	0.002%
Oral mucosal erythema	1	0.002%
Cerebral microangiopathy	1	0.002%
Apparent death	1	0.002%
Cytogenetic abnormality	1	0.002%
Inguinal hernia strangulated	1	0.002%
Quality of life decreased	1	0.002%
Peripheral nerve lesion	1	0.002%
Toxic neuropathy	1	0.002%
Skin plaque	1	0.002%
Mean cell haemoglobin concentration abnormal	1	0.002%
Vascular graft	1	0.002%
Blood creatine phosphokinase decreased	1	0.002%
Pharyngeal abscess	1	0.002%
Pulmonary air leakage	1	0.002%
Post-thoracotomy pain syndrome	1	0.002%

Dementia with Lewy bodies	1	0.002%
Oropharyngeal blistering	1	0.002%
Injection site vasculitis	1	0.002%
Potentiating drug interaction	1	0.002%
Occipital neuralgia	1	0.002%
Foreign body reaction	1	0.002%
Gastroenteritis norovirus	1	0.002%
Cardiorenal syndrome	1	0.002%
Anorectal discomfort	1	0.002%
Gastrointestinal bacterial infection	1	0.002%
Hepatitis B DNA increased	1	0.002%
Drain removal	1	0.002%
Brain stem stroke	1	0.002%
Splenic embolism	1	0.002%
Pulmonary vein occlusion	1	0.002%
Overlap syndrome	1	0.002%
Ligament pain	1	0.002%
Limb reconstructive surgery	1	0.002%
Frontotemporal dementia	1	0.002%
Kounis syndrome	1	0.002%
Liquid product physical issue	1	0.002%
Product label confusion	1	0.002%
Product quality issue	1	0.002%
Hashimoto's encephalopathy	1	0.002%
Bronchial metaplasia	1	0.002%
Thoracic cavity lavage	1	0.002%
Acquired Von Willebrand's disease	1	0.002%
Paraneoplastic encephalomyelitis	1	0.002%
Mesenteritis	1	0.002%
Spinal column injury	1	0.002%
Device related sepsis	1	0.002%
Injury associated with device	1	0.002%
Inflammatory marker increased	1	0.002%
Patient-device incompatibility	1	0.002%
Product complaint	1	0.002%
Bacillus test positive	1	0.002%
Acinetobacter test positive	1	0.002%
Helicobacter test positive	1	0.002%
Nocardia test positive	1	0.002%
Hepatitis C virus test positive	1	0.002%
Foreign body	1	0.002%
Streptococcal urinary tract infection	1	0.002%
Polyomavirus test positive	1	0.002%
Norovirus test positive	1	0.002%
Pneumocystis test positive	1	0.002%
Hypoxic-ischaemic encephalopathy	1	0.002%
Nail bed disorder	1	0.002%
Tumour rupture	1	0.002%
False positive investigation result	1	0.002%

Respiratory muscle weakness	1	0.002%
Gastrointestinal tract irritation	1	0.002%
Cerebral small vessel ischaemic disease	1	0.002%
VIth nerve paresis	1	0.002%
Blood pressure difference of extremities	1	0.002%
Infectious thyroiditis	1	0.002%
Suspected counterfeit product	1	0.002%
Hepatitis B core antibody positive	1	0.002%
Hepatitis B surface antibody positive	1	0.002%
Infected dermal cyst	1	0.002%
Chronic eosinophilic rhinosinusitis	1	0.002%
Susac's syndrome	1	0.002%
Autoimmune retinopathy	1	0.002%
N-terminal prohormone brain natriuretic peptide increased	1	0.002%
RET gene mutation	1	0.002%
Bandaemia	1	0.002%
Stiff person syndrome	1	0.002%
Skin wound	1	0.002%
Computerised tomogram kidney abnormal	1	0.002%
Enzyme level increased	1	0.002%
Neuromyotonia	1	0.002%
Peritumoural oedema	1	0.002%
Oesophageal motility disorder	1	0.002%
Anti-epithelial antibody positive	1	0.002%
Anti-ganglioside antibody positive	1	0.002%
Anti-zinc transporter 8 antibody positive	1	0.002%
Peripheral artery stenosis	1	0.002%
Haemangioma of bone	1	0.002%
Rectal injury	1	0.002%
Microvascular coronary artery disease	1	0.002%
Uveitic glaucoma	1	0.002%
Pancreatic steatosis	1	0.002%
Drain placement	1	0.002%
Vascular wall hypertrophy	1	0.002%
Sensitivity to weather change	1	0.002%
Birdshot chorioretinopathy	1	0.002%
Meniscus injury	1	0.002%
Hepatic cancer	1	0.002%
Hepatic cancer recurrent	1	0.002%
Hepatocellular carcinoma	1	0.002%
Hyponatraemic seizure	1	0.002%
Reversible cerebral vasoconstriction syndrome	1	0.002%
Brenner tumour	1	0.002%
White blood cell analysis abnormal	1	0.002%
Alveolar lung disease	1	0.002%
Eye contusion	1	0.002%
Cardiac contusion	1	0.002%
Haemophobia	1	0.002%
Instillation site paraesthesia	1	0.002%

Colonic abscess	1	0.002%
Obstructive shock	1	0.002%
Pneumocystis jirovecii infection	1	0.002%
Ophthalmic herpes simplex	1	0.002%
Intentional underdose	1	0.002%
Hypothalamic pituitary adrenal axis suppression	1	0.002%
Trigeminal neuritis	1	0.002%
Oesophageal compression	1	0.002%
Papillary cystadenoma lymphomatosum	1	0.002%
Diabetic metabolic decompensation	1	0.002%
Diaphragmatic spasm	1	0.002%
Renal transplant failure	1	0.002%
Tendon discomfort	1	0.002%
Hyperfibrinolysis	1	0.002%
Noninfective gingivitis	1	0.002%
Disease susceptibility	1	0.002%
Expired product administered	1	0.002%
Accidental underdose	1	0.002%
Nasal herpes	1	0.002%
Hyperferritinaemia	1	0.002%
Graft versus host disease in gastrointestinal tract	1	0.002%
Coronary vascular graft occlusion	1	0.002%
Body temperature abnormal	1	0.002%
Cholangitis infective	1	0.002%
Anal hypoaesthesia	1	0.002%
BRAF V600E mutation positive	1	0.002%
Autoimmune uveitis	1	0.002%
Application site joint discomfort	1	0.002%
Infusion site discharge	1	0.002%
Pancreatic toxicity	1	0.002%
Unmasking of previously unidentified disease	1	0.002%
Lung perforation	1	0.002%
Home care	1	0.002%
Steatohepatitis	1	0.002%
Radiotherapy to oral cavity	1	0.002%
Neurological rehabilitation	1	0.002%
Compulsive cheek biting	1	0.002%
Combined pulmonary fibrosis and emphysema	1	0.002%
Radiotherapy to mediastinum	1	0.002%
Marginal zone lymphoma	1	0.002%
Acute motor axonal neuropathy	1	0.002%
External compression headache	1	0.002%
Open globe injury	1	0.002%
Pharyngeal paraesthesia	1	0.002%
Bacterial colitis	1	0.002%
Breast conserving surgery	1	0.002%
Dose calculation error	1	0.002%
Cerebral vascular occlusion	1	0.002%
Systemic immune activation	1	0.002%

Bickerstaff's encephalitis	1	0.002%
Tumour marker abnormal	1	0.002%
Systemic infection	1	0.002%
Penetrating aortic ulcer	1	0.002%
Hemianaesthesia	1	0.002%
Mite allergy	1	0.002%
Cytokine abnormal	1	0.002%
Pulmonary resection	1	0.002%
Procedural pneumothorax	1	0.002%
Vascular access site complication	1	0.002%
Medical procedure	1	0.002%
C3 glomerulopathy	1	0.002%
Procalcitonin decreased	1	0.002%
Obstructive nephropathy	1	0.002%
Oesophagopleural fistula	1	0.002%
Retinal thickening	1	0.002%
Cerebral radiation injury	1	0.002%
Nervous system cyst	1	0.002%
Diffuse uveal melanocytic proliferation	1	0.002%
Anti Kell antibody test positive	1	0.002%
Computerised tomogram liver abnormal	1	0.002%
Contraindicated product administered	1	0.002%
Lymphocytic oesophagitis	1	0.002%
Thyroid gland injury	1	0.002%
Parasitic pneumonia	1	0.002%
MNS system antibodies positive	1	0.002%
Autoimmune endocrine disorder	1	0.002%
Angiopathic neuropathy	1	0.002%
Autoimmune pericarditis	1	0.002%
Cerebellar stroke	1	0.002%
Retinal aneurysm rupture	1	0.002%
Segmental diverticular colitis	1	0.002%
Housebound	1	0.002%
Psychotic symptom	1	0.002%
Product use complaint	1	0.002%
Drain site complication	1	0.002%
Obstructive pancreatitis	1	0.002%
Pneumonia acinetobacter	1	0.002%
Pulmonary tumour thrombotic microangiopathy	1	0.002%
Chronic rhinosinusitis with nasal polyps	1	0.002%
Euglycaemic diabetic ketoacidosis	1	0.002%
CSF volume decreased	1	0.002%
Product dispensing error	1	0.002%
Pulmonary nocardiosis	1	0.002%
Anti-HLA antibody test positive	1	0.002%
Herpes zoster reactivation	1	0.002%
Gastrointestinal vascular malformation haemorrhagic	1	0.002%
Autoimmune lung disease	1	0.002%
Sleep deficit	1	0.002%

Nervous system injury	1	0.002%
Failure to suspend medication	1	0.002%
Paracancerous pneumonia	1	0.002%
Hanging	1	0.002%
Lithiasis	1	0.002%
Digestive enzyme abnormal	1	0.002%
Transdifferentiation of neoplasm	1	0.002%
Central nervous system injury	1	0.002%
Stiff tongue	1	0.002%
Complicated appendicitis	1	0.002%
Intercepted product administration error	1	0.002%
Incorrect product dosage form administered	1	0.002%
Wrong patient received product	1	0.002%
Serpiginous choroiditis	1	0.002%
Intercepted product prescribing error	1	0.002%
Focal myositis	1	0.002%
Nephritis bacterial	1	0.002%
Intracranial infection	1	0.002%
Therapeutic product effect increased	1	0.002%
Internal carotid artery deformity	1	0.002%
Phantom limb syndrome	1	0.002%
Musculoskeletal toxicity	1	0.002%
Muscle strength abnormal	1	0.002%
AST/ALT ratio abnormal	1	0.002%
Achromobacter infection	1	0.002%
Genital disorder	1	0.002%
Post procedural erythema	1	0.002%
Cerebral venous sinus thrombosis	1	0.002%
SJS-TEN overlap	1	0.002%
Drug effect less than expected	1	0.002%
Adenocarcinoma metastatic	1	0.002%
Clinical death	1	0.002%
Meningeal thickening	1	0.002%
Internal device exposed	1	0.002%
Discouragement	1	0.002%
Optic perineuritis	1	0.002%
Coronavirus test negative	1	0.002%
Microscopic enteritis	1	0.002%
TP53 gene mutation	1	0.002%
HRD gene mutation assay positive	1	0.002%
Urinary occult blood	1	0.002%
Troponin I abnormal	1	0.002%
Sarcoid-like reaction	1	0.002%
Treatment delayed	1	0.002%
Cancer with a high tumour mutational burden	1	0.002%
Magnetic resonance imaging head	1	0.002%
Magnetic resonance imaging head abnormal	1	0.002%
Renal-limited thrombotic microangiopathy	1	0.002%
Necrotic lymphadenopathy	1	0.002%

Immune-mediated neurological disorder	1	0.002%
Cytokine increased	1	0.002%
Tooth restoration	1	0.002%
Anti-thyroid antibody increased	1	0.002%
Tertiary adrenal insufficiency	1	0.002%
Radioimmunotherapy	1	0.002%
Eastern Cooperative Oncology Group performance status abnormal	1	0.002%
Hyperglycaemic crisis	1	0.002%
